# The Perioperative NonaGEnaRIan And cenTenarian suRgICal (GERIATRIC) Risk Stratification Tool

**DOI:** 10.1097/AS9.0000000000000524

**Published:** 2024-11-18

**Authors:** Laurence Weinberg, Dong Kyu Lee, Luke Fletcher, Bobby Ou Yang, Jadon Karp, Anoop N Koshy, Ranjan Guha, Hugh Slifirski, Michael R D’Silva, Rinaldo Bellomo, Leonid Churilov

**Affiliations:** From the * Department of Anesthesia, Austin Health, Heidelberg, Australia; † Department of Critical Care, The University of Melbourne, Austin Health, Heidelberg, Australia; ‡ Department of Anesthesiology and Pain Medicine, Dongguk University Ilsan Hospital, Goyang, Republic of Korea; § Data Analytics Research and Evaluation (DARE) Centre, Austin Health, Heidelberg, Australia; ‖ Department of Cardiology, Austin Health, Heidelberg, Australia; ¶ Department of Intensive Care, Austin Hospital, Melbourne, Australia; # Department of Medicine, Royal Melbourne Hospital, Melbourne Brain Centre at Royal Melbourne Hospital, Melbourne Medical School, Faculty of Medicine, The University of Melbourne; Melbourne, Australia

**Keywords:** anesthesia, centenarian, nonagenarian, risk, surgery

## Abstract

**Objective:**

To develop age-appropriate nonaGEnaRIan And cenTenarian suRgICal (GERIATRIC) risk tool for classifying patients who may or may not develop postoperative complications or die within their index hospital admission.

**Background:**

There are no validated perioperative risk stratification tools for use in nonagenarian and centenarian patients—people aged 90 to 99 years and >100 years.

**Methods:**

In this retrospective observational study, nonagenarians and centenarians undergoing any surgical procedure were profiled. Surgery severity was stratified, and the incidence and grade of postoperative complications were recorded. Multivariable logistic regression analysis was performed on a training cohort, followed by calibration on a validation cohort, followed by performance evaluation on a testing cohort. The discriminative accuracy was compared to that of the age-adjusted Charlson Comorbidity Index for each outcome. The primary outcome was the ability of the risk stratification tool to effectively classify patients into those who may or may not experience a postoperative complications or mortality within their index hospital stay.

**Results:**

A total of 3085 patients were enrolled. The GERIATRIC risk tool had good discriminative accuracy for any postoperative complication [area under the receiver operating characteristic curves (AUROC), 0.857; 95% CI = 0.824–0.890] and any severe postoperative complication (AUROC, 0.833; 95% CI = 0.793–0.874), and fair discriminative accuracy for in-hospital mortality (AUROC, 0.780; 95% CI = 0.668–0.893).

**Conclusions:**

Compared to the age-adjusted Charlson Comorbidity Index, The GERIATRIC risk tool was accurate in classifying patients into those who may or may not experience severe complications or die during their index admission. The tool can be used to assist perioperative clinicians with shared decision-making and short-term prognostication.

## Graphical abstract

**Figure:**
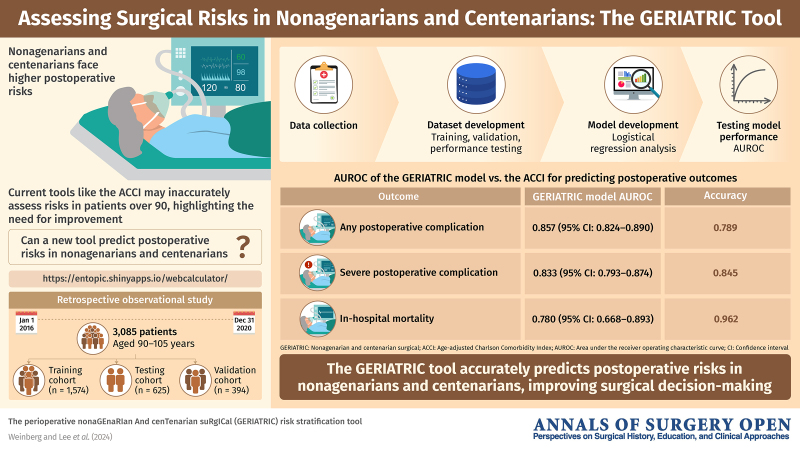

## INTRODUCTION

Nonagenarians and centenarians are a rapidly growing segment of the population. Over the last 20 years, each age group experienced a growth rate of 185% and 215%, respectively.^1,2^ In keeping with such demographic changes, the number of high-risk operations in such patients has also increased.^3,4^ However, these patients are at greater risk of adverse postoperative outcomes,^5^ and advanced age remains a significant risk factor for postoperative mortality.^6^

Perioperative care for nonagenarians and centenarians is complex, and the decision to operate is challenging due to the high incidence of complications.^7^ Accordingly, risk stratification tools have been used to predict the risks of adverse postoperative outcomes in these patients. The Charlson Comorbidity Index (CCI) is a widely used and validated scoring system for predicting 1-year mortality in adult hospitalized patients. It is universally applied by both point-of-care clinicians and clinical researchers undertaking longitudinal studies.^8^ The CCI has been further refined by incorporating age into the risk model, the age-adjusted Charlson Comorbidity Index (ACCI) (Supplemental Table 1, http://links.lww.com/AOSO/A429),^9^ with the highest age group category being ≥71 years old. While the ACCI has been validated in predicting various short- and long-term outcomes across different surgical,^10–16^ it is unknown whether the ACCI is accurate when applied to surgical patients aged greater than 90 years, and more sophisticated risk stratification tools appear desirable. The ACCI may disproportionately elevate risk scores for nonagenarians and place excessive weight on chronological age over comorbidities, potentially leading to overly conservative treatment recommendations. Moreover, the ACCI may not fully capture the elevated risks associated with comorbidities such as anemia, congestive heart failure, electrolyte abnormalities, or the increased perioperative risks of high-risk surgeries. Therefore, a clinical tool that more accurately reflects the higher complication rates in such patients may provide a clearer picture of surgical risk.

This study aimed to develop an age-appropriate nonaGEnaRIan And cenTenarian suRgICal (GERIATRIC) risk stratification tool for adverse postoperative outcomes. We hypothesized that the GERIATRIC risk stratification tool would be more accurate than the ACCI in appropriately classifying patients who may or may not develop a postoperative complication or die within their index hospital admission.

## METHODS

### Study Design

This was a single-center cohort study with retrospective data collection of nonagenarians and centenarians who underwent any surgical or interventional procedures. The study was approved by the Human Research Ethics Committee of Austin Health (No. 21/Austin/30); written informed consent was waived because de-identified retrospective data were used. This study was registered with the Australian and New Zealand Clinical Trials Registry (No. CTR12622000170729, see www.anzctr.com.au). We followed the Transparent Reporting of a Multivariable Prediction Model for Individual Prognosis or Diagnosis reporting guidelines for risk prediction model development and validation.^10^

### Data Sources and Processing

The study population consisted of nonagenarians and centenarians who underwent surgery at Austin Health between January 1, 2016, and December 31, 2020. Austin Health is a university teaching hospital in Australia with a high volume of surgeries performed annually across 2 campuses and multiple subspecialties. All operative and anesthesia techniques across all surgical specialties were included, including endoscopic procedures; superficial skin surgery under local anesthesia; radiological intervention requiring anesthesia support; and cataract surgery under topical, regional, or general anesthesia. De-identified data were collected from the electronic medical records (Cerner Millenium, Kansas). A team of 3 experienced clinicians reviewed the records, and each clinician cross-checked the collected data.

### Data Collected

We collected patients’ demographic data, detailed ACCI components, perioperative laboratory and surgical data, and requirements for intensive care unit (ICU) admission. We stratified the severity of surgery based on a modified Johns Hopkins classification^11,12^ into “low-risk” (Johns Hopkins category Classes I and II), “moderate-risk” (Johns Hopkins Class III), and “high-risk” groups (Johns Hopkins category Classes IV and V; see Supplemental Table 2, http://links.lww.com/AOSO/A429).

We collected the occurrence of postoperative complications during the index hospital admission and in-hospital mortality. Postoperative complications were defined as any deviation from the normal expected postoperative course during the index admission, as guided by the European Perioperative Clinical Outcome definitions.^13^ The severity of complications was graded according to the Clavien–Dindo (CVD) system,^14,15^ a validated classification system that categorizes complication severity according to the level of treatment required.

### Key Outcomes

The key outcomes for the GERIATRIC risk tool included:

1. The development of any postoperative complications (CVD, I– V) within the index hospital admission;
2. The development of any severe complication requiring interventional, intensive, or high-dependency care (CVD, III and IV) within the index hospital admission;
3. In-hospital mortality (CVD, V).

### Datasets for the Development of GERIATRIC Risk Models

We separated the full dataset into 3 components: training, validation, and performance testing. We randomly selected cases using Bernoulli distribution at a ratio of 8:2. Consequently, we considered 80% of the cases as training and validation datasets and 20% of the cases were utilized for performance testing. Among the cases selected for training and validation, we used 80% of patients for model training and the remaining 20% for validation.

### GERIATRIC RISK MODEL INPUT PARAMETER SCREENING

We first evaluated the association between 58 preoperative and demographic variables—including the individual components of the ACCI as well as other clinically meaningful variables—and each outcome among the training dataset using Spearman’s correlation analysis. If the correlation was statistically significant, we considered that parameter as an input parameter for the models. The handling of outliers and missing values are reported in Supplemental File 2, http://links.lww.com/AOSO/A430.^16,17^

### Development of Risk Stratification Models for Outcomes

Risk stratification models for outcomes were developed using logistic regression analysis. The final GERIATRIC model was selected using the Akaike Information Criterion in a stepwise algorithm on the training dataset. We evaluated the presence of influential points using a visual check of Cook’s distance and standardized residuals. The primary aim was to evaluate the ability of the risk stratification tool to effectively classify patients into those who may or may not be experiencing the event of interest (discrimination ability). Secondary aims were to evaluate whether the risk stratification tool could correctly predict individual patient probabilities of experiencing such events (calibration ability).

### Evaluation of Model Performance

The estimated model performance on the training dataset was evaluated using the area under the receiver operating characteristic curves (AUROC). The AUROC estimation followed an empirical approach, and the equality of the AUROC was tested with a 95% confidence interval (CI) using the DeLong algorithm. We considered discrimination as good when the AUROC was greater than 0.80. Based on a rough classifying system, the AUROC can be interpreted as follows: 0.9–1.0, excellent; 0.8–0.9, good; 0.7–0.8, fair; and 0.6–0.7, poor. We performed AUROC curve analysis using the R package pROC.^18^ The optimal cutoff points on the curve were identified using the Youden index.

### Evaluation of Discrimination and Calibration

The selected model was then applied to the validation dataset to evaluate discrimination and calibration. The discrimination ability was evaluated using AUROC as described above. We examined calibration using Bier score and Spiegelhalter’s z-test, and visually checked the calibration plot to assess the agreement between the observed result and predicted probability. When the results of the calibration analysis indicate the need for re-calibrations, the isoregression and the Platt-scaling methods were applied. Calibration analysis was conducted using R package rms.^19,20^ Finally, the estimated models were evaluated with the performance testing dataset for the discrimination and calibrations.

### Statistical Analysis Principles, Reproducibility, and Data Presentation

Statistical analysis was performed using IBM SPSS Statistics for Windows, version 23 (IBM Corp., 2015, Armonk, NY) and R, version 4.2.3 (R Development Core Team, Vienna, Austria, 2023). A detailed description of the development of the GERAIATRIC tool is presented in Supplemental Text, http://links.lww.com/AOSO/A431. The final GERIATRIC model is freely available for clinical use as a web-based calculator at https://entopic.shinyapps.io/webcalculator/.

Data are presented as mean ± standard deviation or as median [interquartile range (IQR), (minimum: maximum)] for continuous variables and as number (percentage) for categorical variables. We tested all continuous variables for normality using a quantile–quantile plot. When a variable violated the normality assumption, we used nonparametric statistical methods. Statistically inferred results are described with respective 95% CI and *P* values. We considered a two-tailed *P* value below 0.05 as indicative of statistical significance.

## RESULTS

### Patient Cohort

**FIGURE 1.**
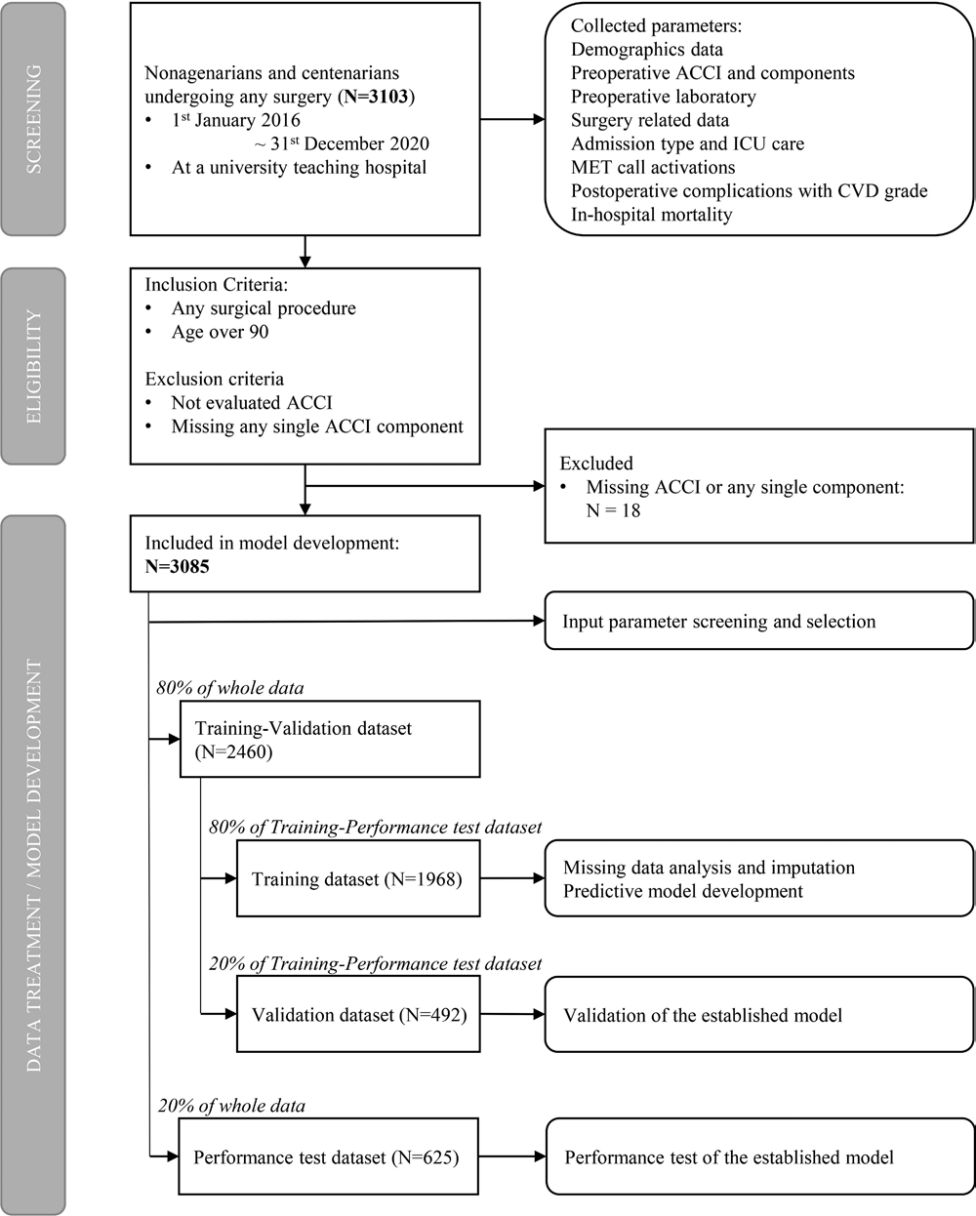
Risk prediction model development flow diagram. CVD indicates Clavien–Dindo postoperative surgical complication grade; MET, Medical emergency team call.

During the study period, 3103 patients were screened. Among these patients, 18 were excluded because of incomplete medical records. A total of 3085 patients were included, of which 1968 patients were utilized for training, 492 patients were utilized for validation, and 625 patients were reserved for performance testing (Fig. 1).

Among the full dataset, the mean age was 92.3 ± 2.4 years (90–105 years), with 31 patients (1.0%) aged ≥100 years. Females accounted for 49.7% of the cohort. Patient characteristics and laboratory findings of the nonagenarian and centenarian cohort are summarized in Table 1. Nearly all patients (2976 patients, 96.5%) had comorbidities. The median ACCI score was 5 (5–6) (5:14). The individual components of the ACCI of the included patients are summarized in Supplemental Table 3, http://links.lww.com/AOSO/A429.

**TABLE 1.** Patient Characteristics and Laboratory Findings of the Nonagenarian and Centenarian Cohort

Variable	No. (%)	Number of Observed Cases	Number of Missing Cases, No. (%)
Female	1534 (49.7)	3085	0 (0.0)
Male	1551 (52.3)	3085	0 (0.0)
Age, mean (SD), y	92.3 (2.4)	3085	0 (0.0)
Smoking status		1069	2016 (65.3)
Nonsmoker	695 (22.5)		
Ex-smoker	366 (11.9)		
Current smoker	10 (0.3)		
Marital status		3034	51 (1.7)
Unmarried	217 (7.0)		
Married	1079 (35.0)		
Widowed	1738 (56.3)		
Residential status		2216	869 (28.2)
Independent living	444 (14.4)		
Living with family (partially dependent)	1140 (37.0)		
Community-based residential support (partially dependent)	25 (0.8)		
Aged-care facility	607 (19.7)		
Weight (SD), kg	66.3 (13.9)	1866	1219 (39.5)
Height (SD), cm	162.3 (9.4)	685	2400 (77.8)
Laboratory findings, mean (SD)			
Laboratory findings, mean (SD)	119.2 (19.5)	2503	582 (18.9)
Hemoglobin, g/L	8.3 (4.1)	2503	582 (18.9)
White blood cell count, ×10^9^/L	243.1 (91.7)	2499	586 (19.0)
Platelet count, ×10^9^/L	139.5 (3.7)	2502	583 (18.9)
Sodium, mEq/L	4.5 (0.5)	2491	594 (19.3)
Potassium, mEq/L	100.9 (4.4)	2501	584 (18.9)
Chloride, mEq/L	25.6 (3.2)	2502	583 (18.9)
Bicarbonate, mEq/L	9.7 (5.1)	2502	583 (18.9)
Urea, mmol/L	105.7 (48.0)	2455	630 (20.4)
Creatinine, µmol/L	55.2 (125.4)	2502	583 (18.9)
eGFR, mL/min/1.73m^2^	5.9 (1.1)	1371	1714 (55.6)
Hemoglobin A_1c_, %	254.1 (440.0)	951	2134 (69.2)
Ferritin, ng/mL	1.2 (0.4)	1692	1393 (45.2)
International normalized ratio	30.3 (8.0)	1383	1702 (55.2)

Data are presented as value or number (percent); eGFR using the CKD-EPI formula.

aPTT indicates activated partial thromboplastin time; eGFR, estimated glomerular filtration rate; Hemoglobin A_1c_, glycated hemoglobin.

Nearly all (96.5%) were American Society of Anesthesiologists (ASA) class 3. While most patients (80.4%) underwent minor surgery, 1 in 3 patients (32.3%) underwent emergency surgery. The most common procedure was plastic surgery (20.6%), followed by orthopedic surgery (15.5%), urological surgery (14.3%), and endoscopy (12.5%). The types of surgery, including surgical risk and patients’ anesthesia-related characteristics, are presented in Supplemental Table 4, http://links.lww.com/AOSO/A429.

### Follow-up and Complications

The median follow-up period was 301.5 days (48.3–808.8 days). In total, 998 patients (32.4%; 95% CI = 30.7%–34.0%) developed postoperative complications during their index hospital admission. A total of 407 patients (13.2%; 95% CI = 12.0%–14.4%) experienced 4 or more complications. Among the 998 patients who developed postoperative complications, 337 (33.8%) experienced either CVD grade III or IV complications (95% CI = 30.8%–36.7%). A total of 108 patients (10.8%) died (CVD grade V; 95% CI = 8.9%–12.7%), of which 3.5% (95% CI = 2.9%–4.2%) died during their index admission (see Supplemental Table 5, http://links.lww.com/AOSO/A429). During the entire observation period, the mortality rate was 23.5% (95% CI = 22.0%–25.0%). The numbers of postoperative complications are presented in Supplemental Table 6, http://links.lww.com/AOSO/A429.

### Validation of the ACCI as a Postoperative Complication Prediction Model

**TABLE 2.** Discrimination Ability of ACCI and GERIATRIC Models with Measured Calibrations in Appropriately Classifying Patients who May or May Not Develop Complications

		Any Complication During Admission		Severe Complications During Admission		In-hospital Mortality	
		ACCI	GERIATRIC MODEL	ACCI	GERIATRIC MODEL	ACCI	GERIATRIC MODEL
Whole dataset	Discriminability (AUROC)	0.672 (0.653–0.691)	0.859 (0.844–0.875)	0.71 (0.69–0.74)	0.845 (0.830–0.867)	0.74 (0.69–0.78)	0.844 (0.803–0.885)
	Low-risk surgery	0.71 (0.68–0.75)	0.839 (0.808–0.869)	-	-	-	-
	Intermediate-risk surgery	0.64 (0.61–0.67)	0.804 (0.776–0.831)	-	-	-	-
	High-risk surgery	0.53 (0.45–0.60)	0.576 (0.490–0.662)	-	-	-	-
Training dataset	Discriminability (AUROC)	-	0.857 (0.837–0.876)		0.847 (0.823–0.871)		0.880 (0.844–0.915)
	Brier score	-	0.147		0.108		0.032
	Spiegelhalter z score (*P*)	-	1.701 (0.089)		1.525 (0.127)		−0.190 (0.850)
Validation dataset	Discriminability (AUROC)	-	0.872 (0.837–0.908)		0.881 (0.837–0.926)		0.768 (0.612–0.925)
	Brier score	-	0.137		0.089		0.032
	Spiegelhalter z score (*P*)	-	−0.267 (0.790)		−0.516 (0.606)		0.810 (0.418)
Test dataset	Discriminability (AUROC)	-	0.857 (0.824–0.890)		0.833 (0.793–0.874)		0.780 (0.668–0.893)
	Brier score	-	0.147		0.112		0.033
	Spiegelhalter z score (*P*)	-	0.390 (0.697)		1.143 (0.253)		0.197 (0.844)

Values are presented with 95% confidence intervals.

The ACCI was poor in predicting the incidence of postoperative complications (AUROC, 0.67; 95% CI = 0.65–0.69) and fair in predicting severe complications during admission (AUROC, 0.71; 95% CI = 0.69–0.74) and in-hospital mortality (AUROC, 0.74; 95% CI = 0.69–0.78) (see Table 2).

### Selecting Input Parameters for GERIATRIC Modeling

The correlation analysis between all 58 preoperative variables and each outcome is presented in Supplemental Table 7, http://links.lww.com/AOSO/A429. The following variables were selected as input parameters for *GERIATRIC* modeling: sex, age, all ACCI components, hemoglobin concentration, white blood cell count, sodium and potassium levels, surgery severity, surgery scheduled type (elective vs. emergency), and preoperative ICU care. The incidence and severity of postoperative complications and postoperative mortality were similarly distributed across the training, validation, and performance-test datasets (Supplemental Table 8, http://links.lww.com/AOSO/A429).

### GERIATRIC Model Development with the Training Dataset

**FIGURE 2.**
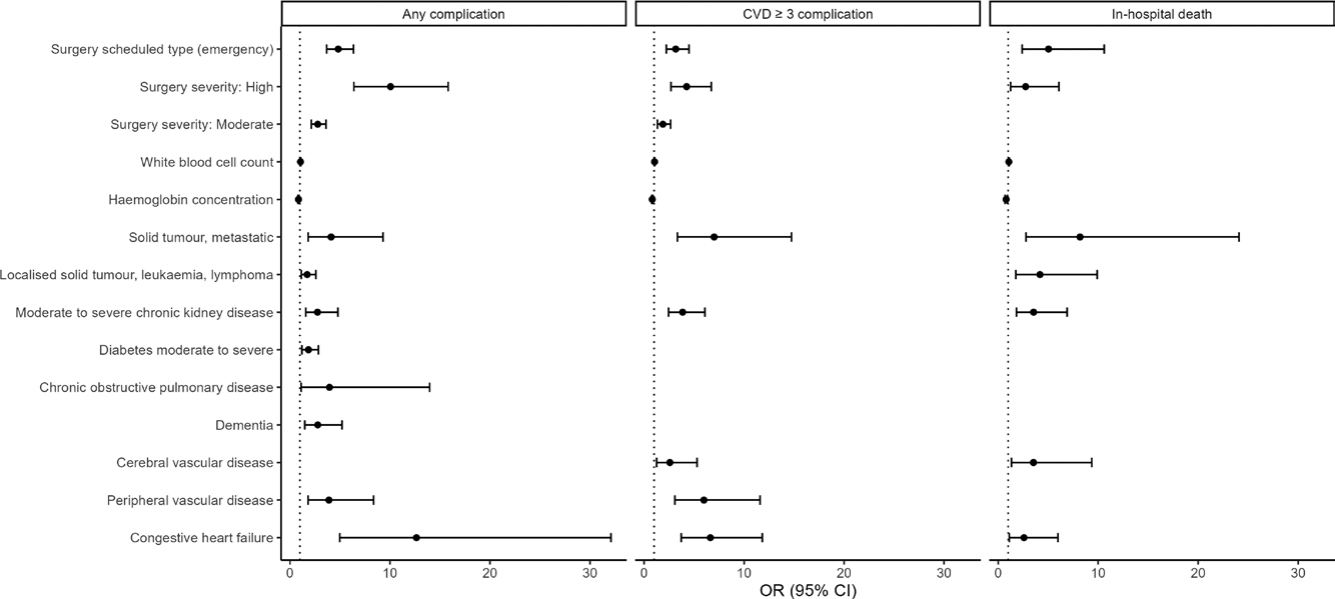
Odds ratios and 95% confidence intervals of the GERIATRIC model for appropriately classifying patients who may or may not develop complications. The dotted line indicates “odds ratio = 1.” Only statistically significant odds ratios are presented in the figure.

Some ACCI components, such as peptic ulcer disease, liver disease, and hemiplegia, were dropped from all 3 models. Others were selected or dropped during the stepwise process according to the outcome variables. Patients with congestive heart failure or those undergoing high-risk surgeries were at the highest risk of developing postoperative complications. Patients with a lower hemoglobin concentration had a higher incidence of postoperative complications. High-risk surgeries considerably increased the risk of postoperative complications. The odds ratios (ORs) of the GERIATRIC model for appropriately classifying patients who may or may not develop complications are presented in Figure 2. Detailed ORs and 95% CIs are presented in Supplemental Table 9, http://links.lww.com/AOSO/A429.

**FIGURE 3.**
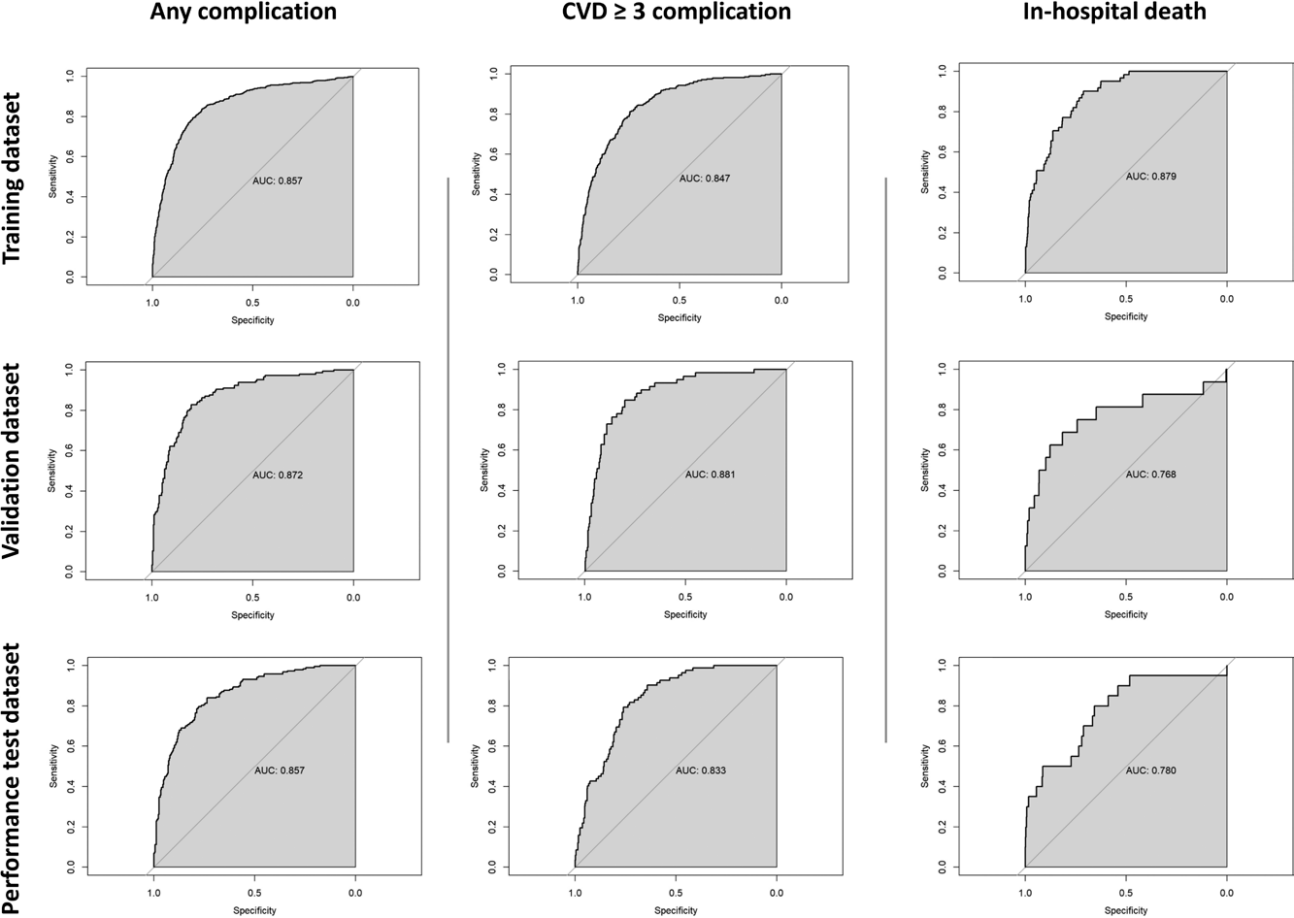
Receiver operating characteristic curves of GERIATRIC models on training, validation, and performance-test dataset.

The AUROC and calibrations measured on the training dataset are reported in Table 2 (Table 2 and Fig. 3). Based on the AUROC, the optimal thresholds for the discrimination were (1) any complication: 0.323 (specificity 0.799 and sensitivity 0.794), (2) severe complication: 0.144 (specificity 0.743 and sensitivity 0.812), and (3) in-hospital death: 0.030 (specificity 0.713 and sensitivity 0.902). With these thresholds, the AUROC was assessed to evaluate whether patients could be classified into those who may or may not be experiencing the event of interest, that is, discrimination ability to support clinical decision-making. The estimated AUROC shows fair to good discrimination ability for the development of any complication (AUROC, 0.796; 95% CI = 0.776–0.817), the development of severe complications (0.778; 95% CI = 0.752–0.804) and for in-hospital mortality (0.807; 95% CI = 0.768–0.847).

### GERIATRIC Model Validation

The performance of estimated GERIATRIC models was evaluated using the validation dataset. (Table 2 and Fig. 3). The models showed good discrimination ability for predicting the risk of postoperative complications and fair discrimination ability for classifying in-hospital mortality. The discrimination ability of the training dataset for predicting complications was similar to the discrimination ability on validation dataset (AUROC, 0.808; 95% CI = 0.768–0.848; specificity, 0.823; sensitivity, 0.793 for any complication; 0.812, 95% CI = 0.761–0.863; specificity, 0.776; sensitivity, 0.847 for severe complication; and 0.723; 95% CI = 0.603–0.842; specificity, 0.758; sensitivity, 0.688 for in-hospital mortality).

### GERIATRIC Model Performance Testing

The developed and validated models showed good discrimination ability for predicting any postoperative complication and severe postoperative complication and fair discrimination ability for predicting in-hospital mortality (Table 2 and Fig. 3). With the thresholds obtained on the training dataset, the AUROC was assessed to evaluate the discrimination ability to predict the risk of developing postoperative complications. The estimated AUROC showed fair discrimination ability for the development of any complication (0.777; 95% CI = 0.740–0.815; specificity, 0.736; sensitivity, 0.840), severe complications (0.763; 95% CI = 0.716–0.811; specificity, 0.709; sensitivity 0.817), and in-hospital death (0.705; 95% CI = 0.605–0.804; specificity, 0.659; sensitivity, 0.750).

### Calibrations

The measured calibrations were adequate for all models; however, a visual check of calibration plots showed that the model performance was only acceptable for predicting the probability of patients experiencing any complication, that is, poor calibration ability to predict severe complications or mortality. Therefore, recalibration methods were applied to the latter 2 models, and the performance of the original and recalibrated models was tested on the testing dataset. The final models were good at discriminating patients into a higher versus lower likelihood of experiencing a severe complication or dying but were unable to correctly predict the individual patient probability of experiencing such events, that is, poor calibration ability.

### Clinical Applications

Examples of the clinical applications of the web-based GERIATRIC risk calculator are presented in Supplemental Figure 1, http://links.lww.com/AOSO/A429. The figure outlines how the calculator provides an estimated risk of postoperative complications in both nonagenarians and centenarians. By choosing the input parameters of the patient, including surgery severity, the age-adjusted Charlson Comorbidity Index and GERIATRIC postoperative complication risk are reported. The GERIATRIC risk calculator is freely available at https://entopic.shinyapps.io/webcalculator/.

## DISCUSSION

### Key Findings

In this single-center, retrospective cohort study of more than three thousand nonagenarian and centenarian patients undergoing surgery, ACCI was a poor predictor of the development of postoperative complications and mortality. In contrast, the GERIATRIC risk tool demonstrated excellent discriminative ability to effectively classify patients into those who may or may not develop postoperative complications or a severe complication during the hospital admission and outperformed the ACCI in classifying patients who may or may not die during their index hospital stay. Patients with congestive heart failure and those undergoing high-risk surgery were at the highest risk of developing postoperative complications. These enhancements, supported by both statistical validation and clinical relevance, offer a more robust tool for risk stratification compared to the ACCI, particularly in high-risk geriatric populations.

### Study Implications

The GERIATRIC risk tool provides an accurate perioperative risk assessment for nonagenarian and centenarian patients. The risk tool is not recommended for predicting individual probabilities but rather for the classification of nonagenarian patients into those that are likely or unlikely to experience postoperative complications including severe complications and in-hospital mortality. The ACCI includes only 19 comorbidities, which may not adequately account for the complexities faced by nonagenarians. In contrast, the GERIATRIC tool integrates additional factors such as electrolyte imbalances, hemoglobin concentration, type of surgery (elective vs. emergency), surgery severity, and preoperative ICU care, which are critical for the classification of patients into those that are likely or unlikely to experience postoperative complications. By integrating these additional variables, the GERIATRIC tool provides a more comprehensive and precise framework than the ACCI for surgical risk and for classifying postoperative outcomes in elderly patients, particularly those aged 90 and above. Therefore, the tool can be utilized to better inform clinical decision-making and suitability for operative management. Furthermore, the GERIATRIC risk tool may help facilitate transparent higher-quality discussions, shared decision-making, and patient-centered care in the context of surgery.

### Relationship to the Literature

Currently, there are no validated perioperative risk assessment tools specifically designed for nonagenarians and centenarians. Moreover, the existing risk scores appear to perform poorly or inconsistently in this vulnerable patient cohort. Larson et al^21^ assessed the CCI in a population aged >95 years across a range of surgeries and found no association between the CCI and major complications or mortality. Subsequent studies have not demonstrated an association between the CCI and important postoperative outcomes.^22^ Even the Physiological and Operative Severity Score for the Enumeration of Mortality and Morbidity^23^ has been assessed in nonagenarians with mixed results.^23^

ASA performance status has also previously been observed to correlate with increased postoperative morbidity and mortality in this population.^22,24–29^ However, in the present study, 96.5% of anesthesiologists graded nonagenarian and centenarian patients as ASA class 3, thus limiting the utility of this metric in this setting. This proportion of categorization of patients as ASA class 3 is higher than elsewhere in the literature,^22^ which may reflect an inherent bias in subjective assessments of these patients due to age and perceived frailty. This suggests that the GERIATRIC risk tool is more objective than the ASA performance status in assisting perioperative clinicians with decision-making and outcome prediction.

### Strengths and Limitations

Our study has several strengths. The cohort included over 3000 such patients undergoing a wide range of surgical procedures at a large, high-volume, tertiary referral center.

This study had several limitations. The retrospective study design limited the quality of data collected, and consequently, model accuracy. While the GERIATRIC risk tool was effective in classifying patients into those who may or may not experience complications and mortality during their index admission (discrimination ability), it was not able to predict individual patient probabilities of experiencing such events occurring (calibration ability). There was only a relatively small proportion of patients undergoing surgery in each subspecialty; therefore, individual surgery-specific risk scores were difficult to validate. Similarly, there were only 31 patients (1.0% of all patients) aged ≥100 years.

Due to the retrospective design, we were not able to evaluate functional status or outcomes using tools such as the World Health Organization Disability Assessment Schedule (WHODAS 2.0) or the EuroQol (EQ5D).^30,31^ In addition, we could not objectively assess frailty. These scores can provide additional evidence on the relative effectiveness of healthcare interventions and are arguably more pertinent outcomes than the objective measures of morbidity and mortality. The Edmonton Frailty Scale has been identified as being a good demonstrating tool for predicting postoperative complications.^32^ From our data collected, only 1 in 5 patients were living independently, with the remaining patients being partially or fully dependent on others, reflecting a moderate to severely frail patient cohort. Finally, as this is a single-center study, the GERIATRIC risk models require further validation in other hospitals and countries.

## CONCLUSION

Nonagenarians and centenarians are high-risk surgical cohorts. The ACCI is a poor predictor of postoperative morbidity and mortality in this vulnerable patient population. The GERIATRIC risk models are modified risk assessment scores that incorporate parameters of the ACCI, in addition to other clinical and surgical factors. Compared with the ACCI, the GERIATRIC risk models more accurately classify patients into those who may or may not experience complications and mortality during their index admission. As such, they can be used to assist perioperative clinicians with shared decision-making and short-term prognostication.

## Supplementary Material

## References

[1] Christensen K Doblhammer G Rau R. Ageing populations: the challenges ahead. Lancet. 2009;374:1196–1208. 19801098 10.1016/S0140-6736(09)61460-4 PMC2810516

[2] Wilson T Temple J. The rapid growth of Australia’s advanced age population. J Popul Res. 2020;37:377–389.

[3] Kim TI Brahmandam A Skrip L. Surgery for the very old: are nonagenarians different? Am Surg. 2020;86:56–64. 32077417

[4] Roque-Castellano C Fariña-Castro R Nogués-Ramia EM. Colorectal cancer surgery in selected nonagenarians is relatively safe and it is associated with a good long-term survival: an observational study. World J Surg Oncol. 2020;18:120. 32493351 10.1186/s12957-020-01895-8 PMC7271489

[5] Kitridis D Tsikopoulos K Givissis P. Mortality and complication rates in nonagenarians and octogenarians undergoing total hip and knee arthroplasty: a systematic review and meta-analysis. Eur Geriatr Med. 2022;13:725–733. 35072938 10.1007/s41999-022-00610-y

[6] Pelavski AD Lacasta A Rochera MI. Observational study of nonogenarians undergoing emergency, non-trauma surgery. Br J Anaesth. 2011;106:189–193. 21112879 10.1093/bja/aeq335

[7] Ogawa T Schermann H Kobayashi H. Age and clinical outcomes after hip fracture surgery: do octogenarian, nonagenarian and centenarian classifications matter? Age Ageing. 2021;50:1952–1960. 34228781 10.1093/ageing/afab137

[8] Charlson ME Pompei P Ales KL. A new method of classifying prognostic comorbidity in longitudinal studies: development and validation. J Chronic Dis. 1987;40:373–383. 3558716 10.1016/0021-9681(87)90171-8

[9] Charlson M Szatrowski TP Peterson J. Validation of a combined comorbidity index. J Clin Epidemiol. 1994;47:1245–1251. 7722560 10.1016/0895-4356(94)90129-5

[10] Moons KG Altman DG Reitsma JB. Transparent Reporting of a multivariable prediction model for Individual Prognosis or Diagnosis (TRIPOD): explanation and elaboration. Ann Intern Med. 2015;162:W1–73. 25560730 10.7326/M14-0698

[11] Pasternak LR. Preanesthesia evaluation of the surgical patient. ASA Refresher Courses Anesthesiol. 1996;24:205–219.

[12] Bravo G Dubois MF Hébert R. A prospective evaluation of the Charlson Comorbidity Index for use in long-term care patients. J Am Geriatr Soc. 2002;50:740–745. 11982678 10.1046/j.1532-5415.2002.50172.x

[13] Jammer I Wickboldt N Sander M; European Society of Anaesthesiology (ESA) and the European Society of Intensive Care Medicine (ESICM). Standards for definitions and use of outcome measures for clinical effectiveness research in perioperative medicine: European Perioperative Clinical Outcome (EPCO) definitions: a statement from the ESA-ESICM joint taskforce on perioperative outcome measures. Eur J Anaesthesiol. 2015;32:88–105. 25058504 10.1097/EJA.0000000000000118

[14] Dindo D Demartines N Clavien PA. Classification of surgical complications: a new proposal with evaluation in a cohort of 6336 patients and results of a survey. Ann Surg. 2004;240:205–213. 15273542 10.1097/01.sla.0000133083.54934.ae PMC1360123

[15] Clavien PA Barkun J de Oliveira ML. The Clavien-Dindo classification of surgical complications: five-year experience. Ann Surg. 2009;250:187–196. 19638912 10.1097/SLA.0b013e3181b13ca2

[16] Kwak SK Kim JH. Statistical data preparation: management of missing values and outliers. Korean J Anesthesiol. 2017;70:407–411. 28794835 10.4097/kjae.2017.70.4.407 PMC5548942

[17] Clark TG Altman DG. Developing a prognostic model in the presence of missing data: an ovarian cancer case study. J Clin Epidemiol. 2003;56:28–37. 12589867 10.1016/s0895-4356(02)00539-5

[18] Robin X Turck N Hainard A. pROC: an open-source package for R and S+ to analyze and compare ROC curves. BMC Bioinf. 2011;12:77. 10.1186/1471-2105-12-77 PMC3068975 21414208

[19] Harrell FE Jr. RMS: regression modeling strategies. R package version 6.3-0. 2022. Available at https://CRAN.R-project.org/package=rms

[20] Leeuw JD Hornik K Mair P. Isotone optimization inR: Pool-Adjacent-Violators Algorithm (PAVA) and active set methods. J Stat Softw. 2009;32:1–24.

[21] Larson KJ Hamlin RJ Sprung J. Associations between Charlson Comorbidity Index and surgical risk severity and the surgical outcomes in advanced-age patients. Am Surg. 2014;80:555–560. 24887792

[22] Fariña-Castro R Roque-Castellano C Marchena-Gómez J. Five-year survival after surgery in nonagenarian patients. Geriatr Gerontol Int. 2017;17:2389–2395. 28675571 10.1111/ggi.13081

[23] Copeland GP Jones D Walters M. POSSUM: a scoring system for surgical audit. Br J Surg. 1991;78:355–360. 2021856 10.1002/bjs.1800780327

[24] Racz J Dubois L Katchky A. Elective and emergency abdominal surgery in patients 90 years of age or older. Can J Surg. 2012;55:322–328. 22992421 10.1503/cjs.007611 PMC3468645

[25] Tecos ME Kern BS Foje NA. Perioperative considerations in nonagenarians. Surg Open Sci. 2020;2:45–49. 33073225 10.1016/j.sopen.2020.03.004 PMC7545003

[26] Hayes AJ Davda A El-Hadi M. Short and medium-term outcomes for general surgery in nonagenarian patients in a district general hospital. Ann R Coll Surg Engl. 2016;98:401–404. 27138856 10.1308/rcsann.2016.0142 PMC5209969

[27] Weinberg L Ou Yang B Cosic L. Factors influencing early and long-term survival following hip fracture among nonagenarians. J Orthop Surg Res. 2021;16:653. 34717695 10.1186/s13018-021-02807-6 PMC8557574

[28] Liu Y Peng M Lin L. Relationship between American Society of Anesthesiologists (ASA) grade and 1-year mortality in nonagenarians undergoing hip fracture surgery. Osteoporos Int. 2015;26:1029–1033. 25300530 10.1007/s00198-014-2931-y

[29] Bovonratwet P Yang BW Wang Z. Operative fixation of hip fractures in nonagenarians: is it safe? J Arthroplasty. 2020;35:3180–3187. 32624381 10.1016/j.arth.2020.06.005

[30] Üstün TB Kostanjsek N Chatterji S Rehm J. Measuring health and disability: manual for WHO disability assessment schedule WHODAS 2.0. Geneva: World Health Organization; 2010.

[31] EuroQol Group. EuroQol--a new facility for the measurement of health-related quality of life. Health Policy. 1990;16:199–208. 10109801 10.1016/0168-8510(90)90421-9

[32] He Y Li LW Hao Y. Assessment of predictive validity and feasibility of Edmonton Frail Scale in identifying postoperative complications among elderly patients: a prospective observational study. Sci Rep. 2020;10:14682. 32895396 10.1038/s41598-020-71140-5 PMC7477578

