# Supplementary material for: The Perioperative NonaGEnaRIan And cenTenarian suRgICal (GERIATRIC) Risk Stratification Tool
Source: Ann Surg Open. 2024 Nov 18;5(4):e524. doi: 10.1097/AS9.0000000000000524 (PMC11661723; doi:10.1097/AS9.0000000000000524)

eTable 1. Age-adjusted Charlson Comorbidity Index (ACCI)

Score	Comorbid condition
1	Myocardial infarction
	Congestive heart failure
	Cerebral vascular disease
	Peripheral vascular disease
	Dementia
	Chronic obstructive pulmonary disease
	Connective tissue disease
	Peptic ulcer disease
	Mild liver disease
	Diabetes without organ damage
2	Hemiplegia
	Moderate to severe renal disease
	Diabetes with end organ damage
	Solid tumor
	Leukemia
	Lymphoma
3	Moderate to severe liver disease
6	Metastatic solid tumor
	Acquired immunodeficiency syndrome
	**Age, year**
1 point	41–50
2 points	51–60
3 points	61–70
4 points	≥71

eTable 2. The Johns Hopkins Surgical Classification.

<table>
<tr><th>Johns Hopkins Surgical Class</th><th>Modified Hopkins Surgical Classification</th><th>Definition</th><th>Procedures included</th><th>Procedures excluded</th></tr>
<tr><td>I</td><td>Mild-risk group</td><td>Minimal risk to patient
Minimally invasive procedures, often done in an ‘office setting,’ with the operating room used principally for anesthesia and monitoring</td><td>Removal of minor skin lesions
Breast biopsy
Flexible bronchoscopy
Vasectomy
Cystoscopy</td><td>Open exposure of internal body organs
Repair of vascular or neurological structures
Placement of prosthetic device
Postoperative monitored care setting in ICU or HDU
Entry into abdomen, thorax, neck, cranium or extremities</td></tr>
<tr><td>II</td><td rowspan="2">Intermediate-risk group</td><td>Minimal to mild invasive procedures
Blood loss <500 mL, mild risk to the patient independent of anesthesia</td><td>Diagnostic laparoscopy
Inguinal hernia repair
Elective laparoscopic cholecystectomy
Septoplasty/rhinoplasty
Extensive superficial procedures</td><td>Open exposure of internal body organs
Repair of vascular or neurological structures
Placement of prosthetic devices
Planned postoperative monitored care setting in ICU or HDU
Open exposure of abdomen, thorax, neck, cranium or extremities</td></tr>
<tr><td>III</td><td>Moderate to significant invasive procedures
Potential blood loss of 500–1500 mL
Moderate risk to patients independent of anesthesia day unit</td><td>Total knee or hip arthroplasty
Major laparoscopic procedures
Thyroidectomy
Laminectomy</td><td>Open thoracic procedures
Open intracranial procedures
Planned postoperative monitored care setting in ICU or HDU</td></tr>
</table>

**IV**	High-risk group	Highly invasive procedures<br>Major risk to patient independent of anesthesia<br>Potential blood loss >1500 mL	Major reconstructions of the digestive tract<br>Major orthopedic or spinal reconstruction<br>Major genitourinary reconstruction<br>Major vascular repair without ICU stay<br>Minimally invasive thoracic procedures	All of above
**V**		Highly invasive procedures<br>Blood loss >1500 mL<br>Critical risk to patient independent of anesthesia<br>Usual postoperative ICU stay with invasive monitoring	Open cardiothoracic procedures<br>Major vascular reconstructions<br>Major upper gastrointestinal reconstructions e.g., esophagectomy<br>Open thoracic procedures	

Abbreviations: HDU, High Dependency Unit; ICU, Intensive Care Unit

eTable 3. The Age-adjusted Charlson Comorbidity Index components (N=3085).

Variable	No. (%)
**Age**	
Myocardial infarction	15 (0.5)
Congestive heart failure	102 (3.3)
Peripheral vascular disease	66 (2.1)
CVA or TIA	59 (1.9)
Dementia	123 (4.0)
Chronic obstructive pulmonary disease	44 (1.4)
Connective tissue disease	2 (0.1)
Peptic ulcer disease	41 (1.3)
**Liver disease**	
Mild	11 (0.4)
Moderate to severe	5 (0.2)
**Diabetes**	
Uncomplicated	215 (7.0)
End organ damage	265 (8.6)
Hemiplegia	27 (0.9)
Moderate to severe chronic kidney disease	180 (5.8)
Localized solid tumour, leukemia, lymphoma	272 (8.8)
Solid tumour, metastatic	61 (2.0)
Acquired immunodeficient disorder syndrome	0 (0.0)

Abbreviations: CVA, Cerebral Vascular Accident; TIA, Transient Ischemic Attack

eTable 4. Surgery- and anesthesia-related parameters characteristics.

		Surgical risk		
	**No. (%)**	**Low**	**Intermediate**	**High**
**Type of surgery/procedures**				
Plastic surgery	636 (20.6)	419	216	1
Orthopedics	478 (15.5)	2	307	169
Urology	445 (14.4)	325	120	0
Endoscopy	385 (12.5)	359	26	0
General Surgery	331 (10.7)	53	253	25
Ophthalmology	247 (8.0)	239	7	1
Flexible cystoscopy	148 (4.8)	148	0	0
Vascular	88 (2.9)	5	67	16
Thoracics/Respiratory	51 (1.7)	26	24	1
Other e.g., ECT, lumbar puncture	49 (1.6)	45	4	0
Radiology	48 (1.6)	8	8	32
Ear Nose and Throat	43 (1.4)	10	32	1
Cardiology	38 (1.2)	7	31	0
Cardiac	27 (0.9)	0	0	27
Maxillofacial	25 (0.8)	16	6	3
Neurosurgery	23 (0.7)	0	9	14
Breast	22 (0.7)	11	11	0
Gynecology	1 (0.0)	1	0	0
**Total / Missing No. (%)**	**3085 / 0 (0.0)**	**1674 (54.3)**	**1121 (36.3)**	**290 (9.4)**
**Severity of performed surgery**				
Class I	1674 (54.3)			
Class II	805 (26.1)			
Class III	316 (10.2)			
Class IV	256 (8.3)			
Class V	34 (1.1)			
**Total / Missing No. (%)**	**3085 / 0 (0.0)**			
**Surgery scheduled urgency**[a]				
Elective	2090 (67.7)			
Emergency	995 (32.3)			
**Total / Missing No. (%)**	**3085 / 0 (0.0)**			
**ASA class**				
1	0 (0.0)			
2	22 (0.7)			
3	2976 (96.5)			
4	86 (2.8)			
5	1 (0.0)			
**Total / Missing No. (%)**	**3085 / 0 (0.0)**			
**Anesthetic type**				
General	1037 (33.6)			

Sedation	377 (12.2)			
Local & sedation	232 (7.5)			
Local	181 (5.9)			
Regional	66 (2.1)			
Spinal	17 (0.6)			
Spinal & sedation	13 (0.4)			
Regional & sedation	9 (0.3)			
None	5 (0.2)			
Topical	1 (0.0)			
**Total / Missing No. (%)**	**1938 / 1147 (37.2)**			

Abbreviations: ASA, American Society of Anesthesiology; ECT, Electroconvulsive Therapy. [a]The severity of surgery was evaluated using the Johns Hopkins Surgical Classification System.

eTable 5. Postoperative complications and mortality.

**Outcome**	**No. (%)**	**Number**	**Number of missing cases, No. (%)**
Postoperative observed period, median (Q1-Q3) [Min:Max], d	301.5 (48.3-808.8) [0:3482]	3080	5 (0.2)
Patient admitted to high dependency unit	40 (1.3)	3085	0 (0.0)
Patients admitted to ICU	62 (2)	3085	0 (0.0)
Total number of ICU hours, median (Q1-Q3) [Min:Max], h	41.3 (23.7-68.5) [2.8:252.2]	62	0 (0.0)
Patients requiring mechanical or non-invasive ventilation	33 (1.1)	3085	0 (0.0)
Total number of mechanical or non-invasive ventilation hours, median (Q1-Q3) [Min:Max], h	19 (13-36.5) [4:133]	33	0 (0.0)
**Mortality and complications**			
Death during the hospital stay	109 (3.5)	3085	0 (0.0)
Death within 90 days after surgery	203 (6.6)	3085	0 (0.0)
Death during observation period	724 (23.5)	3085	0 (0.0)
**Number of complications**		3085	0 (0.0)
Number of patients with any postoperative complications	998 (32.4)		
Patients with 1 complication	246 (8.0)		
Patients with 2 complications	217 (7.0)		
Patients with 3 complications	127 (4.1)		
Patients with 4 or more complications	408 (13.2)		
**Worst complication grade (Clavien-Dindo Classification)**		3085	0 (0.0)
1	129 (4.2)		
2	424 (13.7)		
3	90 (2.9)		
4<br>5	247 (8.0)<br>108 (3.5)		
**Readmissions**		3103	0 (0)
Planned	53 (1.7)		
Unplanned	39 (1.3)		

Abbreviations: ICU, Intensive Care Unit.

eTable 6. Reported frequencies of postoperative complications.

Detailed classifications	Clavien-Dindo Classification					Total No. (%)
	1	2	3	4	5	
**Cardiac**						
Arrhythmia	0	41	3	23	7	74 (2.16)
Hypertension	0	76	13	60	25	174 (5.08)
Bradycardia	8	10	3	14	1	36 (1.05)
Tachycardia	0	25	8	15	8	56 (1.64)
Syncope	0	11	3	5	2	21 (0.61)
Chest Pain/EKG changes	11	16	3	10	6	46 (1.34)
Hypotension	0	119	26	71	49	265 (7.74)
Heart Failure/Infarct	0	0	0	16	26	42 (1.23)
Other	1	6	3	27	6	43 (1.26)
Vascular	0	11	2	6	3	22 (0.64)
**Hematological**						
Thrombosis/Embolism/Clotting	0	0	0	5	0	5 (0.15)
Other	1	10	26	6	7	50 (1.46)
White Blood Cell Count Derangement	0	3	2	4	0	9 (0.26)
Anemia	0	83	37	58	25	203 (5.93)
**Pulmonary**						
Atelectasis/Failure/Pneumothorax	0	0	0	53	13	66 (1.93)
Pulmonary edema/Pleural effusion/Empyema	0	16	2	10	3	31 (0.91)
Pulmonary Embolism	0	0	0	9	2	11 (0.32)
Pneumonia/Aspiration	0	5	1	6	4	16 (0.47)
Tachypnoea/Dyspnea	2	16	2	7	5	32 (0.94)
Severe cough/Wheeze	1	7	1	0	0	9 (0.26)
Other	0	0	0	1	2	3 (0.09)
**Gastrointestinal**						
High Stomal Output/Dysfunction	0	0	1	1	0	2 (0.06)
Abdominal Pain	3	4	0	2	2	11 (0.32)
Dysphagia/Heartburn	1	19	2	17	11	50 (1.46)
Nausea/Vomiting/Appetite	8	7	1	5	4	25 (0.73)
GI Hemorrhage	0	1	22	8	9	39 (1.14)
Diarrhea	0	1	0	0	0	1 (0.03)
Constipation	0	82	9	48	12	151 (4.41)
Anastomotic Leak	0	0	0	0	0	0 (0.00)
Intra-abdominal Infection	0	15	5	9	3	32 (0.94)
Surgical Site Infection/Disruption	0	0	8	6	3	17 (0.50)
Ischemic bowel	0	0	0	1	0	1 (0.03)
Ileus/Bowel obstruction	0	10	2	3	3	18 (0.53)
Delayed Gastric Emptying	0	1	0	0	0	1 (0.03)
Nasogastric tube malposition	0	0	0	0	0	0 (0.00)
Pancreatitis	0	1	2	0	0	3 (0.09)
Abdominal Distension	0	0	0	0	0	0 (0.00)
Liver Derangement	1	7	2	9	2	21 (0.61)
Ascites	0	0	1	0	0	1 (0.03)
Gallbladder	0	0	0	2	0	2 (0.06)
Fecal Incontinence	0	2	0	1	0	3 (0.09)
Other	1	12	5	9	4	31 (0.91)
Biliary obstruction	0	0	1	1	0	2 (0.06)

**Infection**						
Sepsis	0	0	0	14	10	24 (0.70)
Fever	7	18	4	8	6	43 (1.26)
Line infection/Block	0	0	0	0	0	0 (0.00)
Wound/Infection at Other Site	1	32	10	29	14	86 (2.51)
**Metabolic**						
Electrolyte Disturbance	15	79	15	60	23	192 (5.61)
Diabetic ketoacidosis	0	0	0	0	0	0 (0.00)
Acidosis/Alkalosis	0	4	2	9	3	16 (0.47)
SIRS	0	1	0	0	0	1 (0.03)
Nutritional Deficiency	0	12	3	17	7	39 (1.14)
**Neurological**						
Stroke/TIA/Encephalopathy	0	0	0	5	3	8 (0.23)
Delirium/Hallucinations	33	87	14	82	43	262 (7.66)
Headache/Migraine	1	1	0	0	0	2 (0.06)
Other	3	10	6	16	5	40 (1.17)
**Renal**						
Acute kidney injury	0	0	0	134	31	165 (4.82)
Urinary Retention	12	27	6	31	8	84 (2.45)
Urinary Incontinence	3	3	0	0	1	7 (0.20)
Hematuria	0	11	5	6	6	28 (0.82)
Urinary tract infection	0	24	4	27	10	65 (1.9)
Anuria/Oliguria	0	0	0	8	0	8 (0.23)
Polyuria	0	1	0	1	0	2 (0.06)
Fluid Overload	11	34	6	39	15	105 (3.07)
Other including urinary catheter trauma related	2	16	5	23	7	53 (1.55)
**Dermatological**						
Pressure Sore	6	16	6	12	4	44 (1.29)
Rash	0	12	1	6	3	22 (0.64)
Allergy	2	2	0	0	0	4 (0.12)
Skin: Other/Nails	4	9	1	12	5	31 (0.91)
Skin: Cellulitis	0	0	3	5	4	12 (0.35)
**Endocrine**						
Blood sugar complication	0	5	0	1	5	11 (0.32)
SIADH	0	1	0	0	0	1 (0.03)
Hypo/Hyperthyroid	0	7	0	1	0	8 (0.23)
**Other**						
Surgical: Tissue Failure/Rejection	0	0	1	3	0	4 (0.12)
Surgical: Hemorrhage/Hematoma	0	1	0	0	1	2 (0.06)
Psychiatric: Mood disturbance	0	9	0	1	0	10 (0.29)
Other Surgical	2	9	7	14	1	33 (0.96)
Male Genital: Scrotal Edema/Orchitis/Anorectal-Related	0	4	2	3	1	10 (0.29)
Gynecological: Vaginal Bleed/Swelling/Infection	0	3	2	1	1	7 (0.20)
Anesthesia: Uncontrolled Pain/Analgesia/Anesthesia adverse effects	2	5	2	21	4	34 (0.99)
Mechanical Fall/Contusion/Superficial Injury	10	12	12	20	6	60 (1.75)
Bone/Joint-Related	0	4	4	1	1	10 (0.29)
Dizziness/Restlessness/Agitation	0	12	0	4	3	19 (0.56)
Limb Ischemia/Necrosis	0	0	9	6	3	18 (0.53)
Somnolence	0	8	0	7	7	22 (0.64)
Deconditioning/Mobility Impairment	3	9	1	7	5	25 (0.73)

Fracture/Dislocation	0	1	13	5	3	22 (0.64)
Severe Pain	0	6	4	13	3	26 (0.76)
Adverse drug effect	1	19	2	16	10	48 (1.40)
Palliation/Death	0	0	0	0	0	0 (0.00)
Miscellaneous	4	14	8	10	10	46 (1.34)
Other: ENT	2	6	0	4	8	20 (0.58)
Ophthalmologic	4	3	5	4	2	18 (0.53)
**Total**	**166**	**1154**	**359**	**1214**	**529**	**3422 (100)**

Abbreviations: EKG, Electrocardiogram; SIRS, Systemic Inflammatory Response Syndrome; TIA, Transient Ischemic Attack; SAIDH, Syndrome of Inappropriate Antidiuretic Hormone secretion; ENT, Ear Nose & Throat. The same patient may have developed multiple complications, therefore the frequencies described in this table are duplicated, and the total count is higher than the number of patients (N=3085).

eTable 7. Correlation analysis between preoperative parameters and the presence of postoperative complications, severity of complications and in-hospital mortality.

Preoperative parameters	Presence of complications		CVD≥3 complications		In-hospital mortality	
	**ρ**	**P value**	**ρ**	**P value**	**ρ**	**P value**
Sex	-0.14	<0.001	-0.09	<0.001	-0.03	0.056
Age	0.07	<0.001	0.04	0.017	0.06	0.001
ASA physical classification	0.14	<0.001	0.12	<0.001	0.11	<0.001
Myocardial infarction	0.04	0.022	0.05	0.005	0.04	0.039
Congestive heart failure	0.24	<0.001	0.24	<0.001	0.13	<0.001
Peripheral vascular disease	0.15	<0.001	0.17	<0.001	0.02	0.261
Cerebral vascular disease	0.13	<0.001	0.14	<0.001	0.13	<0.001
Dementia	0.20	<0.001	0.13	<0.001	0.03	0.069
Chronic obstructive pulmonary disease	0.13	<0.001	0.08	<0.001	0.07	<0.001
Connective tissue disease	0.01	0.594	0.03	0.152	0.00	0.787
Peptic ulcer disease	0.05	0.003	0.08	<0.001	0.04	0.030
**Liver disease**						
Mild	-0.01	0.718	-0.02	0.173	-0.01	0.525
Moderate to severe	0.01	0.715	-0.02	0.358	-0.01	0.669
**Diabetes**						
Uncomplicated	0.01	0.502	-0.03	0.158	0.01	0.591
End organ damage	0.15	<0.001	0.13	<0.001	0.03	0.107
Hemiplegia	0.10	<0.001	0.10	<0.001	0.10	<0.001
Moderate to severe chronic kidney disease	0.24	<0.001	0.30	<0.001	0.18	<0.001
Localized solid tumor, leukemia, lymphoma	-0.03	0.104	-0.04	0.017	0.00	0.893
Solid tumor, metastatic	0.10	<0.001	0.11	<0.001	0.11	<0.001
Acquired immunodeficiency disorder	-	-	-	-	-	-

syndrome						
Hemoglobin concentration derangement	-0.22	<0.001	-0.19	<0.001	-0.11	<0.001
White blood cell count derangement	0.19	<0.001	0.14	<0.001	0.11	<0.001
Sodium derangement	-0.09	<0.001	-0.04	0.067	0.00	0.954
Potassium derangement	-0.10	<0.001	-0.06	0.002	-0.04	0.068
Surgery severity	0.39	<0.001	0.25	<0.001	0.10	<0.001
Surgery scheduled type (emergency)	0.52	<0.001	0.37	<0.001	0.21	<0.001
Preoperative ICU admission	0.09	<0.001	0.11	<0.001	0.15	<0.001

Abbreviations: ρ, Spearman correlation coefficient; CVD, Clavien-Dindo surgical complication grade; POP, Postoperative; ACCI, Age-adjusted Charlson's Comorbidity Index; ICU, Intensive Care Unit.

eTable 8. Random data extraction results. Data are presented as number (percentile in each dataset).

Target outcomes	Whole data (N=3085)	Training data (N=1968)	Validation data (N=492)	Performance-test data (N=625)
Postoperative complications	998 (32.4%)	647 (32.9%)	148 (30.1%)	203 (32.5%)
CVD≥3 complications	445 (14.4%)	296 (15%)	61 (12.4%)	88 (14.1%)
In-hospital death	109 (3.5%)	70 (3.6%)	17 (3.5%)	22 (3.5%)

Abbreviations: CVD: Clavien-Dindo surgical complication grade

eTable 9. Odds ratios (OR) and corresponding 95% confidence intervals of logistic regression models for any postoperative complication during admission, CVD ≥ 3 complications during admission, and in-hospital mortality.

**Parameter**		**Any complication**		**CVD ≥ 3 complication**		**In-hospital death**	
		**OR (95% CI)**	**P value**	**OR (95% CI)**	**P value**	**OR (95% CI)**	**P value**
Intercept		0.747 (0.157 – 3.551)	0.715	0.004 (0.000 – 1.346)	0.064	0.000 (0.000 – 0.114)	0.018*
Myocardial infarction		(–)		(–)		0.000 (0.000 – Infinite)	0.985
Congestive heart failure		12.645 (4.981 – 32.101)	<0.001*	6.627 (3.715 – 11.821)	<0.001*	2.584 (1.115 – 5.990)	0.027*
Peripheral vascular disease		3.888 (1.809 – 8.354)	0.001*	5.985 (3.088 – 11.598)	<0.001*	(–)	
Cerebral vascular disease		(–)		2.579 (1.258 – 5.288)	0.010*	3.532 (1.333 – 9.360)	0.011*
Dementia		2.775 (1.480 – 5.202)	0.001*	1.692 (0.994 – 2.881)	0.053	0.349 (0.098 – 1.240)	0.104
Chronic obstructive pulmonary disease		3.945 (1.115 – 13.964)	0.033*	(–)		(–)	
Diabetes	Mild	1.52 (0.971 – 2.381)	0.067	(–)		(–)	
	Moderate to severe	1.844 (1.193 – 2.851)	0.006*	(–)		(–)	
Moderate to severe chronic kidney disease		2.747 (1.577 – 4.786)	<0.001*	3.854 (2.440 – 6.088)	<0.001*	3.543 (1.821 – 6.896)	<0.001*
Localized solid tumor, leukemia, lymphoma		1.715 (1.136 – 2.587)	0.010*	1.730 (0.999 – 2.995)	0.050	4.182 (1.767 – 9.899)	0.001*
Solid tumor, metastatic		4.11 (1.816 – 9.303)	0.001*	7.006 (3.331 – 14.734)	<0.001*	8.187 (2.783 – 24.086)	<0.001*
Hemoglobin concentration		0.848 (0.793 – 0.907)	<0.001*	0.820 (0.756 – 0.890)	<0.001*	0.798 (0.682 – 0.933)	0.006*
White blood cell count		1.049 (1.013 – 1.087)	0.007*	1.046 (1.012 – 1.081)	0.008*	1.070 (1.020 – 1.124)	0.007*
Sodium		(–)		1.030 (0.988 – 1.074)	0.160	1.070 (0.995 – 1.151)	0.069
Potassium		0.881 (0.659 – 1.177)	0.396	(–)		(–)	
Surgery and admission	Surgery severity: Moderate	2.772 (2.133 – 3.604)	<0.001*	1.883 (1.336 – 2.653)	<0.001*	1.199 (0.628 – 2.287)	0.582
	Surgery severity: High	10.052 (6.388 – 15.819)	<0.001*	4.253 (2.692 – 6.721)	<0.001*	2.749 (1.245 – 6.069)	0.012*
Surgery scheduled type (emergency)		4.823 (3.665 – 6.347)	<0.001*	3.168 (2.235 – 4.489)	<0.001*	5.041 (2.396 – 10.604)	<0.001*
Preoperative ICU admission		(–)		2.490 (0.776 – 7.993)	0.125	3.257 (0.840 – 12.623)	0.088

Abbreviations: (-): a variable included at the initial regression stage, excluded in the final model by stepwise selection process. Abbreviations: OR, Odds Ratio; CVD, Clavien-Dindo surgical complication grade; POP, Postoperative; OR, Odds Ratio; ACCI, Age-Adjusted Charlson Comorbidity Index; ICU, Intensive Care Unit. Estimated ORs of pooled values using the multiple-imputed training dataset.

eFigure 1. Real-life clinical cases demonstrating the clinical application of the GERIATRIC risk calculator.

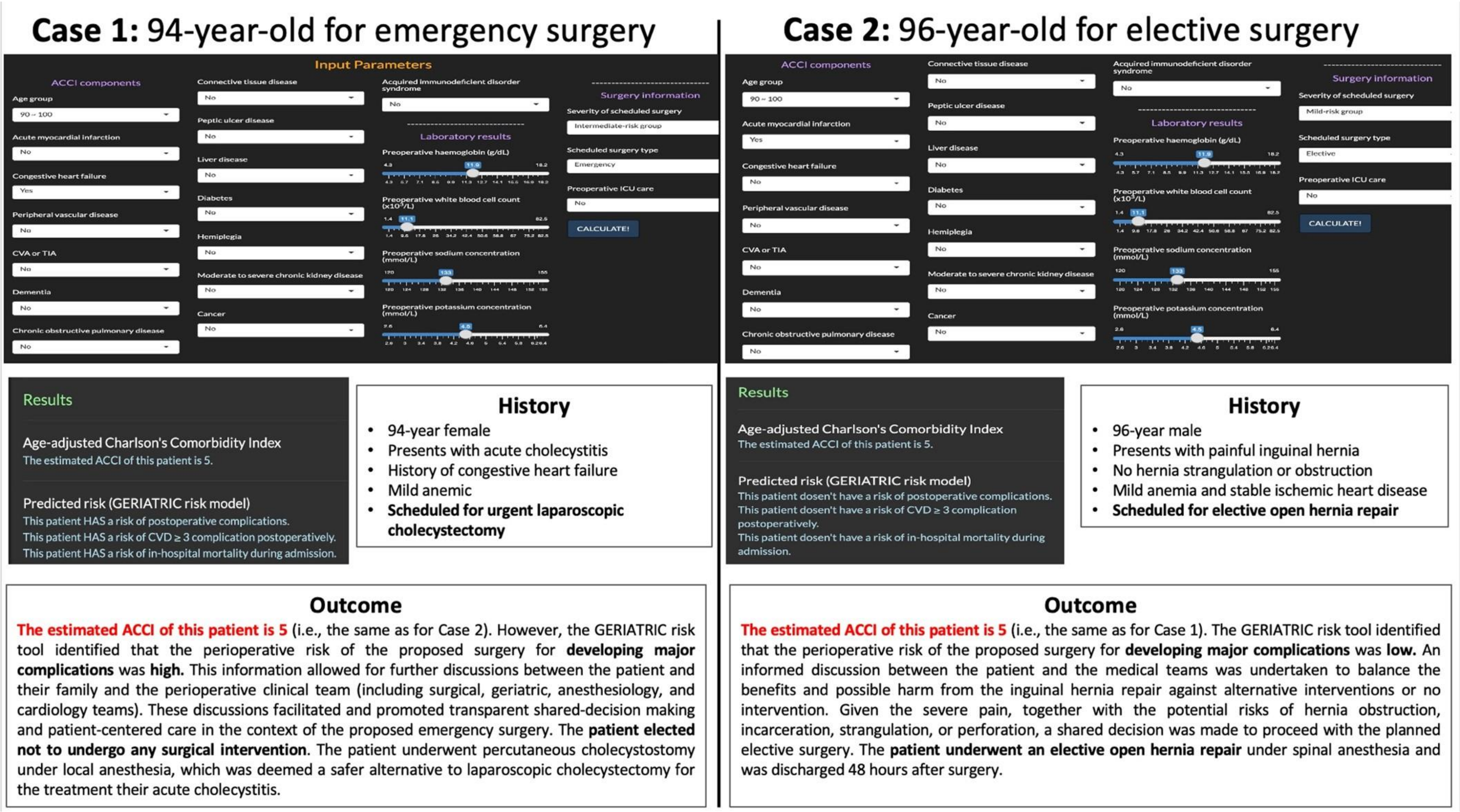

Supplement: Supplementary file 1 [file as9-5-e524-s001.pdf]
