# Supplementary material for: The Perioperative NonaGEnaRIan And cenTenarian suRgICal (GERIATRIC) Risk Stratification Tool
Source: Ann Surg Open. 2024 Nov 18;5(4):e524. doi: 10.1097/AS9.0000000000000524 (PMC11661723; doi:10.1097/AS9.0000000000000524)
Supplement: Supplementary file 2 [file as9-5-e524-s002.pdf]

Supplementary File 2: Outlier handling and missing values

We explored the collected preoperative parameters for outliers and range checks. For the continuous variables, any value outside the 1.5 times interquartile range from the 1st and 3rd quartiles was considered an outlier. For the categorical variables, we examined the value of unusual low proportions. All detected outliers were reconciled manually with the patient's original medical record. If the value was not recorded, it was treated as a missing value.

We also evaluated the missing values in the training dataset. Missing data patterns were visualized and checked using R packages VIM[1] and mice.[2] For the continuous variables, missingness mechanisms were evaluated with Little's missing completely at random (MCAR) test using the R package naniar.[3] As described above, we avoided imputing and replacing missing values in a large proportion. Only the candidate variables as input parameters for the risk model prediction were considered for the missing imputation.

Missing value imputation was performed using the multivariate imputation by chained equations algorithm, which is based on the regression models with information from all other input parameters. The R package mice[2] provided this algorithm and generated the 5 imputed datasets and 10 iterations for each imputed dataset by the predictive mean matching method. The convergence of the imputation procedure was visually checked. Imputation diagnosis was also visually checked using the strip and density plot of observed and imputed values.

During the GERIATRIC model development, a sensitivity analysis was applied for the imputed dataset. The sensitivity analysis was performed with the use of delta adjustment method. To obtain an adjusted imputation model, we add a fixed amount (delta) from the imputed values. The adjustment was applied to the preoperative hemoglobin. Given that the imputed hemoglobin values were used to input other missing values the delta would also affect the imputations, the set delta adjustment was 0 for assuming missing at random, and the other two plausible values that assume a missing not at random (MNAR) situation; one SD higher than the mean of imputed preoperative hemoglobin values (small effect), two SD higher than the mean of imputed preoperative hemoglobin values (large effect).[4] The estimated ORs and corresponding 95% CI were compared using delta-adjusted imputation data.

References

1. Alexander Kowarik, Matthias Templ (2016). Imputation with the R Package VIM. Journal of Statistical Software, 74(7), 1-16. doi:10.18637/jss.v074.i07

2. Stef van Buuren, Karin Groothuis-Oudshoorn (2011). mice: Multivariate Imputation by Chained Equations in R. Journal of Statistical Software, 45(3), 1-67. DOI 10.18637/jss.v045.i03

3. Nicholas Tierney, Di Cook, Miles McBain and Colin Fay (2021). naniar: Data Structures, Summaries, and Visualisations for Missing Data. R package version 0.6.1. https://CRAN.R-project.org/package=naniar

4. Van Buuren, Stef. Flexible imputation of missing data. CRC press, 2018. Ch 9: Measurement issues, PP259-294
