# Supplementary material for: The Perioperative NonaGEnaRIan And cenTenarian suRgICal (GERIATRIC) Risk Stratification Tool
Source: Ann Surg Open. 2024 Nov 18;5(4):e524. doi: 10.1097/AS9.0000000000000524 (PMC11661723; doi:10.1097/AS9.0000000000000524)
Supplement: Supplementary file 3 [file as9-5-e524-s003.pdf]

1 Data Explanations

2 Validation of the ACCI as a postoperative complication prediction model

3 Random data extraction

4 Input parameter screening and selection for the model development

5 Missing analysis and Multiple Imputation

6 Risk prediction model development: Presence of any complications

7 Risk prediction model development: Presence of CVD3 or higher complications

8 Risk prediction model development: In-hospital death

9 References: Interpretation of the calibrations

The risk score to predict postoperative complications in nonagenarians and centenarians
# : Core Statistical Analysis Code

10 Sep 2022 / Revised 29 Oct 2022 / 23 Apr 2023 / 06 May 2023 / 08 May 2023

1 Data Explanations

Data file: "preop_acci_master.csv"
This data file is trimmed from the original master dataset.
It has the parameters of demographics, detailed age-adjusted Charlson Comorbidity score index components, preoperative laboratory findings, surgical procedure characteristics, ASA physical classifications, Preoperative ICU care. Also, it has information on postoperative complications, numbers, severity of complications, and mortality indicators. This dataset has indicators of training, test, and validation datasets for reproducible data splitting. We produced these indicators using the Bernoulli distribution.
Please refer to the manuscript for detailed information on data splitting.

1.1 Data summary

```
master <- read.csv("preop_acci_master.csv"
                   , header = TRUE
                   , fileEncoding = "UTF-8-BOM"
                   , stringsAsFactors = TRUE)

dim(master)
```

```
## [1] 3085   40
```

A total of 3085 cases, 40 variables.

1.2 Define factor variables

Factor variables are:
Variable selection, sex, ACCI components, Hospital admission type, surgery severity, surgery scheduled type, ASA classification, preoperative ICU care.

```
## 'data.frame':    3085 obs. of  40 variables:
##  $ Sel_train          : int  0 0 1 1 1 1 0 1 1 0 ...
##  $ Sel_test           : int  0 1 0 0 0 0 0 0 0 1 ...
##  $ Sel_validate       : int  1 0 0 0 0 0 1 0 0 0 ...
##  $ Patient.No         : int  1 2 3 4 5 6 7 8 9 10 ...
##  $ GENDER             : int  1 1 1 0 0 1 1 1 0 0 ...
##  $ AGE                : int  90 91 91 93 91 90 90 96 93 93 ...
##  $ CIA_AMI            : int  0 0 0 0 0 0 0 0 0 0 ...
##  $ CIA_CHF            : int  0 1 0 0 0 0 0 0 0 0 ...
##  $ CIA_PVD            : int  0 0 0 0 0 0 0 0 0 1 ...
##  $ CIA_CEVD           : int  0 0 0 0 0 0 0 0 0 0 ...
##  $ CIA_DEM            : int  0 0 0 0 0 0 0 0 0 0 ...
##  $ CIA_COPD           : int  0 0 0 0 0 0 0 0 0 0 ...
##  $ CIA_RHEUM          : int  0 0 0 0 0 0 0 0 0 0 ...
##  $ CIA_PUD            : int  0 0 0 0 0 0 0 0 0 0 ...
##  $ CIA_LD_MLD         : int  0 0 0 0 0 0 0 0 0 0 ...
##  $ CIA_LD_SEV         : int  0 0 0 0 0 0 0 0 0 0 ...
##  $ CIA_DM             : int  0 0 0 0 0 0 0 0 0 0 ...
##  $ CIA_DM_CX          : int  0 0 0 0 0 0 0 0 0 0 ...
##  $ CIA_HEMIPLAEGIA    : int  0 0 0 0 0 0 0 0 0 0 ...
##  $ CIA_CKD            : int  0 1 0 0 0 0 0 0 0 0 ...
##  $ CIA_CA             : int  0 0 0 0 0 0 0 0 0 0 ...
##  $ CIA_CA_MET         : int  0 0 0 0 0 0 0 0 0 0 ...
##  $ CIA_HIV            : int  0 0 0 0 0 0 0 0 0 0 ...
##  $ HOSPITAL_ADMIT_TYPE: int  1 0 0 0 1 1 0 0 1 1 ...
##  $ PREOP_HB           : int  115 95 119 124 NA 144 121 133 110 105 ...
##  $ PREOP_WCC          : num  7.9 10.2 9.4 11 NA 6.1 4.1 11.2 7.6 6 ...
##  $ PREOP_NA           : int  138 144 146 134 NA 142 140 141 139 136 ...
##  $ PREOP_K            : num  4.8 3.6 4 4.4 NA 4.7 4.5 4.7 4.2 4.9 ...
##  $ SURG_SEV           : int  1 0 2 1 1 0 2 2 1 1 ...
##  $ SURG_SCHEDULED_TYPE: int  0 0 1 1 0 0 1 1 0 0 ...
##  $ ASAc               : int  1 1 2 1 1 1 1 2 1 1 ...
##  $ PreopICU           : int  0 0 0 0 0 0 0 0 0 0 ...
##  $ Complication10     : int  0 1 1 0 0 1 1 1 0 1 ...
##  $ NoCx               : int  0 2 2 0 0 2 7 1 0 1 ...
##  $ CVD                : int  0 2 4 0 0 2 2 5 0 2 ...
##  $ CVD_23             : int  0 0 1 0 0 0 0 1 0 0 ...
##  $ DECEASED           : int  1 1 0 0 0 0 0 1 0 0 ...
##  $ DEATH_INHOSPITAL   : int  0 0 0 0 0 0 0 1 0 0 ...
##  $ DEATH_90_OH        : int  0 0 0 0 0 0 0 1 0 0 ...
##  $ ACCI_corrected     : int  5 8 5 5 5 5 5 5 5 6 ...
```

```
# Converted result
str(master)
```

```
## 'data.frame':    3085 obs. of  40 variables:
##  $ Sel_train          : Factor w/ 2 levels "0","1": 1 1 2 2 2 2 1 2 2 1 ...
##  $ Sel_test           : Factor w/ 2 levels "0","1": 1 2 1 1 1 1 1 1 1 2 ...
##  $ Sel_validate       : Factor w/ 2 levels "0","1": 2 1 1 1 1 1 2 1 1 1 ...
##  $ Patient.No         : int  1 2 3 4 5 6 7 8 9 10 ...
##  $ GENDER             : Factor w/ 2 levels "0","1": 2 2 2 1 1 2 2 2 1 1 ...
##  $ AGE                : int  90 91 91 93 91 90 90 96 93 93 ...
##  $ CIA_AMI            : Factor w/ 2 levels "0","1": 1 1 1 1 1 1 1 1 1 1 ...
##  $ CIA_CHF            : Factor w/ 2 levels "0","1": 1 2 1 1 1 1 1 1 1 1 ...
##  $ CIA_PVD            : Factor w/ 2 levels "0","1": 1 1 1 1 1 1 1 1 1 2 ...
##  $ CIA_CEVD           : Factor w/ 2 levels "0","1": 1 1 1 1 1 1 1 1 1 1 ...
##  $ CIA_DEM            : Factor w/ 2 levels "0","1": 1 1 1 1 1 1 1 1 1 1 ...
##  $ CIA_COPD           : Factor w/ 2 levels "0","1": 1 1 1 1 1 1 1 1 1 1 ...
##  $ CIA_RHEUM          : Factor w/ 2 levels "0","1": 1 1 1 1 1 1 1 1 1 1 ...
##  $ CIA_PUD            : Factor w/ 2 levels "0","1": 1 1 1 1 1 1 1 1 1 1 ...
##  $ CIA_LD_MLD         : Factor w/ 2 levels "0","1": 1 1 1 1 1 1 1 1 1 1 ...
##  $ CIA_LD_SEV         : Factor w/ 2 levels "0","1": 1 1 1 1 1 1 1 1 1 1 ...
##  $ CIA_DM             : Factor w/ 2 levels "0","1": 1 1 1 1 1 1 1 1 1 1 ...
##  $ CIA_DM_CX          : Factor w/ 2 levels "0","1": 1 1 1 1 1 1 1 1 1 1 ...
##  $ CIA_HEMIPLAEGIA    : Factor w/ 2 levels "0","1": 1 1 1 1 1 1 1 1 1 1 ...
##  $ CIA_CKD            : Factor w/ 2 levels "0","1": 1 2 1 1 1 1 1 1 1 1 ...
##  $ CIA_CA             : Factor w/ 2 levels "0","1": 1 1 1 1 1 1 1 1 1 1 ...
##  $ CIA_CA_MET         : Factor w/ 2 levels "0","1": 1 1 1 1 1 1 1 1 1 1 ...
##  $ CIA_HIV            : Factor w/ 1 level "0": 1 1 1 1 1 1 1 1 1 1 ...
##  $ HOSPITAL_ADMIT_TYPE: Factor w/ 3 levels "0","1","2": 2 1 1 1 2 2 1 1 2 2 ...
##  $ PREOP_HB           : int  115 95 119 124 NA 144 121 133 110 105 ...
##  $ PREOP_WCC          : num  7.9 10.2 9.4 11 NA 6.1 4.1 11.2 7.6 6 ...
##  $ PREOP_NA           : int  138 144 146 134 NA 142 140 141 139 136 ...
##  $ PREOP_K            : num  4.8 3.6 4 4.4 NA 4.7 4.5 4.7 4.2 4.9 ...
##  $ SURG_SEV           : Factor w/ 3 levels "0","1","2": 2 1 3 2 2 1 3 3 2 2 ...
##  $ SURG_SCHEDULED_TYPE: Factor w/ 2 levels "0","1": 1 1 2 2 1 1 2 2 1 1 ...
##  $ ASAc               : Factor w/ 3 levels "0","1","2": 2 2 3 2 2 2 2 3 2 2 ...
##  $ PreopICU           : Factor w/ 2 levels "0","1": 1 1 1 1 1 1 1 1 1 1 ...
##  $ Complication10     : int  0 1 1 0 0 1 1 1 0 1 ...
##  $ NoCx               : int  0 2 2 0 0 2 7 1 0 1 ...
##  $ CVD                : int  0 2 4 0 0 2 2 5 0 2 ...
##  $ CVD_23             : int  0 0 1 0 0 0 0 1 0 0 ...
##  $ DECEASED           : int  1 1 0 0 0 0 0 1 0 0 ...
##  $ DEATH_INHOSPITAL   : int  0 0 0 0 0 0 0 1 0 0 ...
##  $ DEATH_90_OH        : int  0 0 0 0 0 0 0 1 0 0 ...
##  $ ACCI_corrected     : int  5 8 5 5 5 5 5 5 5 6 ...
```

```
summary(master)
```

```
##  Sel_train Sel_test Sel_validate   Patient.No   GENDER        AGE
##  0:1117    0:2593   0:2460       Min.   :   1   0:1534   Min.   : 90.00
##  1:1968    1: 492   1: 625       1st Qu.: 774   1:1551   1st Qu.: 90.00
##                                  Median :1551            Median : 92.00
##                                  Mean   :1550            Mean   : 92.26
##                                  3rd Qu.:2326            3rd Qu.: 93.00
##                                  Max.   :3103            Max.   :105.00
##
##  CIA_AMI  CIA_CHF  CIA_PVD  CIA_CEVD CIA_DEM  CIA_COPD CIA_RHEUM CIA_PUD
##  0:3070   0:2983   0:3019   0:3026   0:2962   0:3041   0:3083    0:3044
##  1:  15   1: 102   1:  66   1:  59   1: 123   1:  44   1:   2    1:  41
##
##
##
##
##
##  CIA_LD_MLD CIA_LD_SEV CIA_DM   CIA_DM_CX CIA_HEMIPLAEGIA CIA_CKD  CIA_CA
##  0:3074     0:3080     0:2870   0:2820    0:3058          0:2905   0:2813
##  1:  11     1:   5     1: 215   1: 265    1:  27          1: 180   1: 272
##
##
##
##
##
##  CIA_CA_MET CIA_HIV  HOSPITAL_ADMIT_TYPE    PREOP_HB       PREOP_WCC
##  0:3024     0:3085   0:1038              Min.   : 43.0   Min.   : 1.40
##  1:  61              1:2041              1st Qu.:106.0   1st Qu.: 6.10
##                      2:   6              Median :121.0   Median : 7.60
##                                          Mean   :119.2   Mean   : 8.34
##                                          3rd Qu.:134.0   3rd Qu.: 9.60
##                                          Max.   :182.0   Max.   :82.50
##                                          NA's   :582     NA's   :582
##     PREOP_NA        PREOP_K      SURG_SEV SURG_SCHEDULED_TYPE ASAc     PreopICU
##  Min.   :120.0   Min.   :2.600   0:1674   0:2090              0:  22   0:3064
##  1st Qu.:138.0   1st Qu.:4.100   1:1121   1: 995              1:2976   1:  21
##  Median :140.0   Median :4.500   2: 290                       2:  87
##  Mean   :139.5   Mean   :4.469
##  3rd Qu.:142.0   3rd Qu.:4.800
##  Max.   :155.0   Max.   :6.400
##  NA's   :583     NA's   :594
##  Complication10        NoCx             CVD            CVD_23
##  Min.   :0.0000   Min.   : 0.000   Min.   :0.0000   Min.   :0.0000
##  1st Qu.:0.0000   1st Qu.: 0.000   1st Qu.:0.0000   1st Qu.:0.0000
##  Median :0.0000   Median : 0.000   Median :0.0000   Median :0.0000
##  Mean   :0.3235   Mean   : 1.215   Mean   :0.8995   Mean   :0.1442
##  3rd Qu.:1.0000   3rd Qu.: 1.000   3rd Qu.:2.0000   3rd Qu.:0.0000
##  Max.   :1.0000   Max.   :25.000   Max.   :5.0000   Max.   :1.0000
##
##     DECEASED      DEATH_INHOSPITAL   DEATH_90_OH     ACCI_corrected
##  Min.   :0.0000   Min.   :0.00000   Min.   :0.0000   Min.   : 5.000
##  1st Qu.:0.0000   1st Qu.:0.00000   1st Qu.:0.0000   1st Qu.: 5.000
##  Median :0.0000   Median :0.00000   Median :0.0000   Median : 5.000
##  Mean   :0.2347   Mean   :0.03533   Mean   :0.0658   Mean   : 5.836
##  3rd Qu.:0.0000   3rd Qu.:0.00000   3rd Qu.:0.0000   3rd Qu.: 6.000
##  Max.   :1.0000   Max.   :1.00000   Max.   :1.0000   Max.   :14.000
##
```

1.3 Variables dictionary

Sel: Flags of training, testing and validation datasets.
Patient.No: Patient's ID
GENDER: 0 = Female, 1= Male
CIA_AMI: Age-adjusted Charlson's comorbidity score index flag for acute myocardial infarct
CIA_CHF: Age-adjusted Charlson's comorbidity score index flag for congestive heart failure
CIA_PVD: Age-adjusted Charlson's comorbidity score index flag for Peripheral vascular disease
CIA_CEVD: Age-adjusted Charlson's comorbidity score index flag for Cerebrovascular disease
CIA_DEM: Age-adjusted Charlson's comorbidity score index flag for dementia
CIA_COPD: Age-adjusted Charlson's comorbidity score index flag for chronic obstructive pulmonary disease
CIA_RHEUM: Age-adjusted Charlson's comorbidity score index flag for rheumatic disease
CIA_PUD: Age-adjusted Charlson's comorbidity score index flag for peptic ulcer disease
CIA_LD_MLD: Age-adjusted Charlson's comorbidity score index flag for mild liver disease
CIA_LD_SEV: Age-adjusted Charlson's comorbidity score index flag for severe liver disease
CIA_DM: Age-adjusted Charlson's comorbidity score index flag for non-complicated diabetes mellitus
CIA_DM_CX: Age-adjusted Charlson's comorbidity score index flag for complicated diabetes mellitus
CIA_HEMIPLAEGIA: Age-adjusted Charlson's comorbidity score index flag for hemiplaegia
CIA_CKD: Age-adjusted Charlson's comorbidity score index flag for chronic kidney disease
CIA_CA: Age-adjusted Charlson's comorbidity score index flag for cancer without metastasis
CIA_CA_MET: Age-adjusted Charlson's comorbidity score index flag for cancer with metastasis
CIA_HIV: Age-adjusted Charlson's comorbidity score index flag for HIV infection
HOSPITAL_ADMIT_TYPE: First inpatient episode admission type 0 = Emergency Admission, 1 = Planned Admission, 3 = Other, Blank = Missing
PREOP_HB: Preoperative Haemoglobin
PREOP_WCC: Preoperative white blood cell count
PREOP_NA: Preoperative sodium
PREOP_K: Preoperative potassium
SURG_SEV: Severity of performed surgery. 0 = Mild, 1 = intermediate, 2 = high
SURG_SCHEDULED_TYPE: 0 = Elective, 1 = Emergency
ASAc: 0 = ASA I & II, 1 = ASA III, 2 = ASA IV & V
PreopICU: Patient admitted to ICU pre-operatively. 0 = No, 1 = Yes
CVD_23: Any postoperative complications. 0 = None, 1 = Yes
NoCx: Number of complications
CVD: Clavien-Dindo postoperative surgical complication grade
CVD_23: Categorised CVD grade. 0 = Not complicated or CVD 1 & 2 complications, 1 = CVD 3 & 4 complications
DECEASED: 0 = Alive, 1 = Deceased during observation period.
DEATH_INHOSPITAL: In-hospital death. 0 = Alive, 1 = In-hospital death
DEATH_90_OH: Death within postoperative 90 days. 0 = Alive, 1 = death within postoperative 90 days
ACCI_corredted: Age-adjusted Charlson's comorbidity score

```
library(dplyr)

master <- master %>% mutate(PREOP_HB = PREOP_HB/10)
```

We transformed the haemoglobin concentration unit from g/dL to 10×g/dL, to clarify the preoperative haemoglobin effect on postoperative complications.

2 Validation of the ACCI as a postoperative complication prediction model

2.1 Discrimination ability of ACCI for any postoperative complications

```
library(pROC)
```

```
roc.acci.anycx <- roc(master$CVD_23, master$ACCI_corrected)
```

```
## Setting levels: control = 0, case = 1
```

```
## Setting direction: controls < cases
```

```
roc.acci.anycx$auc
```

```
## Area under the curve: 0.7144
```

```
ci(roc.acci.anycx)
```

```
## 95% CI: 0.6892-0.7396 (DeLong)
```

```
coords(roc.acci.anycx,
       "best",
       transpose = TRUE,
       best.method = "youden")
```

```
##   threshold specificity sensitivity
##   5.5000000   0.6787879   0.7078652
```

```
plot.roc(roc.acci.anycx
         , print.auc = TRUE
         , print.thres = FALSE
         , auc.polygon = TRUE
         )
```

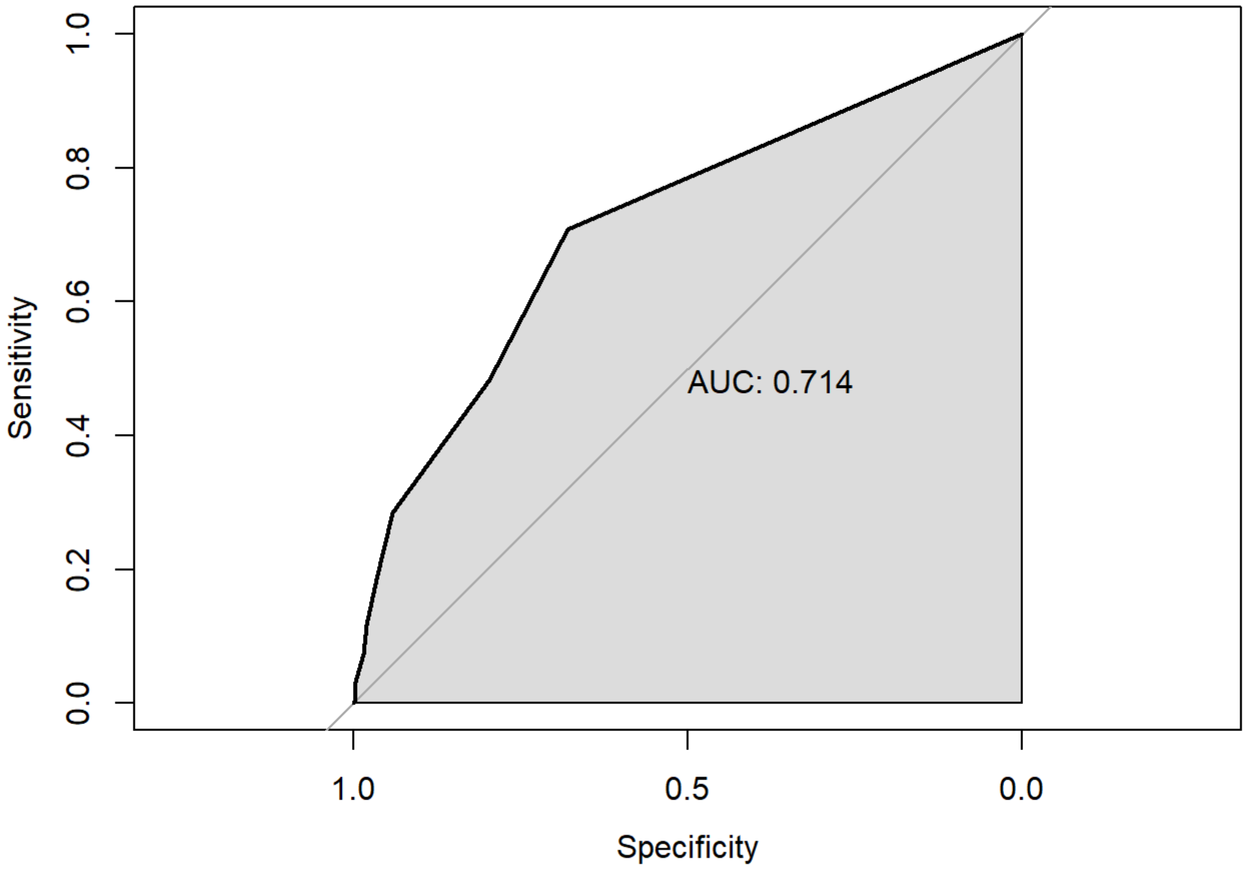

2.1.1 Discrimination ability of ACCI for any postoperative complications:
According to the surgery severity

2.1.1.1 Low surgical risk

```
master.lowrisk <- filter(master, SURG_SEV == 0)

roc.acci.anycx.low <- roc(master.lowrisk$CVD_23,
                          master.lowrisk$ACCI_corrected
                          )
```

```
## Setting levels: control = 0, case = 1
```

```
## Setting direction: controls < cases
```

```
roc.acci.anycx.low$auc
```

```
## Area under the curve: 0.7487
```

```
ci(roc.acci.anycx.low)
```

```
## 95% CI: 0.7028-0.7945 (DeLong)
```

```
coords(roc.acci.anycx.low,
       "best",
       transpose = TRUE,
       best.method = "youden")
```

```
##   threshold specificity sensitivity
##   5.5000000   0.7236927   0.7120000
```

```
plot.roc(roc.acci.anycx.low
         , print.auc = TRUE
         , print.thres = FALSE
         , auc.polygon = TRUE
)
```

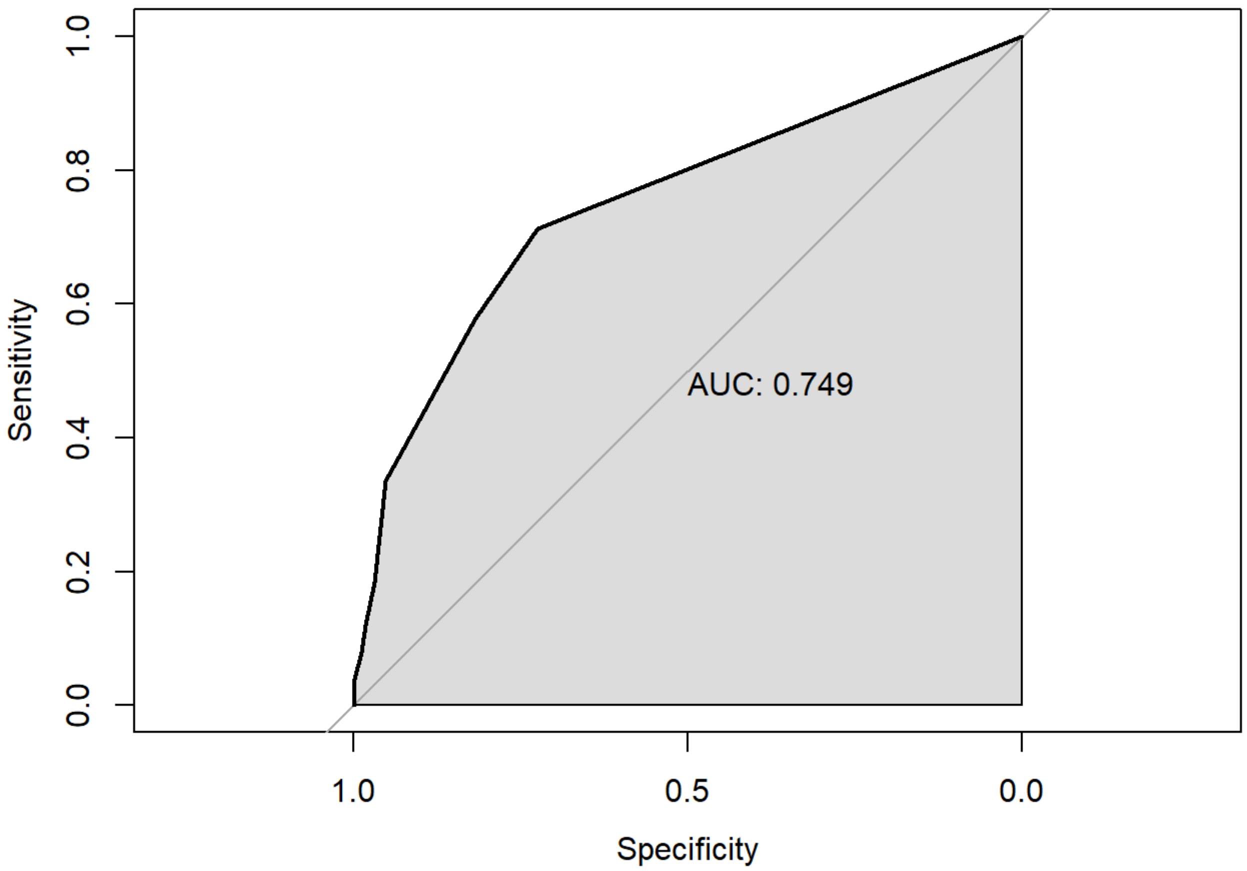

2.1.1.2 Intermediate surgical risk

```
master.imrisk <- filter(master, SURG_SEV == 1)

roc.acci.anycx.im <- roc(master.imrisk$CVD_23,
                         master.imrisk$ACCI_corrected
                         )
```

```
## Setting levels: control = 0, case = 1
```

```
## Setting direction: controls < cases
```

```
roc.acci.anycx.im$auc
```

```
## Area under the curve: 0.6918
```

```
ci(roc.acci.anycx.im)
```

```
## 95% CI: 0.6535-0.73 (DeLong)
```

```
coords(roc.acci.anycx.im,
       "best",
       transpose = TRUE,
       best.method = "youden")
```

```
##   threshold specificity sensitivity
##   5.5000000   0.6355653   0.7095238
```

```
plot.roc(roc.acci.anycx.im
         , print.auc = TRUE
         , print.thres = FALSE
         , auc.polygon = TRUE
)
```

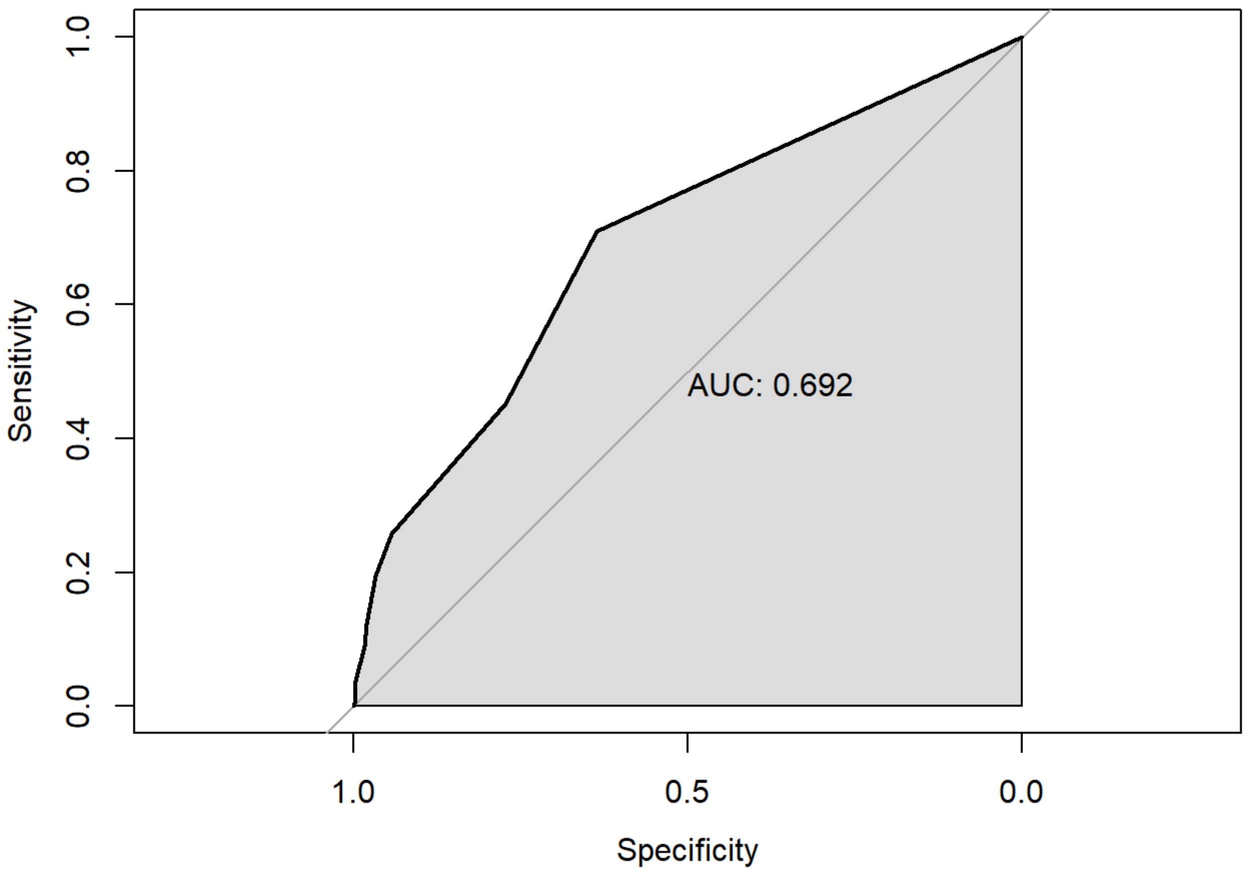

2.1.1.3 High surgical risk

```
master.hirisk <- filter(master, SURG_SEV == 2)

roc.acci.anycx.hi <- roc(master.hirisk$CVD_23,
                         master.hirisk$ACCI_corrected
                           )
```

```
## Setting levels: control = 0, case = 1
```

```
## Setting direction: controls < cases
```

```
roc.acci.anycx.hi$auc
```

```
## Area under the curve: 0.6272
```

```
ci(roc.acci.anycx.hi)
```

```
## 95% CI: 0.5637-0.6907 (DeLong)
```

```
coords(roc.acci.anycx.hi,
       "best",
       transpose = TRUE,
       best.method = "youden")
```

```
##   threshold specificity sensitivity
##   5.5000000   0.5111111   0.7000000
```

```
plot.roc(roc.acci.anycx.hi
         , print.auc = TRUE
         , print.thres = FALSE
         , auc.polygon = TRUE
)
```

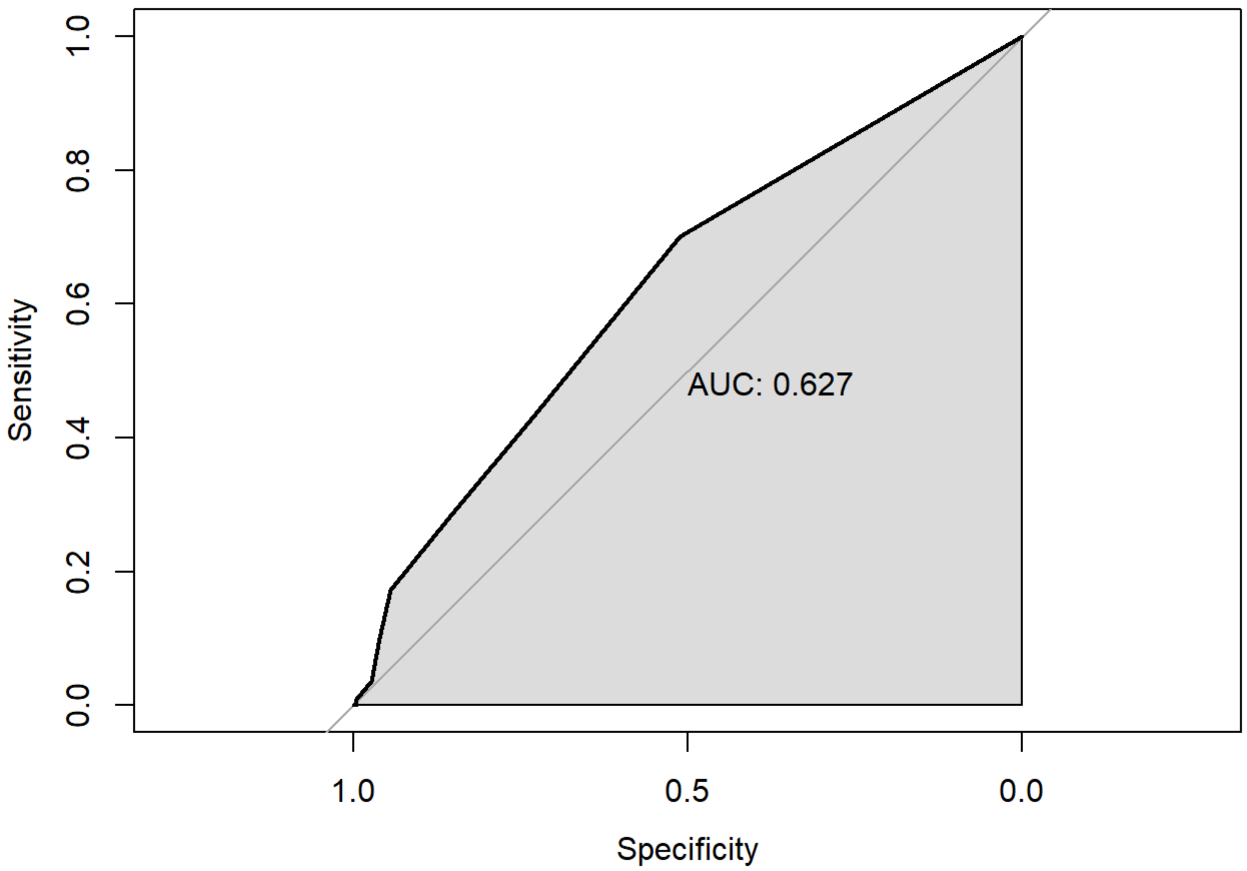

2.2 Discrimination ability of ACCI for CVD 3 or higher complication

```
roc.acci.cvd23 <- roc(master$CVD_23,
                      master$ACCI_corrected
                      )
```

```
## Setting levels: control = 0, case = 1
```

```
## Setting direction: controls < cases
```

```
roc.acci.cvd23$auc
```

```
## Area under the curve: 0.7144
```

```
ci(roc.acci.cvd23)
```

```
## 95% CI: 0.6892-0.7396 (DeLong)
```

```
coords(roc.acci.cvd23,
       "best",
       transpose = TRUE,
       best.method = "youden")
```

```
##   threshold specificity sensitivity
##   5.5000000   0.6787879   0.7078652
```

```
plot.roc(roc.acci.cvd23
         , print.auc = TRUE
         , print.thres = FALSE
         , auc.polygon = TRUE
)
```

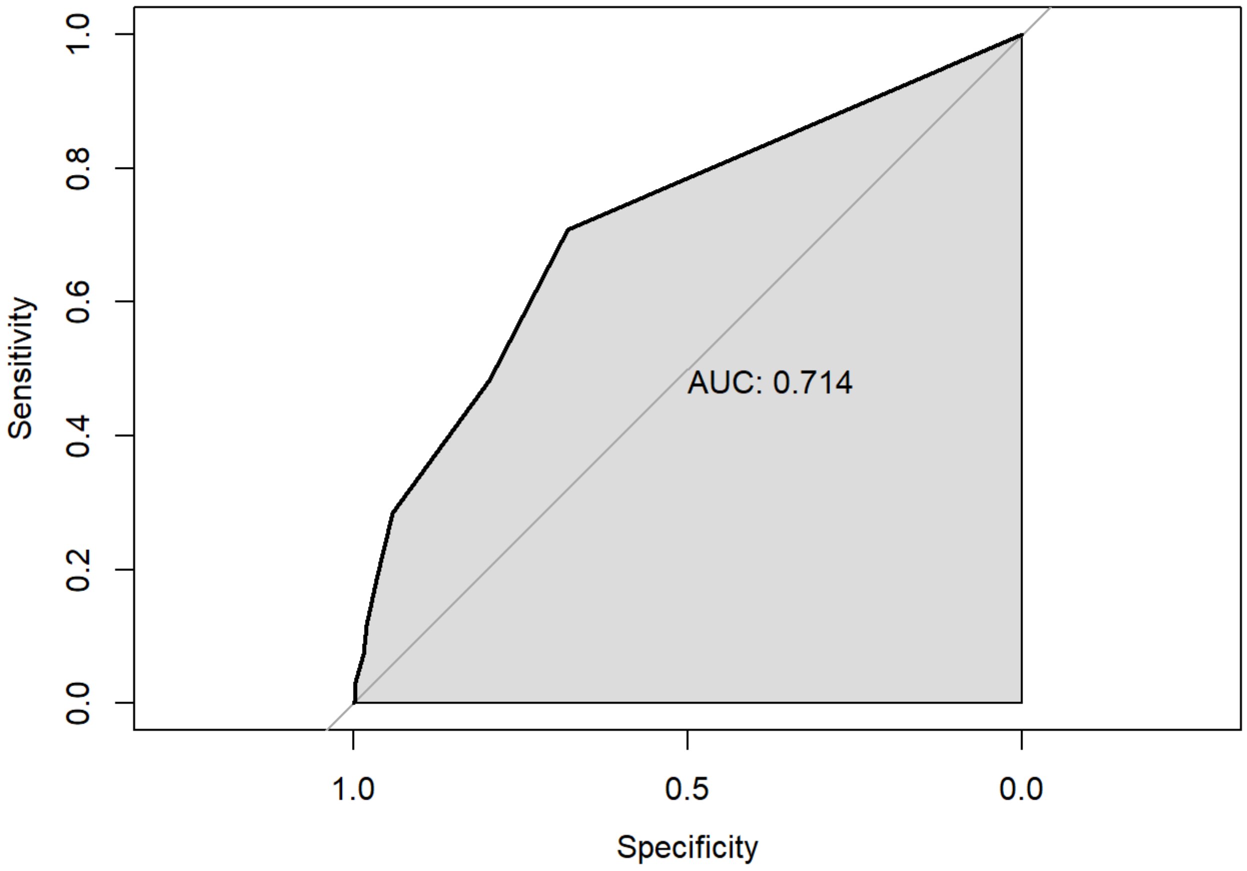

2.3 Discrimination ability of ACCI for in-hospital death

```
roc.acci.ihdeath <- roc(master$DEATH_INHOSPITAL,
                        master$ACCI_corrected
                        )
```

```
## Setting levels: control = 0, case = 1
```

```
## Setting direction: controls < cases
```

```
roc.acci.ihdeath$auc
```

```
## Area under the curve: 0.7359
```

```
ci(roc.acci.ihdeath)
```

```
## 95% CI: 0.6871-0.7847 (DeLong)
```

```
coords(roc.acci.ihdeath,
       "best",
       transpose = TRUE,
       best.method = "youden")
```

```
##   threshold specificity sensitivity
##   5.5000000   0.6367608   0.7522936
```

```
plot.roc(roc.acci.ihdeath
         , print.auc = TRUE
         , print.thres = FALSE
         , auc.polygon = TRUE
)
```

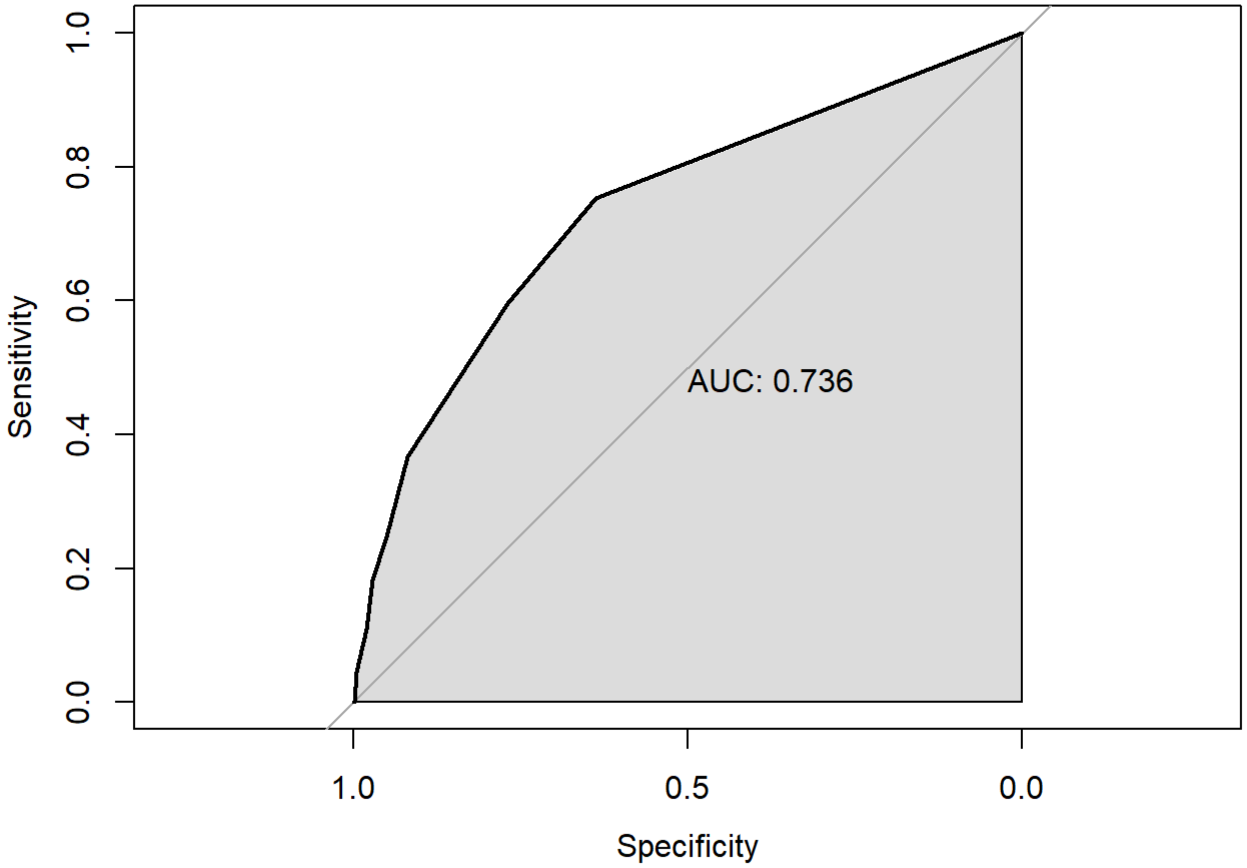

3 Random data extraction

Using the predefined randomization variables, we seperate the whole data (N=3085) into
1. Training data: for model development, N = 1968, 63.8% of the whole data
2. Test data: for estimated model performance validation, N = 492, 15.9% of the whole data
3. Validation data: for Re-Calibration of the estimated model if required, N = 625, 20.3% of the whole data

The following code split and generate the datasets and summarise the results.

```
#original data Splitting

train.org <- master%>%filter(Sel_train==1)
test.org <- master%>%filter(Sel_test==1)
valid.org <- master%>%filter(Sel_validate==1)

# Summarise the splitting results
random.result <-
  rbind(
    cbind( table(master$Complication10)[2]
       , table(train.org$Complication10)[2]
       , table(test.org$Complication10)[2]
       , table(valid.org$Complication10)[2])
    , cbind( table(master$CVD_23)[2]
             , table(train.org$CVD_23)[2]
             , table(test.org$CVD_23)[2]
             , table(valid.org$CVD_23)[2])
    , cbind( table(master$DEATH_INHOSPITAL)[2]
             , table(train.org$DEATH_INHOSPITAL)[2]
             , table(test.org$DEATH_INHOSPITAL)[2]
             , table(valid.org$DEATH_INHOSPITAL)[2])
    , cbind( table(master$DEATH_90_OH)[2]
             , table(train.org$DEATH_90_OH)[2]
             , table(test.org$DEATH_90_OH)[2]
             , table(valid.org$DEATH_90_OH)[2])
  )

rownames(random.result) <- c("Any complications"
                             , "CVD≥3"
                             , "In-hospital death"
                             , "Death within 90 days")

colnames(random.result) <- c("Whole data"
                             , "Training data"
                             , "Testing data"
                             , "Validation data")

random.result
```

```
##                        Whole data Training data Testing data Validation data
## Any complications             998           647          148             203
## CVD≥3                         445           296           61              88
## In-hospital death             109            70           17              22
## Death within 90 days          203           131           31              41
```

4 Input parameter screening and selection for the model development

4.1 Correlation analysis

```
library(Hmisc)
```

```r
master.num <- mutate_all(master, function(x) as.numeric(x)) %>%
  select(!c(Sel_train
          , Sel_test
          , Sel_validate
          , Patient.No
          , HOSPITAL_ADMIT_TYPE
          , ACCI_corrected))



cor.matrix <- rcorr(as.matrix(master.num), type = "spearman")

# Correlation coefficients
cor.matrix$r[c(1:27),c(28,31,33,34)]
```

```
##                      Complication10      CVD_23 DEATH_INHOSPITAL  DEATH_90_OH
## GENDER                 -0.138236249 -0.08622121     -0.034414964 -0.026303296
## AGE                     0.067887853  0.04314931      0.058213844  0.048997924
## CIA_AMI                 0.041314165  0.05088286      0.037105028  0.037833958
## CIA_CHF                 0.240278042  0.23883849      0.131545942  0.155658109
## CIA_PVD                 0.146771151  0.16884313      0.020241213  0.033043616
## CIA_CEVD                0.131097219  0.13802042      0.127108648  0.087032885
## CIA_DEM                 0.202605386  0.13324929      0.032791759  0.032784686
## CIA_COPD                0.133039369  0.07511180      0.065826367  0.067311606
## CIA_RHEUM               0.009609474  0.02578997     -0.004874443 -0.006759729
## CIA_PUD                 0.052862811  0.08125939      0.039118838  0.037699626
## CIA_LD_MLD             -0.006492478 -0.02455967     -0.011448305 -0.015876159
## CIA_LD_SEV              0.006588630 -0.01654199     -0.007710924 -0.010693273
## CIA_DM                  0.012102287 -0.02541059      0.009678276  0.019780807
## CIA_DM_CX               0.153984138  0.12766573      0.029054316  0.021286581
## CIA_HEMIPLAEGIA         0.098684957  0.10009765      0.095120602  0.073317514
## CIA_CKD                 0.238763893  0.30321848      0.184569641  0.196085396
## CIA_CA                 -0.029306163 -0.04306536      0.002412712 -0.004141639
## CIA_CA_MET              0.095890277  0.11399095      0.111546090  0.112559163
## CIA_HIV                         NaN         NaN              NaN          NaN
## PREOP_HB               -0.219569074 -0.18731683     -0.111790279 -0.156743651
## PREOP_WCC               0.189151138  0.13821827      0.114955977  0.115037984
## PREOP_NA               -0.087364505 -0.03667576     -0.001141847 -0.026331468
## PREOP_K                -0.097142723 -0.06072686     -0.036574399 -0.030371304
## SURG_SEV                0.392078232  0.24554976      0.102540993  0.100763881
## SURG_SCHEDULED_TYPE     0.524916701  0.36607798      0.213515065  0.197255582
## ASAc                    0.137909074  0.11708068      0.110342277  0.096409685
## PreopICU                0.094437391  0.11187945      0.154985029  0.121131135
```

```r
# Corresponding P values
cor.matrix$P[c(1:27),c(28,31,33,34)]
```

```
##                     Complication10       CVD_23 DEATH_INHOSPITAL  DEATH_90_OH
## GENDER                1.243450e-14 1.618996e-06     5.596679e-02 1.441201e-01
## AGE                   1.609697e-04 1.653997e-02     1.217316e-03 6.488563e-03
## CIA_AMI               2.174727e-02 4.700674e-03     3.932301e-02 3.561431e-02
## CIA_CHF               0.000000e+00 0.000000e+00     2.211564e-13 0.000000e+00
## CIA_PVD               2.220446e-16 0.000000e+00     2.610501e-01 6.649214e-02
## CIA_CEVD              2.668976e-13 1.376677e-14     1.381784e-12 1.290071e-06
## CIA_DEM               0.000000e+00 1.074696e-13     6.859212e-02 6.865188e-02
## CIA_COPD              1.176836e-13 2.965139e-05     2.535316e-04 1.829965e-04
## CIA_RHEUM             5.936648e-01 1.521140e-01     7.866741e-01 7.074327e-01
## CIA_PUD               3.313924e-03 6.212718e-06     2.980140e-02 3.627445e-02
## CIA_LD_MLD            7.184972e-01 1.726416e-01     5.250151e-01 3.780450e-01
## CIA_LD_SEV            7.145095e-01 3.583685e-01     6.685645e-01 5.527058e-01
## CIA_DM                5.016176e-01 1.582356e-01     5.910238e-01 2.720547e-01
## CIA_DM_CX             0.000000e+00 1.101563e-12     1.066481e-01 2.372174e-01
## CIA_HEMIPLAEGIA       3.964496e-08 2.526347e-08     1.202428e-07 4.579843e-05
## CIA_CKD               0.000000e+00 0.000000e+00     0.000000e+00 0.000000e+00
## CIA_CA                1.036446e-01 1.675182e-02     8.934385e-01 8.181338e-01
## CIA_CA_MET            9.493899e-08 2.159966e-10     5.215997e-10 3.627998e-10
## CIA_HIV                        NaN          NaN              NaN          NaN
## PREOP_HB              0.000000e+00 0.000000e+00     2.050832e-08 3.108624e-15
## PREOP_WCC             0.000000e+00 3.785638e-12     8.045491e-09 7.850165e-09
## PREOP_NA              1.207896e-05 6.662107e-02     9.544762e-01 1.879493e-01
## PREOP_K               1.189110e-06 2.428187e-03     6.798297e-02 1.296666e-01
## SURG_SEV              0.000000e+00 0.000000e+00     1.142075e-08 2.038318e-08
## SURG_SCHEDULED_TYPE   0.000000e+00 0.000000e+00     0.000000e+00 0.000000e+00
## ASAc                  1.421085e-14 6.900924e-11     7.996124e-10 8.086275e-08
## PreopICU              1.480755e-07 4.630292e-10     0.000000e+00 1.477418e-11
```

FYI, we excluded ASA, CIA_HIV, CIA_RHEUM, and HOSPITAL_ADMIT_TYPE.
ASA has a duplicated meaning with ACCI parameters.
CIA_HIV and CIA_RHEUM have a positive number of 0 and 2 in 3083 cases.
HOSPITAL_ADMIT_TYPE also has a duplicated meaning with emergency / elective surgery.
All of these variables could be a cause of bias or multicollinearity.

5 Missing analysis and Multiple Imputation

5.1 Missing data patterns

```
library(mice)
library(VIM)
```

```
md.pattern(master, rotate.names = TRUE)
```

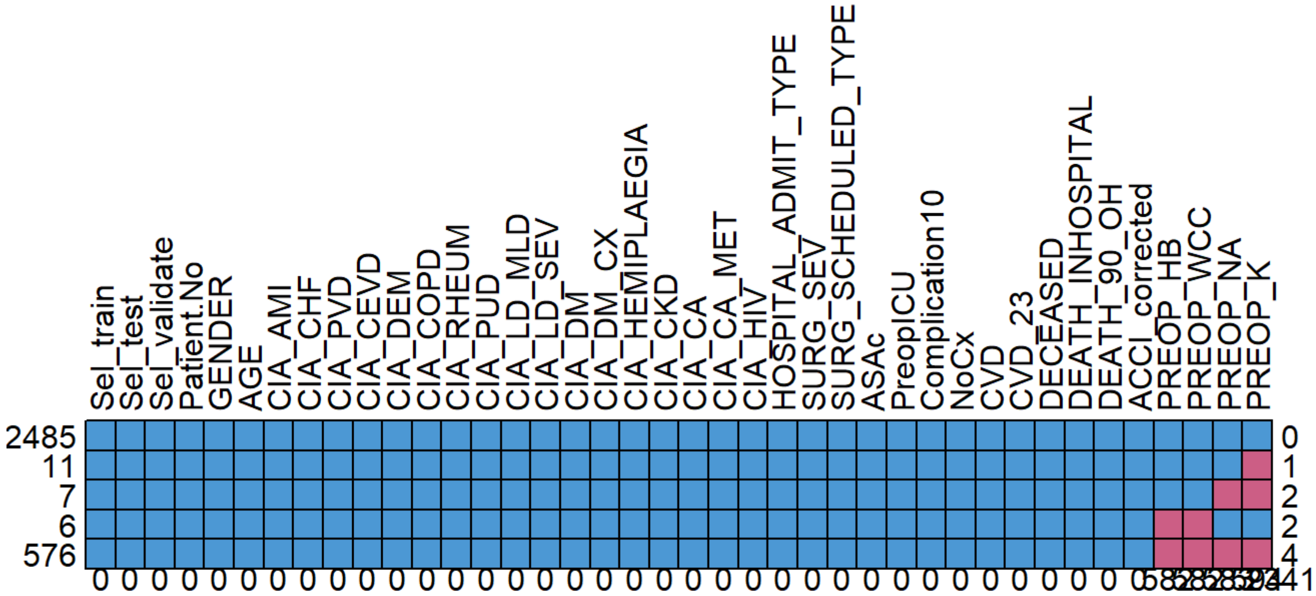
Sel_train
Sel_test
Sel_validate
Patient.No
GENDER
AGE
CIA_AMI
CIA_CHF
CIA_PVD
CIA_CEVD
CIA_DEM
CIA_COPD
CIA_RHEUM
CIA_PUD
CIA_LD_MLD
CIA_LD_SEV
CIA_DM
CIA_DM_CX
CIA_HEMIPLAEGIA
CIA_CKD
CIA_CA
CIA_CA_MET
CIA_HIV
HOSPITAL_ADMIT_TYPE
SURG_SEV
SURG_SCHEDULED_TYPE
ASAc
PreopICU
Complication10
NoCx
CVD
CVD_23
DECEASED
DEATH_INHOSPITAL
DEATH_90_OH
ACCI_corrected
PREOP_HB
PREOP_WCC
PREOP_NA
PREOP_K
2485
11
7
6
576

```
##      Sel_train Sel_test Sel_validate Patient.No GENDER AGE CIA_AMI CIA_CHF
## 2485         1        1            1          1      1   1       1       1
## 11           1        1            1          1      1   1       1       1
## 7            1        1            1          1      1   1       1       1
## 6            1        1            1          1      1   1       1       1
## 576          1        1            1          1      1   1       1       1
##              0        0            0          0      0   0       0       0
##      CIA_PVD CIA_CEVD CIA_DEM CIA_COPD CIA_RHEUM CIA_PUD CIA_LD_MLD CIA_LD_SEV
## 2485       1        1       1        1         1       1          1          1
## 11         1        1       1        1         1       1          1          1
## 7          1        1       1        1         1       1          1          1
## 6          1        1       1        1         1       1          1          1
## 576        1        1       1        1         1       1          1          1
##            0        0       0        0         0       0          0          0
##      CIA_DM CIA_DM_CX CIA_HEMIPLAEGIA CIA_CKD CIA_CA CIA_CA_MET CIA_HIV
## 2485      1         1               1       1      1          1       1
## 11        1         1               1       1      1          1       1
## 7         1         1               1       1      1          1       1
## 6         1         1               1       1      1          1       1
## 576       1         1               1       1      1          1       1
##           0         0               0       0      0          0       0
##      HOSPITAL_ADMIT_TYPE SURG_SEV SURG_SCHEDULED_TYPE ASAc PreopICU
## 2485                   1        1                   1    1        1
## 11                     1        1                   1    1        1
## 7                      1        1                   1    1        1
## 6                      1        1                   1    1        1
## 576                    1        1                   1    1        1
##                        0        0                   0    0        0
##      Complication10 NoCx CVD CVD_23 DECEASED DEATH_INHOSPITAL DEATH_90_OH
## 2485              1    1   1      1        1                1           1
## 11                1    1   1      1        1                1           1
## 7                 1    1   1      1        1                1           1
## 6                 1    1   1      1        1                1           1
## 576               1    1   1      1        1                1           1
##                   0    0   0      0        0                0           0
##      ACCI_corrected PREOP_HB PREOP_WCC PREOP_NA PREOP_K
## 2485              1        1         1        1       1    0
## 11                1        1         1        1       0    1
## 7                 1        1         1        0       0    2
## 6                 1        0         0        1       1    2
## 576               1        0         0        0       0    4
##                   0      582       582      583     594 2341
```

```
aggr(master, col=c("white", "red"), numbers = TRUE, sortVars=TRUE
          , cex.axis=.7, gap=3, ylab=c("Percentage of missing data", "Missing data Pattern"))
```

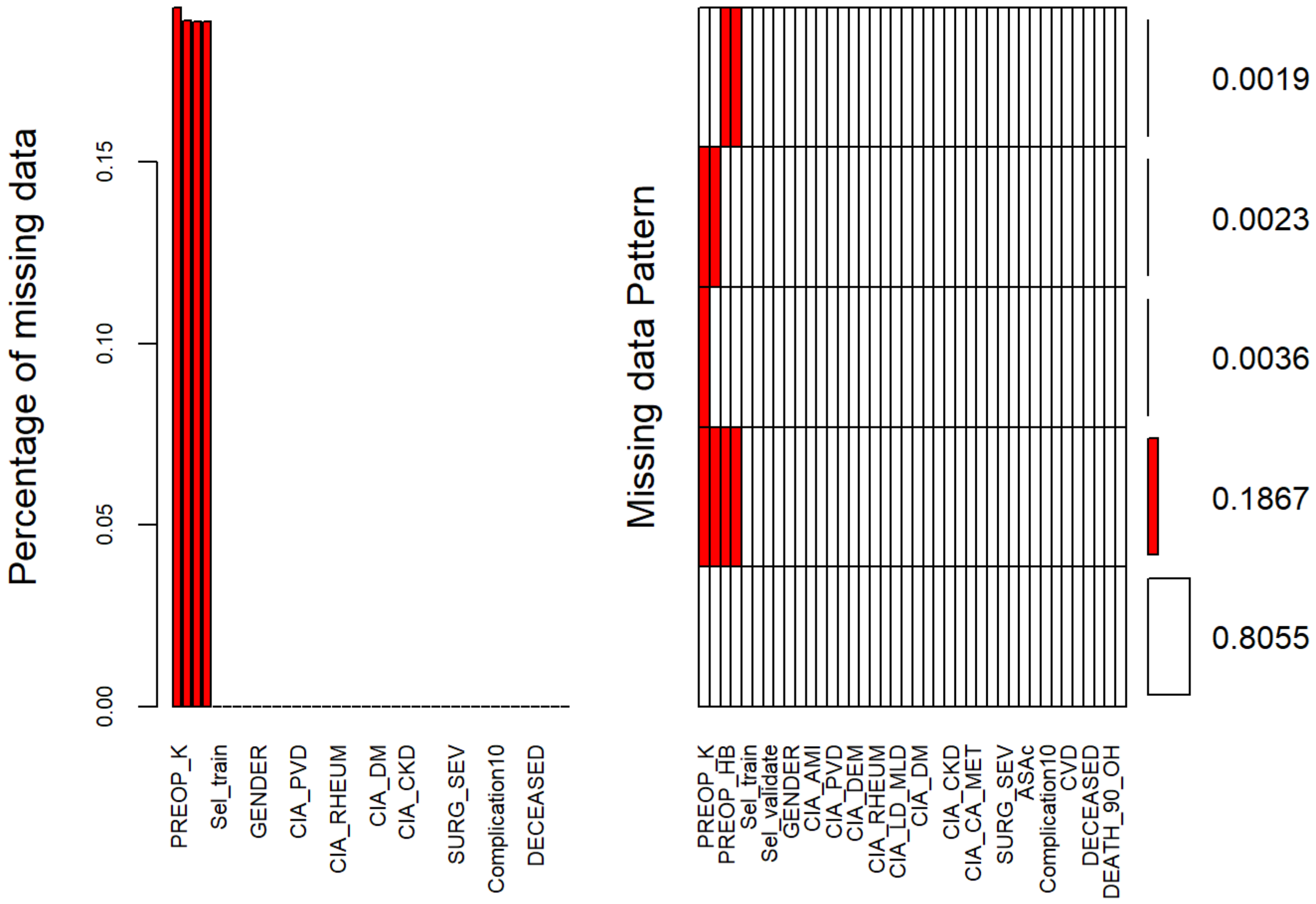

Percentage of missing data
0.00
0.05
0.10
0.15
PREOP_K
Sel_train
GENDER
CIA_PVD
CIA_RHEUM
CIA_DM
CIA_CKD
SURG_SEV
Complication10
DECEASED
Missing data Pattern
PREOP_K
PREOP_HB
Sel_train
Sel_validate
GENDER
CIA_AMI
CIA_PVD
CIA_DEM
CIA_RHEUM
CIA_LD_MLD
CIA_DM
CIA_CKD
CIA_CA_MET
SURG_SEV
ASAc
Complication10
CVD
DECEASED
DEATH_90_OH
0.0019
0.0023
0.0036
0.1867
0.8055

```
##
##  Variables sorted by number of missings:
##             Variable     Count
##              PREOP_K 0.1925446
##             PREOP_NA 0.1889789
##             PREOP_HB 0.1886548
##            PREOP_WCC 0.1886548
##            Sel_train 0.0000000
##             Sel_test 0.0000000
##         Sel_validate 0.0000000
##           Patient.No 0.0000000
##               GENDER 0.0000000
##                  AGE 0.0000000
##              CIA_AMI 0.0000000
##              CIA_CHF 0.0000000
##              CIA_PVD 0.0000000
##             CIA_CEVD 0.0000000
##              CIA_DEM 0.0000000
##             CIA_COPD 0.0000000
##            CIA_RHEUM 0.0000000
##              CIA_PUD 0.0000000
##           CIA_LD_MLD 0.0000000
##           CIA_LD_SEV 0.0000000
##               CIA_DM 0.0000000
##            CIA_DM_CX 0.0000000
##       CIA_HEMIPLAEGIA 0.0000000
##              CIA_CKD 0.0000000
##               CIA_CA 0.0000000
##           CIA_CA_MET 0.0000000
##              CIA_HIV 0.0000000
##  HOSPITAL_ADMIT_TYPE 0.0000000
##             SURG_SEV 0.0000000
##  SURG_SCHEDULED_TYPE 0.0000000
##                 ASAc 0.0000000
##             PreopICU 0.0000000
##        Complication10 0.0000000
##                 NoCx 0.0000000
##                  CVD 0.0000000
##               CVD_23 0.0000000
##             DECEASED 0.0000000
##     DEATH_INHOSPITAL 0.0000000
##          DEATH_90_OH 0.0000000
##       ACCI_corrected 0.0000000
```

Missing arises mainly in preoperative lab findings.

5.2 Missing data evaluation

Little’s MCAR test. Nonsignificant P value indicates possible MAR, MCAR

```
library(naniar)
```

```
dt.select <- master[,c("AGE","PREOP_HB","PREOP_WCC","PREOP_NA","PREOP_K")]
mcar_test(data=dt.select)
```

```
## # A tibble: 1 × 4
##   statistic    df p.value missing.patterns
##       <dbl> <dbl>   <dbl>            <int>
## 1      19.5    11  0.0523                5
```

```
dt.select <- master[,c("SURG_SCHEDULED_TYPE","PREOP_HB","PREOP_WCC","PREOP_NA","PREOP_K")]
mcar_test(data=dt.select)
```

```
## # A tibble: 1 × 4
##   statistic    df p.value missing.patterns
##       <dbl> <dbl>   <dbl>            <int>
## 1      200.    11       0                5
```

```
dt.select <- master[,c("SURG_SEV","PREOP_HB","PREOP_WCC","PREOP_NA","PREOP_K")]
mcar_test(data=dt.select)
```

```
## # A tibble: 1 × 4
##   statistic    df p.value missing.patterns
##       <dbl> <dbl>   <dbl>            <int>
## 1      142.    11       0                5
```

```
dt.select <- master[,c("CVD_23","PREOP_HB","PREOP_WCC","PREOP_NA","PREOP_K")]
mcar_test(data=dt.select)
```

```
## # A tibble: 1 × 4
##   statistic    df  p.value missing.patterns
##       <dbl> <dbl>    <dbl>            <int>
## 1      71.1    11 7.39e-11                5
```

```
dt.select <- master[, c("CVD_23", "PREOP_HB","PREOP_WCC","PREOP_NA","PREOP_K")]
mcar_test(data=dt.select)
```

```
## # A tibble: 1 × 4
##   statistic    df  p.value missing.patterns
##       <dbl> <dbl>    <dbl>            <int>
## 1      71.1    11 7.39e-11                5
```

```
dt.select <- master[, c("DEATH_INHOSPITAL", "PREOP_HB","PREOP_WCC","PREOP_NA","PREOP_K")]
mcar_test(data=dt.select)
```

```
## # A tibble: 1 × 4
##   statistic    df p.value missing.patterns
##       <dbl> <dbl>   <dbl>            <int>
## 1      16.5    11   0.122                5
```

```
dt.select <- master[, c("DEATH_90_OH", "PREOP_HB","PREOP_WCC","PREOP_NA","PREOP_K")]
mcar_test(data=dt.select)
```

```
## # A tibble: 1 × 4
##   statistic    df  p.value missing.patterns
##       <dbl> <dbl>    <dbl>            <int>
## 1      32.2    11 0.000705                5
```

Missing patterns of preoperative laboratory findings are suspected non-ramdom missing,
look like to relate with elective, low risk surgery

```
gg_miss_upset(master)
```

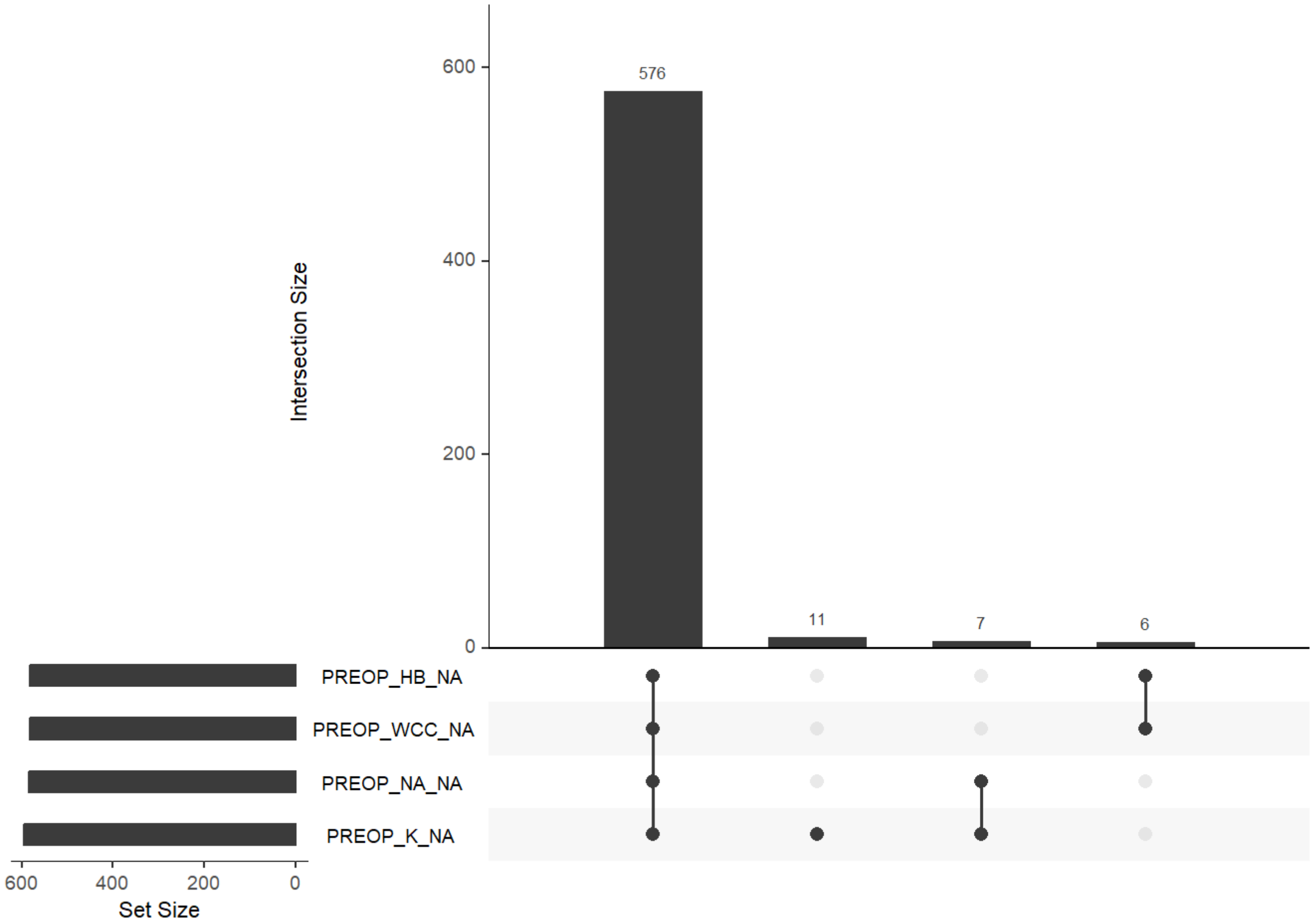


```
gg_miss_fct(x = master, fct = SURG_SCHEDULED_TYPE)
```

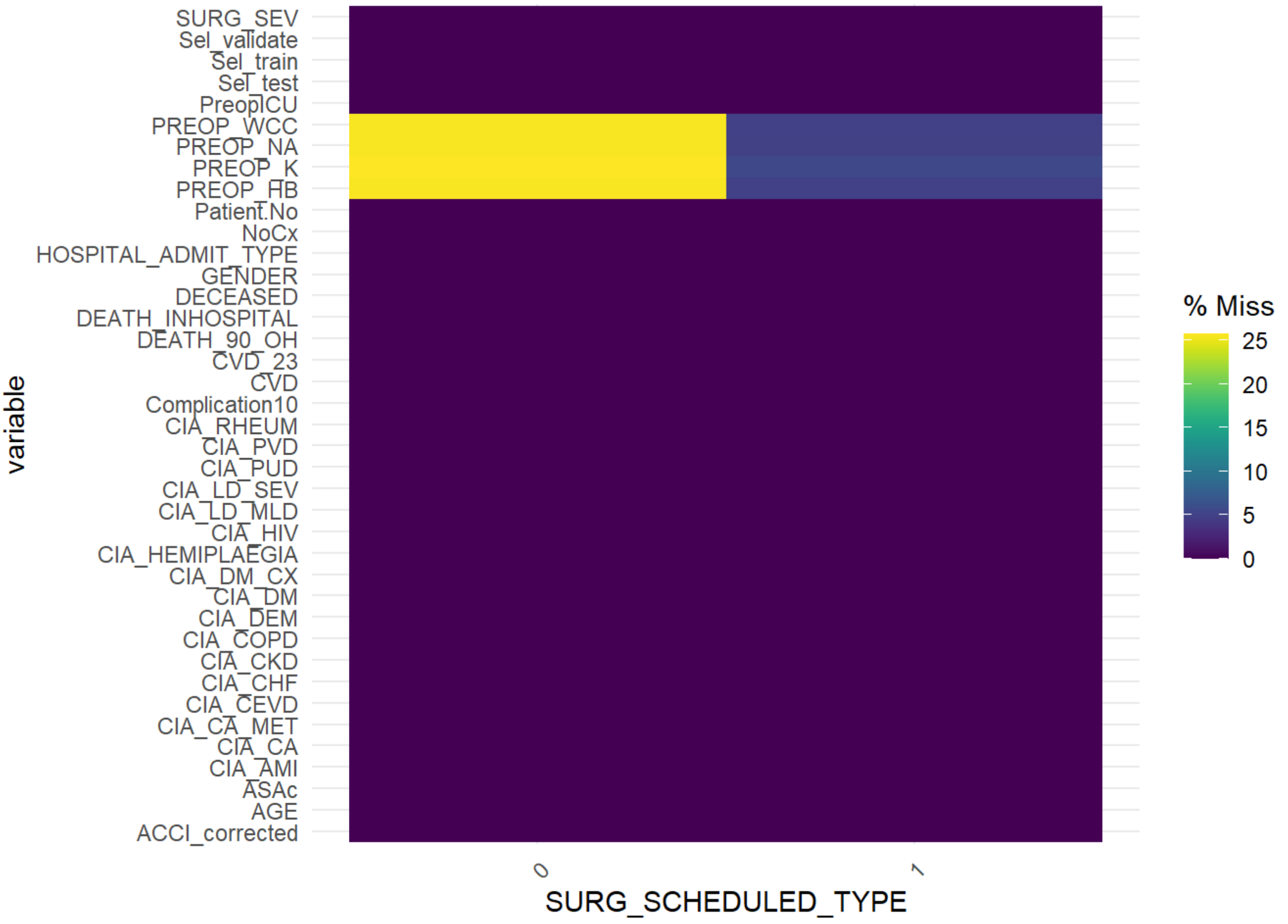


```
gg_miss_fct(x = master, fct = SURG_SEV)
```

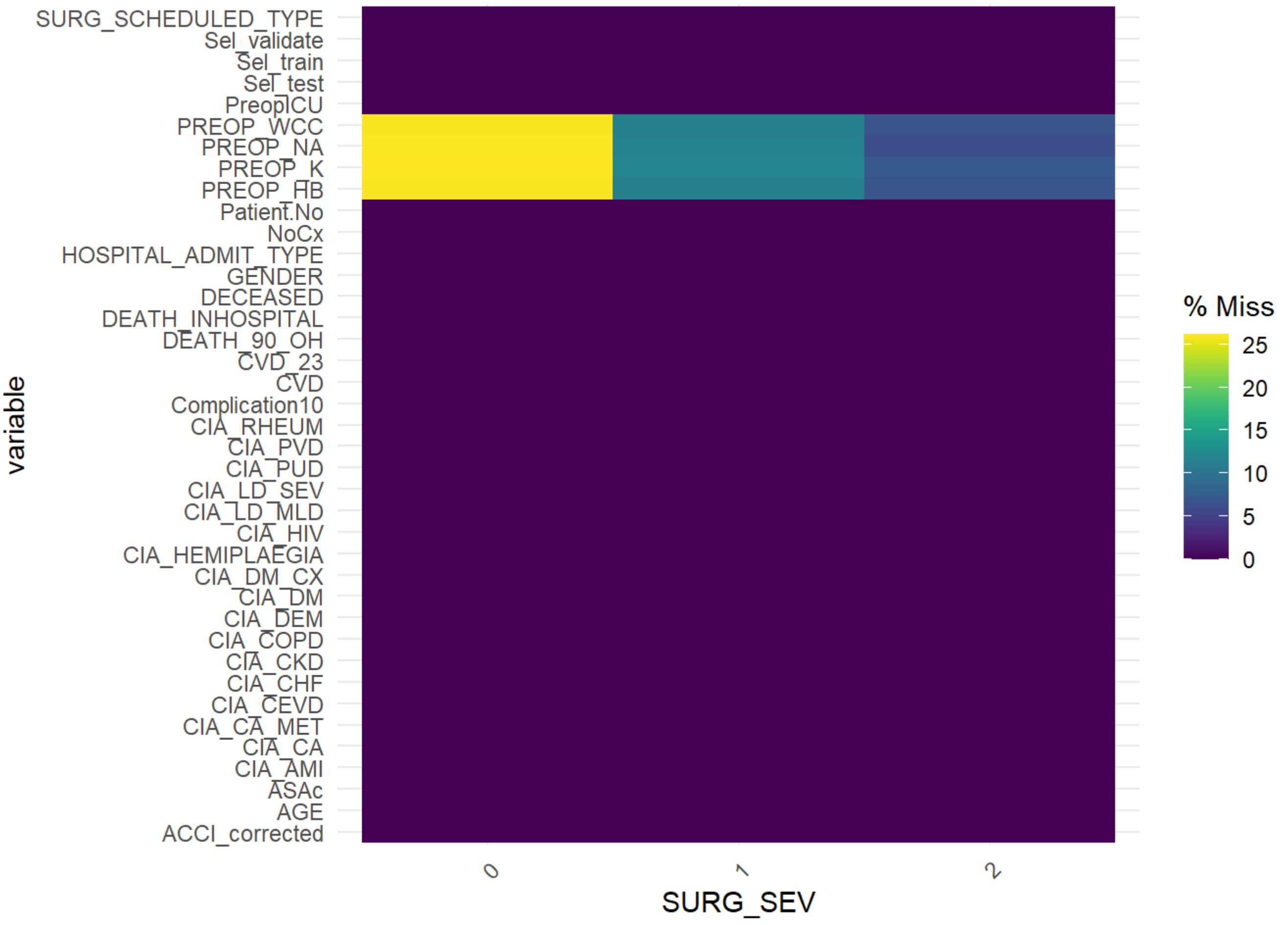


```
gg_miss_fct(x = master, fct = CVD_23)
```

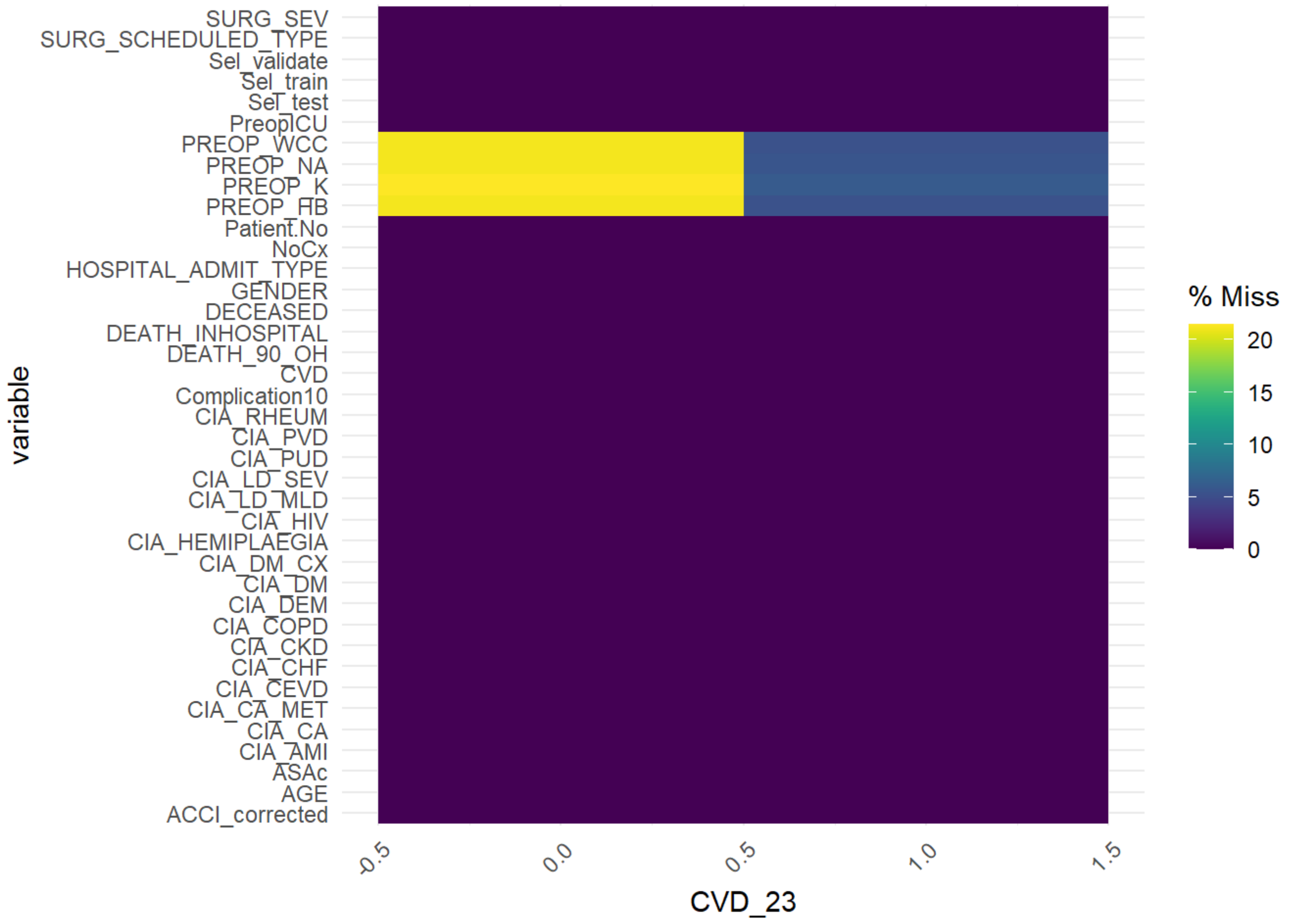


```
gg_miss_fct(x = master, fct = DEATH_INHOSPITAL)
```

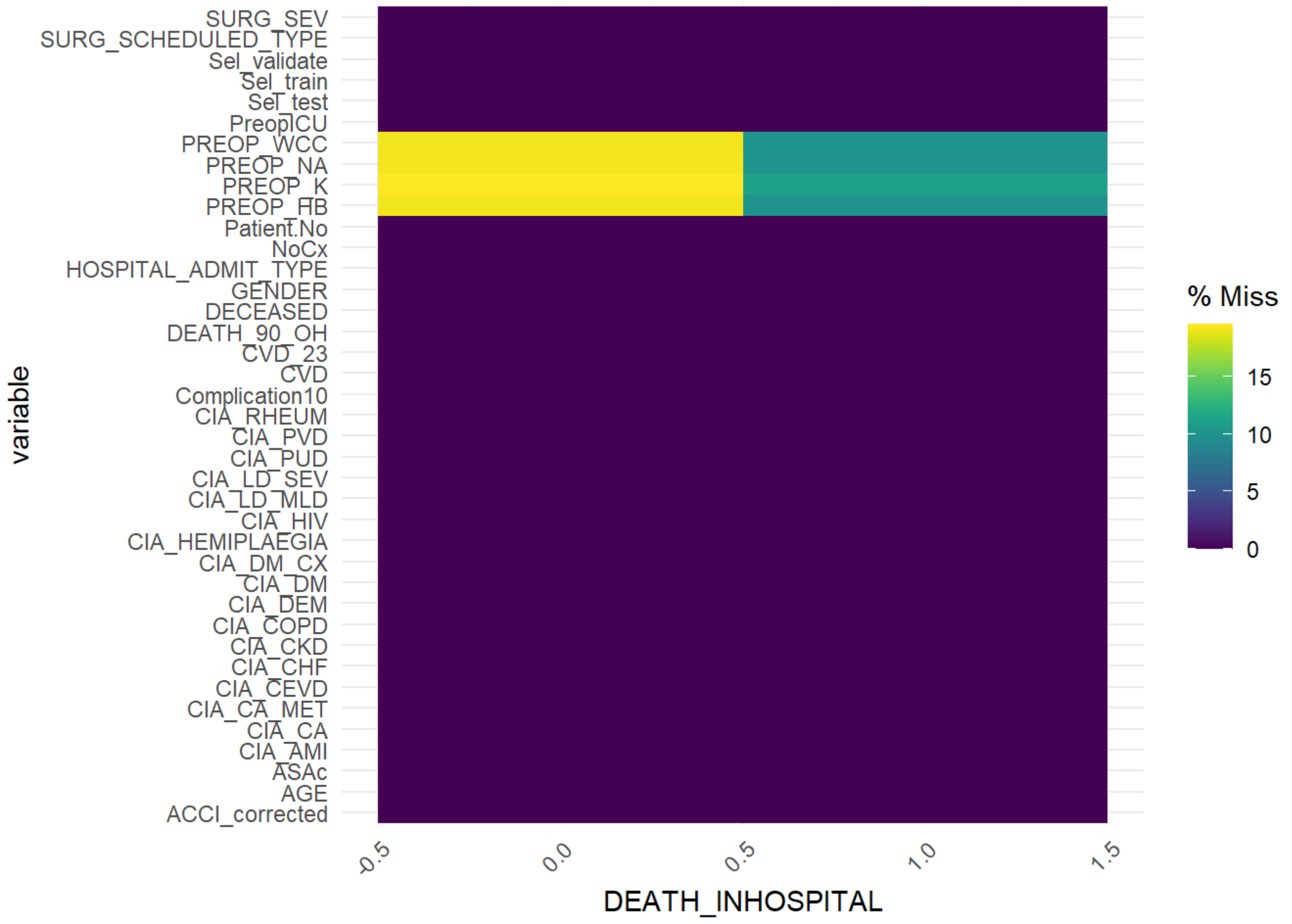


```
gg_miss_fct(x = master, fct = DEATH_90_OH)
```

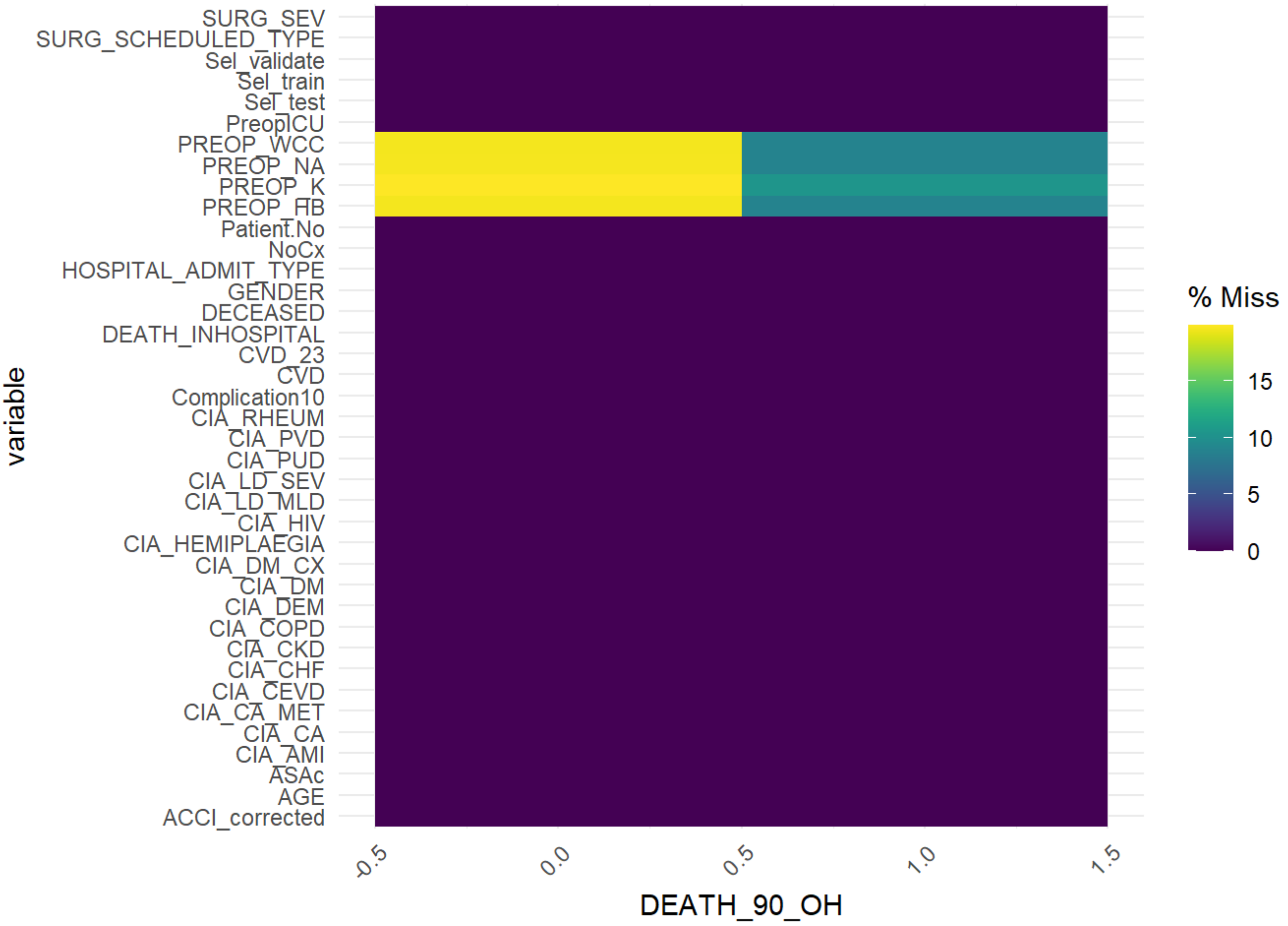


```
# Check the missing rate
master$missing<-ifelse(apply(master, 1, anyNA), 1, 0)

missing.schedule <-table(master$SURG_SCHEDULED_TYPE, master$missing) %>%
  addmargins()

missing.schedule
```

```
##
##          0    1  Sum
##   0   1546  544 2090
##   1    939   56  995
##   Sum 2485  600 3085
```

```
# missing rate of elective surgery
missing.schedule[1,2]/(missing.schedule[1,1]+missing.schedule[1,2])
```

```
## [1] 0.2602871
```

```
# missing rate of emergency surgery
missing.schedule[2,2]/(missing.schedule[2,1]+missing.schedule[2,2])
```

```
## [1] 0.05628141
```

```
missing.risk <- table(master$SURG_SEV, master$missing) %>%
  addmargins()

missing.risk
```

```
##
##          0    1  Sum
##   0   1233  441 1674
##   1    986  135 1121
##   2    266   24  290
##   Sum 2485  600 3085
```

```
# missing rate of low-risk surgery
missing.risk[1,2]/(missing.risk[1,1] + missing.risk[1,2])
```

```
## [1] 0.2634409
```

```
# missing rate of moderate-risk surgery
missing.risk[2,2]/(missing.risk[2,1]+missing.risk[2,2])
```

```
## [1] 0.1204282
```

```
# missing rate of high-risk surgery
missing.risk[3,2]/(missing.risk[3,1]+missing.risk[3,2])
```

```
## [1] 0.08275862
```

As confirmed by the above graphs, missing occurs in relation with surgery types, severity. That is, missing pattern is not “Missing At Random,” or “Missing Completely At Random.” Thus, we make a plan to perform the detailed sensitivity analysis between original and missing imputed datasets at the end of model estimation. Also, we adopt the multiple imputation method rather than single imputation method to reduce the bias generation during imputation process.

5.3 Missing imputation

As planned, multiple imputation is performed on the training dataset.

```
# Multiple imputation: 5 imputed datasets, 10 iterations, the predictive mean matching method
# Output suppressed
train.imp<- mice(train.org, m=5, maxit=10, method = "pmm", seed = 13)
```

```
# Complete imputed dataset
train.impall <- complete(train.imp, action = "long", include = TRUE)

# each imputed dataset
train.imp1 <- complete(train.imp, action = 1)
train.imp2 <- complete(train.imp, action = 2)
train.imp3 <- complete(train.imp, action = 3)
train.imp4 <- complete(train.imp, action = 4)
train.imp5 <- complete(train.imp, action = 5)

# Compare summaries of original and imputed dataset
summary(train.org)
```

```
##  Sel_train Sel_test Sel_validate   Patient.No     GENDER       AGE
##  0:   0    0:1968   0:1968       Min.   :   3.0   0: 954   Min.   : 90.00
##  1:1968    1:   0   1:   0       1st Qu.: 775.2   1:1014   1st Qu.: 90.00
##                                  Median :1553.5            Median : 92.00
##                                  Mean   :1557.6            Mean   : 92.24
##                                  3rd Qu.:2345.0            3rd Qu.: 93.00
##                                  Max.   :3103.0            Max.   :105.00
##
##  CIA_AMI  CIA_CHF  CIA_PVD  CIA_CEVD CIA_DEM  CIA_COPD CIA_RHEUM CIA_PUD
##  0:1959   0:1899   0:1924   0:1928   0:1884   0:1946   0:1966    0:1945
##  1:   9   1:  69   1:  44   1:  40   1:  84   1:  22   1:   2    1:  23
##
##
##
##
##
##  CIA_LD_MLD CIA_LD_SEV CIA_DM   CIA_DM_CX CIA_HEMIPLAEGIA CIA_CKD  CIA_CA
##  0:1962     0:1965     0:1829   0:1806    0:1949          0:1851   0:1797
##  1:   6     1:   3     1: 139   1: 162    1:  19          1: 117   1: 171
##
##
##
##
##
##  CIA_CA_MET CIA_HIV  HOSPITAL_ADMIT_TYPE    PREOP_HB       PREOP_WCC
##  0:1934     0:1968   0: 666              Min.   : 4.30   Min.   : 1.400
##  1:  34              1:1301              1st Qu.:10.70   1st Qu.: 6.100
##                      2:   1              Median :12.10   Median : 7.600
##                                          Mean   :11.94   Mean   : 8.412
##                                          3rd Qu.:13.40   3rd Qu.: 9.600
##                                          Max.   :18.20   Max.   :82.500
##                                          NA's   :382     NA's   :382
##     PREOP_NA        PREOP_K      SURG_SEV SURG_SCHEDULED_TYPE ASAc     PreopICU
##  Min.   :120.0   Min.   :2.800   0:1076   0:1323              0:  15   0:1953
##  1st Qu.:138.0   1st Qu.:4.200   1: 703   1: 645              1:1898   1:  15
##  Median :140.0   Median :4.500   2: 189                       2:  55
##  Mean   :139.5   Mean   :4.479
##  3rd Qu.:142.0   3rd Qu.:4.800
##  Max.   :155.0   Max.   :6.400
##  NA's   :384     NA's   :393
##  Complication10       NoCx             CVD             CVD_23
##  Min.   :0.0000   Min.   : 0.000   Min.   :0.0000   Min.   :0.0000
##  1st Qu.:0.0000   1st Qu.: 0.000   1st Qu.:0.0000   1st Qu.:0.0000
##  Median :0.0000   Median : 0.000   Median :0.0000   Median :0.0000
##  Mean   :0.3288   Mean   : 1.234   Mean   :0.9243   Mean   :0.1504
##  3rd Qu.:1.0000   3rd Qu.: 1.000   3rd Qu.:2.0000   3rd Qu.:0.0000
##  Max.   :1.0000   Max.   :25.000   Max.   :5.0000   Max.   :1.0000
##
##     DECEASED      DEATH_INHOSPITAL   DEATH_90_OH      ACCI_corrected
##  Min.   :0.0000   Min.   :0.00000   Min.   :0.00000   Min.   : 5.000
##  1st Qu.:0.0000   1st Qu.:0.00000   1st Qu.:0.00000   1st Qu.: 5.000
##  Median :0.0000   Median :0.00000   Median :0.00000   Median : 5.000
##  Mean   :0.2322   Mean   :0.03557   Mean   :0.06656   Mean   : 5.818
##  3rd Qu.:0.0000   3rd Qu.:0.00000   3rd Qu.:0.00000   3rd Qu.: 6.000
##  Max.   :1.0000   Max.   :1.00000   Max.   :1.00000   Max.   :14.000
##
```

```
summary (complete(train.imp))
```

```
##  Sel_train Sel_test Sel_validate   Patient.No     GENDER       AGE
##  0:   0    0:1968   0:1968       Min.   :   3.0   0: 954   Min.   : 90.00
##  1:1968    1:   0   1:   0       1st Qu.: 775.2   1:1014   1st Qu.: 90.00
##                                   Median :1553.5           Median : 92.00
##                                   Mean   :1557.6           Mean   : 92.24
##                                   3rd Qu.:2345.0           3rd Qu.: 93.00
##                                   Max.   :3103.0           Max.   :105.00
##  CIA_AMI  CIA_CHF  CIA_PVD  CIA_CEVD CIA_DEM  CIA_COPD CIA_RHEUM CIA_PUD
##  0:1959   0:1899   0:1924   0:1928   0:1884   0:1946   0:1966    0:1945
##  1:   9   1:  69   1:  44   1:  40   1:  84   1:  22   1:   2    1:  23
##
##
##
##
##  CIA_LD_MLD CIA_LD_SEV CIA_DM   CIA_DM_CX CIA_HEMIPLAEGIA CIA_CKD  CIA_CA
##  0:1962     0:1965     0:1829   0:1806    0:1949          0:1851   0:1797
##  1:   6     1:   3     1: 139   1: 162    1:  19          1: 117   1: 171
##
##
##
##
##  CIA_CA_MET CIA_HIV  HOSPITAL_ADMIT_TYPE    PREOP_HB       PREOP_WCC
##  0:1934     0:1968   0: 666              Min.   : 4.30   Min.   : 1.400
##  1:  34              1:1301              1st Qu.:10.80   1st Qu.: 6.000
##                      2:   1              Median :12.20   Median : 7.500
##                                          Mean   :12.07   Mean   : 8.263
##                                          3rd Qu.:13.50   3rd Qu.: 9.500
##                                          Max.   :18.20   Max.   :82.500
##     PREOP_NA        PREOP_K      SURG_SEV SURG_SCHEDULED_TYPE ASAc     PreopICU
##  Min.   :120.0   Min.   :2.800   0:1076   0:1323              0:  15   0:1953
##  1st Qu.:138.0   1st Qu.:4.200   1: 703   1: 645              1:1898   1:  15
##  Median :140.0   Median :4.500   2: 189                       2:  55
##  Mean   :139.6   Mean   :4.491
##  3rd Qu.:142.0   3rd Qu.:4.800
##  Max.   :155.0   Max.   :6.400
##  Complication10       NoCx             CVD             CVD_23
##  Min.   :0.0000   Min.   : 0.000   Min.   :0.0000   Min.   :0.0000
##  1st Qu.:0.0000   1st Qu.: 0.000   1st Qu.:0.0000   1st Qu.:0.0000
##  Median :0.0000   Median : 0.000   Median :0.0000   Median :0.0000
##  Mean   :0.3288   Mean   : 1.234   Mean   :0.9243   Mean   :0.1504
##  3rd Qu.:1.0000   3rd Qu.: 1.000   3rd Qu.:2.0000   3rd Qu.:0.0000
##  Max.   :1.0000   Max.   :25.000   Max.   :5.0000   Max.   :1.0000
##     DECEASED      DEATH_INHOSPITAL   DEATH_90_OH      ACCI_corrected
##  Min.   :0.0000   Min.   :0.00000   Min.   :0.00000   Min.   : 5.000
##  1st Qu.:0.0000   1st Qu.:0.00000   1st Qu.:0.00000   1st Qu.: 5.000
##  Median :0.0000   Median :0.00000   Median :0.00000   Median : 5.000
##  Mean   :0.2322   Mean   :0.03557   Mean   :0.06656   Mean   : 5.818
##  3rd Qu.:0.0000   3rd Qu.:0.00000   3rd Qu.:0.00000   3rd Qu.: 6.000
##  Max.   :1.0000   Max.   :1.00000   Max.   :1.00000   Max.   :14.000
```

5.4 Checking the convergence of imputed dataset

```
plot(train.imp)
```

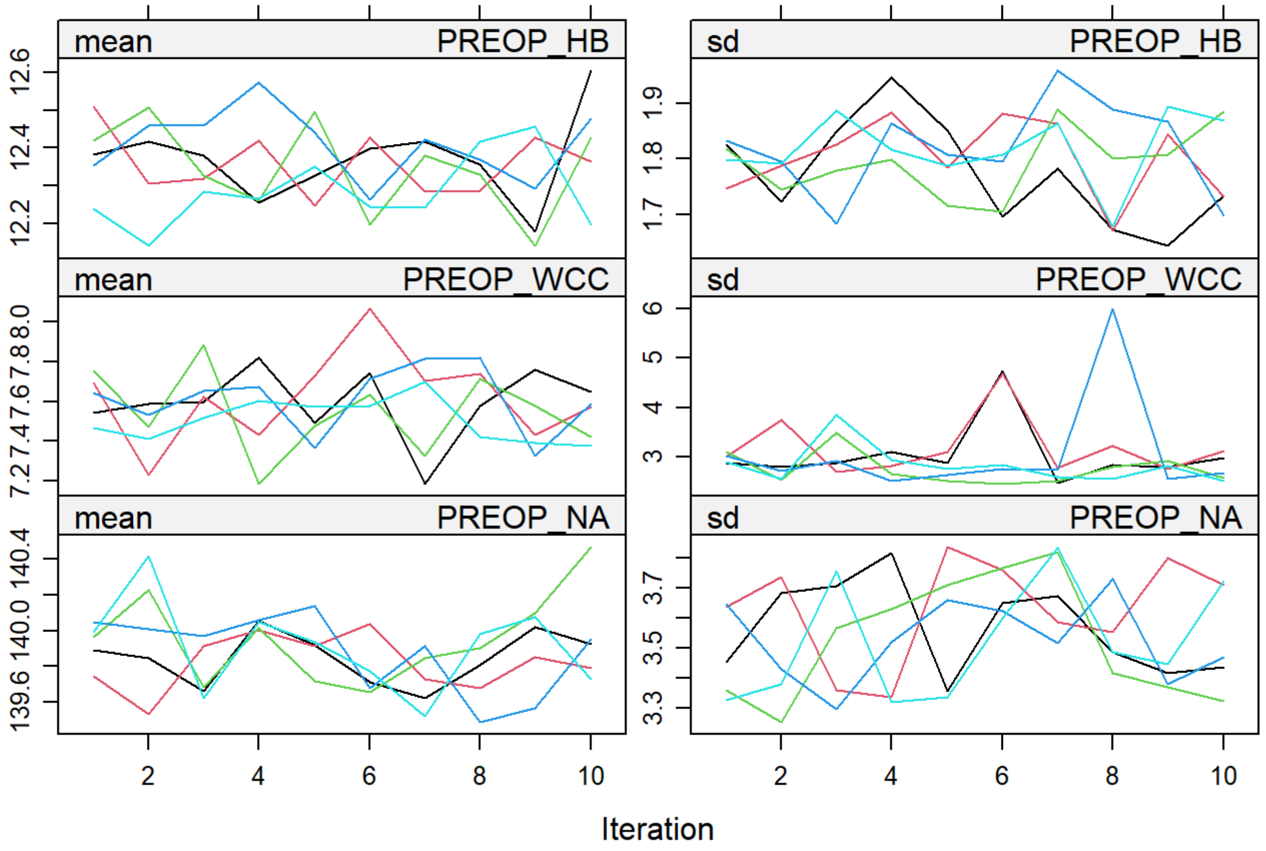

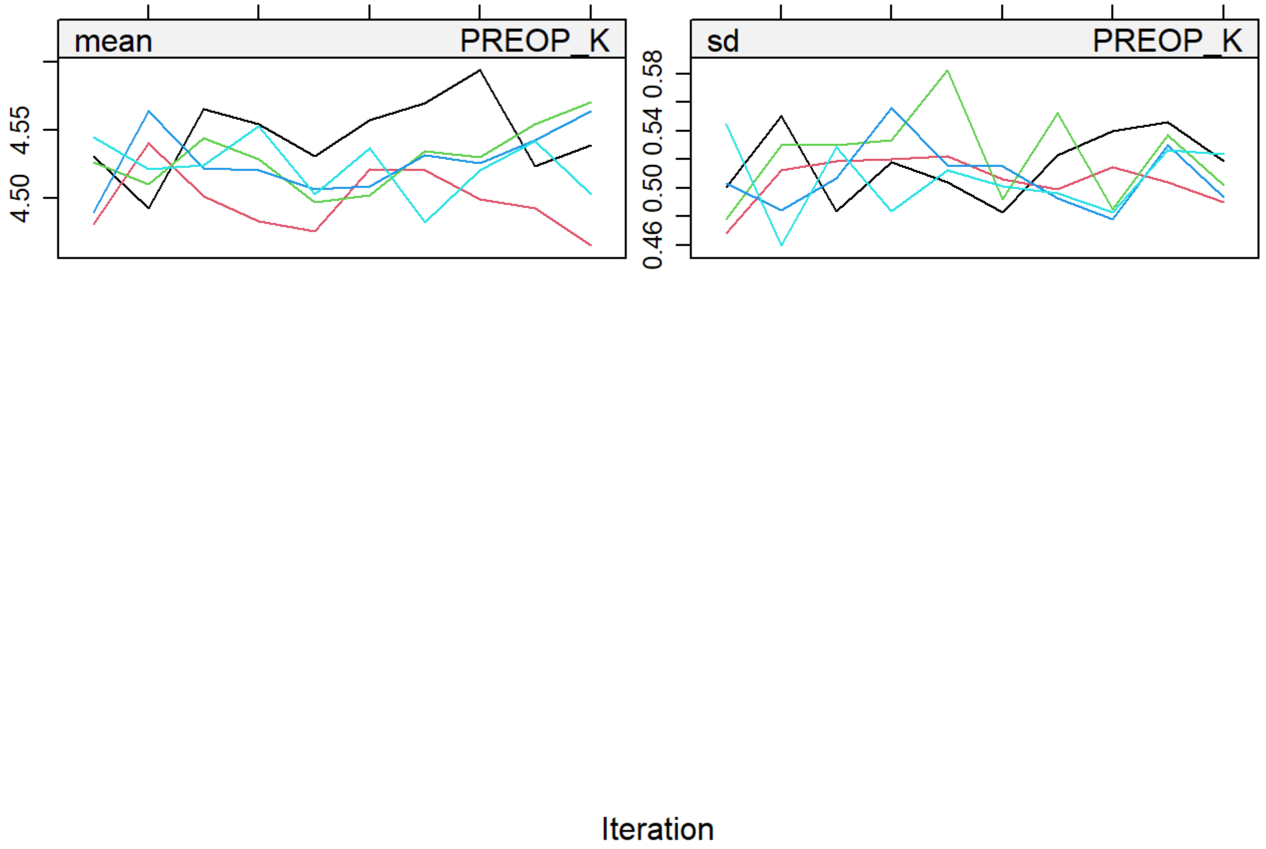

The variance between the imputation chains is almost equal to the variance within the chains, which indicates healthy convergence.

5.5 Imputation diagnostics

```
# Density plot
densityplot(train.imp)
```

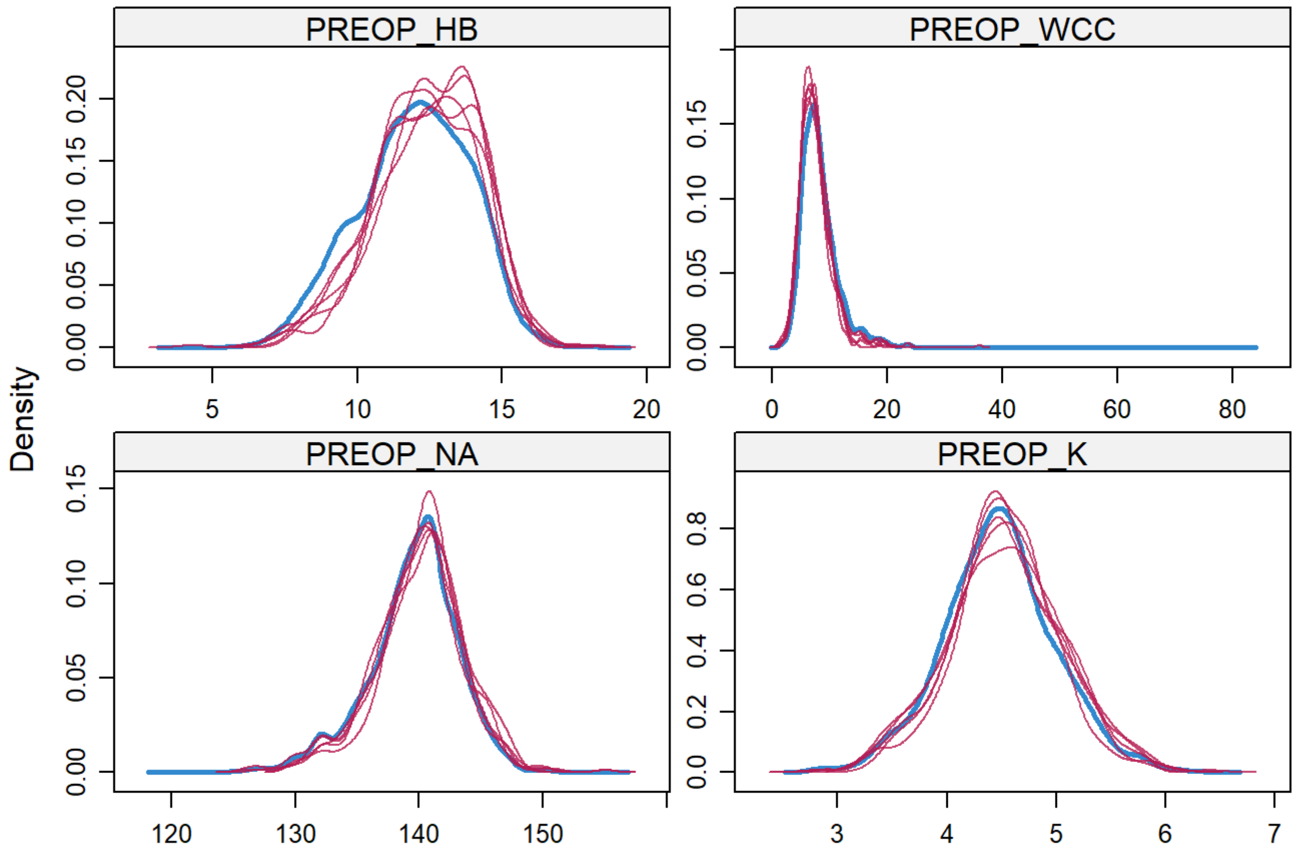


```
# Strip plot
stripplot(train.imp, pch = 20, cex = 1.2)[3]
```

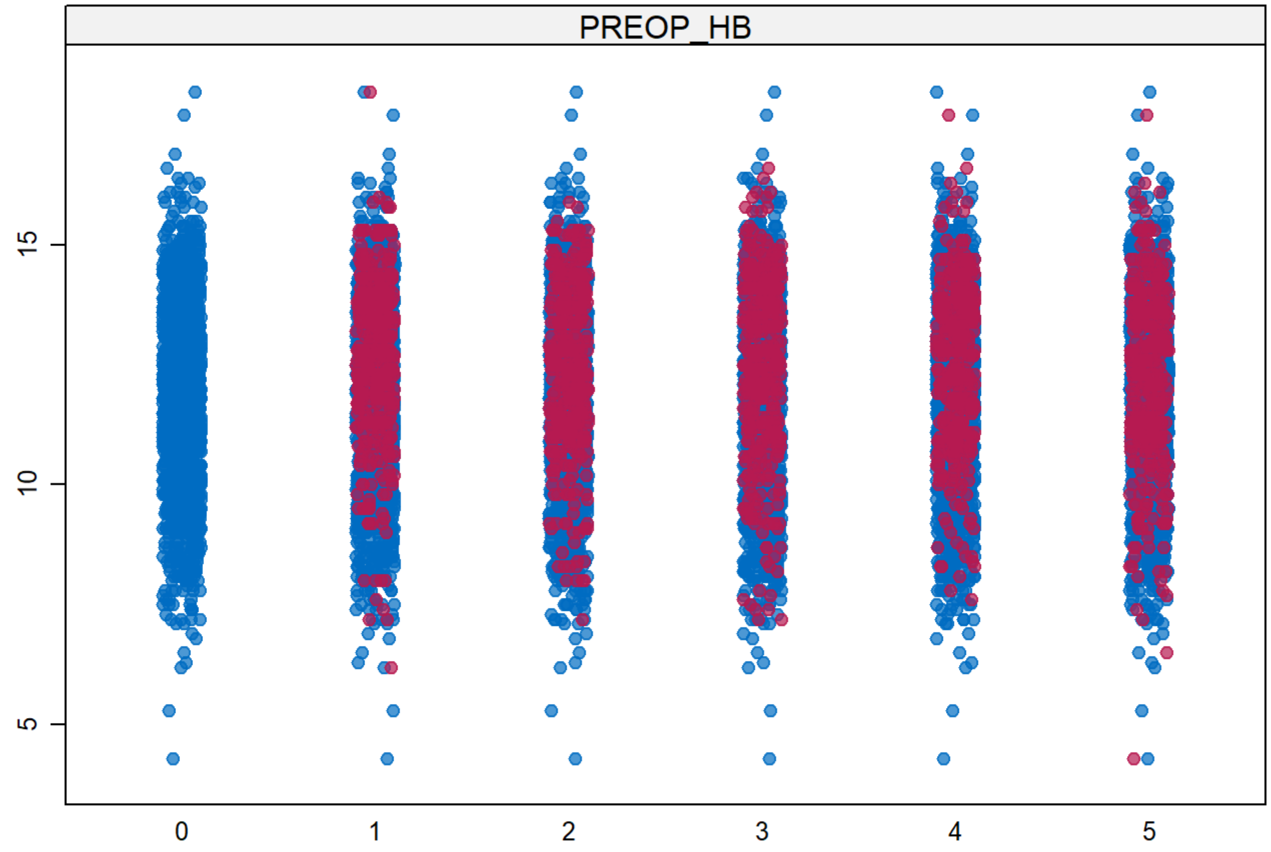


```
stripplot(train.imp, pch = 20, cex = 1.2)[4]
```

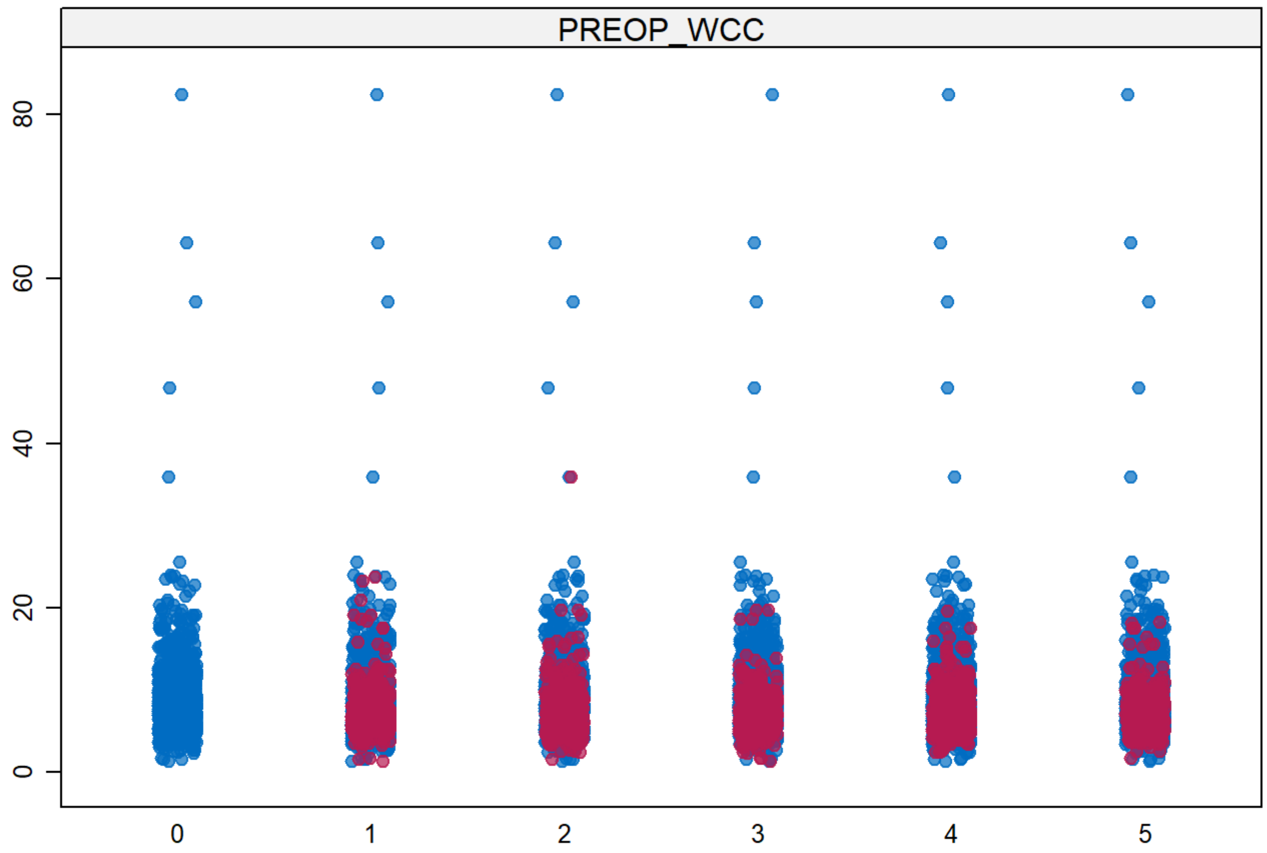


```
stripplot(train.imp, pch = 20, cex = 1.2)[5]
```

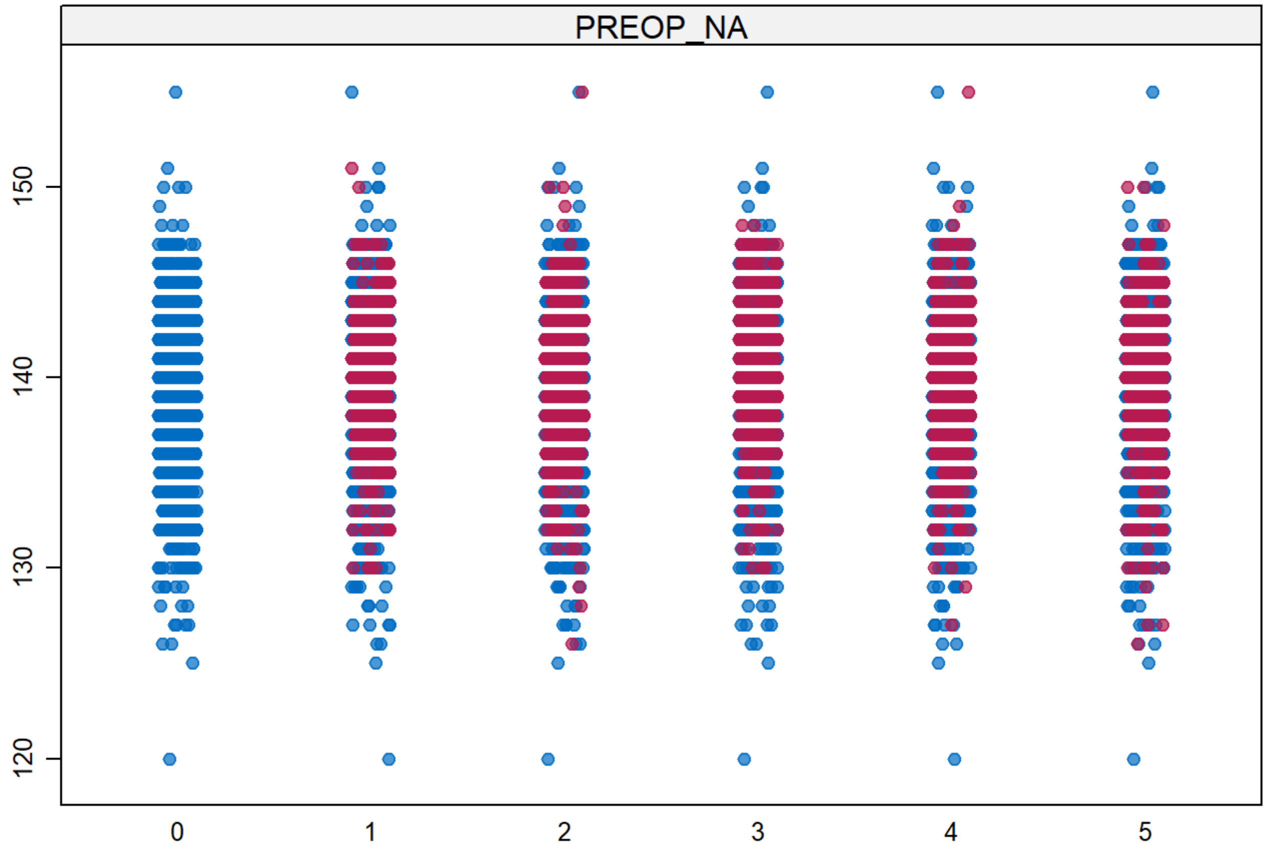


```
stripplot(train.imp, pch = 20, cex = 1.2)[6]
```

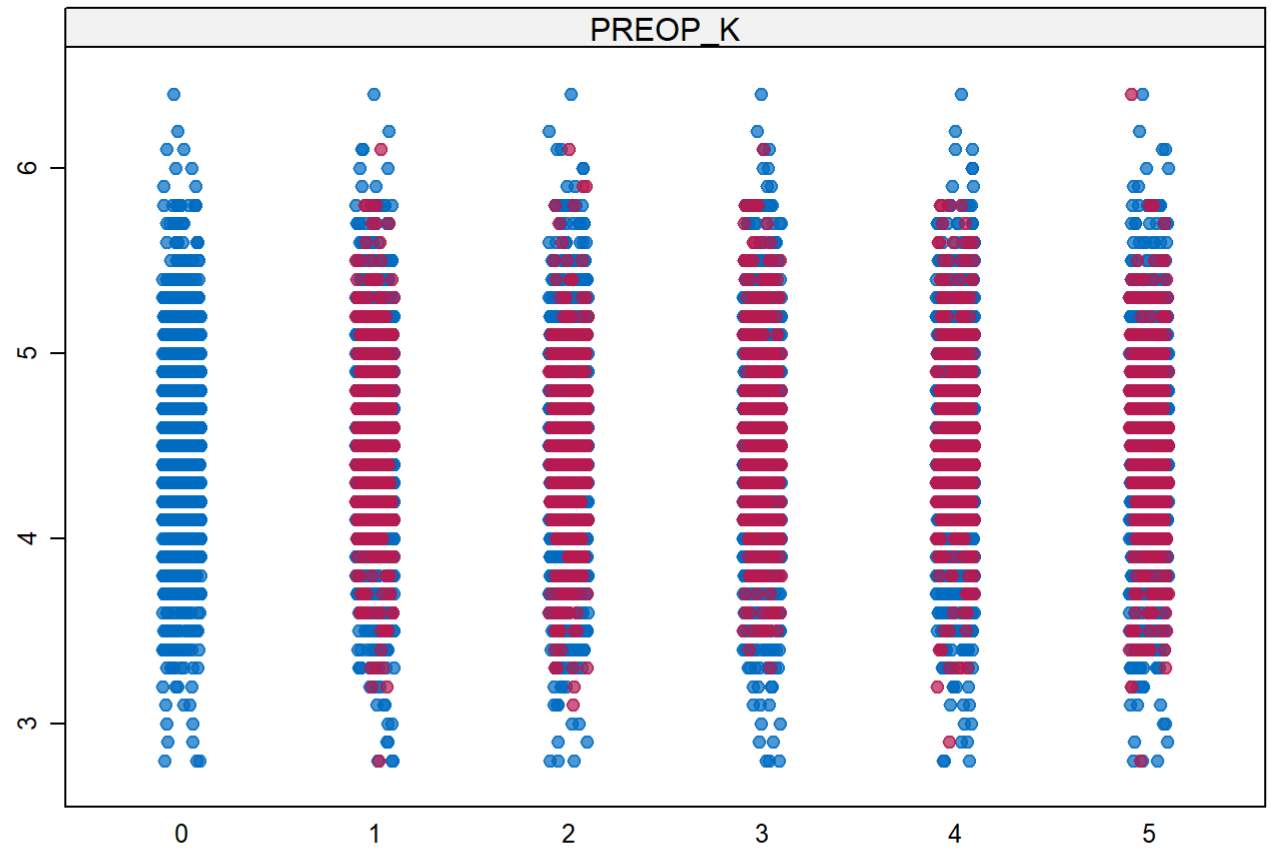

Comparing the observed and the imputed data points, the imputed values are in range of the observed data. There are no large difference between the imputed and observed values, we can conclude the imputed values are plausible.

5.5.1 Function definitions for calibration calculation

Because the package “rms” dose not provide the calibrations: Hosmer-Lemeshow statistics C & H,
it is required the following custom functions to calculate HL-C & HL-H.

Reference: Yingxiang Huang, Wentao Li, Fima Macheret, Rodney A Gabriel, Lucila Ohno-Machado, A tutorial on calibration measurements and calibration models for clinical prediction models, Journal of the American Medical Informatics Association, Volume 27, Issue 4, April 2020, Pages 621–633, https://doi.org/10.1093/jamia/ocz228 (https://doi.org/10.1093/jamia/ocz228)

```
library(reshape)
library(ggplot2)
library(rms)
```

5.6 Data prepration for the sensitivity analysis

We generate imputations under the delta-adjustment. To get an adjusted imputation model, we add a fixed amount (delta) from the imputed values. Adjustment is applied to the preoprative haemoglobin (PREOP_HB). Because imputed PREOP_HB is used to imput other missing values, delta will also affect the imputations in those.
We set the delta adjustment as 0 (assuming missing at random), and other two plausible values that assume a MNAR situation; one SD higher than the mean of imputed PREOP_HB (small effect), two SD higher than the mean of imputed PREOP_HB (large effect).

Reference: Van Buuren, Stef. Flexible imputation of missing data. CRC press, 2018.

```
ini <- mice(train.org, maxit = 0)
imp.data <- complete(train.imp, "long")
m <- mean(means <- with(imp.data, tapply(PREOP_HB, .imp, mean)))
var <- mean(variances <- with(imp.data, tapply(PREOP_HB, .imp, var)))

post <- ini$post

delta <- c(0, m+sqrt(var), m+2*sqrt(var))
train.imp.delta <- vector("list", length(delta))

for (i in 1:length(delta)) {
  d <- delta[i]
  cmd <- paste("imp[[j]][,i] <- imp[[j]][,i] +", d)
  post["PREOP_HB"] <- cmd
  imp <- mice(train.org, post = post, maxit = 10,
            seed = 13)
train.imp.delta[[i]] <- imp
}
```

6 Risk prediction model development: Presence of any complications

6.1 Original data analysis

Original data analysis includes complete cases only.

```
# Remove cases with NA values
train.org.na <- na.omit(train.org)

# Logistic regression model: Enter all input parameters
lranycx.org.enter <- glm(Complication10 ~ GENDER
                                         + CIA_AMI
                                         + CIA_CHF
                                         + CIA_PVD
                                         + CIA_CEVD
                                         + CIA_DEM
                                         + CIA_COPD
                                         + CIA_PUD
                                         + CIA_LD_MLD
                                         + CIA_LD_SEV
                                         + CIA_DM
                                         + CIA_DM_CX
                                         + CIA_HEMIPLAEGIA
                                         + CIA_CKD
                                         + CIA_CA
                                         + CIA_CA_MET
                                         + PREOP_HB
                                         + PREOP_WCC
                                         + PREOP_NA
                                         + PREOP_K
                                         + SURG_SEV
                                         + SURG_SCHEDULED_TYPE
                                         + PreopICU
            , data =  train.org.na, family = binomial, na.action = "na.omit")


summary(lranycx.org.enter)
```

```
##
## Call:
## glm(formula = Complication10 ~ GENDER + CIA_AMI + CIA_CHF + CIA_PVD +
##     CIA_CEVD + CIA_DEM + CIA_COPD + CIA_PUD + CIA_LD_MLD + CIA_LD_SEV +
##     CIA_DM + CIA_DM_CX + CIA_HEMIPLAEGIA + CIA_CKD + CIA_CA +
##     CIA_CA_MET + PREOP_HB + PREOP_WCC + PREOP_NA + PREOP_K +
##     SURG_SEV + SURG_SCHEDULED_TYPE + PreopICU, family = binomial,
##     data = train.org.na, na.action = "na.omit")
##
## Deviance Residuals:
##     Min       1Q   Median       3Q      Max
## -2.7506  -0.6489  -0.4189   0.6745   2.5162
##
## Coefficients:
##                       Estimate Std. Error z value Pr(>|z|)
## (Intercept)           0.489818   2.706105   0.181 0.856364
## GENDER1              -0.191456   0.136533  -1.402 0.160835
## CIA_AMI1             -0.544975   0.889427  -0.613 0.540058
## CIA_CHF1              2.463749   0.513524   4.798 1.60e-06 ***
## CIA_PVD1              1.473469   0.421462   3.496 0.000472 ***
## CIA_CEVD1             0.434595   0.599906   0.724 0.468796
## CIA_DEM1              1.185394   0.340749   3.479 0.000504 ***
## CIA_COPD1             1.054906   0.664766   1.587 0.112539
## CIA_PUD1              0.036982   0.541473   0.068 0.945547
## CIA_LD_MLD1           0.219622   0.928246   0.237 0.812968
## CIA_LD_SEV1          -0.179637   1.359231  -0.132 0.894857
## CIA_DM1               0.395657   0.240549   1.645 0.100009
## CIA_DM_CX1            0.657760   0.234313   2.807 0.004998 **
## CIA_HEMIPLAEGIA1      1.288793   1.157246   1.114 0.265420
## CIA_CKD1              1.119090   0.301746   3.709 0.000208 ***
## CIA_CA1               0.523868   0.222052   2.359 0.018314 *
## CIA_CA_MET1           1.189442   0.419297   2.837 0.004557 **
## PREOP_HB             -0.146342   0.036556  -4.003 6.25e-05 ***
## PREOP_WCC             0.046918   0.018150   2.585 0.009738 **
## PREOP_NA             -0.004665   0.018311  -0.255 0.798912
## PREOP_K              -0.122849   0.133685  -0.919 0.358127
## SURG_SEV1             0.921318   0.143554   6.418 1.38e-10 ***
## SURG_SEV2             2.170106   0.252135   8.607  < 2e-16 ***
## SURG_SCHEDULED_TYPE1  1.374906   0.147478   9.323  < 2e-16 ***
## PreopICU1             0.721331   0.822306   0.877 0.380375
## ---
## Signif. codes:  0 '***' 0.001 '**' 0.01 '*' 0.05 '.' 0.1 ' ' 1
##
## (Dispersion parameter for binomial family taken to be 1)
##
##     Null deviance: 2099.7  on 1570  degrees of freedom
## Residual deviance: 1427.4  on 1546  degrees of freedom
## AIC: 1477.4
##
## Number of Fisher Scoring iterations: 5
```

```
# Stepwise selection method: AIC guided.
# Suppressed output
lranycx.org.sel <- step(lranycx.org.enter)
```

```
summary(lranycx.org.sel)
```

```
##
## Call:
## glm(formula = Complication10 ~ CIA_CHF + CIA_PVD + CIA_DEM +
##     CIA_COPD + CIA_DM + CIA_DM_CX + CIA_HEMIPLAEGIA + CIA_CKD +
##     CIA_CA + CIA_CA_MET + PREOP_HB + PREOP_WCC + SURG_SEV + SURG_SCHEDULED_TYPE,
##     family = binomial, data = train.org.na, na.action = "na.omit")
##
## Deviance Residuals:
##     Min       1Q   Median       3Q      Max
## -2.7565  -0.6452  -0.4221   0.6567   2.4505
##
## Coefficients:
##                      Estimate Std. Error z value Pr(>|z|)
## (Intercept)          -0.75134    0.45621  -1.647 0.099573 .
## CIA_CHF1              2.48280    0.50665   4.900 9.56e-07 ***
## CIA_PVD1              1.41781    0.41722   3.398 0.000678 ***
## CIA_DEM1              1.19307    0.33994   3.510 0.000449 ***
## CIA_COPD1             1.02583    0.66218   1.549 0.121344
## CIA_DM1               0.36621    0.23845   1.536 0.124580
## CIA_DM_CX1            0.57610    0.22999   2.505 0.012250 *
## CIA_HEMIPLAEGIA1      1.67413    1.08364   1.545 0.122367
## CIA_CKD1              1.12669    0.30128   3.740 0.000184 ***
## CIA_CA1               0.52686    0.21993   2.396 0.016595 *
## CIA_CA_MET1           1.20600    0.41920   2.877 0.004016 **
## PREOP_HB             -0.15176    0.03586  -4.232 2.31e-05 ***
## PREOP_WCC             0.04692    0.01795   2.614 0.008938 **
## SURG_SEV1             0.92906    0.14098   6.590 4.40e-11 ***
## SURG_SEV2             2.22977    0.24681   9.034  < 2e-16 ***
## SURG_SCHEDULED_TYPE1  1.42900    0.14438   9.898  < 2e-16 ***
## ---
## Signif. codes:  0 '***' 0.001 '**' 0.01 '*' 0.05 '.' 0.1 ' ' 1
##
## (Dispersion parameter for binomial family taken to be 1)
##
##     Null deviance: 2099.7  on 1570  degrees of freedom
## Residual deviance: 1432.0  on 1555  degrees of freedom
## AIC: 1464
##
## Number of Fisher Scoring iterations: 5
```

```
# Final logistic regression model with the selected input parameters
lranycx.org.final <- glm(Complication10 ~ CIA_CHF
                                         + CIA_PVD
                                         + CIA_DEM
                                         + CIA_COPD
                                         + CIA_DM
                                         + CIA_DM_CX
                                         + CIA_HEMIPLAEGIA
                                         + CIA_CKD
                                         + CIA_CA
                                         + CIA_CA_MET
                                         + PREOP_HB
                                         + PREOP_WCC
                                         + SURG_SEV
                                         + SURG_SCHEDULED_TYPE
                         , data =  train.org.na
                         , family = binomial, na.action = "na.omit")

summary(lranycx.org.final)
```

```
##
## Call:
## glm(formula = Complication10 ~ CIA_CHF + CIA_PVD + CIA_DEM +
##     CIA_COPD + CIA_DM + CIA_DM_CX + CIA_HEMIPLAEGIA + CIA_CKD +
##     CIA_CA + CIA_CA_MET + PREOP_HB + PREOP_WCC + SURG_SEV + SURG_SCHEDULED_TYPE,
##     family = binomial, data = train.org.na, na.action = "na.omit")
##
## Deviance Residuals:
##     Min       1Q   Median       3Q      Max
## -2.7565  -0.6452  -0.4221   0.6567   2.4505
##
## Coefficients:
##                      Estimate Std. Error z value Pr(>|z|)
## (Intercept)          -0.75134    0.45621  -1.647 0.099573 .
## CIA_CHF1              2.48280    0.50665   4.900 9.56e-07 ***
## CIA_PVD1              1.41781    0.41722   3.398 0.000678 ***
## CIA_DEM1              1.19307    0.33994   3.510 0.000449 ***
## CIA_COPD1             1.02583    0.66218   1.549 0.121344
## CIA_DM1               0.36621    0.23845   1.536 0.124580
## CIA_DM_CX1            0.57610    0.22999   2.505 0.012250 *
## CIA_HEMIPLAEGIA1      1.67413    1.08364   1.545 0.122367
## CIA_CKD1              1.12669    0.30128   3.740 0.000184 ***
## CIA_CA1               0.52686    0.21993   2.396 0.016595 *
## CIA_CA_MET1           1.20600    0.41920   2.877 0.004016 **
## PREOP_HB             -0.15176    0.03586  -4.232 2.31e-05 ***
## PREOP_WCC             0.04692    0.01795   2.614 0.008938 **
## SURG_SEV1             0.92906    0.14098   6.590 4.40e-11 ***
## SURG_SEV2             2.22977    0.24681   9.034  < 2e-16 ***
## SURG_SCHEDULED_TYPE1  1.42900    0.14438   9.898  < 2e-16 ***
## ---
## Signif. codes:  0 '***' 0.001 '**' 0.01 '*' 0.05 '.' 0.1 ' ' 1
##
## (Dispersion parameter for binomial family taken to be 1)
##
##     Null deviance: 2099.7  on 1570  degrees of freedom
## Residual deviance: 1432.0  on 1555  degrees of freedom
## AIC: 1464
##
## Number of Fisher Scoring iterations: 5
```

```
# OR and 95%CI
exp(cbind(OR = coef(lranycx.org.final), confint(lranycx.org.final)))
```

```
##                             OR     2.5 %      97.5 %
## (Intercept)           0.4717331 0.1917425   1.1481044
## CIA_CHF1             11.9746947 4.8314993  36.5238634
## CIA_PVD1              4.1280575 1.8882782   9.8434136
## CIA_DEM1              3.2971955 1.7349949   6.6204445
## CIA_COPD1             2.7894019 0.8181410  11.5320041
## CIA_DM1               1.4422612 0.8990227   2.2927131
## CIA_DM_CX1            1.7790890 1.1332700   2.7950333
## CIA_HEMIPLAEGIA1      5.3341535 0.9328066 101.4649844
## CIA_CKD1              3.0854392 1.7346539   5.6721521
## CIA_CA1               1.6936035 1.0936882   2.5942095
## CIA_CA_MET1           3.3400914 1.4949750   7.8307433
## PREOP_HB              0.8591959 0.8005547   0.9214706
## PREOP_WCC             1.0480349 1.0136966   1.0872439
## SURG_SEV1             2.5321194 1.9228265   3.3428265
## SURG_SEV2             9.2977449 5.7932321  15.2773342
## SURG_SCHEDULED_TYPE1  4.1745179 3.1477499   5.5453318
```

```
# ANOVA for individual terms: requires library "car"
car::Anova(lranycx.org.final, type = "II", test = "Wald")
```

```
## Analysis of Deviance Table (Type II tests)
##
## Response: Complication10
##                     Df   Chisq Pr(>Chisq)
## CIA_CHF              1 24.0140  9.564e-07 ***
## CIA_PVD              1 11.5478  0.0006783 ***
## CIA_DEM              1 12.3175  0.0004487 ***
## CIA_COPD             1  2.3999  0.1213438
## CIA_DM               1  2.3588  0.1245799
## CIA_DM_CX            1  6.2743  0.0122501 *
## CIA_HEMIPLAEGIA      1  2.3868  0.1223665
## CIA_CKD              1 13.9852  0.0001843 ***
## CIA_CA               1  5.7387  0.0165954 *
## CIA_CA_MET           1  8.2765  0.0040161 **
## PREOP_HB             1 17.9137  2.312e-05 ***
## PREOP_WCC            1  6.8353  0.0089375 **
## SURG_SEV             2 96.3236  < 2.2e-16 ***
## SURG_SCHEDULED_TYPE  1 97.9658  < 2.2e-16 ***
## ---
## Signif. codes:  0 '***' 0.001 '**' 0.01 '*' 0.05 '.' 0.1 ' ' 1
```

```
# Pseudo R sqaured: requires library "rcompanion"
rcompanion::nagelkerke(lranycx.org.final)
```

```
## $Models
##
## Model: "glm, Complication10 ~ CIA_CHF + CIA_PVD + CIA_DEM + CIA_COPD + CIA_DM + CIA_DM_CX + CIA_H
EMIPLAEGIA + CIA_CKD + CIA_CA + CIA_CA_MET + PREOP_HB + PREOP_WCC + SURG_SEV + SURG_SCHEDULED_TYPE,
binomial, train.org.na, na.omit"
## Null:  "glm, Complication10 ~ 1, binomial, train.org.na, na.omit"
##
## $Pseudo.R.squared.for.model.vs.null
##                              Pseudo.R.squared
## McFadden                             0.318005
## Cox and Snell (ML)                   0.346245
## Nagelkerke (Cragg and Uhler)         0.469648
##
## $Likelihood.ratio.test
##  Df.diff LogLik.diff  Chisq     p.value
##      -15     -333.86 667.71 1.4063e-132
##
## $Number.of.observations
##
## Model: 1571
## Null:  1571
##
## $Messages
## [1] "Note: For models fit with REML, these statistics are based on refitting with ML"
##
## $Warnings
## [1] "None"
```

```
# Overall p value for model
anova(lranycx.org.final
    , update(lranycx.org.final, ~1) # update here produces null model fo comparison
    , test = "Chisq")
```

```
## Analysis of Deviance Table
##
## Model 1: Complication10 ~ CIA_CHF + CIA_PVD + CIA_DEM + CIA_COPD + CIA_DM +
##     CIA_DM_CX + CIA_HEMIPLAEGIA + CIA_CKD + CIA_CA + CIA_CA_MET +
##     PREOP_HB + PREOP_WCC + SURG_SEV + SURG_SCHEDULED_TYPE
## Model 2: Complication10 ~ 1
##   Resid. Df Resid. Dev  Df Deviance  Pr(>Chi)
## 1      1555     1432.0
## 2      1570     2099.7 -15  -667.71 < 2.2e-16 ***
## ---
## Signif. codes:  0 '***' 0.001 '**' 0.01 '*' 0.05 '.' 0.1 ' ' 1
```

```
# Standardized residuals
plot(fitted(lranycx.org.final)
     , rstandard(lranycx.org.final))
```

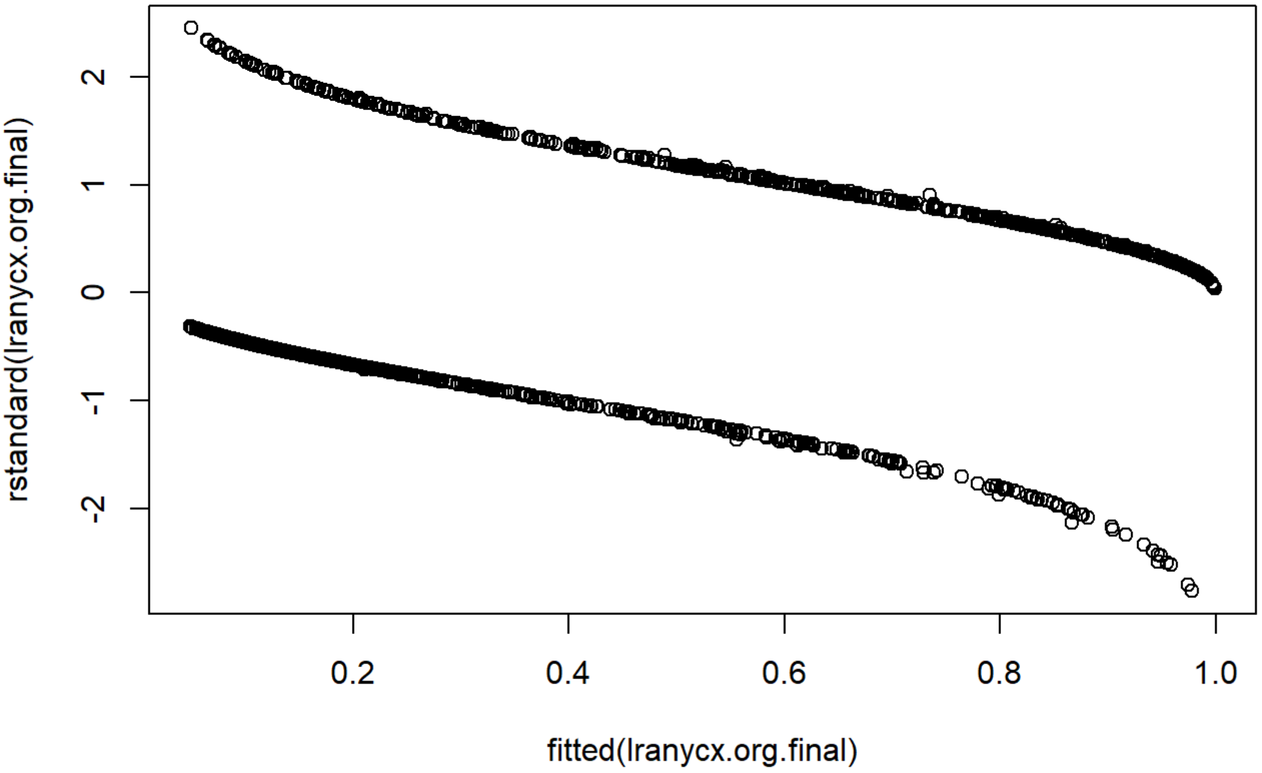


```
plot(lranycx.org.final)
```

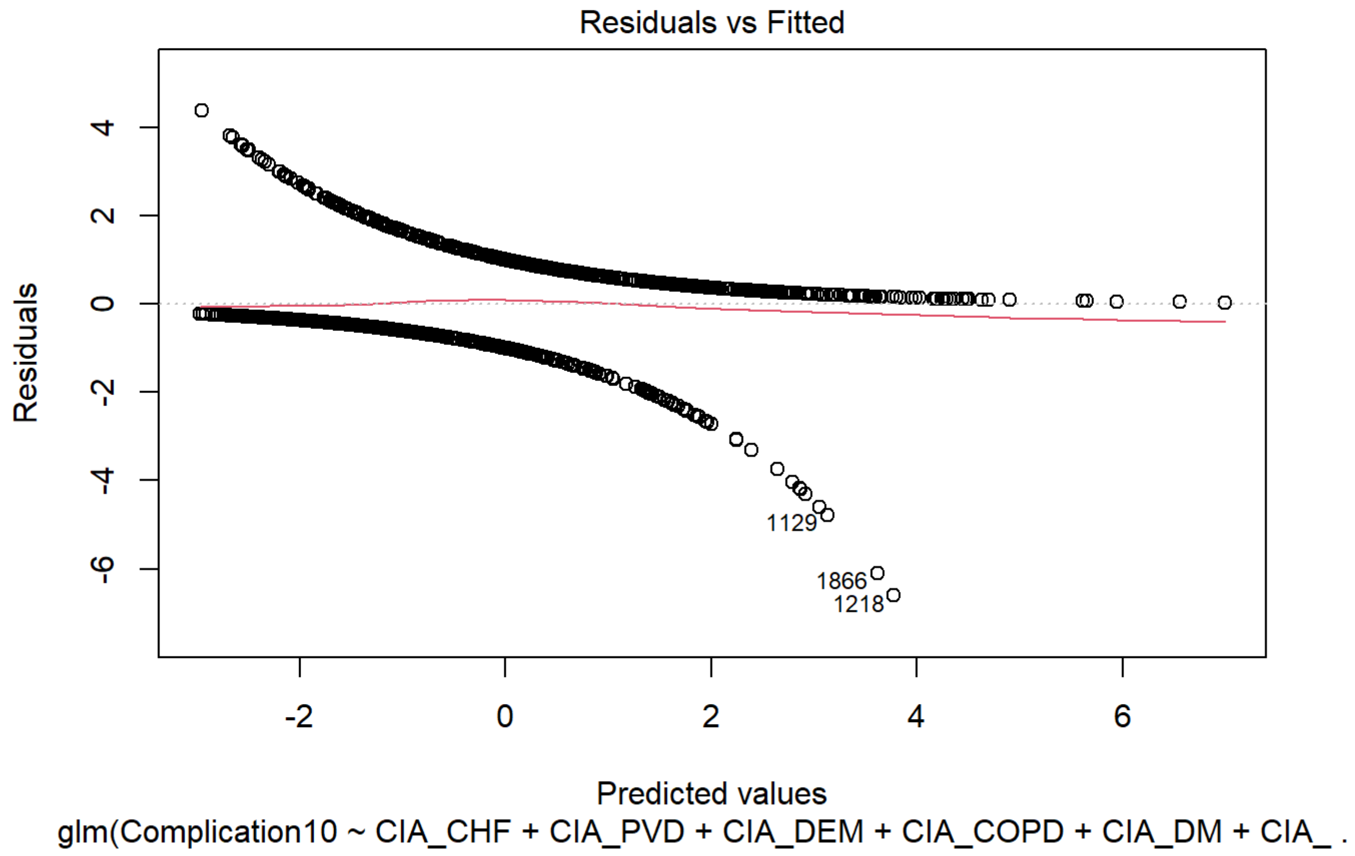
Residuals vs Fitted
Residuals
4
2
0
-2
-4
-6
1129
1866
1218
-2
0
2
4
6
Predicted values
glm(Complication10 ~ CIA_CHF + CIA_PVD + CIA_DEM + CIA_COPD + CIA_DM + CIA_ .

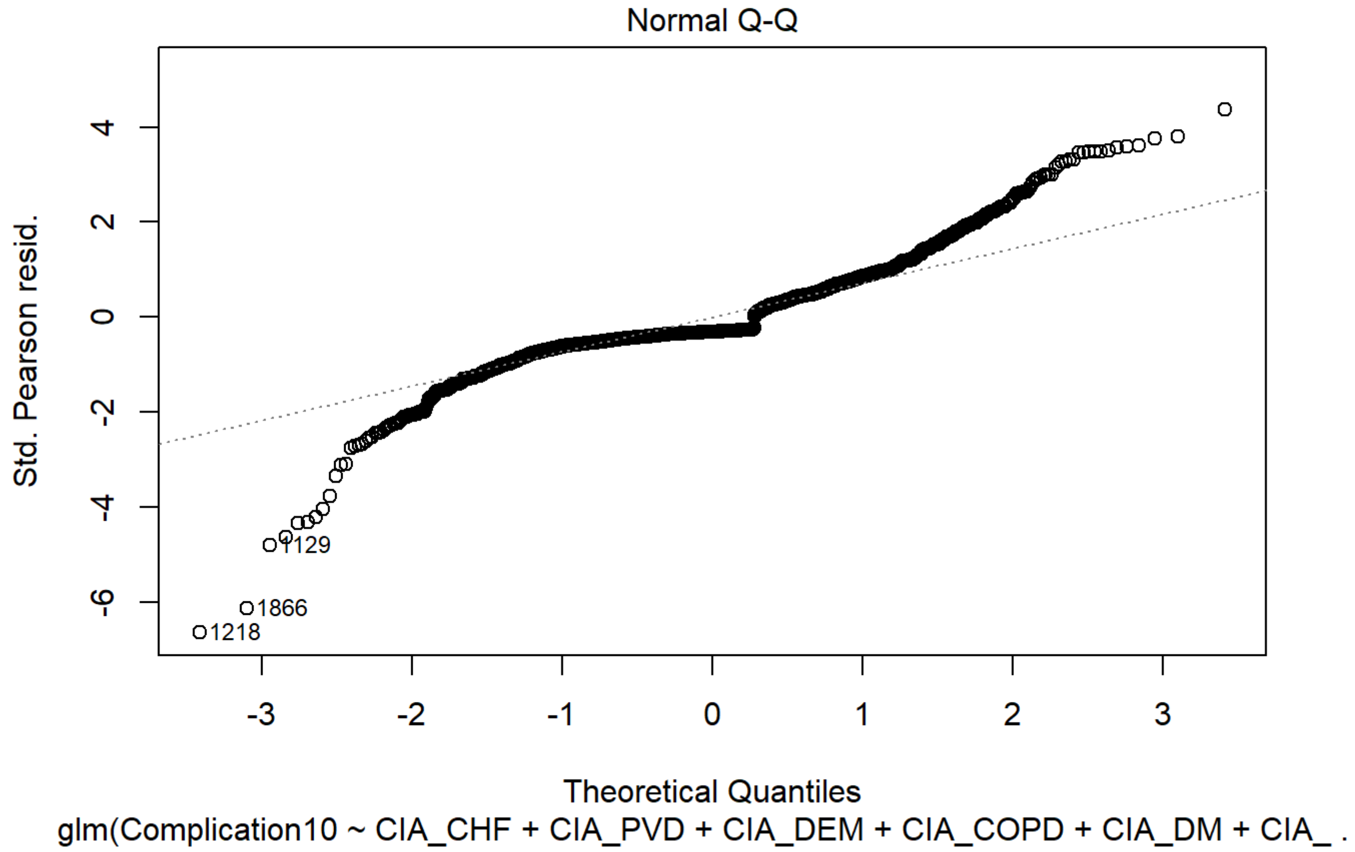
Normal Q-Q
Std. Pearson resid.
4
2
0
-2
-4
-6
1129
1866
1218
-3
-2
-1
0
1
2
3
Theoretical Quantiles
glm(Complication10 ~ CIA_CHF + CIA_PVD + CIA_DEM + CIA_COPD + CIA_DM + CIA_ .

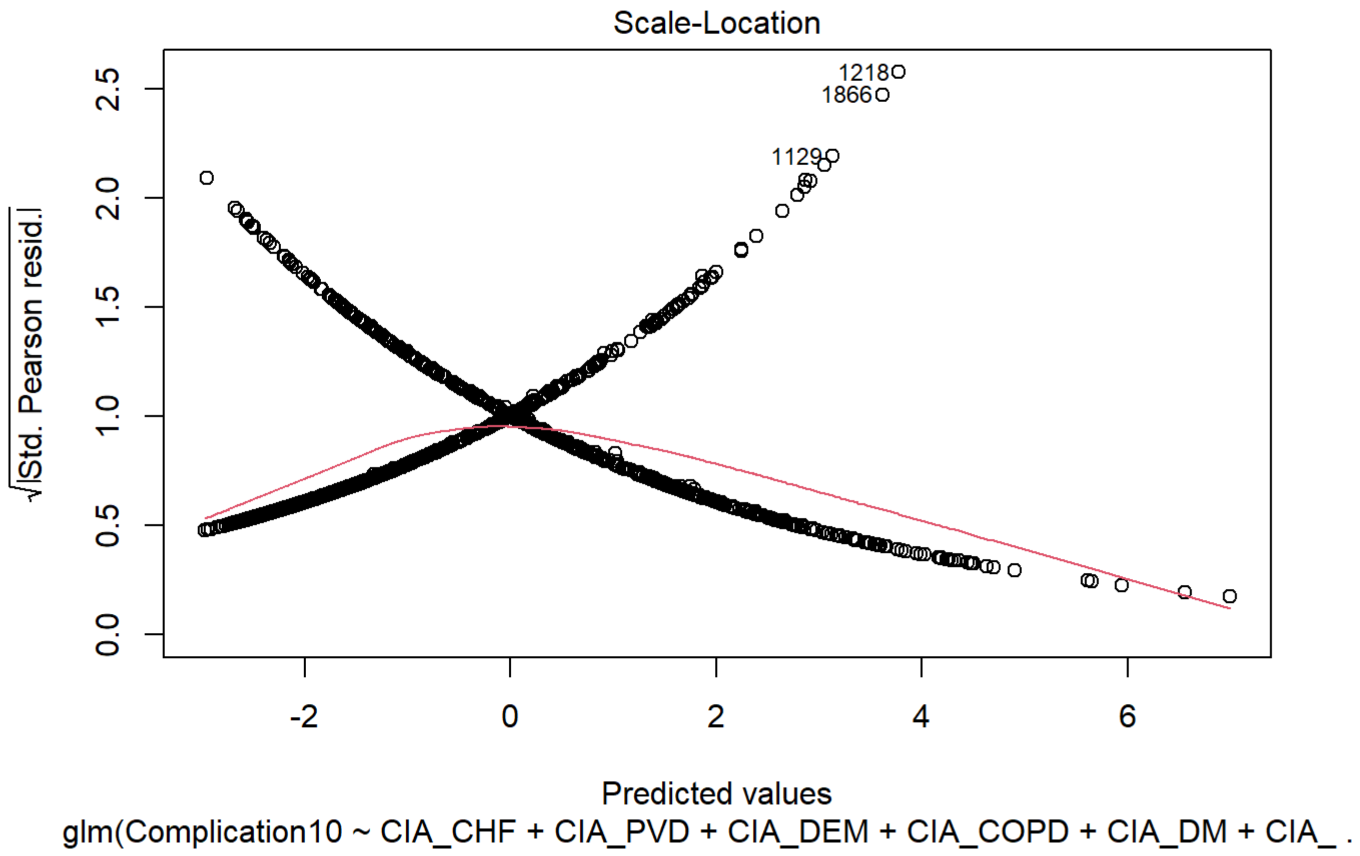
Scale-Location
√|Std. Pearson resid.|
0.0
0.5
1.0
1.5
2.0
2.5
-2
0
2
4
6
1218
1866
1129
Predicted values
glm(Complication10 ~ CIA_CHF + CIA_PVD + CIA_DEM + CIA_COPD + CIA_DM + CIA_ .

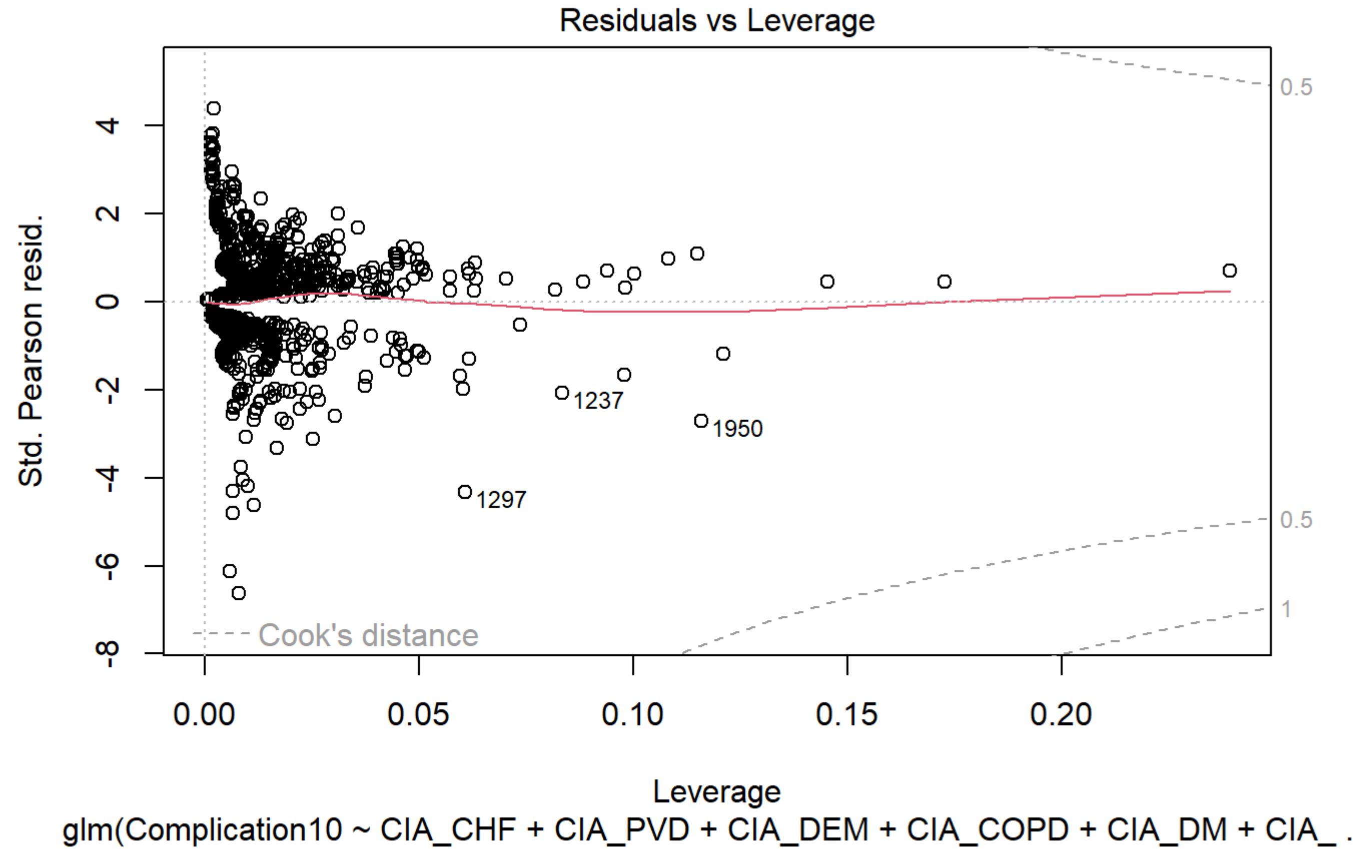
Residuals vs Leverage
Std. Pearson resid.
-8
-6
-4
-2
0
2
4
0.00
0.05
0.10
0.15
0.20
0.5
0.5
1
1237
1950
1297
Cook's distance
Leverage
glm(Complication10 ~ CIA_CHF + CIA_PVD + CIA_DEM + CIA_COPD + CIA_DM + CIA_ .

```
#
#
#
#

#Classification table (confusion matrix)
lranycx.org.final.pred <- predict(lranycx.org.final
                                  , newdata = train.org.na
                                  , type = "response")
lranycx.org.final.class <- ifelse(lranycx.org.final.pred>=0.5, 1, 0)
cl_table.lranycx.org <- table (train.org.na$Complication10, lranycx.org.final.class)
rownames(cl_table.lranycx.org) <- c("No Cx", "Complicated")
colnames(cl_table.lranycx.org) <- c("No Cx", "Complicated")
addmargins(cl_table.lranycx.org)
```

```
##              lranycx.org.final.class
##               No Cx Complicated  Sum
##   No Cx         831         129  960
##   Complicated   184         427  611
##   Sum          1015         556 1571
```

```
#model accuracy (classification accuracy)
mean((train.org.na$Complication10 == lranycx.org.final.class))
```

```
## [1] 0.8007638
```

```
#Discriminability

roc.lranycx.train <- roc(train.org.na$Complication10
          ,lranycx.org.final.pred
          , add = TRUE)
roc.lranycx.train$auc
```

```
## Area under the curve: 0.8587
```

```
ci(roc.lranycx.train)
```

```
## 95% CI: 0.8394-0.8779 (DeLong)
```

```
plot.roc(roc.lranycx.train
        , print.auc = TRUE
        , print.thres = FALSE
        , auc.polygon = TRUE
        )
```

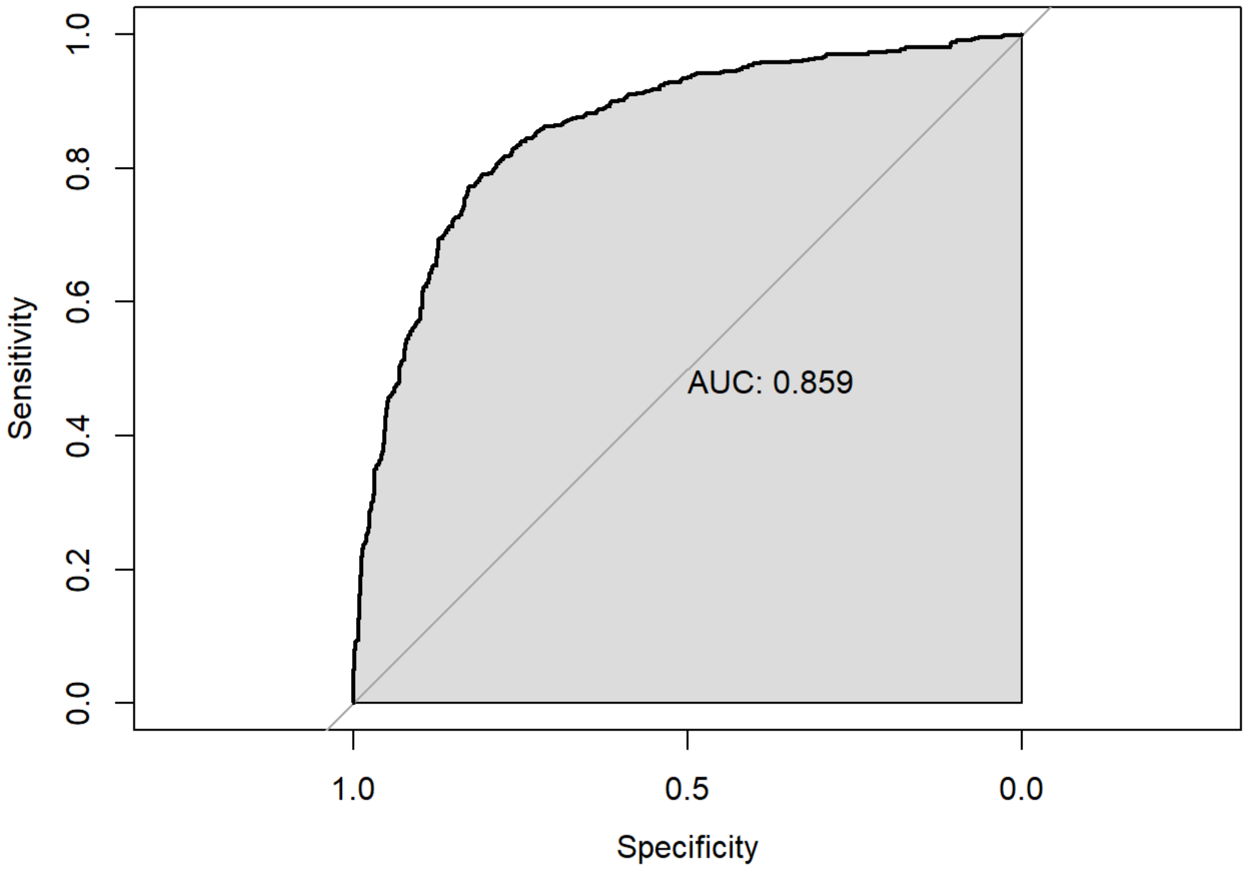


```
pROC::coords(roc.lranycx.train,
             "best",
             transpose = TRUE,
             best.method = "youden")
```

```
##   threshold specificity sensitivity
##   0.4002878   0.8270833   0.7725041
```

6.2 Imputed data analysis

6.2.1 Pooled logistic regression model

This is a logistic regression model with imputed datasets, results presented as “Pooled” coefficients.

```
# Logistic regression model with imput data: Enter all input parameters
lranycx.imp.full <- with(data = train.imp
               , exp = glm(Complication10 ~ GENDER
                                          + CIA_AMI
                                          + CIA_CHF
                                          + CIA_PVD
                                          + CIA_CEVD
                                          + CIA_DEM
                                          + CIA_COPD
                                          + CIA_PUD
                                          + CIA_LD_MLD
                                          + CIA_LD_SEV
                                          + CIA_DM
                                          + CIA_DM_CX
                                          + CIA_HEMIPLAEGIA
                                          + CIA_CKD
                                          + CIA_CA
                                          + CIA_CA_MET
                                          + PREOP_HB
                                          + PREOP_WCC
                                          + PREOP_NA
                                          + PREOP_K
                                          + SURG_SEV
                                          + SURG_SCHEDULED_TYPE
                                          + PreopICU
                           , family = "binomial"))

summary(pool(lranycx.imp.full))
```

```
##                  term     estimate  std.error   statistic         df
## 1         (Intercept)  0.462162192 2.86362654  0.16139053   94.81708
## 2             GENDER1 -0.155598742 0.12932015 -1.20320574 1847.20571
## 3            CIA_AMI1 -0.884285488 0.80860145 -1.09359869 1923.79492
## 4            CIA_CHF1  2.567431674 0.48292554  5.31641312 1922.99577
## 5            CIA_PVD1  1.381725120 0.39277361  3.51786646 1920.90001
## 6           CIA_CEVD1  0.275517320 0.51687274  0.53304672 1936.78825
## 7            CIA_DEM1  1.003091754 0.32186093  3.11653779 1940.79957
## 8           CIA_COPD1  1.384903041 0.65102785  2.12725621 1874.03545
## 9            CIA_PUD1  0.093854338 0.52564476  0.17855089 1928.67787
## 10        CIA_LD_MLD1  0.445897464 0.92172895  0.48376203 1939.28254
## 11        CIA_LD_SEV1  0.026917420 1.36914815  0.01965998 1934.87357
## 12            CIA_DM1  0.437913642 0.23041779  1.90052006 1905.75796
## 13         CIA_DM_CX1  0.657126303 0.22445107  2.92770398 1843.89194
## 14    CIA_HEMIPLAEGIA1 -0.127898588 0.72846876 -0.17557182 1923.31087
## 15           CIA_CKD1  1.010484463 0.28449812  3.55181426 1939.69765
## 16            CIA_CA1  0.535854136 0.21119446  2.53725470 1861.23134
## 17        CIA_CA_MET1  1.426246150 0.41634294  3.42565233 1899.19698
## 18           PREOP_HB -0.161514242 0.03496688 -4.61906309 1416.92127
## 19          PREOP_WCC  0.047139431 0.01817748  2.59328711  324.69807
## 20           PREOP_NA -0.004943976 0.01858106 -0.26607607  218.21032
## 21            PREOP_K -0.128640894 0.15093046 -0.85231899   41.97107
## 22          SURG_SEV1  1.009200290 0.13585046  7.42875877 1921.93414
## 23          SURG_SEV2  2.241897033 0.24023819  9.33197619 1929.89633
## 24 SURG_SCHEDULED_TYPE1  1.539161891 0.14231451 10.81521388 1727.12387
## 25          PreopICU1  0.861819733 0.81376698  1.05904976 1930.96677
##         p.value
## 1  8.721292e-01
## 2  2.290509e-01
## 3  2.742679e-01
## 4  1.181441e-07
## 5  4.451442e-04
## 6  5.940624e-01
## 7  1.856682e-03
## 8  3.352906e-02
## 9  8.583091e-01
## 10 6.286094e-01
## 11 9.843166e-01
## 12 5.751567e-02
## 13 3.456604e-03
## 14 8.606488e-01
## 15 3.917163e-04
## 16 1.125358e-02
## 17 6.263766e-04
## 18 4.204586e-06
## 19 9.936670e-03
## 20 7.904319e-01
## 21 3.988746e-01
## 22 1.639902e-13
## 23 2.757133e-20
## 24 2.004930e-26
## 25 2.897096e-01
```

```
# Stepwise selection method: AIC guided.
# Suppressed output
lranycx.imp.sel <- with(data = train.imp
                     , exp = step(glm(Complication10 ~ GENDER
                                        + CIA_AMI
                                        + CIA_CHF
                                        + CIA_PVD
                                        + CIA_CEVD
                                        + CIA_DEM
                                        + CIA_COPD
                                        + CIA_PUD
                                        + CIA_LD_MLD
                                        + CIA_LD_SEV
                                        + CIA_DM
                                        + CIA_DM_CX
                                        + CIA_HEMIPLAEGIA
                                        + CIA_CKD
                                        + CIA_CA
                                        + CIA_CA_MET
                                        + PREOP_HB
                                        + PREOP_WCC
                                        + PREOP_NA
                                        + PREOP_K
                                        + SURG_SEV
                                        + SURG_SCHEDULED_TYPE
                                        + PreopICU
                                 , family = binomial)))
```

```
summary(pool(lranycx.imp.sel))
```

```
##                   term    estimate  std.error  statistic         df
## 1          (Intercept) -0.52581282 0.76578754 -0.6866302   16.79791
## 2             CIA_CHF1  2.55225168 0.47570232  5.3652286 1919.27958
## 3             CIA_PVD1  1.35514575 0.39007225  3.4740891 1935.40424
## 4             CIA_DEM1  1.01798593 0.32066346  3.1746240 1949.46416
## 5            CIA_COPD1  1.36746382 0.64575021  2.1176359 1880.90787
## 6              CIA_DM1  0.41558336 0.22869658  1.8171822 1937.67653
## 7           CIA_DM_CX1  0.60277552 0.22128867  2.7239330 1928.77187
## 8             CIA_CKD1  1.01192310 0.28331790  3.5716878 1947.61041
## 9              CIA_CA1  0.54204896 0.20952158  2.5870794 1888.95951
## 10         CIA_CA_MET1  1.42244417 0.41639894  3.4160610 1914.07496
## 11            PREOP_HB -0.16482315 0.03436457 -4.7963108 1409.69440
## 12           PREOP_WCC  0.04754028 0.01797082  2.6454144  326.83677
## 13             PREOP_K -0.18348827 0.12475790 -1.4707547 1932.19583
## 14           SURG_SEV1  1.01800901 0.13371441  7.6133081 1937.46838
## 15           SURG_SEV2  2.30482055 0.23128139  9.9654390 1933.71557
## 16 SURG_SCHEDULED_TYPE1  1.58214237 0.14038567 11.2699703 1354.16406
##         p.value
## 1  5.016852e-01
## 2  9.062800e-08
## 3  5.238727e-04
## 4  1.523728e-03
## 5  3.433681e-02
## 6  6.934362e-02
## 7  6.509142e-03
## 8  3.632984e-04
## 9  9.753440e-03
## 10 6.486143e-04
## 11 1.787556e-06
## 12 8.553394e-03
## 13 1.415203e-01
## 14 4.147004e-14
## 15 7.596410e-23
## 16 3.188225e-28
```

```
# Final logistic regression model with the selected input parameters
lranycx.imp.final <- with(data = train.imp
                         , exp = glm(Complication10 ~ CIA_CHF
                                        + CIA_PVD
                                        + CIA_DEM
                                        + CIA_COPD
                                        + CIA_DM
                                        + CIA_DM_CX
                                        + CIA_CKD
                                        + CIA_CA
                                        + CIA_CA_MET
                                        + PREOP_HB
                                        + PREOP_WCC
                                        + PREOP_K
                                        + SURG_SEV
                                        + SURG_SCHEDULED_TYPE
                                        , family = binomial))
pool(lranycx.imp.final)
```

```
## Class: mipo    m = 5
##                    term m    estimate         ubar            b            t
## 1           (Intercept) 5 -0.29177137 0.4827815406 1.248781e-01 0.6326352701
## 2              CIA_CHF1 5  2.53727109 0.2250516062 7.270367e-04 0.2259240502
## 3              CIA_PVD1 5  1.35778263 0.1517945222 4.343455e-04 0.1523157368
## 4              CIA_DEM1 5  1.02056469 0.1027916454 1.117762e-05 0.1028050585
## 5             CIA_COPD1 5  1.37251052 0.4127752383 2.602592e-03 0.4158983485
## 6               CIA_DM1 5  0.41879189 0.0521637172 1.807874e-04 0.0523806621
## 7            CIA_DM_CX1 5  0.61191112 0.0490237411 3.124883e-04 0.0493987270
## 8              CIA_CKD1 5  1.01062086 0.0801006268 6.490266e-05 0.0801785100
## 9               CIA_CA1 5  0.53916241 0.0436749289 2.990420e-04 0.0440337793
## 10          CIA_CA_MET1 5  1.41351186 0.1728296016 6.958332e-04 0.1736646015
## 11             PREOP_HB 5 -0.16471828 0.0011494047 2.694836e-05 0.0011817427
## 12            PREOP_WCC 5  0.04811816 0.0002917358 2.325285e-05 0.0003196392
## 13              PREOP_K 5 -0.12670702 0.0155563792 5.245813e-03 0.0218513551
## 14            SURG_SEV1 5  1.01969679 0.0178486739 5.034963e-05 0.0179090934
## 15            SURG_SEV2 5  2.30782017 0.0533718851 1.105648e-04 0.0535045629
## 16 SURG_SCHEDULED_TYPE1 5  1.57335101 0.0193509004 2.380366e-04 0.0196365444
##    dfcom         df          riv       lambda         fmi
## 1   1952   68.03133 0.3103965602 0.2368722337 0.258359310
## 2   1952 1928.50698 0.0038766398 0.0038616695 0.004893132
## 3   1952 1932.33736 0.0034336852 0.0034219353 0.004451811
## 4   1952 1949.73247 0.0001304887 0.0001304717 0.001154544
## 5   1952 1883.95868 0.0075661277 0.0075093113 0.008561259
## 6   1952 1925.88835 0.0041589233 0.0041416983 0.005174271
## 7   1952 1882.71395 0.0076490676 0.0075910035 0.008643559
## 8   1952 1947.21406 0.0009723169 0.0009713724 0.001995905
## 9   1952 1873.93460 0.0082163932 0.0081494342 0.009206318
## 10  1952 1919.10285 0.0048313474 0.0048081177 0.005843642
## 11  1952 1399.66952 0.0281345937 0.0273646990 0.028751534
## 12  1952  405.34352 0.0956461932 0.0872966052 0.091766878
## 13  1952   46.58073 0.4046555999 0.2880817190 0.316799258
## 14  1952 1932.73658 0.0033851008 0.0033736806 0.004403393
## 15  1952 1939.36831 0.0024859100 0.0024797456 0.003506863
## 16  1952 1744.31759 0.0147612744 0.0145465488 0.015674510
```

```
lranycx.imp.final.est <- summary(pool(lranycx.imp.final))

lranycx.imp.final.est %>%
  mutate(OR = exp(estimate)
         , Low.CI = exp(estimate - 1.96*std.error)
         , Up.CI = exp(estimate + 1.96*std.error)
         , Sig = ifelse(p.value < 0.05, "*","")
         ) %>%
  select(-c("statistic", "df"))
```

```
##                   term    estimate  std.error      p.value         OR
## 1          (Intercept) -0.29177137 0.79538373 7.148836e-01  0.7469393
## 2             CIA_CHF1  2.53727109 0.47531469 1.050086e-07 12.6451165
## 3             CIA_PVD1  1.35778263 0.39027649 5.143900e-04  3.8875636
## 4             CIA_DEM1  1.02056469 0.32063228 1.480674e-03  2.7747612
## 5            CIA_COPD1  1.37251052 0.64490181 3.344605e-02  3.9452429
## 6              CIA_DM1  0.41879189 0.22886822 6.742859e-02  1.5201240
## 7           CIA_DM_CX1  0.61191112 0.22225824 5.959096e-03  1.8439520
## 8             CIA_CKD1  1.01062086 0.28315810 3.668773e-04  2.7473062
## 9              CIA_CA1  0.53916241 0.20984227 1.026510e-02  1.7145702
## 10         CIA_CA_MET1  1.41351186 0.41673085 7.081664e-04  4.1103651
## 11            PREOP_HB -0.16471828 0.03437649 1.830684e-06  0.8481326
## 12           PREOP_WCC  0.04811816 0.01787846 7.409732e-03  1.0492946
## 13             PREOP_K -0.12670702 0.14782204 3.957455e-01  0.8809917
## 14           SURG_SEV1  1.01969679 0.13382486 3.958503e-14  2.7723540
## 15           SURG_SEV2  2.30782017 0.23131053 6.766225e-23 10.0524880
## 16 SURG_SCHEDULED_TYPE1  1.57335101 0.14013045 2.711281e-28  4.8227823
##       Low.CI      Up.CI Sig
## 1  0.1571235  3.5508277
## 2  4.9811177 32.1010223   *
## 3  1.8091203  8.3538670   *
## 4  1.4801233  5.2017960   *
## 5  1.1146082 13.9644955   *
## 6  0.9706495  2.3806501
## 7  1.1927779  2.8506222   *
## 8  1.5771682  4.7855968   *
## 9  1.1364070  2.5868819   *
## 10 1.8161509  9.3026967   *
## 11 0.7928698  0.9072472   *
## 12 1.0131623  1.0867156   *
## 13 0.6593913  1.1770651
## 14 2.1327241  3.6038168   *
## 15 6.3881935 15.8186372   *
## 16 3.6645125  6.3471551   *
```

6.2.1.1 Object for the combined-predict probability and model diagnostics

mice::pool function does not provide the object with pooled estimates, we need a small trick to make this object. FYI, CI cannot be calculated with this trick.

```
# Pooled logistic regression model object

# Copy one of the fitted lr models
lranycx.pool <- lranycx.imp.final$analyses[1]

# Pooled coefficients
repl.pool <- summary(pool(lranycx.imp.final))$estimate

# Insert name: named numeric vector objective
names(repl.pool) <- names(lranycx.pool[[1]]$coefficients)

# Replace the fitted coefficents with the pooled estimates
lranycx.pool[[1]]$coefficients <- repl.pool
lranycx.imp <- lranycx.pool[[1]]

# Check the replaced result

  # Coefficient: imputed data 1
  lranycx.imp.final$analyses[1]
```

```
## [[1]]
##
## Call:  glm(formula = Complication10 ~ CIA_CHF + CIA_PVD + CIA_DEM +
##     CIA_COPD + CIA_DM + CIA_DM_CX + CIA_CKD + CIA_CA + CIA_CA_MET +
##     PREOP_HB + PREOP_WCC + PREOP_K + SURG_SEV + SURG_SCHEDULED_TYPE,
##     family = binomial)
##
## Coefficients:
##         (Intercept)              CIA_CHF1              CIA_PVD1
##             0.01583               2.50717               1.36026
##            CIA_DEM1             CIA_COPD1              CIA_DM1
##             1.02170               1.33887               0.41353
##          CIA_DM_CX1              CIA_CKD1              CIA_CA1
##             0.60556               1.00494               0.53265
##         CIA_CA_MET1              PREOP_HB             PREOP_WCC
##             1.37480              -0.16934               0.05194
##             PREOP_K             SURG_SEV1             SURG_SEV2
##            -0.18665               1.00825               2.31137
## SURG_SCHEDULED_TYPE1
##             1.55656
##
## Degrees of Freedom: 1967 Total (i.e. Null);  1952 Residual
## Null Deviance:       2493
## Residual Deviance: 1634  AIC: 1666
```

```
# Coefficient: pooled model generated
lranycx.imp
```

```
##
## Call:  glm(formula = Complication10 ~ CIA_CHF + CIA_PVD + CIA_DEM +
##     CIA_COPD + CIA_DM + CIA_DM_CX + CIA_CKD + CIA_CA + CIA_CA_MET +
##     PREOP_HB + PREOP_WCC + PREOP_K + SURG_SEV + SURG_SCHEDULED_TYPE,
##     family = binomial)
##
## Coefficients:
##         (Intercept)              CIA_CHF1              CIA_PVD1
##            -0.29177               2.53727               1.35778
##            CIA_DEM1             CIA_COPD1              CIA_DM1
##             1.02056               1.37251               0.41879
##          CIA_DM_CX1              CIA_CKD1              CIA_CA1
##             0.61191               1.01062               0.53916
##         CIA_CA_MET1              PREOP_HB             PREOP_WCC
##             1.41351              -0.16472               0.04812
##             PREOP_K             SURG_SEV1             SURG_SEV2
##            -0.12671               1.01970               2.30782
## SURG_SCHEDULED_TYPE1
##             1.57335
##
## Degrees of Freedom: 1967 Total (i.e. Null);  1952 Residual
## Null Deviance:       2493
## Residual Deviance: 1634  AIC: 1666
```

```
# Coefficient: estimated pooled model
summary(pool(lranycx.imp.final))$estimate
```

```
## [1] -0.29177137  2.53727109  1.35778263  1.02056469  1.37251052  0.41879189
## [7]  0.61191112  1.01062086  0.53916241  1.41351186 -0.16471828  0.04811816
## [13] -0.12670702  1.01969679  2.30782017  1.57335101
```

Then, we can get the combined-predict probability

Reference: Miles A. Obtaining Predictions from Models Fit to Multiply Imputed Data. Sociological Methods & Research. 2016;45(1):175-185. doi:10.1177/0049124115610345 (doi:10.1177/0049124115610345)

```
# Combined-predict probability of training dataset
lranycx.imp.train.pred <- predict(lranycx.imp
                                  , newdata = train.org.na
                                  , type = "response")
lranycx.imp.train.class <- ifelse(lranycx.imp.train.pred>=0.5, 1, 0)

# Confusion matrix
cl_table.lranycx.imp.train <- table (train.org.na$Complication10, lranycx.imp.train.class)
rownames(cl_table.lranycx.imp.train) <- c("No Cx", "Complicated")
colnames(cl_table.lranycx.imp.train) <- c("No Cx", "Complicated")
addmargins(cl_table.lranycx.imp.train)
```

```
##              lranycx.imp.train.class
##               No Cx Complicated  Sum
##   No Cx         838         122  960
##   Complicated   204         407  611
##   Sum          1042         529 1571
```

```
# Sensitivity
round(cl_table.lranycx.imp.train[2,2]/(cl_table.lranycx.imp.train[2,1] + cl_table.lranycx.imp.train
[2,2]),3)
```

```
## [1] 0.666
```

```
# Specificity
round(cl_table.lranycx.imp.train[1,1]/(cl_table.lranycx.imp.train[1,1] + cl_table.lranycx.imp.train
[1,2]),3)
```

```
## [1] 0.873
```

```
# Positive predictive rate
round(cl_table.lranycx.imp.train[2,2]/(cl_table.lranycx.imp.train[1,2] + cl_table.lranycx.imp.train
[2,2]),3)
```

```
## [1] 0.769
```

```
# Negative predictive rate
round(cl_table.lranycx.imp.train[1,1]/(cl_table.lranycx.imp.train[1,1] + cl_table.lranycx.imp.train
[2,1]),3)
```

```
## [1] 0.804
```

```
# Model accuracy (classification accuracy)
mean((train.org.na$Complication10 == lranycx.imp.train.class))
```

```
## [1] 0.7924889
```

```
# Discriminability
roc.lranycx.imp.train <- roc(train.org.na$Complication10
                         ,lranycx.imp.train.pred
                         , add = TRUE)
```

```
## Setting levels: control = 0, case = 1
```

```
## Setting direction: controls < cases
```

```
roc.lranycx.imp.train$auc
```

```
## Area under the curve: 0.8568
```

```
ci(roc.lranycx.imp.train)
```

```
## 95% CI: 0.8374-0.8761 (DeLong)
```

```
plot.roc(roc.lranycx.imp.train
         , print.auc = TRUE
         , print.thres = FALSE
         , auc.polygon = TRUE
)
```

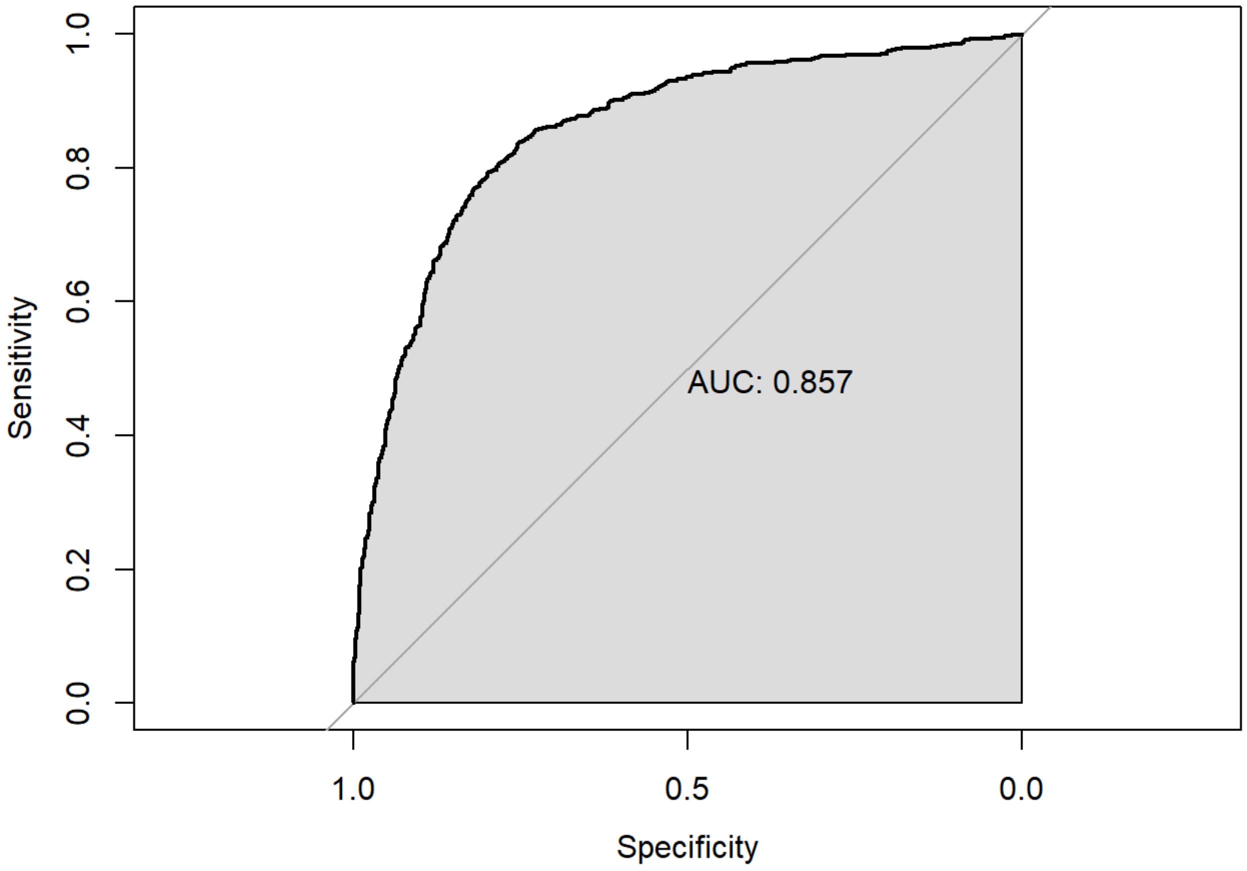


```
pROC::coords(roc.lranycx.imp.train,
             "best",
             transpose = TRUE,
             best.method = "youden")
```

```
##   threshold specificity sensitivity
##   0.3226489   0.7989583   0.7937807
```

Using the estimated threshold from ROC (0.3226489), make a variable of assisting clinical decision of Yes/No.

```
#Threshold discrimination ability

lranycx.imp.train.pred.dcs <- ifelse(lranycx.imp.train.pred > 0.3226489, 1, 0)

roc.lranycx.imp.train.dcs <- roc(train.org.na$Complication10
                        ,lranycx.imp.train.pred.dcs
                        , add = TRUE)
```

```
## Setting levels: control = 0, case = 1
```

```
## Setting direction: controls < cases
```

```
roc.lranycx.imp.train.dcs$auc
```

```
## Area under the curve: 0.7964
```

```
ci(roc.lranycx.imp.train.dcs)
```

```
## 95% CI: 0.7759-0.8168 (DeLong)
```

```
plot.roc(roc.lranycx.imp.train.dcs
         , print.auc = TRUE
         , print.thres = FALSE
         , auc.polygon = TRUE
)
```

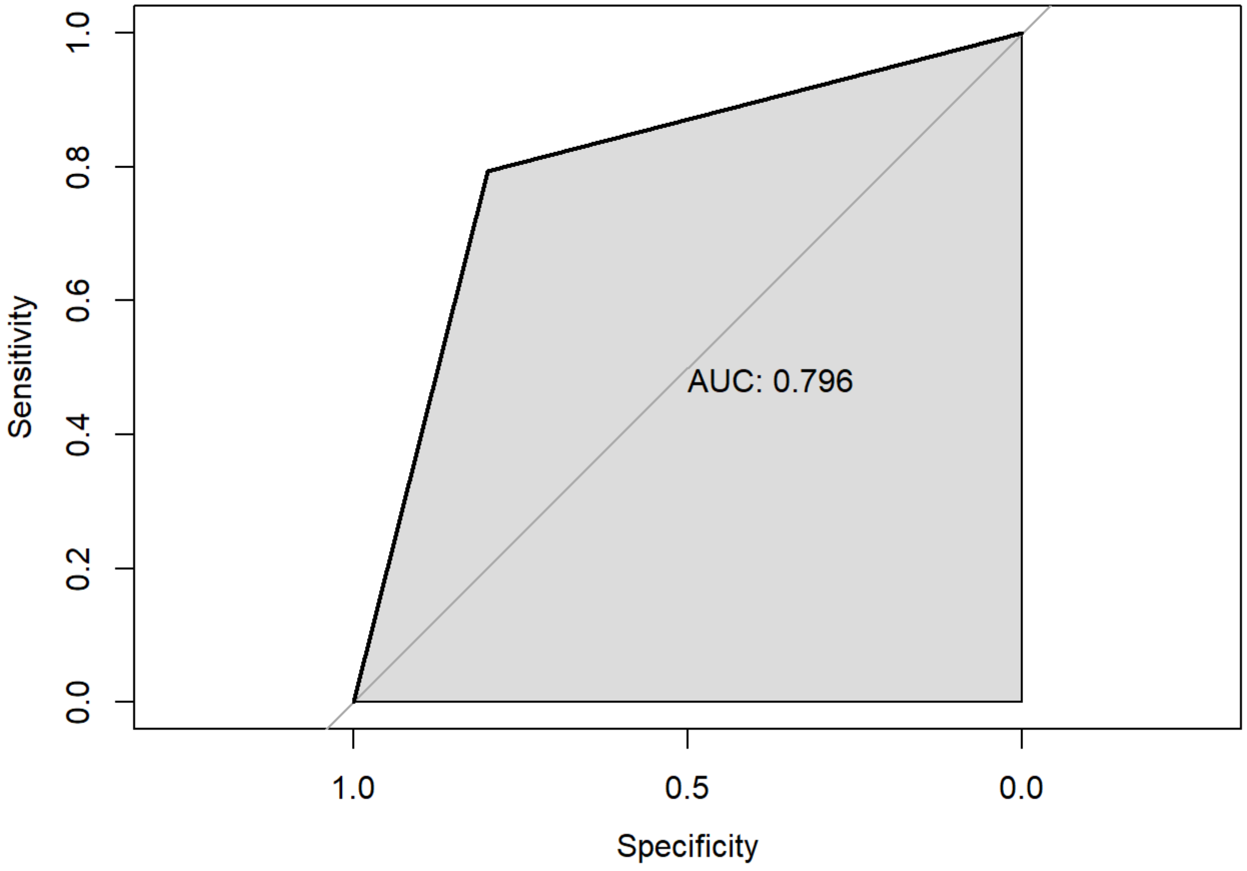


```
pROC::coords(roc.lranycx.imp.train.dcs,
             "best",
             transpose = TRUE,
             best.method = "youden")
```

```
##   threshold specificity sensitivity
##   0.5000000   0.7989583   0.7937807
```

```
# Calibrations: on the training dataset
cal.lranycx.imp.train <- val.prob(lranycx.imp.train.pred
                        , train.org.na$Complication10
                        , pl = FALSE)
cal.lranycx.imp.train
```

```
##          Dxy      C (ROC)           R2            D     D:Chi-sq          D:p
## 7.135911e-01 8.567956e-01 4.643560e-01 4.184362e-01 6.583632e+02           NA
##            U     U:Chi-sq          U:p            Q        Brier    Intercept
## 2.082488e-03 5.271589e+00 7.166202e-02 4.163537e-01 1.471414e-01 1.088167e-01
##        Slope         Emax          E90         Eavg          S:z          S:p
## 9.496596e-01 7.802466e-02 6.620994e-02 2.752226e-02 1.701470e+00 8.885485e-02
```

```
CalibrationCurves::val.prob.ci.2(lranycx.imp.train.pred, train.org.na$Complication10
              , lty.smooth = 2
              , CL.smooth = FALSE
              , col.ideal = "black")
```

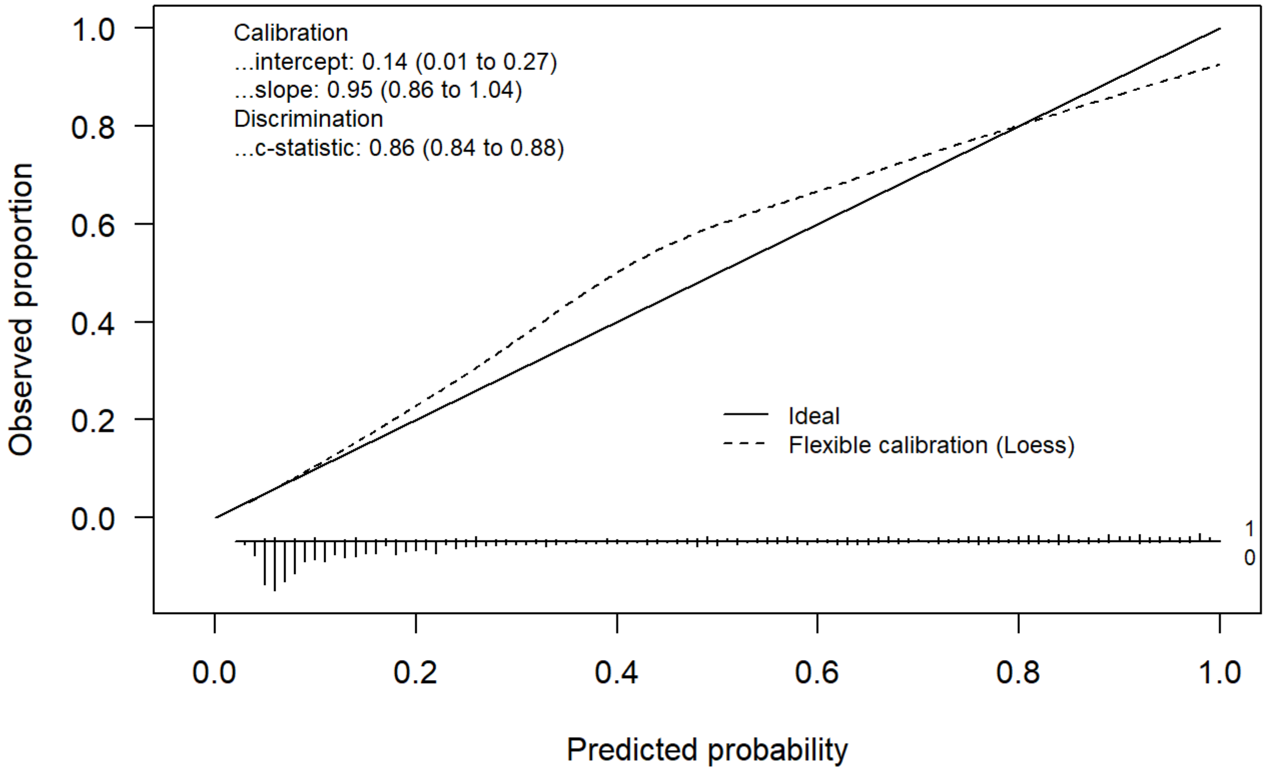


```
hosmer_lemeshow(train.org.na$Complication10, lranycx.imp.train.pred, 10, 'C')
```

```
## PVALUE 0.00511857
## stat 21.8927
```

```
## [1] 0.00511857
```

```
hosmer_lemeshow(train.org.na$Complication10, lranycx.imp.train.pred, 10, 'H')
```

```
## PVALUE 0.02594132
## stat 17.42878
```

```
## [1] 0.02594132
```

6.3 Sensitivity analysis

Using the delta adjusted imputation dataset, compare the estimated ORs.

```
library(finalfit)
```

```
# delta = 0
lranycx.dlt0 <- with(data = train.imp.delta[[1]]
                     , exp = glm(Complication10 ~ CIA_CHF
                                 + CIA_PVD
                                 + CIA_DEM
                                 + CIA_COPD
                                 + CIA_DM
                                 + CIA_DM_CX
                                 + CIA_CKD
                                 + CIA_CA
                                 + CIA_CA_MET
                                 + PREOP_HB
                                 + PREOP_WCC
                                 + PREOP_K
                                 + SURG_SEV
                                 + SURG_SCHEDULED_TYPE
                                 , family = binomial))

lranycx.dlt0.est <- summary(pool(lranycx.dlt0)
                       , conf.int = TRUE
                       , exponentiate = TRUE)

# delta = mean + 1*SD
lranycx.dlt1 <- with(data = train.imp.delta[[2]]
                     , exp = glm(Complication10 ~ CIA_CHF
                                 + CIA_PVD
                                 + CIA_DEM
                                 + CIA_COPD
                                 + CIA_DM
                                 + CIA_DM_CX
                                 + CIA_CKD
                                 + CIA_CA
                                 + CIA_CA_MET
                                 + PREOP_HB
                                 + PREOP_WCC
                                 + PREOP_K
                                 + SURG_SEV
                                 + SURG_SCHEDULED_TYPE
                                 , family = binomial))

lranycx.dlt1.est <- summary(pool(lranycx.dlt1)
                       , conf.int = TRUE
                       , exponentiate = TRUE)

# delta = mean + 2*SD
lranycx.dlt2 <- with(data = train.imp.delta[[3]]
                     , exp = glm(Complication10 ~ CIA_CHF
                                 + CIA_PVD
                                 + CIA_DEM
                                 + CIA_COPD
                                 + CIA_DM
                                 + CIA_DM_CX
                                 + CIA_CKD
                                 + CIA_CA
                                 + CIA_CA_MET
                                 + PREOP_HB
                                 + PREOP_WCC
                                 + PREOP_K
                                 + SURG_SEV
```

```
                                              + SURG_SCHEDULED_TYPE
                                              , family = binomial))

lranycx.dlt2.est <- summary(pool(lranycx.dlt2)
                            , conf.int = TRUE
                            , exponentiate = TRUE)

lranycx.dlt0.est
```

```
##                    term   estimate  std.error  statistic         df
## 1           (Intercept)  0.7469393 0.79538373 -0.3668309   68.03133
## 2              CIA_CHF1 12.6451165 0.47531469  5.3380869 1928.50698
## 3              CIA_PVD1  3.8875636 0.39027649  3.4790275 1932.33736
## 4              CIA_DEM1  2.7747612 0.32063228  3.1829755 1949.73247
## 5             CIA_COPD1  3.9452429 0.64490181  2.1282473 1883.95868
## 6               CIA_DM1  1.5201240 0.22886822  1.8298386 1925.88835
## 7            CIA_DM_CX1  1.8439520 0.22225824  2.7531538 1882.71395
## 8              CIA_CKD1  2.7473062 0.28315810  3.5691045 1947.21406
## 9               CIA_CA1  1.7145702 0.20984227  2.5693699 1873.93460
## 10          CIA_CA_MET1  4.1103651 0.41673085  3.3919060 1919.10285
## 11             PREOP_HB  0.8481326 0.03437649 -4.7915976 1399.66952
## 12            PREOP_WCC  1.0492946 0.01787846  2.6914046  405.34352
## 13              PREOP_K  0.8809917 0.14782204 -0.8571592   46.58073
## 14            SURG_SEV1  2.7723540 0.13382486  7.6196365 1932.73658
## 15            SURG_SEV2 10.0524880 0.23131053  9.9771512 1939.36831
## 16 SURG_SCHEDULED_TYPE1  4.8227823 0.14013045 11.2277593 1744.31759
##         p.value     2.5 %     97.5 %
## 1  7.148836e-01 0.1527547  3.6523796
## 2  1.050086e-07 4.9782896 32.1192586
## 3  5.143900e-04 1.8082786  8.3577556
## 4  1.480674e-03 1.4795628  5.2037669
## 5  3.344605e-02 1.1137287 13.9755226
## 6  6.742859e-02 0.9703838  2.3813021
## 7  5.959096e-03 1.1924532  2.8513983
## 8  3.668773e-04 1.5766400  4.7872001
## 9  1.026510e-02 1.1361136  2.5875501
## 10 7.081664e-04 1.8152423  9.3073533
## 11 1.830684e-06 0.7928246  0.9072989
## 12 7.409732e-03 1.0130566  1.0868289
## 13 3.957455e-01 0.6543210  1.1861861
## 14 3.958503e-14 2.1323838  3.6043918
## 15 6.766225e-23 6.3864384 15.8229846
## 16 2.711281e-28 3.6638322  6.3483337
```

```
lranycx.dlt1.est
```

```
##                   term   estimate  std.error  statistic         df
## 1          (Intercept)  0.5648242 0.63088217 -0.9054636  854.07553
## 2             CIA_CHF1 12.5758826 0.47150621  5.3695600 1919.73570
## 3             CIA_PVD1  3.8560437 0.39239110  3.4395319 1922.81499
## 4             CIA_DEM1  2.5649890 0.31416222  2.9983050 1888.43058
## 5            CIA_COPD1  4.0879527 0.64394104  2.1866043 1936.01948
## 6              CIA_DM1  1.4527178 0.22859809  1.6335927 1948.22553
## 7           CIA_DM_CX1  1.8197101 0.22116335  2.7069458 1949.66680
## 8             CIA_CKD1  2.8671494 0.28161088  3.7403324 1936.98022
## 9              CIA_CA1  1.6510860 0.21129833  2.3731056 1947.61838
## 10         CIA_CA_MET1  3.8281694 0.41630262  3.2245455 1946.69208
## 11            PREOP_HB  0.8989390 0.01595550 -6.6773257  867.64587
## 12           PREOP_WCC  1.0445643 0.02087834  2.0882810   20.22176
## 13             PREOP_K  0.8570787 0.12840445 -1.2010919  917.42323
## 14           SURG_SEV1  2.5450297 0.13441829  6.9495182 1939.49303
## 15           SURG_SEV2  9.1720957 0.23027717  9.6239057 1946.74284
## 16 SURG_SCHEDULED_TYPE1  4.4364240 0.14102900 10.5641295 1116.82802
##         p.value     2.5 %     97.5 %
## 1  3.654752e-01 0.1637357  1.9484229
## 2  8.850728e-08 4.9881380 31.7057837
## 3  5.951554e-04 1.7861900  8.3244633
## 4  2.750306e-03 1.3851558  4.7497678
## 5  2.889075e-02 1.1562170 14.4534778
## 6  1.025060e-01 0.9278489  2.2744965
## 7  6.849773e-03 1.1793173  2.8078489
## 8  1.891199e-04 1.6504137  4.9809000
## 9  1.773578e-02 1.0909388  2.4988432
## 10 1.282627e-03 1.6920503  8.6610199
## 11 4.340620e-11 0.8712242  0.9275355
## 12 4.962331e-02 1.0000790  1.0910283
## 13 2.300256e-01 0.6661595  1.1027147
## 14 4.987180e-12 1.9552593  3.3126943
## 15 1.886945e-21 5.8389445 14.4079704
## 16 6.263490e-25 3.3640211  5.8506941
```

```
lranycx.dlt2.est
```

```
##                   term   estimate  std.error statistic        df      p.value
## 1          (Intercept)  0.4738958 0.64643306 -1.155213  251.8108 2.490982e-01
## 2             CIA_CHF1 12.8854492 0.47076810  5.429634 1948.0140 6.353172e-08
## 3             CIA_PVD1  3.8956437 0.39333210  3.457279 1921.1453 5.575401e-04
## 4             CIA_DEM1  2.5455995 0.31314293  2.983833 1942.9768 2.882108e-03
## 5            CIA_COPD1  3.9466663 0.64439890  2.130468 1945.3478 3.325812e-02
## 6              CIA_DM1  1.4455278 0.22875263  1.610799 1931.9214 1.073869e-01
## 7           CIA_DM_CX1  1.8382726 0.22157771  2.747688 1931.7816 6.057482e-03
## 8             CIA_CKD1  2.9220505 0.28227608  3.798712 1922.7339 1.499688e-04
## 9              CIA_CA1  1.6470968 0.21144616  2.360006 1945.3156 1.837287e-02
## 10         CIA_CA_MET1  3.8477052 0.41576018  3.240996 1937.0338 1.211337e-03
## 11            PREOP_HB  0.9067645 0.01517910 -6.447846  262.4612 5.418369e-10
## 12           PREOP_WCC  1.0506431 0.01651511  2.991348  183.9096 3.158348e-03
## 13             PREOP_K  0.8624507 0.13246416 -1.117112  304.1743 2.648279e-01
## 14           SURG_SEV1  2.5379759 0.13440878  6.929360 1942.8492 5.729189e-12
## 15           SURG_SEV2  9.0359717 0.23017341  9.563283 1922.4890 3.350871e-21
## 16 SURG_SCHEDULED_TYPE1  4.4140625 0.13888031 10.691188 1859.8640 6.232312e-26
##        2.5 %     97.5 %
## 1  0.1326724  1.6927197
## 2  5.1183726 32.4389831
## 3  1.8012055  8.4254903
## 4  1.3774511  4.7043970
## 5  1.1152586 13.9664244
## 6  0.9229747  2.2639305
## 7  1.1903766  2.8388042
## 8  1.6798192  5.0829157
## 9  1.0879872  2.4935293
## 10 1.7024910  8.6959847
## 11 0.8800639  0.9342753
## 12 1.0169612  1.0854405
## 13 0.6645538  1.1192791
## 14 1.9498770  3.3034502
## 15 5.7534384 14.1913024
## 16 3.3616025  5.7960296
```

```
explanatory.anycx = c("CIA_CHF", "CIA_PVD", "CIA_DEM", "CIA_COPD", "CIA_DM"
                , "CIA_DM_CX", "CIA_CKD", "CIA_CA", "CIA_CA_MET"
                , "PREOP_HB", "PREOP_WCC", "PREOP_K", "SURG_SEV", "SURG_SCHEDULED_TYPE")

complete(train.imp.delta[[1]]) %>%
  or_plot("Complication10", explanatory.anycx
          , glmfit = pool(lranycx.dlt0)
          , confint_type = "profile"
          , table_text_size = 3
          , title_text_size = 10
          , dependent_label = "Any complication: delta = 0")
```

Any complication: delta = 0: OR (95% CI, p-value)

CIA_CHF	0	-
	1	12.65 (4.98-32.12, p<0.001)
CIA_PVD	0	-
	1	3.89 (1.81-8.36, p=0.001)
CIA_DEM	0	-
	1	2.77 (1.48-5.20, p=0.001)
CIA_COPD	0	-
	1	3.95 (1.11-13.98, p=0.033)
CIA_DM	0	-
	1	1.52 (0.97-2.38, p=0.067)
CIA_DM_CX	0	-
	1	1.84 (1.19-2.85, p=0.006)
CIA_CKD	0	-
	1	2.75 (1.58-4.79, p<0.001)
CIA_CA	0	-
	1	1.71 (1.14-2.59, p=0.010)
CIA_CA_MET	0	-
	1	4.11 (1.82-9.31, p=0.001)
PREOP_HB	[4.3,18.2]	0.85 (0.79-0.91, p<0.001)
PREOP_WCC	[1.4,82.5]	1.05 (1.01-1.09, p=0.007)
PREOP_K	[2.8,6.4]	0.88 (0.65-1.19, p=0.396)
SURG_SEV	0	-
	1	2.77 (2.13-3.60, p<0.001)
	2	10.05 (6.39-15.82, p<0.001)
SURG_SCHEDULED_TYPE	0	-
	1	4.82 (3.66-6.35, p<0.001)

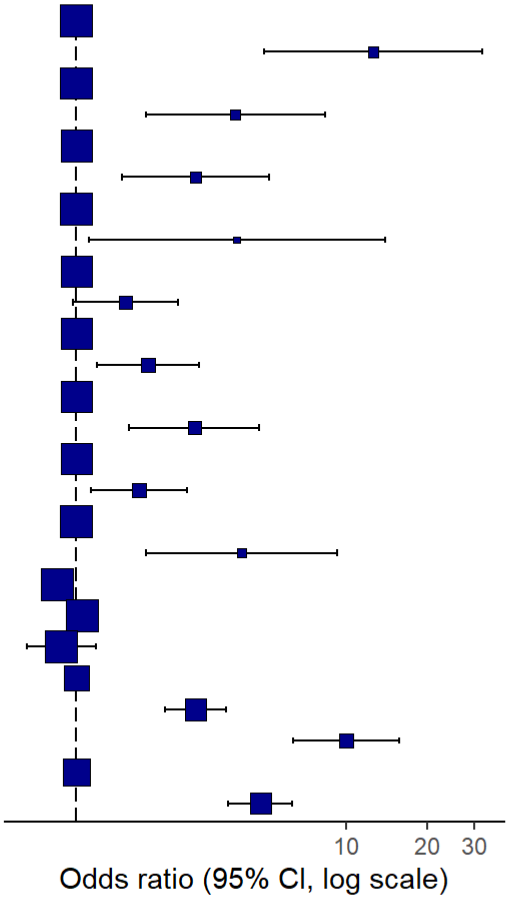


```
complete(train.imp.delta[[2]]) %>%
  or_plot("Complication10", explanatory.anycx
          , glmfit = pool(lranycx.dlt1)
          , confint_type = "profile"
          , table_text_size = 3
          , title_text_size = 10
          , dependent_label = "Any complication: delta = m + 1*SD")
```

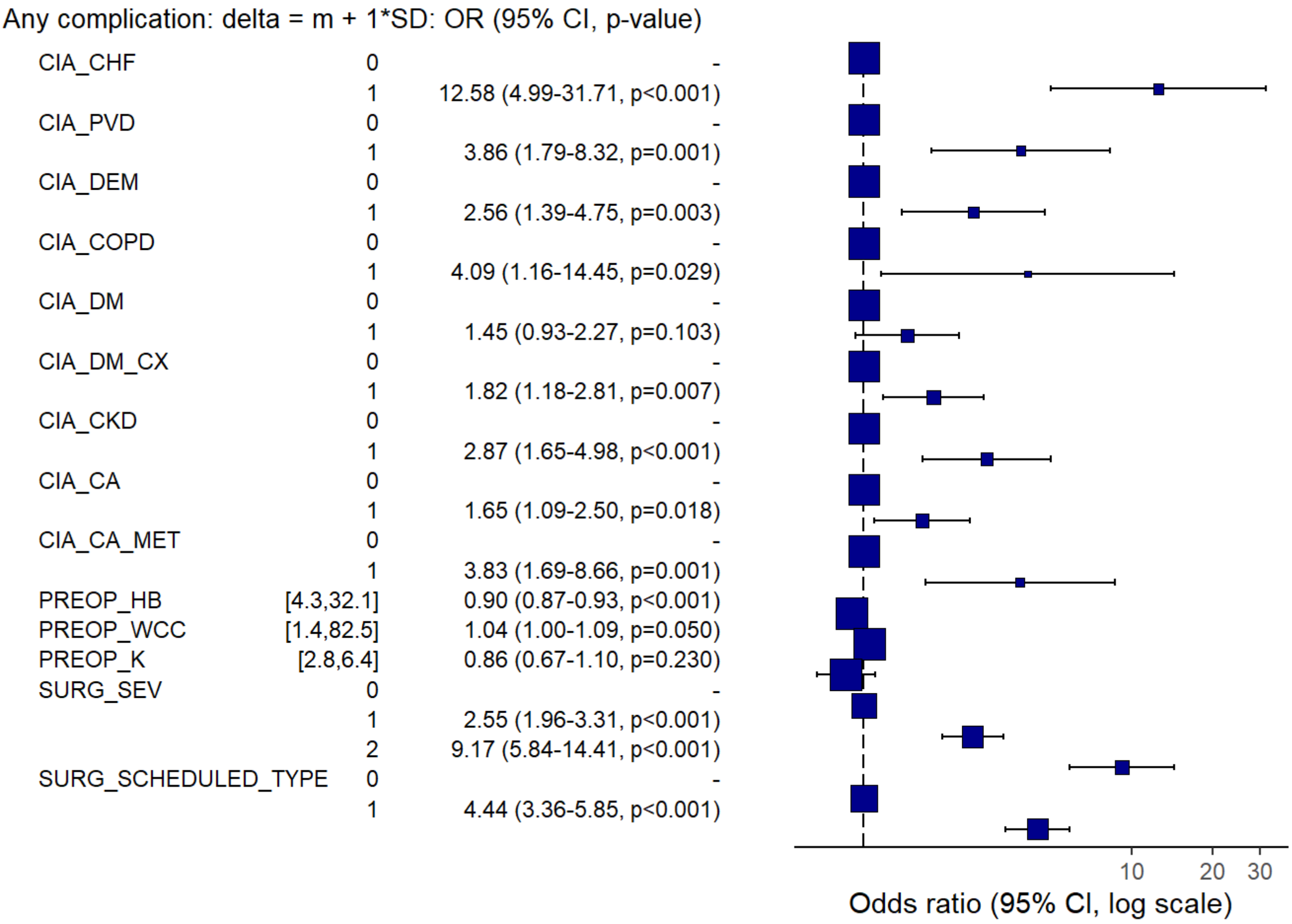


```
complete(train.imp.delta[[3]]) %>%
  or_plot("Complication10", explanatory.anycx
          , glmfit = pool(lranycx.dlt2)
          , confint_type = "profile"
          , table_text_size = 3
          , title_text_size = 10
          , dependent_label = "Any complication: delta = m + 2*SD")
```

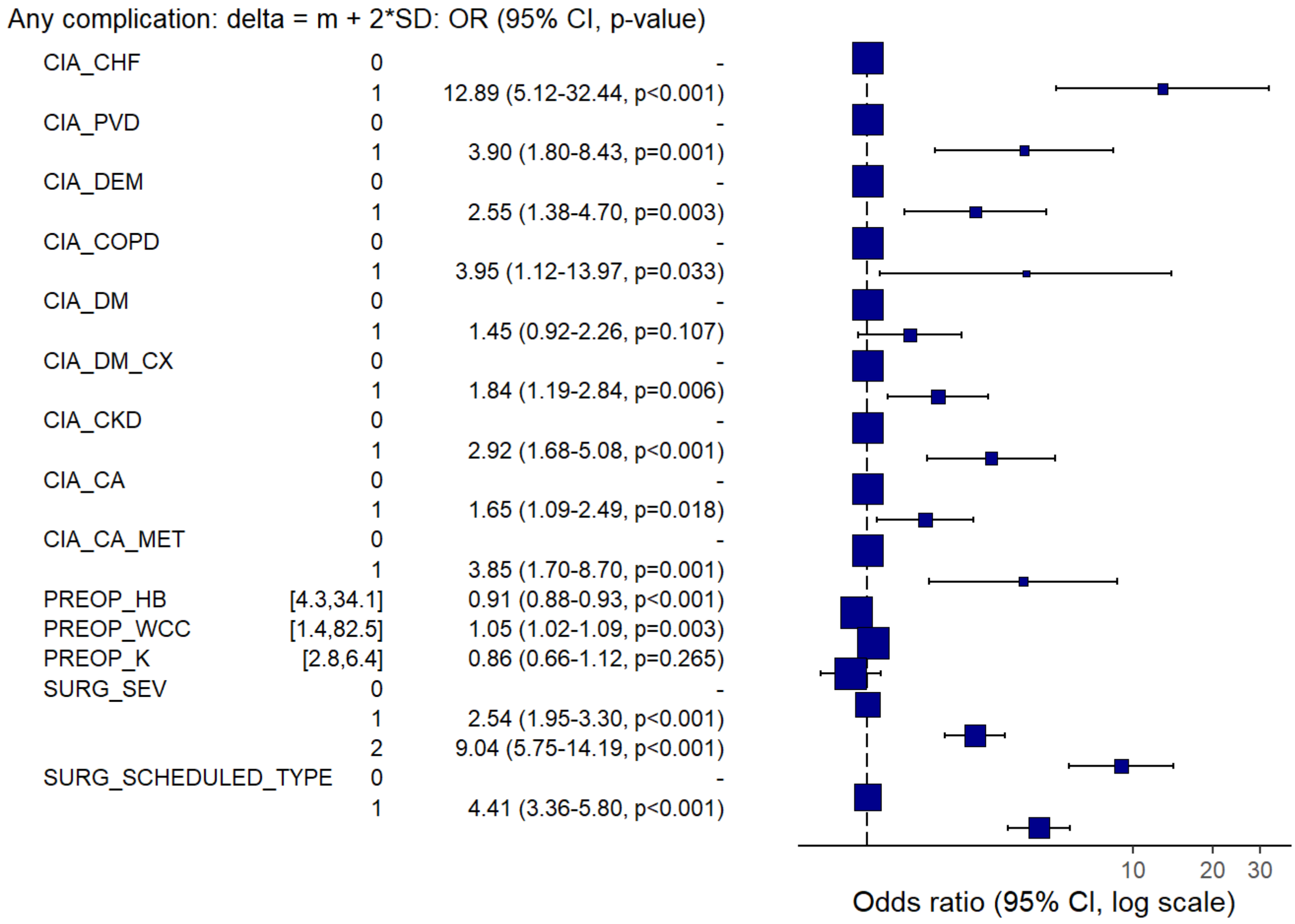

6.4 Model testing

We performed the model testing using the testing dataset for original and pooled imputed models.
Model testing has three components.
First, model discrimination ability using ROC curve analysis.
Second, regarding the threshold estimated from the training dataset, ROC analysis on testing dataset. This process assess the ability of the clinical dichromotous decision making such as “Yes” or “No” risk of complications.
Third, calibrations measurement as usual.

6.4.1 Original model testing

```
# Remove NA
test.org.na <- na.omit(test.org)

# Predicted probability and classification
lranycx.org.test.pred <- predict(lranycx.org.final
                                  , newdata = test.org.na
                                  , type = "response")
lranycx.org.test.class <- ifelse(lranycx.org.test.pred>=0.5, 1, 0)

# Confusion matrix
cl_table.lranycx.org.test <- table (test.org.na$Complication10, lranycx.org.test.class)
rownames(cl_table.lranycx.org.test) <- c("No Cx", "Complicated")
colnames(cl_table.lranycx.org.test) <- c("No Cx", "Complicated")
addmargins(cl_table.lranycx.org.test)
```

```
##              lranycx.org.test.class
##               No Cx Complicated Sum
##   No Cx         238          33 271
##   Complicated    47          98 145
##   Sum           285         131 416
```

```
# Model accuracy (classification accuracy)
mean((test.org.na$Complication10 == lranycx.org.test.class))
```

```
## [1] 0.8076923
```

```
# Discriminability
roc.lranycx.org.test <- roc(test.org.na$Complication10
                          ,lranycx.org.test.pred
                          , add = TRUE)
```

```
## Setting levels: control = 0, case = 1
```

```
## Setting direction: controls < cases
```

```
roc.lranycx.org.test$auc
```

```
## Area under the curve: 0.871
```

```
ci(roc.lranycx.org.test)
```

```
## 95% CI: 0.8351-0.9069 (DeLong)
```

```
plot.roc(roc.lranycx.org.test
         , print.auc = TRUE
         , print.thres = FALSE
         , auc.polygon = TRUE
)
```

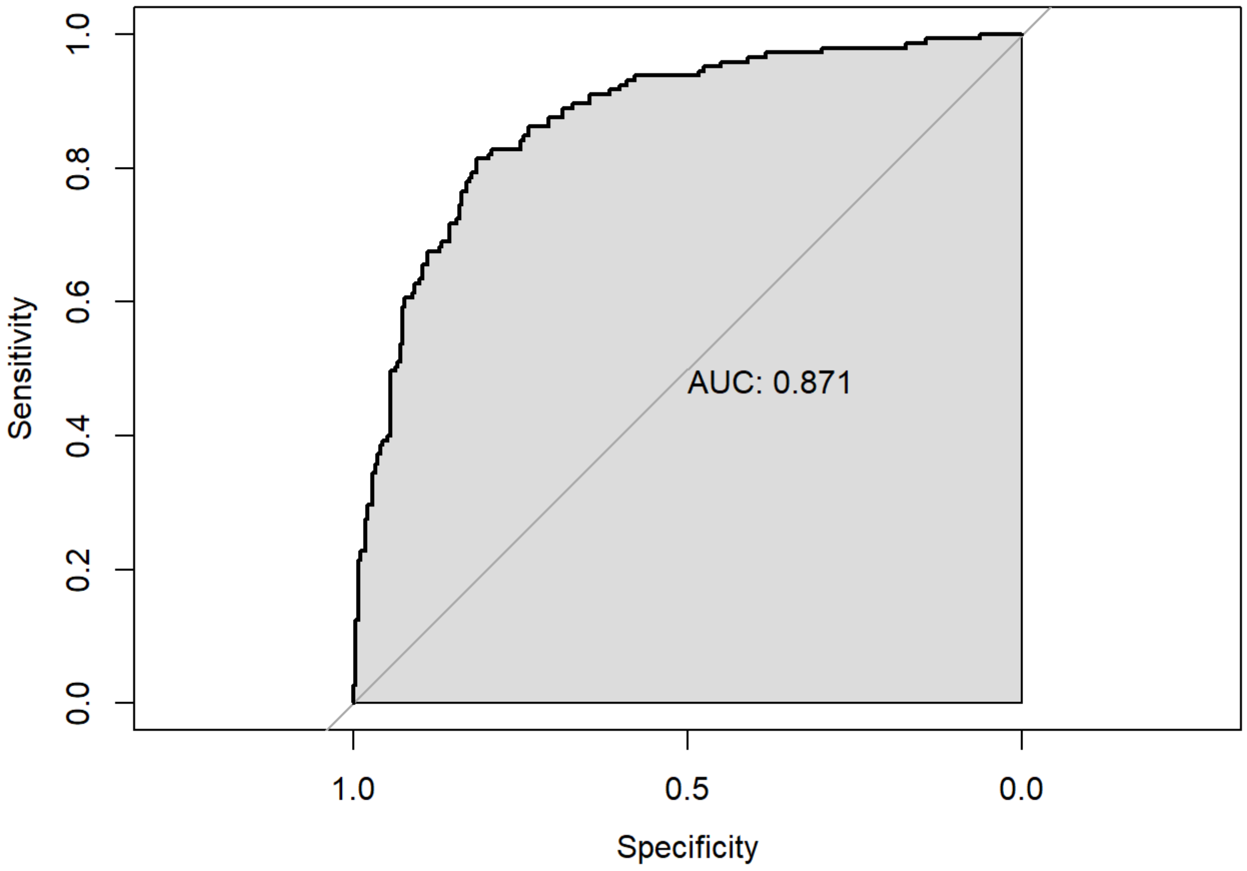


```
pROC::coords(roc.lranycx.org.test,
             "best",
             transpose = TRUE,
             best.method = "youden")
```

```
##   threshold specificity sensitivity
##   0.3158899   0.8154982   0.8137931
```

```
#Threshold discrimination ability

lranycx.org.test.pred.dcs <- ifelse(lranycx.org.test.pred > 0.3226489, 1, 0)

roc.lranycx.org.test.dcs <- roc(test.org.na$Complication10
                          ,lranycx.org.test.pred.dcs
                          , add = TRUE)
```

```
## Setting levels: control = 0, case = 1
```

```
## Setting direction: controls < cases
```

```
roc.lranycx.org.test.dcs$auc
```

```
## Area under the curve: 0.8112
```

```
ci(roc.lranycx.org.test.dcs)
```

```
## 95% CI: 0.7715-0.8509 (DeLong)
```

```
plot.roc(roc.lranycx.org.test.dcs
         , print.auc = TRUE
         , print.thres = FALSE
         , auc.polygon = TRUE
)
```

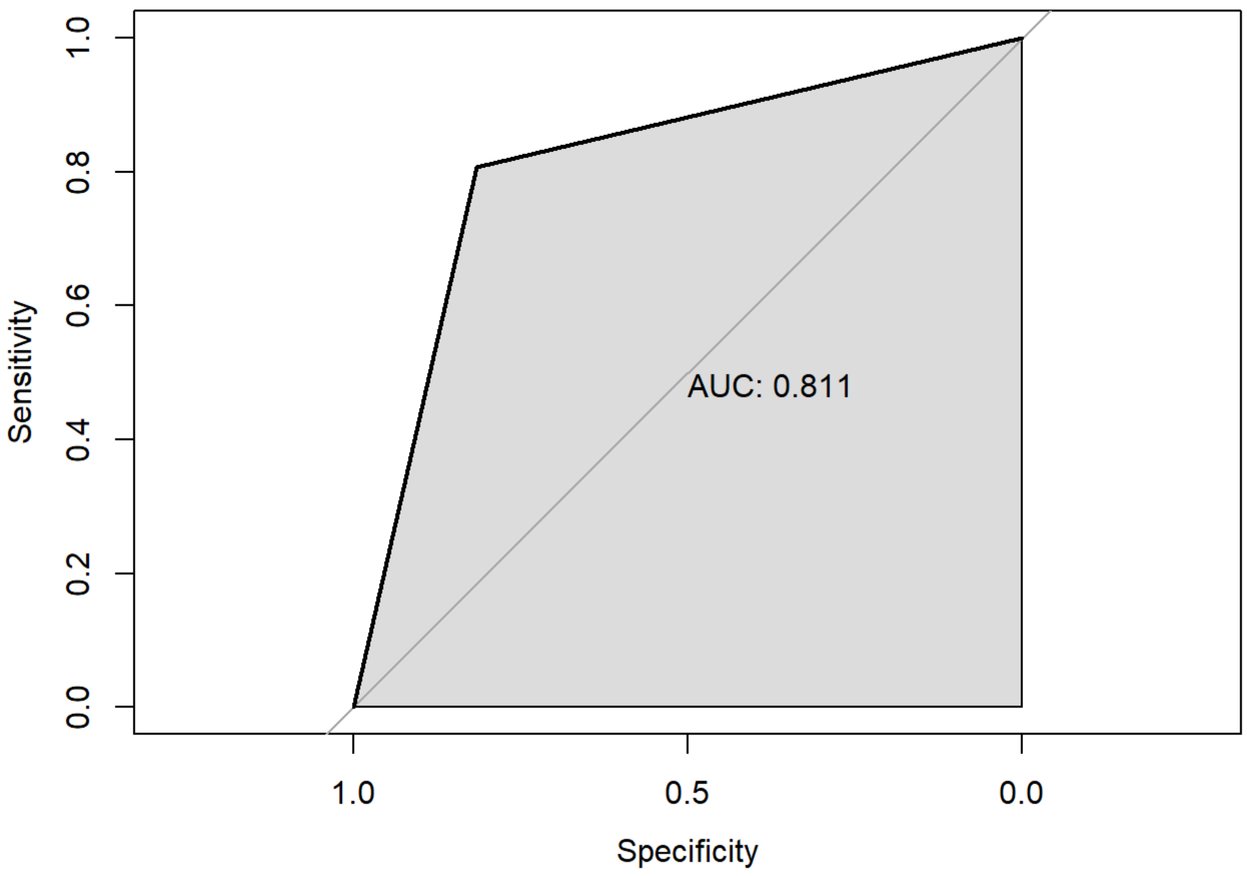


```
pROC::coords(roc.lranycx.org.test.dcs,
             "best",
             transpose = TRUE,
             best.method = "youden")
```

```
##   threshold specificity sensitivity
##   0.5000000   0.8154982   0.8068966
```

6.4.2 Imputed model Testing

6.4.2.1 Testing with the imputted result.

```
# Predicted probability and classification: pooled model
lranycx.imp.test.pred <- as.data.frame(predict(lranycx.imp, test.org.na, type = "response"))
lranycx.imp.test.class <- ifelse(lranycx.imp.test.pred>=0.5, 1, 0)

# Confusion matrix
cl_table.lranycx.imp.test <- table (test.org.na$Complication10, lranycx.imp.test.class)
rownames(cl_table.lranycx.imp.test) <- c("No Cx", "Complicated")
colnames(cl_table.lranycx.imp.test) <- c("No Cx", "Complicated")
addmargins(cl_table.lranycx.imp.test)
```

```
##              lranycx.imp.test.class
##               No Cx Complicated Sum
##   No Cx         240          31 271
##   Complicated    53          92 145
##   Sum           293         123 416
```

```
# Sensitivity
round(cl_table.lranycx.imp.test[2,2]/(cl_table.lranycx.imp.test[2,1] + cl_table.lranycx.imp.test[2,
2]),3)
```

```
## [1] 0.634
```

```
# Specificity
round(cl_table.lranycx.imp.test[1,1]/(cl_table.lranycx.imp.test[1,1] + cl_table.lranycx.imp.test[1,
2]),3)
```

```
## [1] 0.886
```

```
# Positive predictive rate
round(cl_table.lranycx.imp.test[2,2]/(cl_table.lranycx.imp.test[1,2] + cl_table.lranycx.imp.test[2,
2]),3)
```

```
## [1] 0.748
```

```
# Negative predictive rate
round(cl_table.lranycx.imp.test[1,1]/(cl_table.lranycx.imp.test[1,1] + cl_table.lranycx.imp.test[2,
1]),3)
```

```
## [1] 0.819
```

```
#model accuracy (classification accuracy)
mean((test.org.na$CVD_23 == lranycx.imp.test.class))
```

```
## [1] 0.7932692
```

```
#Discriminability

roc.lranycx.imp.test <- roc(test.org.na$Complication10
                            , lranycx.imp.test.pred[,1]
                            , add = TRUE)
```

```
## Setting levels: control = 0, case = 1
```

```
## Setting direction: controls < cases
```

```
roc.lranycx.imp.test$auc
```

```
## Area under the curve: 0.8721
```

```
ci(roc.lranycx.imp.test)
```

```
## 95% CI: 0.8366-0.9076 (DeLong)
```

```
pROC::coords(roc.lranycx.imp.test
       , "best"
       , transpose = TRUE
       , best.method="youden"
       )
```

```
##   threshold specificity sensitivity
##   0.2804979   0.8044280   0.8275862
```

```
plot.roc(roc.lranycx.imp.test
         , print.auc = TRUE
         , print.thres = FALSE
         , auc.polygon = TRUE
)
```

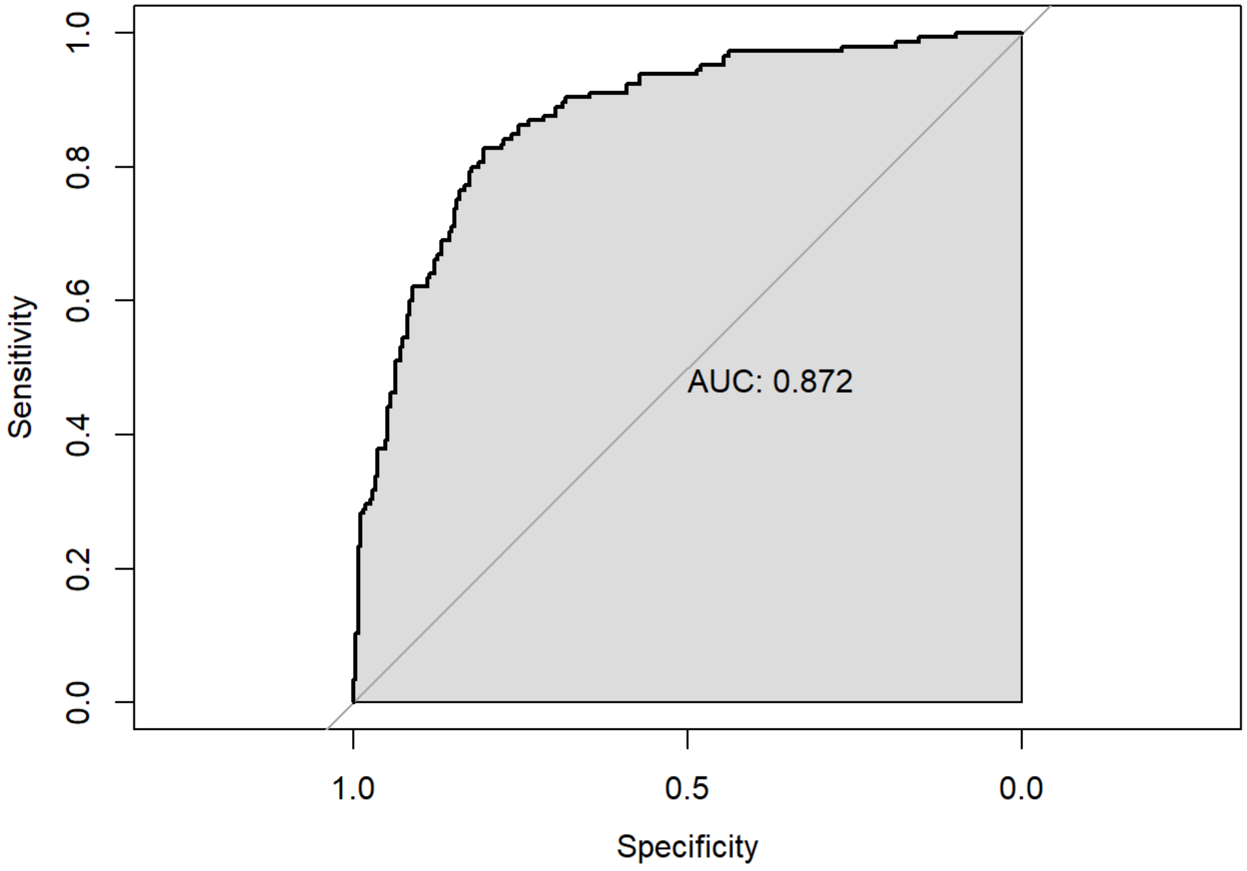


```
# Threshold discrimination ability

lranycx.imp.test.pred.dcs <- ifelse(lranycx.imp.test.pred[,1] > 0.3226489, 1, 0)

roc.lranycx.imp.test.dcs <- roc(test.org.na$Complication10
                          ,lranycx.imp.test.pred.dcs
                          , add = TRUE)
```

```
## Setting levels: control = 0, case = 1
## Setting direction: controls < cases
```

```
roc.lranycx.imp.test.dcs$auc
```

```
## Area under the curve: 0.808
```

```
ci(roc.lranycx.imp.test.dcs)
```

```
## 95% CI: 0.7678-0.8482 (DeLong)
```

```
plot.roc(roc.lranycx.imp.test.dcs
         , print.auc = TRUE
         , print.thres = FALSE
         , auc.polygon = TRUE
)
```

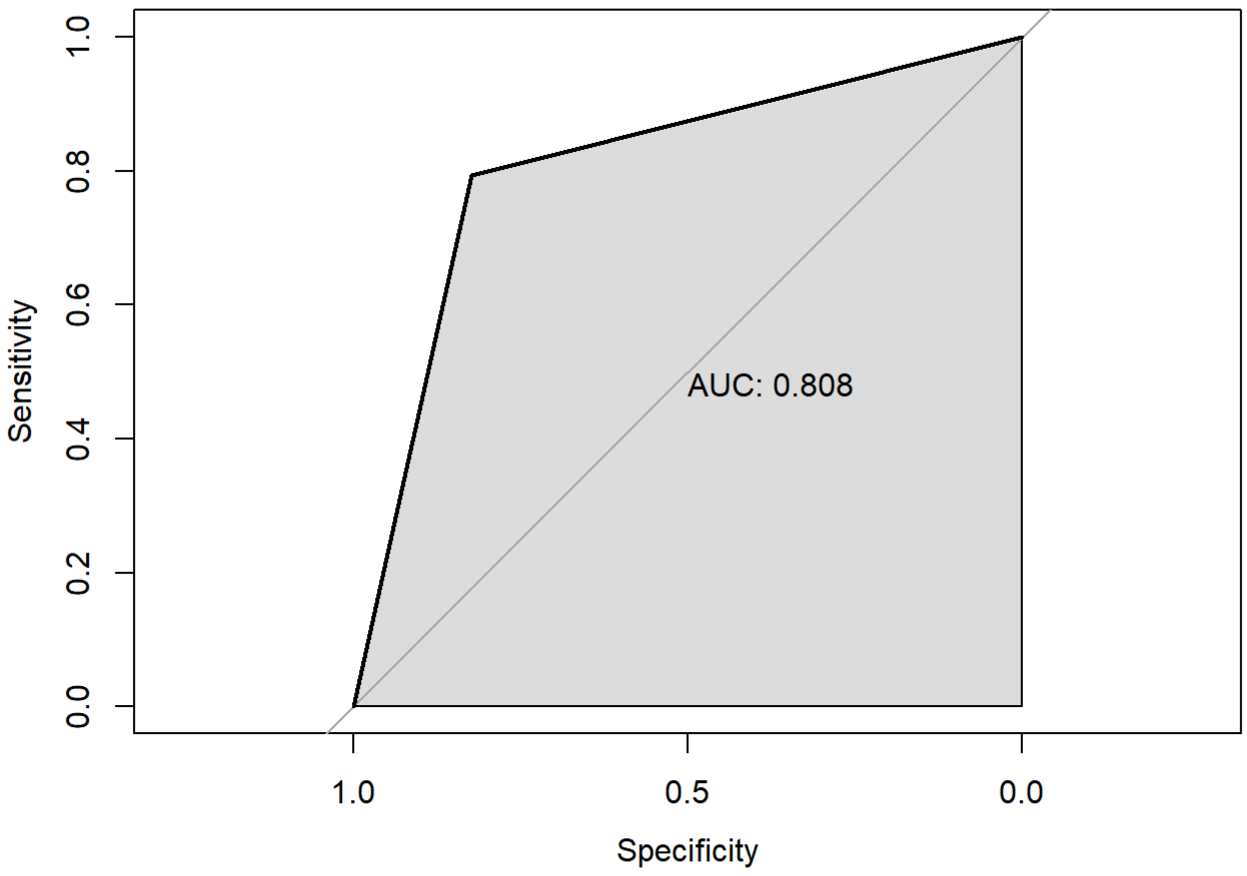


```
pROC::coords(roc.lranycx.imp.test.dcs,
             "best",
             transpose = TRUE,
             best.method = "youden")
```

```
##   threshold specificity sensitivity
##   0.5000000   0.8228782   0.7931034
```

```r
# Calibrations: on the testing dataset
cal.lranycx.imp.test <- val.prob(lranycx.imp.test.pred[,1]
                       , test.org.na$Complication10
                       , pl = FALSE)

cal.lranycx.imp.test
```

```
##           Dxy       C (ROC)            R2             D      D:Chi-sq
##   0.744089579   0.872044789   0.485822226   0.432237709 180.810886858
##           D:p             U      U:Chi-sq           U:p             Q
##            NA  -0.001658056   1.310248730   0.519377466   0.433895765
##         Brier     Intercept         Slope          Emax           E90
##   0.137117303   0.161434197   1.080188690   0.097133782   0.077102611
##          Eavg           S:z           S:p
##   0.034430646  -0.266957762   0.789501683
```

```r
hosmer_lemeshow(test.org.na$Complication10, lranycx.imp.test.pred[,1], 10, 'C')
```

```
## PVALUE 0.326506
## stat 9.1902
```

```
## [1] 0.326506
```

```r
hosmer_lemeshow(test.org.na$Complication10, lranycx.imp.test.pred[,1], 10, 'H')
```

```
## PVALUE 0.1990415
## stat 11.04716
```

```
## [1] 0.1990415
```

Calibration interpretations & decision of re-calibration requirement

Brier score: 0.137 <<1
Spiegelhalter Z-test P = 0.7895: Fail to reject the null hypothesis
Hosmer-Lemeshow C & H test P values = 0.327 & 0.199 > 0.100: appropriate

Regarding the estimated calibrations, there is no need of re-calibration.
However, I try out the re-calibration with testing dataset to see how it works and to gain experience.

6.5 Recalibrations using testing dataset

Recalibration: Isotonic regression model

```
# predicted probability testing dataset with pooled logistic regression
lranycx.imp.test.pred.prob <- predict(lranycx.imp,
                                      test.org.na[,-length(test.org.na)],
                                      type = "response")
lranycx.imp.iso.data = cbind(y=test.org.na$Complication10
                         , yhat=lranycx.imp.test.pred.prob)
lranycx.imp.iso.data.order = lranycx.imp.iso.data[order(lranycx.imp.iso.data[,2]),]

# create calibration model: isoanycx.imp
isoanycx.imp.wostepfunc <- isoreg(lranycx.imp.iso.data.order[,2], lranycx.imp.iso.data.order[,1])
isoanycx.imp.stepf_data = cbind(isoanycx.imp.wostepfunc$x, isoanycx.imp.wostepfunc$yf)
isoanycx.imp = stepfun(isoanycx.imp.stepf_data[,1], c(0,isoanycx.imp.stepf_data[,2]))

# Predicted probability of isotonic regression on testing dataset. Imputed model

isoanycx.imp.pred <- isoanycx.imp(as.data.frame(lranycx.imp.test.pred.prob)[,1])
```

Recalibration: Platt scailing

```
pltanycx.imp <- glm(y~yhat, as.data.frame(lranycx.imp.iso.data), family=binomial)
  ygrid_norm = as.data.frame(lranycx.imp.test.pred)
  colnames(lranycx.imp.test.pred) <- c("yhat")


  # Predicted probability of platt scailing
pltanycx.imp.pred <- as.data.frame(predict(pltanycx.imp, lranycx.imp.test.pred, type = "response" ))
```

6.5.1 Discriminations & Calibration measurements of recalibrated models

6.5.1.1 Discrimination

```
# Discrimination ability: Isoregression model

roc.isoanycx.test.imp <- roc(test.org.na$Complication10
                             , isoanycx.imp.pred
                        , add = TRUE)
```

```
## Setting levels: control = 0, case = 1
```

```
## Setting direction: controls < cases
```

```
roc.isoanycx.test.imp$auc
```

```
## Area under the curve: 0.8839
```

```
ci(roc.isoanycx.test.imp)
```

```
## 95% CI: 0.8512-0.9166 (DeLong)
```

```
pROC::coords(roc.isoanycx.test.imp
       , "best"
       , transpose = TRUE
       , best.method="youden"
       )
```

```
##   threshold specificity sensitivity
##   0.3538012   0.8044280   0.8275862
```

```
plot.roc(roc.isoanycx.test.imp
         , print.auc = TRUE
         , print.thres = FALSE
         , auc.polygon = TRUE
)
```

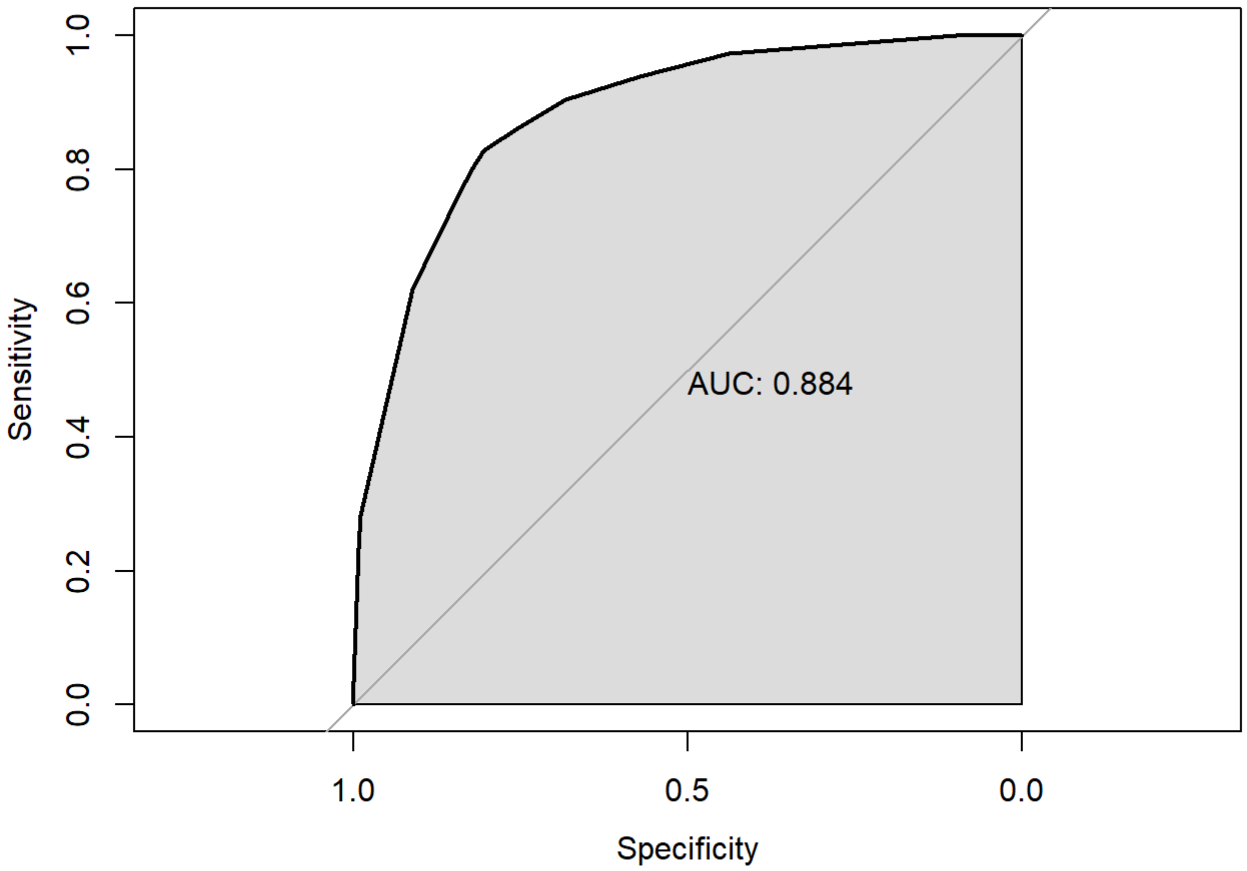


```
# Discrimination ability: Platt Scaling model
roc.pltanycx.test.imp <- roc(test.org.na$Complication10
                             , pltanycx.imp.pred[,1]
                      , add = TRUE)
```

```
## Setting levels: control = 0, case = 1
## Setting direction: controls < cases
```

```
roc.pltanycx.test.imp$auc
```

```
## Area under the curve: 0.8721
```

```
ci(roc.pltanycx.test.imp)
```

```
## 95% CI: 0.8366-0.9076 (DeLong)
```

```
pROC::coords(roc.pltanycx.test.imp
       , "best"
       , transpose = TRUE
       , best.method="youden"
       )
```

```
##   threshold specificity sensitivity
##   0.2486225   0.8044280   0.8275862
```

```
plot.roc(roc.pltanycx.test.imp
         , print.auc = TRUE
         , print.thres = FALSE
         , auc.polygon = TRUE
)
```

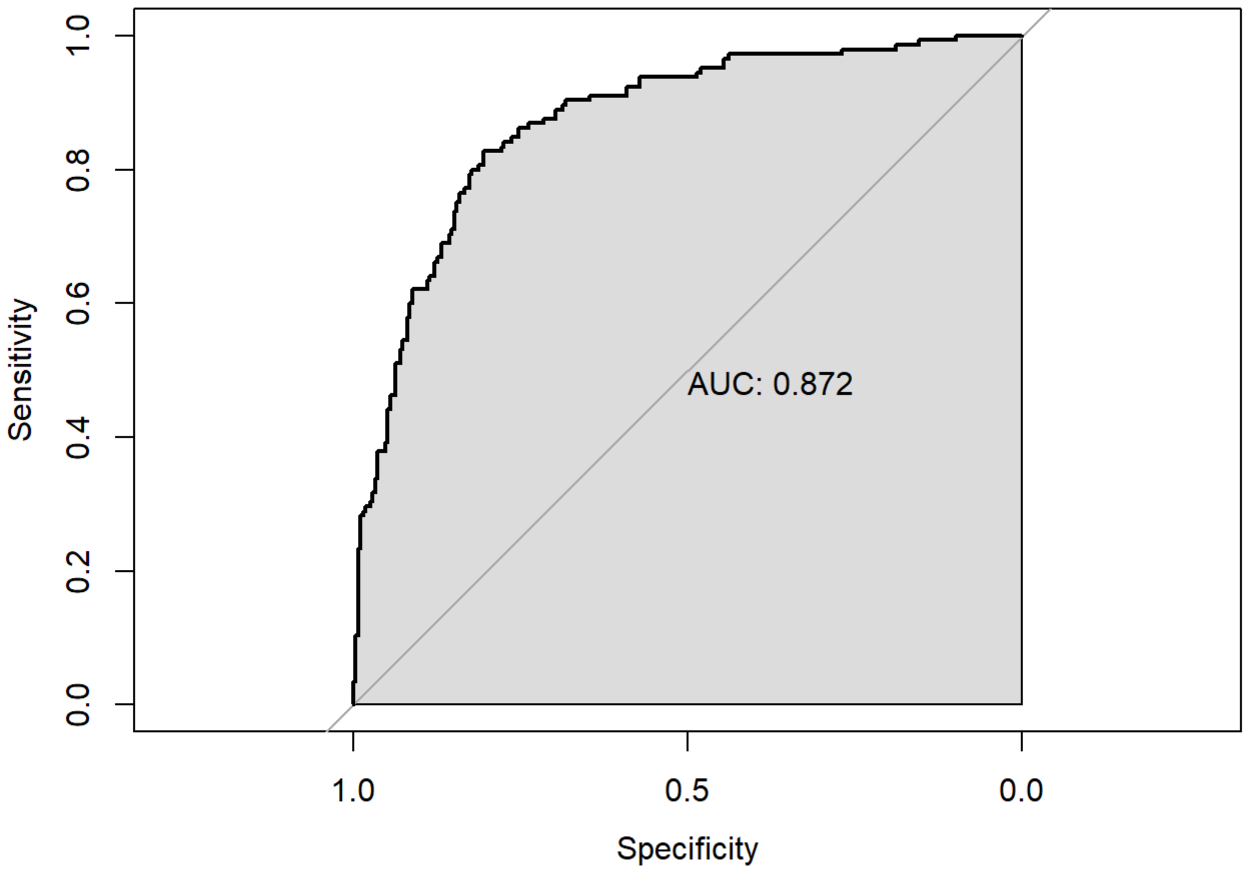

6.5.2 Threshold discrimination ability: Treshold from lrmodel with training data

```
# lsoregression model

isoanycx.imp.test.pred.dcs <- ifelse(isoanycx.imp.pred > 0.3226489, 1, 0)

roc.isoanycx.imp.test.dcs <- roc(test.org.na$Complication10
                         ,isoanycx.imp.test.pred.dcs
                         , add = TRUE)
```

```
## Setting levels: control = 0, case = 1
```

```
## Setting direction: controls < cases
```

```
roc.isoanycx.imp.test.dcs$auc
```

```
## Area under the curve: 0.816
```

```
ci(roc.isoanycx.imp.test.dcs)
```

```
## 95% CI: 0.7771-0.8549 (DeLong)
```

```
plot.roc(roc.isoanycx.imp.test.dcs
         , print.auc = TRUE
         , print.thres = FALSE
         , auc.polygon = TRUE
)
```

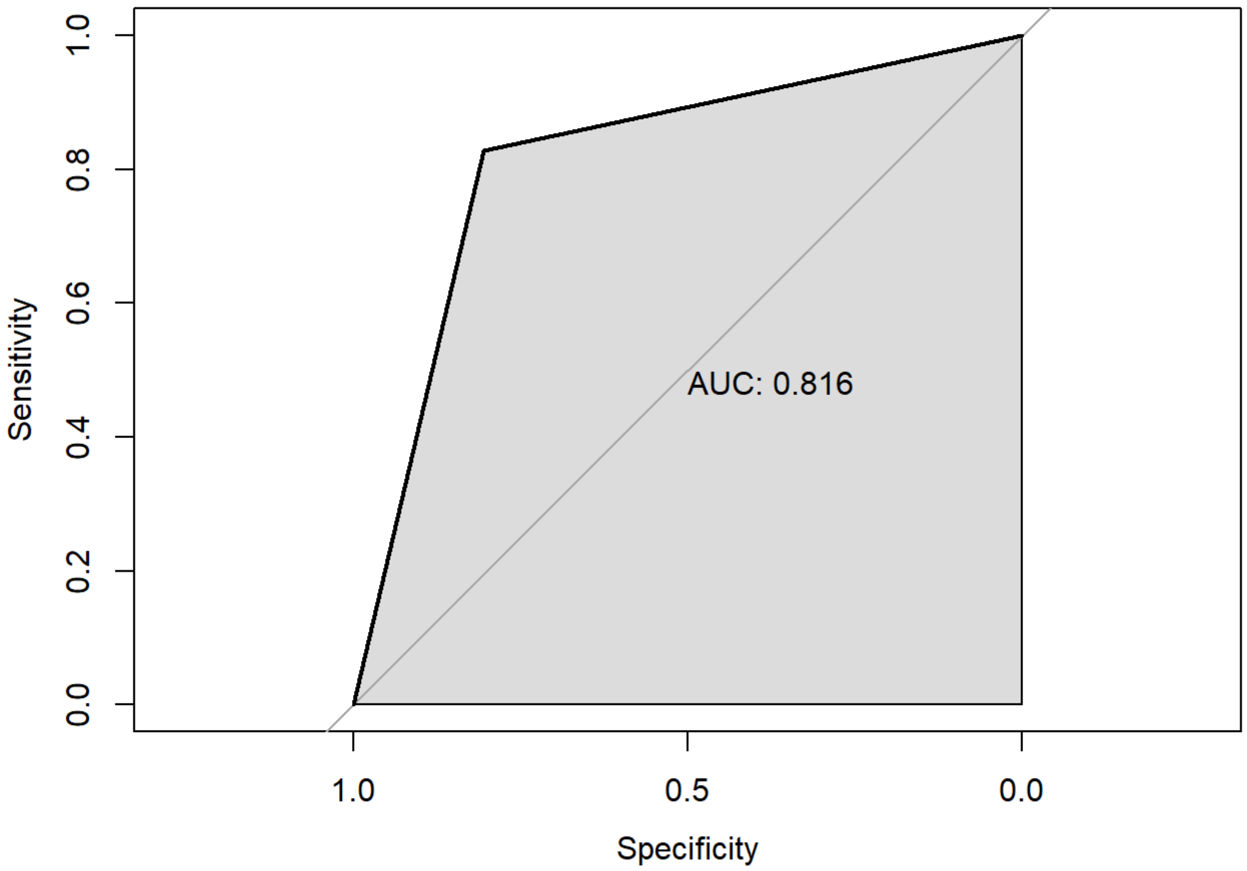


```
pROC::coords(roc.isoanycx.imp.test.dcs,
             "best",
             transpose = TRUE,
             best.method = "youden")
```

```
##   threshold specificity sensitivity
##   0.5000000   0.8044280   0.8275862
```

```
# Platt Scailing model


pltanycx.imp.test.pred.dcs <- ifelse(pltanycx.imp.pred > 0.3226489, 1, 0)

roc.pltanycx.imp.test.dcs <- roc(test.org.na$Complication10
                         ,pltanycx.imp.test.pred.dcs
                         , add = TRUE)
```

```
## Setting levels: control = 0, case = 1
## Setting direction: controls < cases
```

```
roc.pltanycx.imp.test.dcs$auc
```

```
## Area under the curve: 0.7997
```

```
ci(roc.pltanycx.imp.test.dcs)
```

```
## 95% CI: 0.7586-0.8408 (DeLong)
```

```
plot.roc(roc.pltanycx.imp.test.dcs
         , print.auc = TRUE
         , print.thres = FALSE
         , auc.polygon = TRUE
)
```

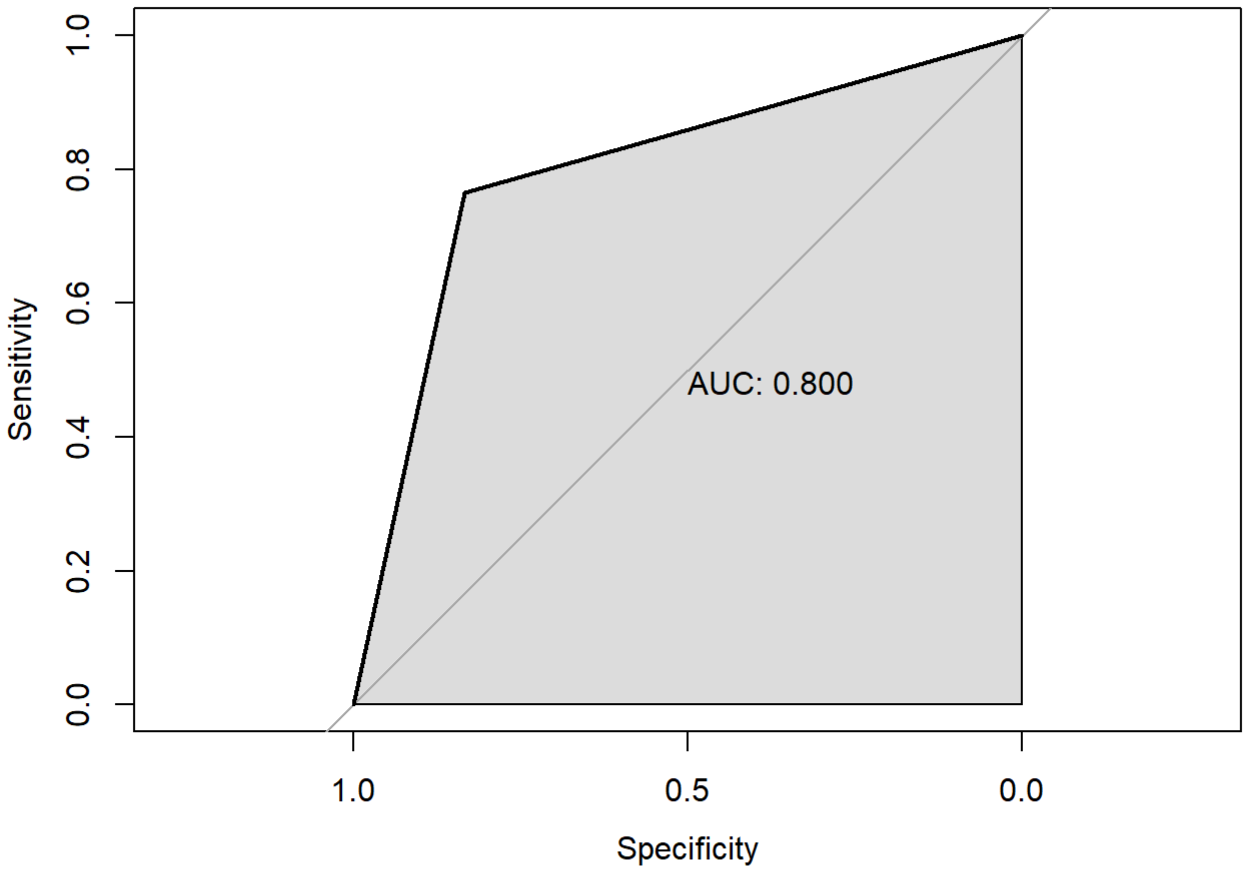

```
pROC::coords(roc.pltanycx.imp.test.dcs,
             "best",
             transpose = TRUE,
             best.method = "youden")
```

```
##   threshold specificity sensitivity
##   0.5000000   0.8339483   0.7655172
```

6.5.3 Calibrations of recalibrated models

```
cal.isoanycx.imp.test <- val.prob(isoanycx.imp.pred
                        , test.org.na$Complication10
                        , pl = FALSE)



cal.pltanycx.imp.test <- val.prob(pltanycx.imp.pred[,1]
                        , test.org.na$Complication10
                        , pl = FALSE)



cbind(cal.lranycx.imp.test, cal.isoanycx.imp.test, cal.pltanycx.imp.test)
```

```
##           cal.lranycx.imp.test cal.isoanycx.imp.test cal.pltanycx.imp.test
## Dxy                0.744089579          7.328162e-01          7.441405e-01
## C (ROC)            0.872044789          8.664081e-01          8.720702e-01
## R2                 0.485822226          4.987083e-01          4.783520e-01
## D                  0.432237709          4.506105e-01          4.239015e-01
## D:Chi-sq         180.810886858          1.740344e+02          1.773430e+02
## D:p                         NA                    NA                    NA
## U                 -0.001658056         -5.208333e-03         -4.807692e-03
## U:Chi-sq           1.310248730          2.842171e-13          1.136868e-13
## U:p                0.519377466          1.000000e+00          1.000000e+00
## Q                  0.433895765          4.558188e-01          4.287092e-01
## Brier              0.137117303          1.271540e-01          1.381670e-01
## Intercept          0.161434197          8.634518e-11          9.915802e-11
## Slope              1.080188690          1.000000e+00          1.000000e+00
## Emax               0.097133782          1.054712e-14          1.650091e-01
## E90                0.077102611          1.776357e-15          8.730744e-02
## Eavg               0.034430646          1.397832e-15          4.524229e-02
## S:z               -0.266957762         -9.544020e-17          2.313248e-01
## S:p                0.789501683          1.000000e+00          8.170625e-01
```

```
hosmer_lemeshow(test.org.na$Complication10, lranycx.imp.test.pred[,1], 10, 'C')
```

```
## PVALUE 0.326506
## stat 9.1902
```

```
## [1] 0.326506
```

```
hosmer_lemeshow(test.org.na$Complication10, isoanycx.imp.pred, 10, 'C')
```

```
## PVALUE 0.9405954
## stat 2.897985
```

```
## [1] 0.9405954
```

```
hosmer_lemeshow(test.org.na$Complication10, pltanycx.imp.pred[,1], 10, 'C')
```

```
## PVALUE 0.1406855
## stat 12.24303
```

```
## [1] 0.1406855
```

```
hosmer_lemeshow(test.org.na$Complication10, lranycx.imp.test.pred[,1], 10, 'H')
```

```
## PVALUE 0.1990415
## stat 11.04716
```

```
## [1] 0.1990415
```

```
hosmer_lemeshow(test.org.na$Complication10, isoanycx.imp.pred, 10, 'H')
```

```
## PVALUE 1
## stat 1.577722e-30
```

```
## [1] 1
```

```
hosmer_lemeshow(test.org.na$Complication10, pltanycx.imp.pred[,1], 10, 'H')
```

```
## PVALUE 0.07765963
## stat 14.16119
```

```
## [1] 0.07765963
```

```
CalibrationCurves::val.prob.ci.2(lranycx.imp.test.pred[,1], test.org.na$Complication10
              , lty.smooth = 2
              , CL.smooth = FALSE
              , col.ideal = "black")
```

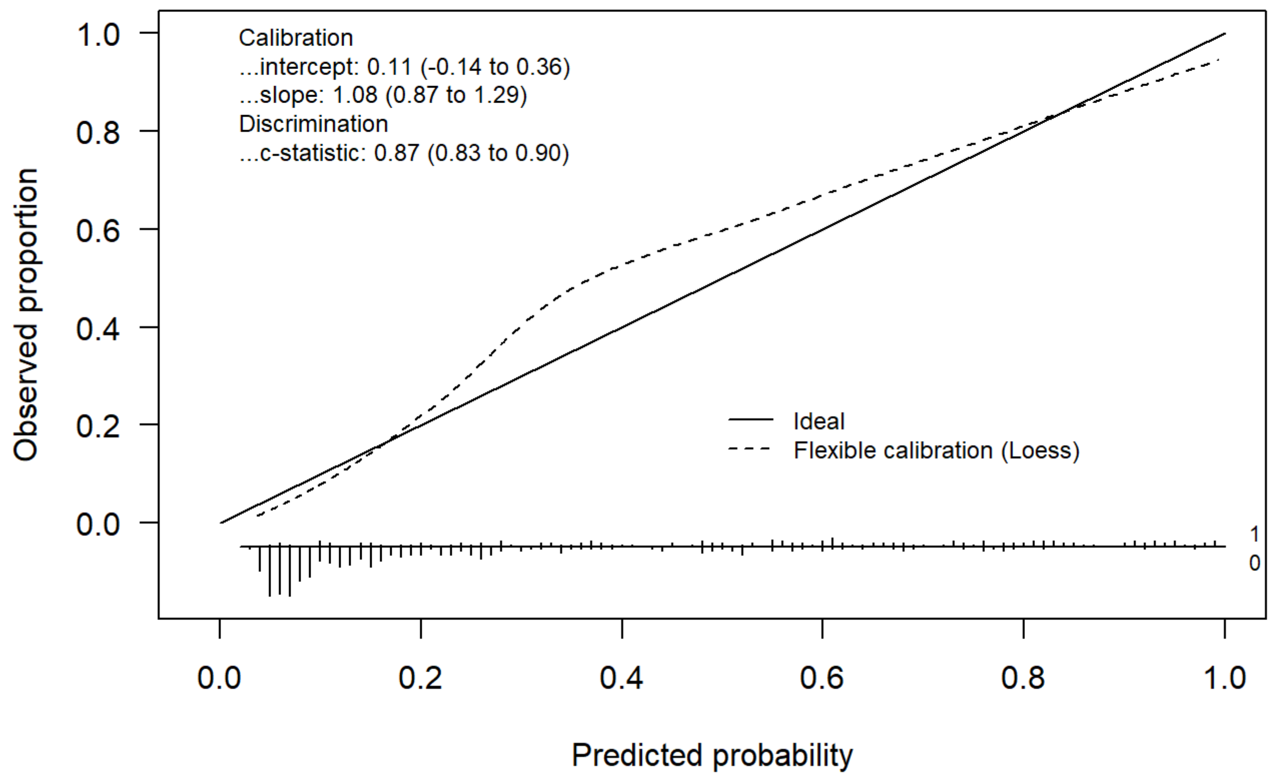


```
CalibrationCurves::val.prob.ci.2(isoanycx.imp.pred, test.org.na$Complication10
                , lty.smooth = 2
                , CL.smooth = FALSE
                , col.ideal = "black")
```

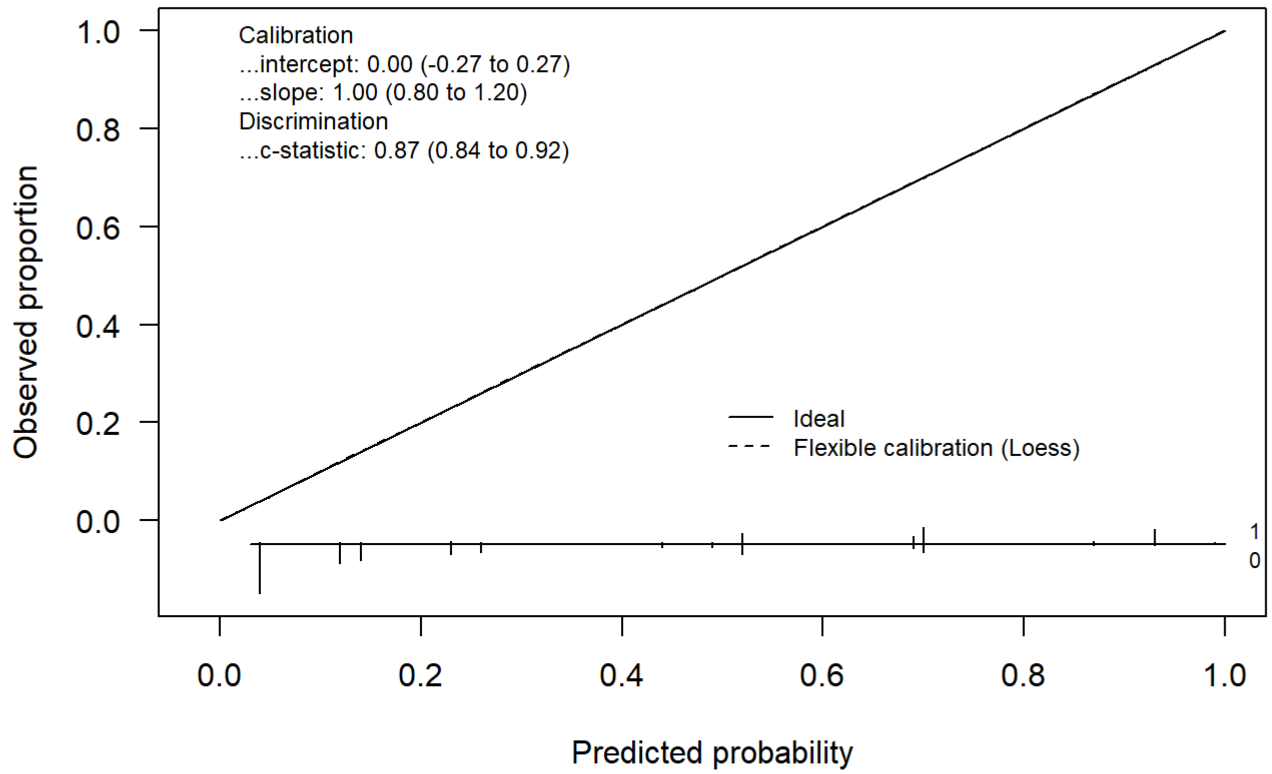

```
CalibrationCurves::val.prob.ci.2(pltanycx.imp.pred[,1], test.org.na$Complication10
              , lty.smooth = 2
              , CL.smooth = FALSE
              , col.ideal = "black")
```

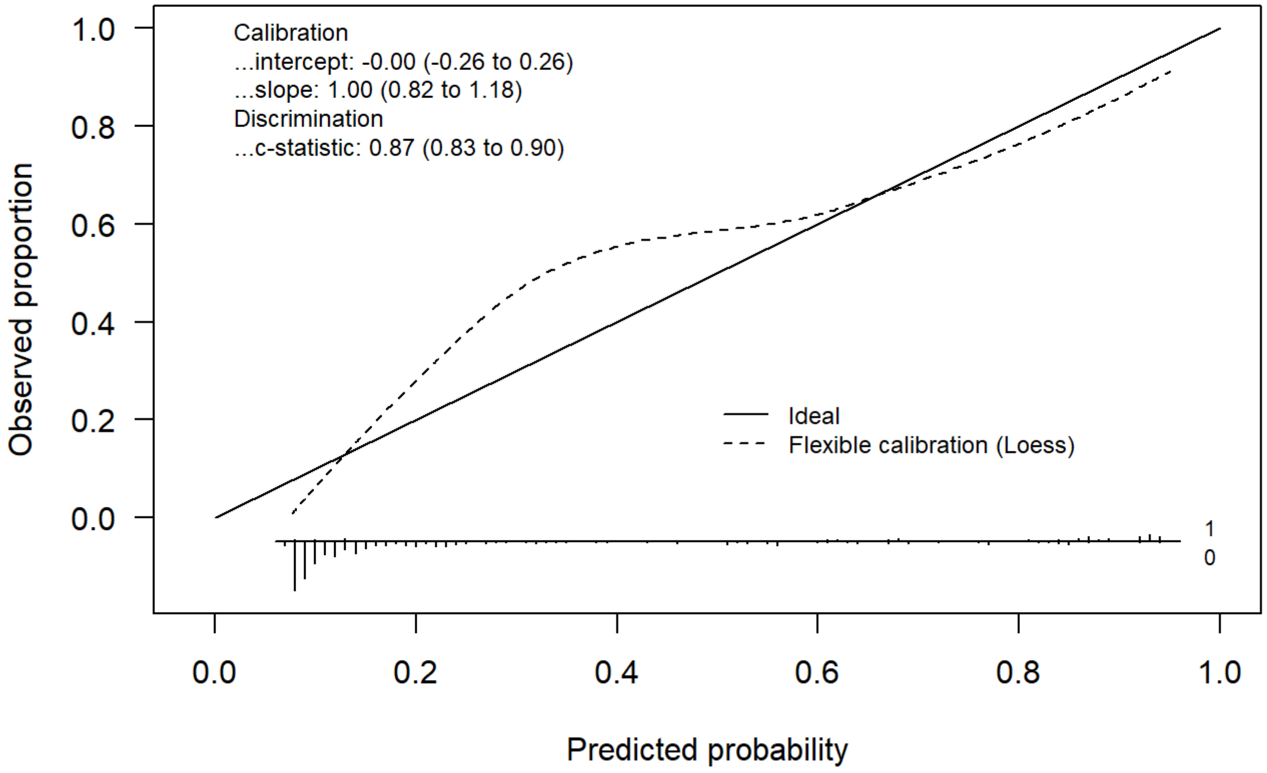

Isoregression model shows perfect fit?!
Going on to the validation.

6.6 Validation

6.6.1 Original data analysis: Validation

```
# Remove NA
valid.org.na <- na.omit(valid.org)

# Predicted probability and classification
lranycx.org.valid.pred <- predict(lranycx.org.final
                                  , newdata = valid.org.na
                                  , type = "response")

lranycx.org.valid.class <- ifelse(lranycx.org.valid.pred>=0.5, 1, 0)

# Confusion matrix
cl_table.lranycx.org.valid <- table (valid.org.na$Complication10, lranycx.org.valid.class)
rownames(cl_table.lranycx.org.valid) <- c("No Cx", "Complicated")
colnames(cl_table.lranycx.org.valid) <- c("No Cx", "Complicated")
addmargins(cl_table.lranycx.org.valid)
```

```
##              lranycx.org.valid.class
##               No Cx Complicated Sum
##   No Cx         267          44 311
##   Complicated    56         131 187
##   Sum           323         175 498
```

```
# Model accuracy (classification accuracy)
mean((valid.org.na$Complication10 == lranycx.org.valid.class))
```

```
## [1] 0.7991968
```

```
# Discriminability
roc.lranycx.org.valid <- roc(valid.org.na$Complication10
                         ,lranycx.org.valid.pred
                         , add = TRUE)
```

```
## Setting levels: control = 0, case = 1
```

```
## Setting direction: controls < cases
```

```
roc.lranycx.org.valid$auc
```

```
## Area under the curve: 0.8598
```

```
ci(roc.lranycx.org.valid)
```

```
## 95% CI: 0.8265-0.893 (DeLong)
```

```
pROC::coords(roc.lranycx.org.valid,
             "best",
             transpose = TRUE,
             best.method = "youden")
```

```
##   threshold specificity sensitivity
##   0.4137994   0.7909968   0.7914439
```

```
plot.roc(roc.lranycx.org.valid
         , print.auc = TRUE
         , print.thres = FALSE
         , auc.polygon = TRUE
)
```

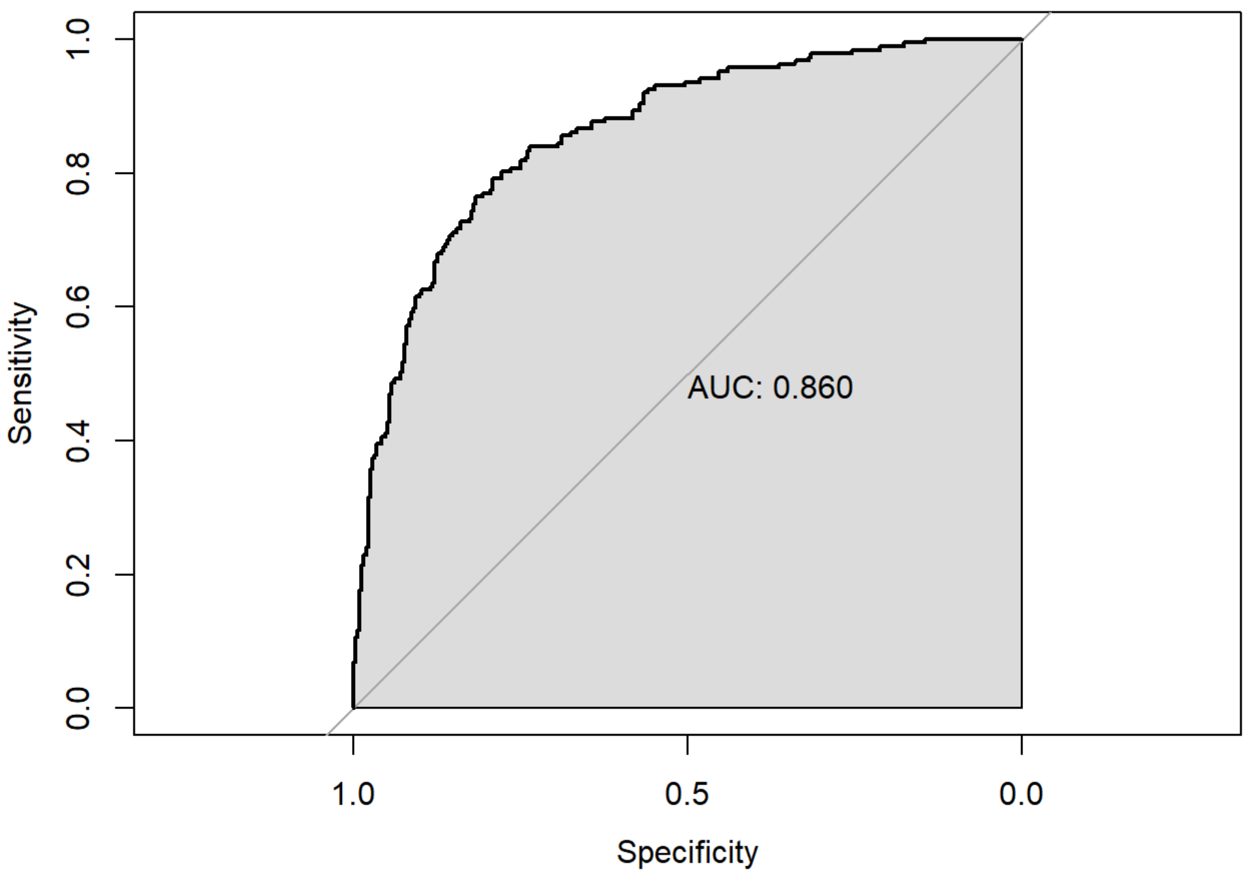


```
#Threshold discrimination ability

lranycx.org.valid.pred.dcs <- ifelse(lranycx.org.valid.pred > 0.3226489, 1, 0)

roc.lranycx.org.valid.dcs <- roc(valid.org.na$Complication10
                         ,lranycx.org.valid.pred.dcs
                         , add = TRUE)
```

```
## Setting levels: control = 0, case = 1
## Setting direction: controls < cases
```

```
roc.lranycx.org.valid.dcs$auc
```

```
## Area under the curve: 0.7863
```

```
ci(roc.lranycx.org.valid.dcs)
```

```
## 95% CI: 0.7503-0.8224 (DeLong)
```

```
pROC::coords(roc.lranycx.org.valid.dcs,
             "best",
             transpose = TRUE,
             best.method = "youden")
```

```
##   threshold specificity sensitivity
##   0.5000000   0.7331190   0.8395722
```

```
plot.roc(roc.lranycx.org.valid.dcs
         , print.auc = TRUE
         , print.thres = FALSE
         , auc.polygon = TRUE
)
```

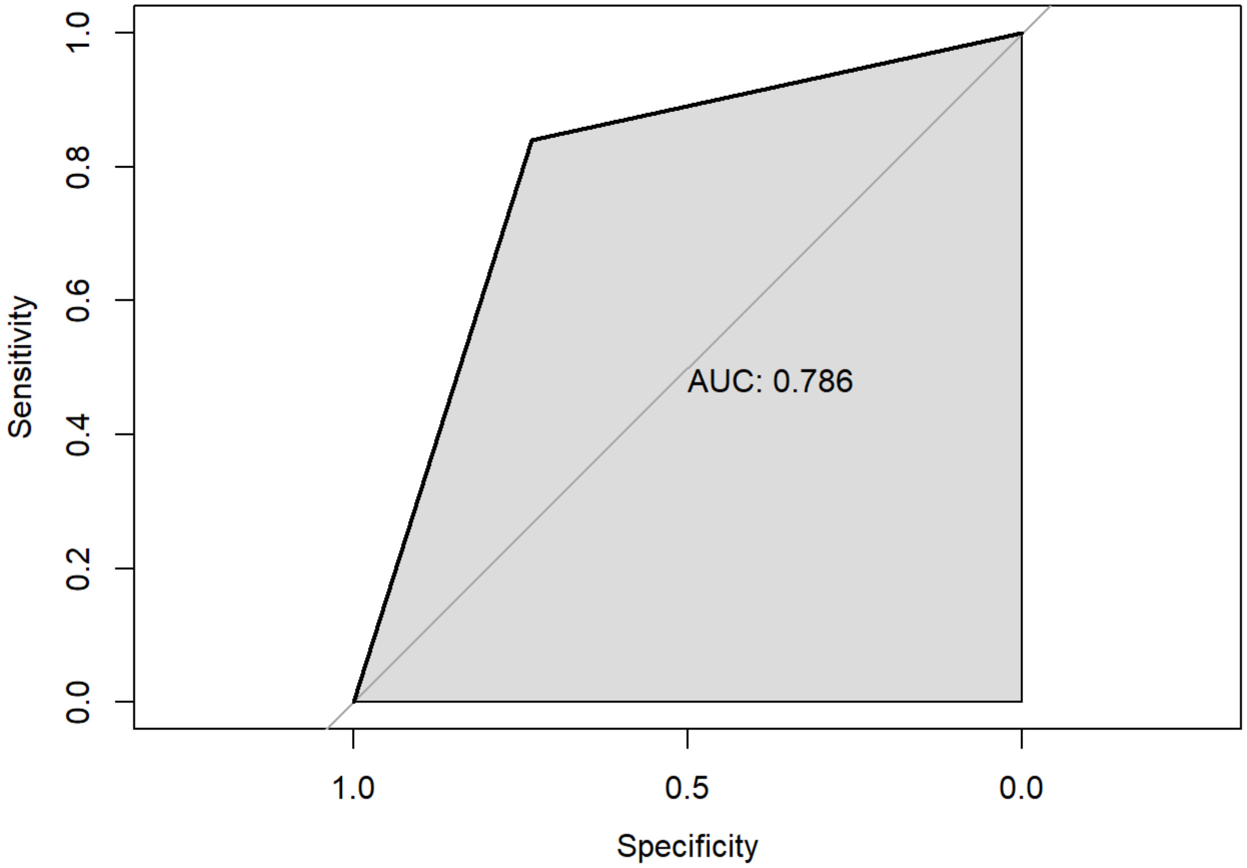

6.6.2 Imputted data analaysis: Validation

```
# Predicted probability and classification: pooled model
lranycx.imp.valid.pred <- as.data.frame(predict(lranycx.imp, valid.org.na, type = "response"))
lranycx.imp.valid.class <- ifelse(lranycx.imp.valid.pred>=0.5, 1, 0)

# Confusion matrix
cl_table.lranycx.imp.valid <- table (valid.org.na$Complication10, lranycx.imp.valid.class)
rownames(cl_table.lranycx.imp.valid) <- c("No Cx", "Complicated")
colnames(cl_table.lranycx.imp.valid) <- c("No Cx", "Complicated")
addmargins(cl_table.lranycx.imp.valid)
```

```
##              lranycx.imp.valid.class
##               No Cx Complicated Sum
##   No Cx         272          39 311
##   Complicated    60         127 187
##   Sum           332         166 498
```

```
# Sensitivity
round(cl_table.lranycx.imp.valid[2,2]/(cl_table.lranycx.imp.valid[2,1] + cl_table.lranycx.imp.valid
[2,2]),3)
```

```
## [1] 0.679
```

```
# Specificity
round(cl_table.lranycx.imp.valid[1,1]/(cl_table.lranycx.imp.valid[1,1] + cl_table.lranycx.imp.valid
[1,2]),3)
```

```
## [1] 0.875
```

```
# Positive predictive rate
round(cl_table.lranycx.imp.valid[2,2]/(cl_table.lranycx.imp.valid[1,2] + cl_table.lranycx.imp.valid
[2,2]),3)
```

```
## [1] 0.765
```

```
# Negative predictive rate
round(cl_table.lranycx.imp.valid[1,1]/(cl_table.lranycx.imp.valid[1,1] + cl_table.lranycx.imp.valid
[2,1]),3)
```

```
## [1] 0.819
```

```
#model accuracy (classification accuracy)
mean((valid.org.na$Complication10 == lranycx.imp.valid.class))
```

```
## [1] 0.8012048
```

```
#Discriminability

roc.lranycx.imp.valid <- roc(valid.org.na$Complication10
                            , lranycx.imp.valid.pred[,1]
                            , add = TRUE)
```

```
## Setting levels: control = 0, case = 1
```

```
## Setting direction: controls < cases
```

```
roc.lranycx.imp.valid$auc
```

```
## Area under the curve: 0.857
```

```
ci(roc.lranycx.imp.valid)
```

```
## 95% CI: 0.8238-0.8903 (DeLong)
```

```
pROC::coords(roc.lranycx.imp.valid
       , "best"
       , transpose = TRUE
       , best.method="youden"
       )
```

```
##   threshold specificity sensitivity
##   0.2866123   0.7363344   0.8395722
```

```
plot.roc(roc.lranycx.imp.valid
         , print.auc = TRUE
         , print.thres = FALSE
         , auc.polygon = TRUE
)
```

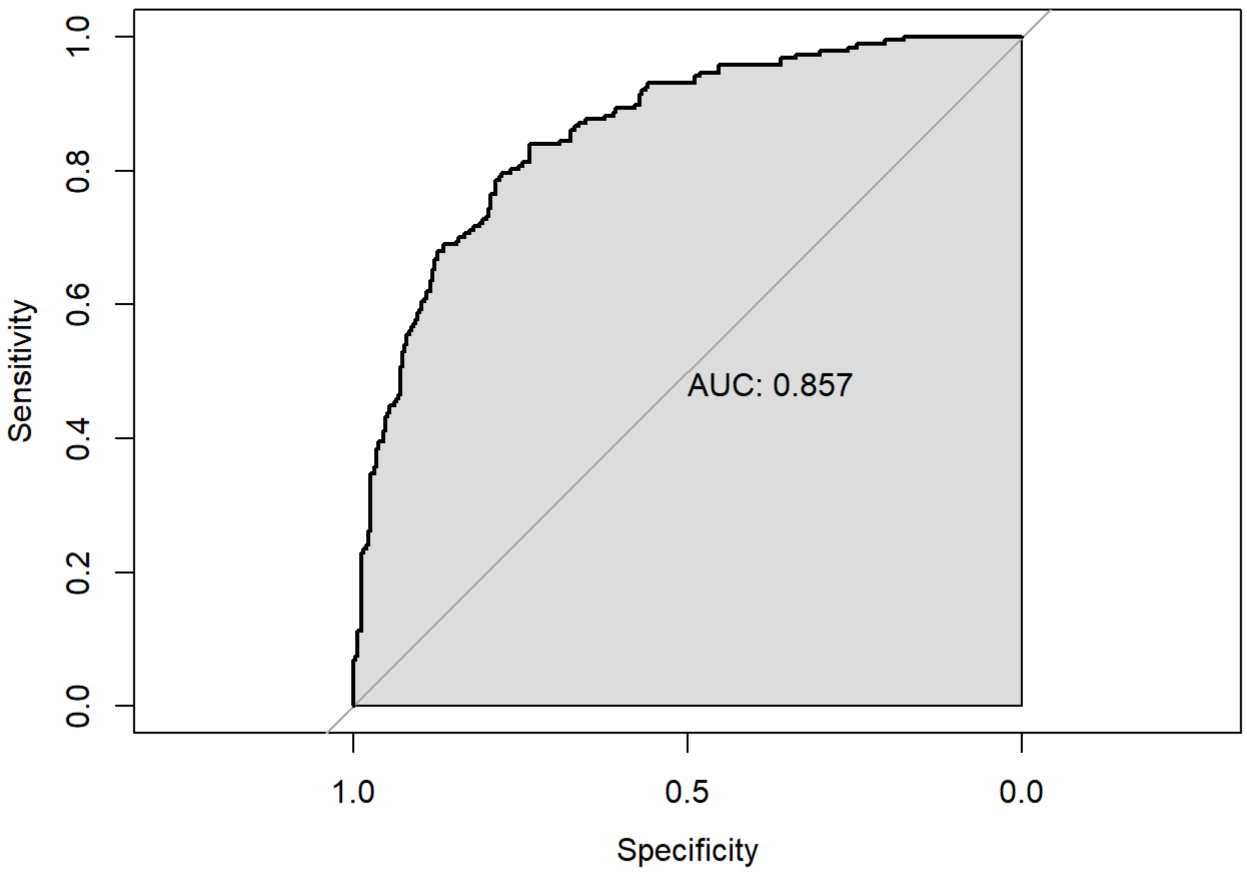


```
# Threshold discrimination ability

lranycx.imp.valid.pred.dcs <- ifelse(lranycx.imp.valid.pred[,1] > 0.3226489, 1, 0)

roc.lranycx.imp.valid.dcs <- roc(valid.org.na$Complication10
                          ,lranycx.imp.valid.pred.dcs
                          , add = TRUE)
```

```
## Setting levels: control = 0, case = 1
## Setting direction: controls < cases
```

```
roc.lranycx.imp.valid.dcs$auc
```

```
## Area under the curve: 0.7773
```

```
ci(roc.lranycx.imp.valid.dcs)
```

```
## 95% CI: 0.7399-0.8146 (DeLong)
```

```
pROC::coords(roc.lranycx.imp.valid.dcs,
             "best",
             transpose = TRUE,
             best.method = "youden")
```

```
##   threshold specificity sensitivity
##   0.5000000   0.7524116   0.8021390
```

```
plot.roc(roc.lranycx.imp.valid.dcs
         , print.auc = TRUE
         , print.thres = FALSE
         , auc.polygon = TRUE
)
```

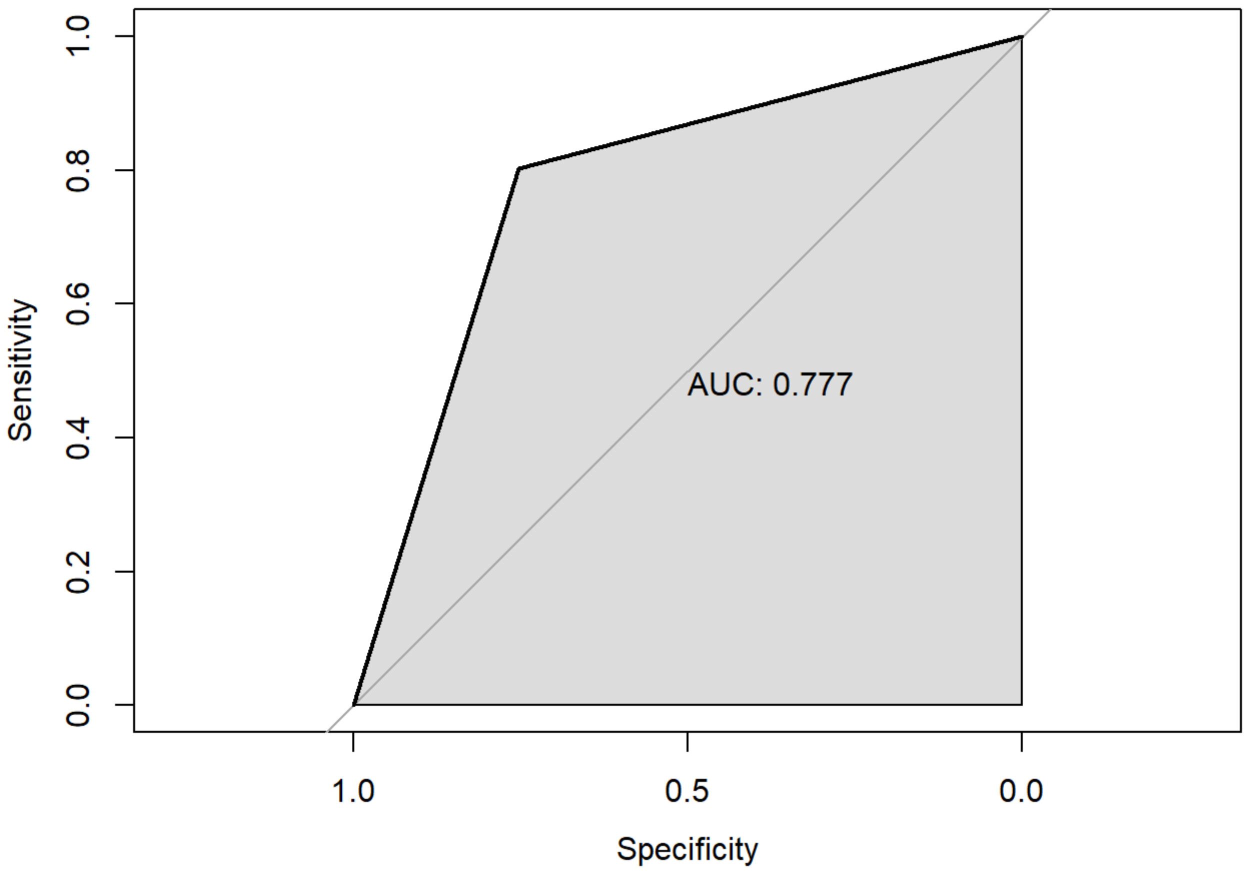


```
# Calibrations: on the validating dataset
cal.lranycx.imp.valid <- val.prob(lranycx.imp.valid.pred[,1]
                        , valid.org.na$Complication10
                        , pl = FALSE)

cal.lranycx.imp.valid
```

```
##           Dxy       C (ROC)            R2             D      D:Chi-sq
##   0.714101484   0.857050742   0.458727414   0.408414585 204.390463506
##           D:p             U      U:Chi-sq           U:p             Q
##            NA  -0.003724183   0.145356768   0.929899853   0.412138769
##         Brier     Intercept         Slope          Emax           E90
##   0.147463927  -0.003803765   0.967311705   0.043469325   0.025091963
##          Eavg           S:z           S:p
##   0.014309951   0.389736767   0.696731206
```

```
hosmer_lemeshow(valid.org.na$Complication10, lranycx.imp.valid.pred[,1], 10, 'C')
```

```
## PVALUE 0.517105
## stat 7.182232
```

```
## [1] 0.517105
```

```
hosmer_lemeshow(valid.org.na$Complication10, lranycx.imp.valid.pred[,1], 10, 'H')
```

```
## PVALUE 0.2065468
## stat 10.91526
```

```
## [1] 0.2065468
```

```
CalibrationCurves::val.prob.ci.2(lranycx.imp.valid.pred[,1], valid.org.na$Complication10
              , lty.smooth = 2
              , CL.smooth = FALSE
              , col.ideal = "black")
```

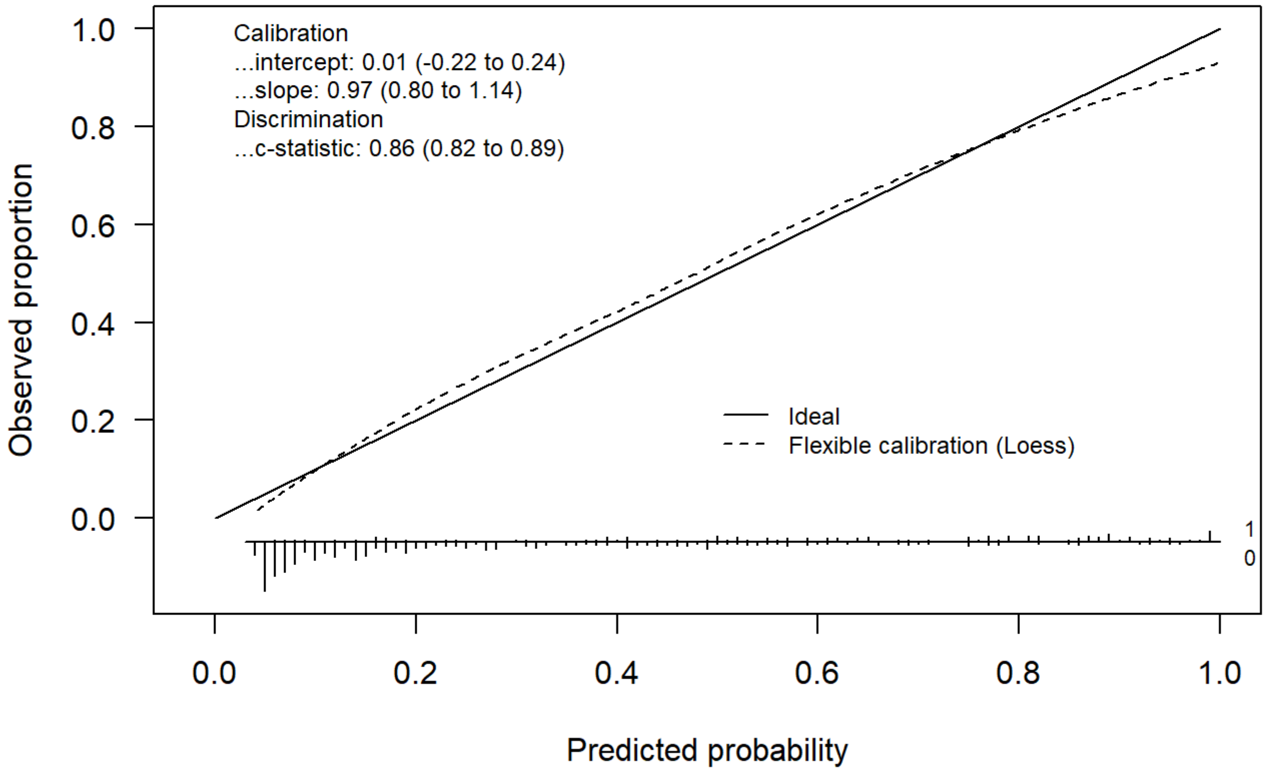

```
## Call:
## CalibrationCurves::val.prob.ci.2(p = lranycx.imp.valid.pred[,
##     1], y = valid.org.na$Complication10, CL.smooth = FALSE, lty.smooth = 2,
##     col.ideal = "black")
##
## A 95% confidence interval is given for the calibration intercept, calibration slope and c-statist
ic.
##
##           Dxy       C (ROC)            R2             D      D:Chi-sq
##   0.714118679   0.857059339   0.458991230   0.408706466 204.535820273
##           D:p             U      U:Chi-sq           U:p             Q
##   0.000000000  -0.003724183   0.145356768   0.929899853   0.412430650
##         Brier     Intercept         Slope          Emax  Brier scaled
##   0.147463927   0.011196537   0.967311705   0.008012940   0.371156630
##          Eavg           ECI
##   0.019304933   0.051834169
```

6.6.3 Isoregression model validation

```
# Predicted probability and classification: isoregression model
isoanycx.imp.valid.pred.prob <- predict(lranycx.imp,
                                       valid.org.na[,-length(valid.org.na)],
                                       type = "response")
isoanycx.imp.valid.pred <- isoanycx.imp(as.data.frame(isoanycx.imp.valid.pred.prob)[,1])
isoanycx.imp.valid.class <- ifelse(isoanycx.imp.valid.pred>=0.5, 1, 0)

# Confusion matrix
cl_table.isoanycx.imp.valid <- table (valid.org.na$Complication10, isoanycx.imp.valid.class)
rownames(cl_table.isoanycx.imp.valid) <- c("No Cx", "Complicated")
colnames(cl_table.isoanycx.imp.valid) <- c("No Cx", "Complicated")
addmargins(cl_table.isoanycx.imp.valid)
```

```
##              isoanycx.imp.valid.class
##               No Cx Complicated Sum
##   No Cx         234          77 311
##   Complicated    36         151 187
##   Sum           270         228 498
```

```
# Sensitivity
round(cl_table.isoanycx.imp.valid[2,2]/(cl_table.isoanycx.imp.valid[2,1] + cl_table.isoanycx.imp.val
id[2,2]),3)
```

```
## [1] 0.807
```

```
# Specificity
round(cl_table.isoanycx.imp.valid[1,1]/(cl_table.isoanycx.imp.valid[1,1] + cl_table.isoanycx.imp.val
id[1,2]),3)
```

```
## [1] 0.752
```

```
# Positive predictive rate
round(cl_table.isoanycx.imp.valid[2,2]/(cl_table.isoanycx.imp.valid[1,2] + cl_table.isoanycx.imp.val
id[2,2]),3)
```

```
## [1] 0.662
```

```
# Negative predictive rate
round(cl_table.isoanycx.imp.valid[1,1]/(cl_table.isoanycx.imp.valid[1,1] + cl_table.isoanycx.imp.val
id[2,1]),3)
```

```
## [1] 0.867
```

```
#model accuracy (classification accuracy)
mean((valid.org.na$Complication10 == isoanycx.imp.valid.class))
```

```
## [1] 0.7730924
```

```
#Discriminability

roc.isoanycx.imp.valid <- roc(valid.org.na$Complication10
                            , isoanycx.imp.valid.pred
                            , add = TRUE)
```

```
## Setting levels: control = 0, case = 1
```

```
## Setting direction: controls < cases
```

```
roc.isoanycx.imp.valid$auc
```

```
## Area under the curve: 0.8528
```

```
ci(roc.isoanycx.imp.valid)
```

```
## 95% CI: 0.8188-0.8867 (DeLong)
```

```
pROC::coords(roc.isoanycx.imp.valid
       , "best"
       , transpose = TRUE
       , best.method="youden"
       )
```

```
##   threshold specificity sensitivity
##   0.5125000   0.7684887   0.7967914
```

```
plot.roc(roc.isoanycx.imp.valid
         , print.auc = TRUE
         , print.thres = FALSE
         , auc.polygon = TRUE
)
```

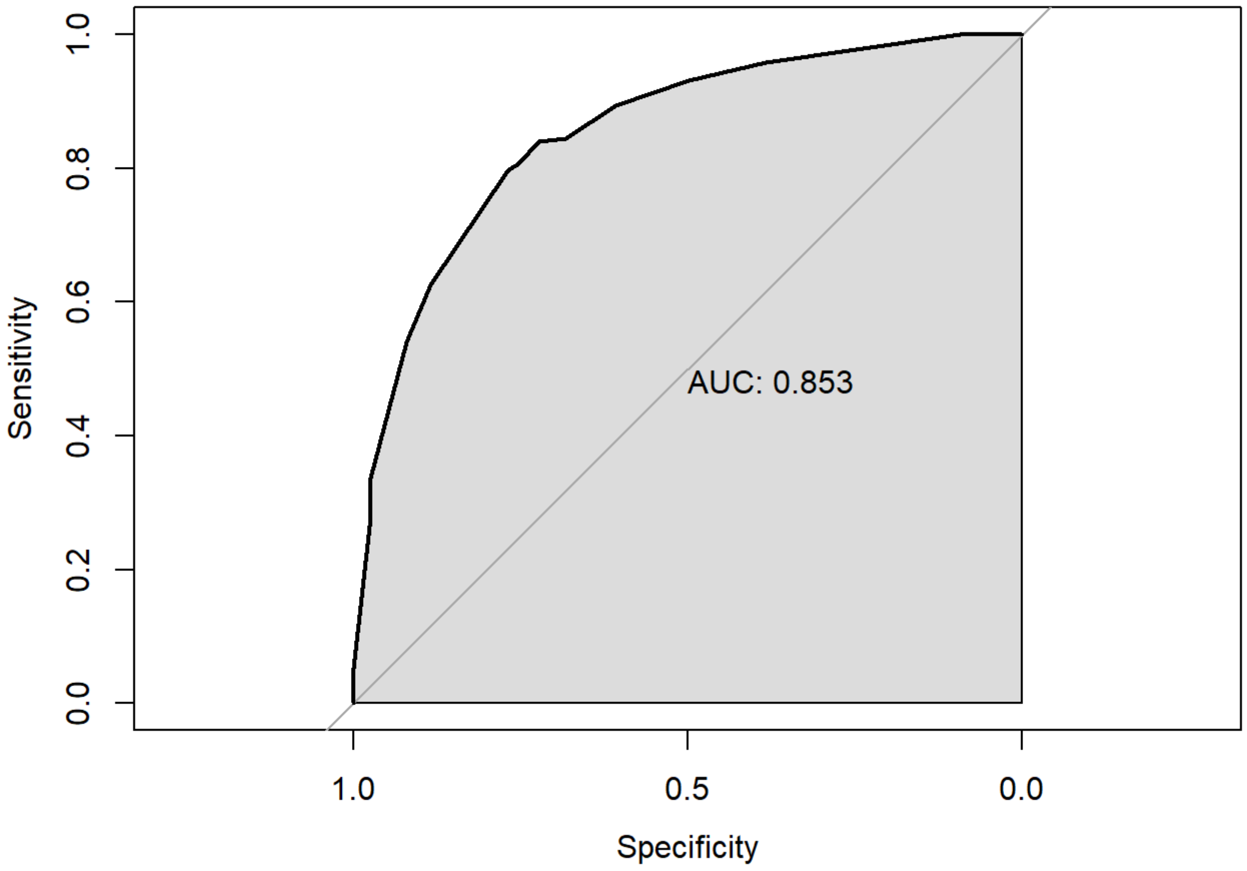


```
# Threshold discrimination ability

isoanycx.imp.valid.pred.dcs <- ifelse(isoanycx.imp.valid.pred > 0.3226489, 1, 0)

roc.isoanycx.imp.valid.dcs <- roc(valid.org.na$Complication10
                          ,isoanycx.imp.valid.pred.dcs
                          , add = TRUE)
```

```
## Setting levels: control = 0, case = 1
## Setting direction: controls < cases
```

```
roc.lranycx.imp.valid.dcs$auc
```

```
## Area under the curve: 0.7773
```

```
ci(roc.lranycx.imp.valid.dcs)
```

```
## 95% CI: 0.7399-0.8146 (DeLong)
```

```
pROC::coords(roc.lranycx.imp.valid.dcs,
             "best",
             transpose = TRUE,
             best.method = "youden")
```

```
##   threshold specificity sensitivity
##   0.5000000   0.7524116   0.8021390
```

```
plot.roc(roc.lranycx.imp.valid.dcs
         , print.auc = TRUE
         , print.thres = FALSE
         , auc.polygon = TRUE
)
```

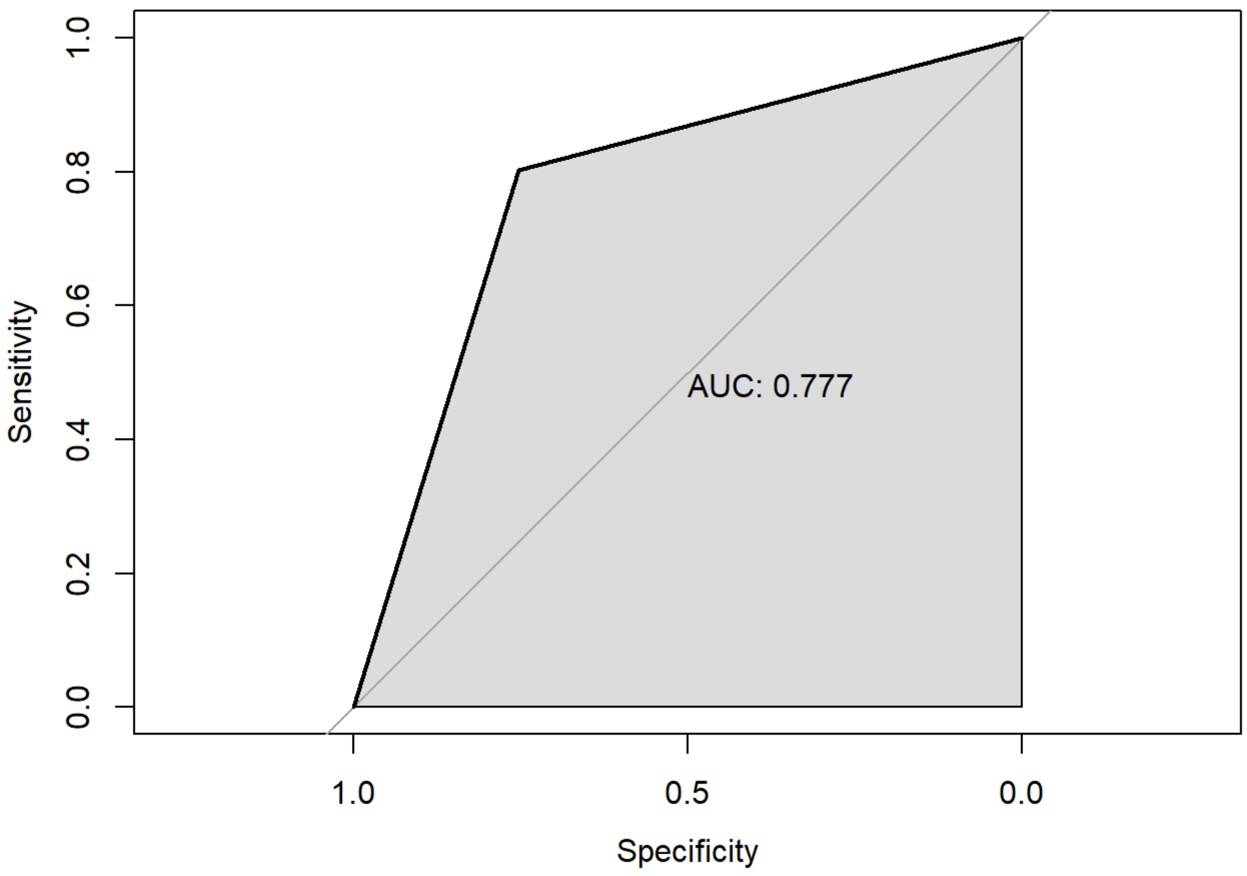


```
# Calibrations: on the validing dataset
cal.isoanycx.imp.valid <- val.prob(isoanycx.imp.valid.pred
                        , valid.org.na$Complication10
                        , pl = FALSE)

cal.isoanycx.imp.valid
```

```
##          Dxy      C (ROC)           R2            D     D:Chi-sq          D:p
##   0.65688985   0.82844493   0.38582314   0.33208921 153.42894780           NA
##            U     U:Chi-sq          U:p            Q        Brier    Intercept
##   0.01107484   7.08335337   0.02896472   0.32101437   0.14884417  -0.16744159
##        Slope         Emax          E90         Eavg          S:z          S:p
##   0.80015880   0.05544314   0.05544314   0.03634088   2.07644207   0.03785308
```

```
hosmer_lemeshow(valid.org.na$Complication10, isoanycx.imp.valid.pred, 10, 'C')
```

```
## PVALUE 0.06712634
## stat 14.6128
```

```
## [1] 0.06712634
```

```
hosmer_lemeshow(valid.org.na$Complication10, isoanycx.imp.valid.pred, 10, 'H')
```

```
## PVALUE 0.03789525
## stat 16.32983
```

```
## [1] 0.03789525
```

```
CalibrationCurves::val.prob.ci.2(isoanycx.imp.valid.pred, valid.org.na$Complication10
              , lty.smooth = 2
              , CL.smooth = FALSE
              , col.ideal = "black")
```

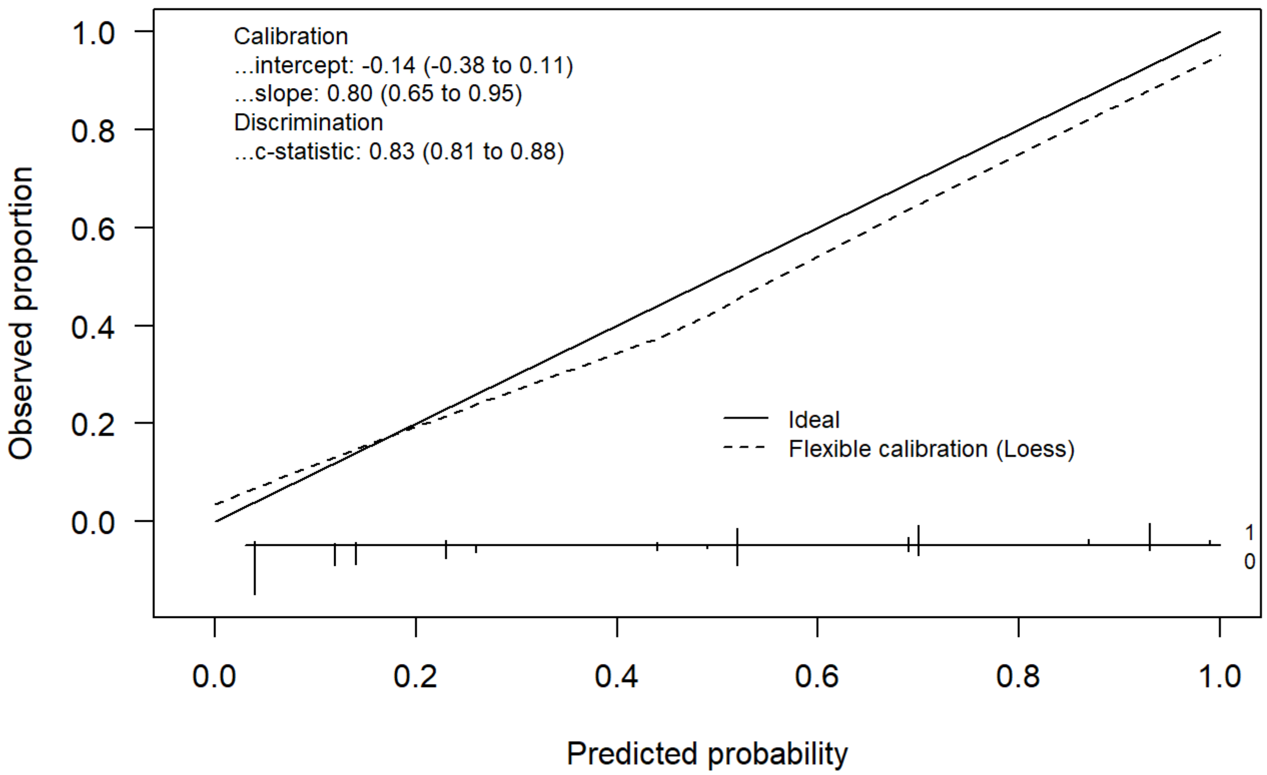


```
## Call:
## CalibrationCurves::val.prob.ci.2(p = isoanycx.imp.valid.pred,
##     y = valid.org.na$Complication10, CL.smooth = FALSE, lty.smooth = 2,
##     col.ideal = "black")
##
## A 95% confidence interval is given for the calibration intercept, calibration slope and c-statist
ic.
##
##          Dxy       C (ROC)           R2            D     D:Chi-sq          D:p
##   0.65688985    0.82844493   0.40070879   0.34752135 160.51230118   0.00000000
##            U      U:Chi-sq          U:p            Q        Brier    Intercept
##   0.01107484    7.08335337   0.02896472   0.33644651   0.16149106  -0.13709846
##        Slope          Emax Brier scaled         Eavg          ECI
##   0.80015880    0.08056243   0.31133949   0.03848239   0.18641793
```

6.6.4 Platt scaling model validation

```
# Predicted probability and classification: Platt scaling model
colnames(lranycx.imp.valid.pred) <- c("yhat")
pltanycx.imp.valid.pred <- as.data.frame(predict(pltanycx.imp, lranycx.imp.valid.pred, type = "respo
nse" ))
pltanycx.imp.valid.class <- ifelse(pltanycx.imp.valid.pred>=0.5, 1, 0)


# Confusion matrix
cl_table.pltanycx.imp.valid <- table (valid.org.na$Complication10, pltanycx.imp.valid.class)
rownames(cl_table.pltanycx.imp.valid) <- c("No Cx", "Complicated")
colnames(cl_table.pltanycx.imp.valid) <- c("No Cx", "Complicated")
addmargins(cl_table.pltanycx.imp.valid)
```

```
##              pltanycx.imp.valid.class
##               No Cx Complicated Sum
##   No Cx         264          47 311
##   Complicated    58         129 187
##   Sum           322         176 498
```

```
# Sensitivity
round(cl_table.pltanycx.imp.valid[2,2]/(cl_table.pltanycx.imp.valid[2,1] + cl_table.pltanycx.imp.val
id[2,2]),3)
```

```
## [1] 0.69
```

```
# Specificity
round(cl_table.pltanycx.imp.valid[1,1]/(cl_table.pltanycx.imp.valid[1,1] + cl_table.pltanycx.imp.val
id[1,2]),3)
```

```
## [1] 0.849
```

```
# Positive predictive rate
round(cl_table.pltanycx.imp.valid[2,2]/(cl_table.pltanycx.imp.valid[1,2] + cl_table.pltanycx.imp.val
id[2,2]),3)
```

```
## [1] 0.733
```

```
# Negative predictive rate
round(cl_table.pltanycx.imp.valid[1,1]/(cl_table.pltanycx.imp.valid[1,1] + cl_table.pltanycx.imp.val
id[2,1]),3)
```

```
## [1] 0.82
```

```
#model accuracy (classification accuracy)
mean((valid.org.na$Complication10 == pltanycx.imp.valid.class))
```

```
## [1] 0.7891566
```

```
#Discriminability

roc.pltanycx.imp.valid <- roc(valid.org.na$Complication10
                              , pltanycx.imp.valid.pred[,1]
                              , add = TRUE)
```

```
## Setting levels: control = 0, case = 1
```

```
## Setting direction: controls < cases
```

```
roc.pltanycx.imp.valid$auc
```

```
## Area under the curve: 0.857
```

```
ci(roc.pltanycx.imp.valid)
```

```
## 95% CI: 0.8238-0.8903 (DeLong)
```

```
pROC::coords(roc.pltanycx.imp.valid
       , "best"
       , transpose = TRUE
       , best.method="youden"
       )
```

```
##   threshold specificity sensitivity
##   0.2551738   0.7363344   0.8395722
```

```
plot.roc(roc.pltanycx.imp.valid
         , print.auc = TRUE
         , print.thres = FALSE
         , auc.polygon = TRUE
)
```

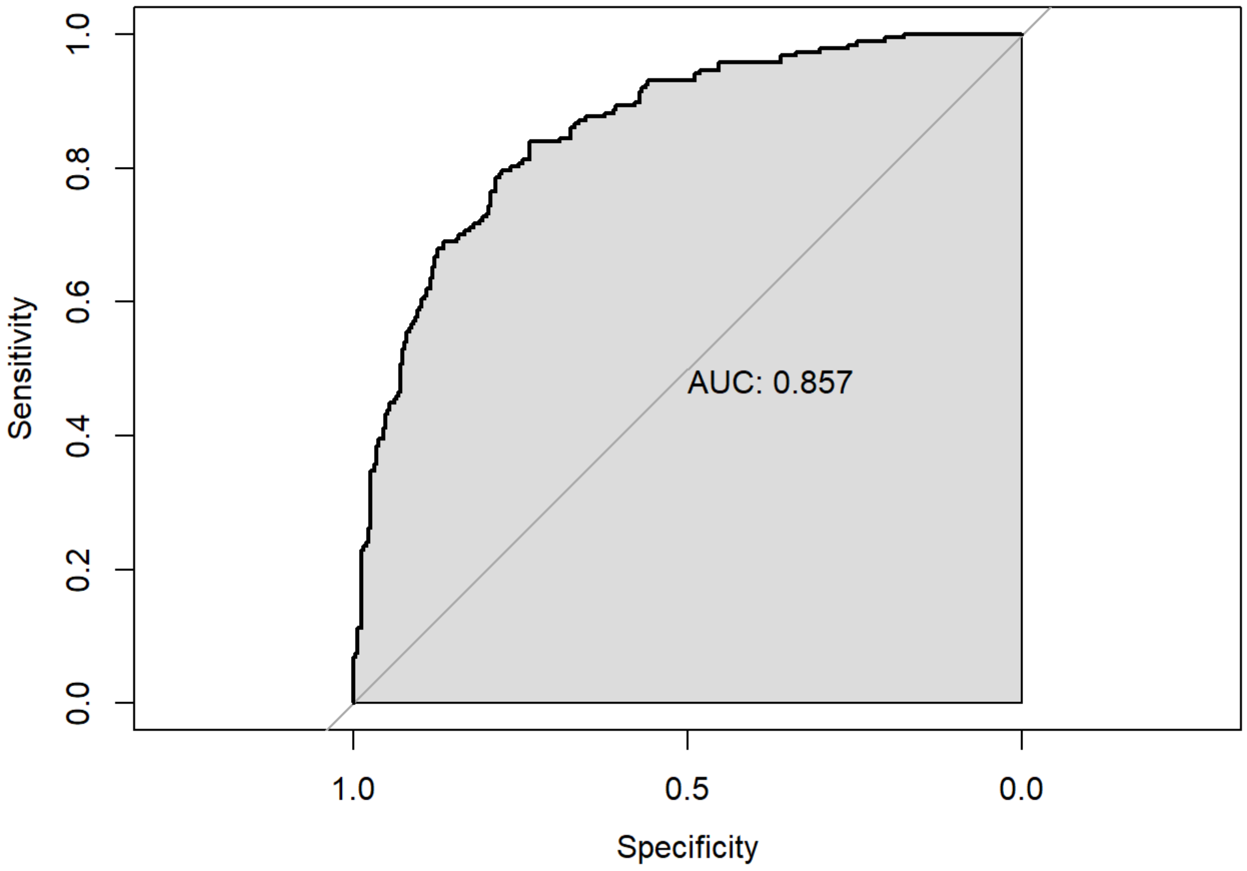


```
# Threshold discrimination ability

pltanycx.imp.valid.pred.dcs <- ifelse(pltanycx.imp.valid.pred > 0.3226489, 1, 0)

roc.pltanycx.imp.valid.dcs <- roc(valid.org.na$Complication10
                          ,pltanycx.imp.valid.pred.dcs[,1]
                          , add = TRUE)
```

```
## Setting levels: control = 0, case = 1
## Setting direction: controls < cases
```

```
roc.lranycx.imp.valid.dcs$auc
```

```
## Area under the curve: 0.7773
```

```
ci(roc.lranycx.imp.valid.dcs)
```

```
## 95% CI: 0.7399-0.8146 (DeLong)
```

```
pROC::coords(roc.lranycx.imp.valid.dcs,
             "best",
             transpose = TRUE,
             best.method = "youden")
```

```
##   threshold specificity sensitivity
##   0.5000000   0.7524116   0.8021390
```

```
plot.roc(roc.lranycx.imp.valid.dcs
         , print.auc = TRUE
         , print.thres = FALSE
         , auc.polygon = TRUE
)
```

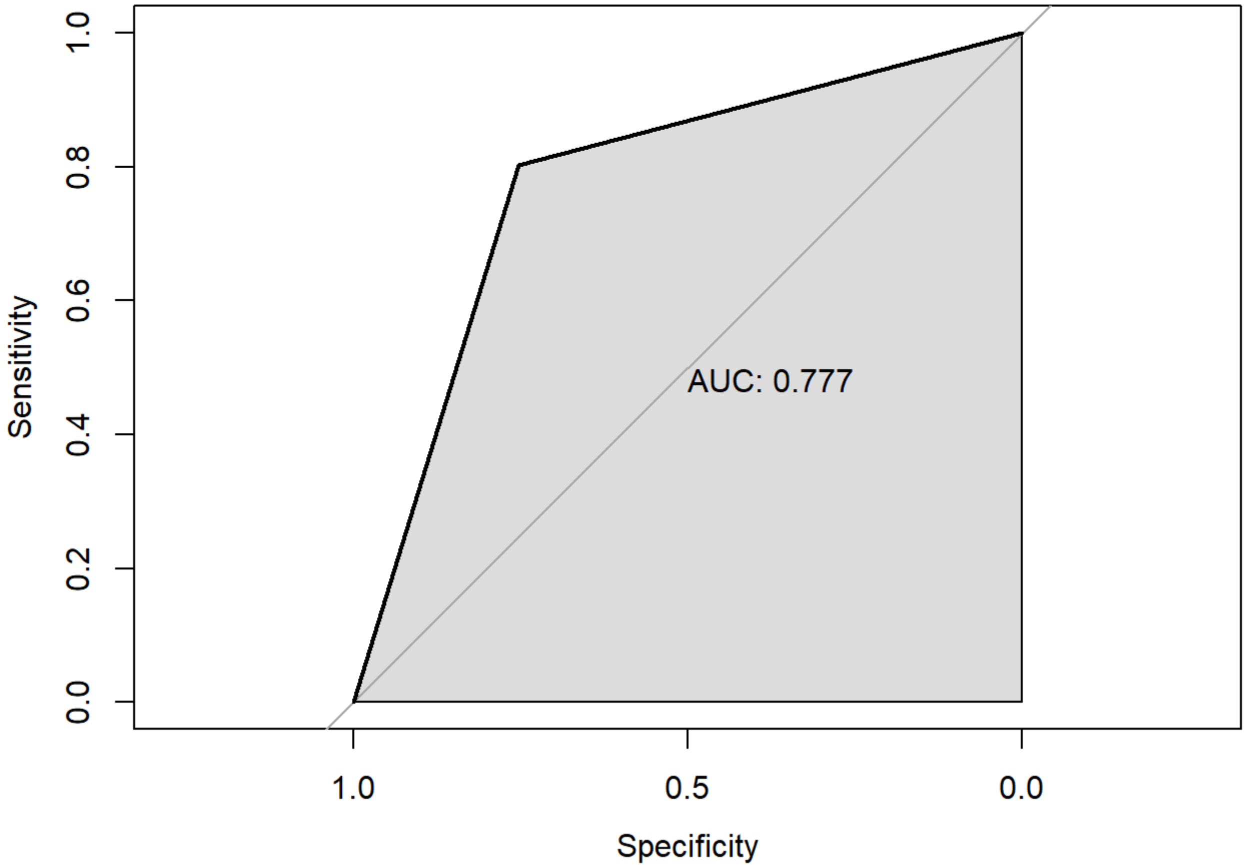


```
# Calibrations: on the validing dataset
cal.pltanycx.imp.valid <- val.prob(pltanycx.imp.valid.pred[,1]
                        , valid.org.na$Complication10
                        , pl = FALSE)

cal.pltanycx.imp.valid
```

```
##           Dxy       C (ROC)            R2             D      D:Chi-sq
##  7.141187e-01  8.570593e-01  4.480335e-01  3.966542e-01  1.985338e+02
##           D:p             U      U:Chi-sq           U:p             Q
##            NA -1.115461e-04  1.944450e+00  3.782405e-01  3.967657e-01
##         Brier     Intercept         Slope          Emax           E90
##  1.487777e-01 -1.340124e-01  9.105652e-01  6.544416e-02  5.957961e-02
##          Eavg           S:z           S:p
##  2.603249e-02  1.004377e+00  3.151971e-01
```

```
hosmer_lemeshow(valid.org.na$Complication10, pltanycx.imp.valid.pred[,1], 10, 'C')
```

```
## PVALUE 0.1091489
## stat 13.07934
```

```
## [1] 0.1091489
```

```
hosmer_lemeshow(valid.org.na$Complication10, pltanycx.imp.valid.pred[,1], 10, 'H')
```

```
## PVALUE 0.4320466
## stat 8.014586
```

```
## [1] 0.4320466
```

```
CalibrationCurves::val.prob.ci.2(pltanycx.imp.valid.pred[,1], valid.org.na$Complication10
              , lty.smooth = 2
              , CL.smooth = FALSE
              , col.ideal = "black")
```

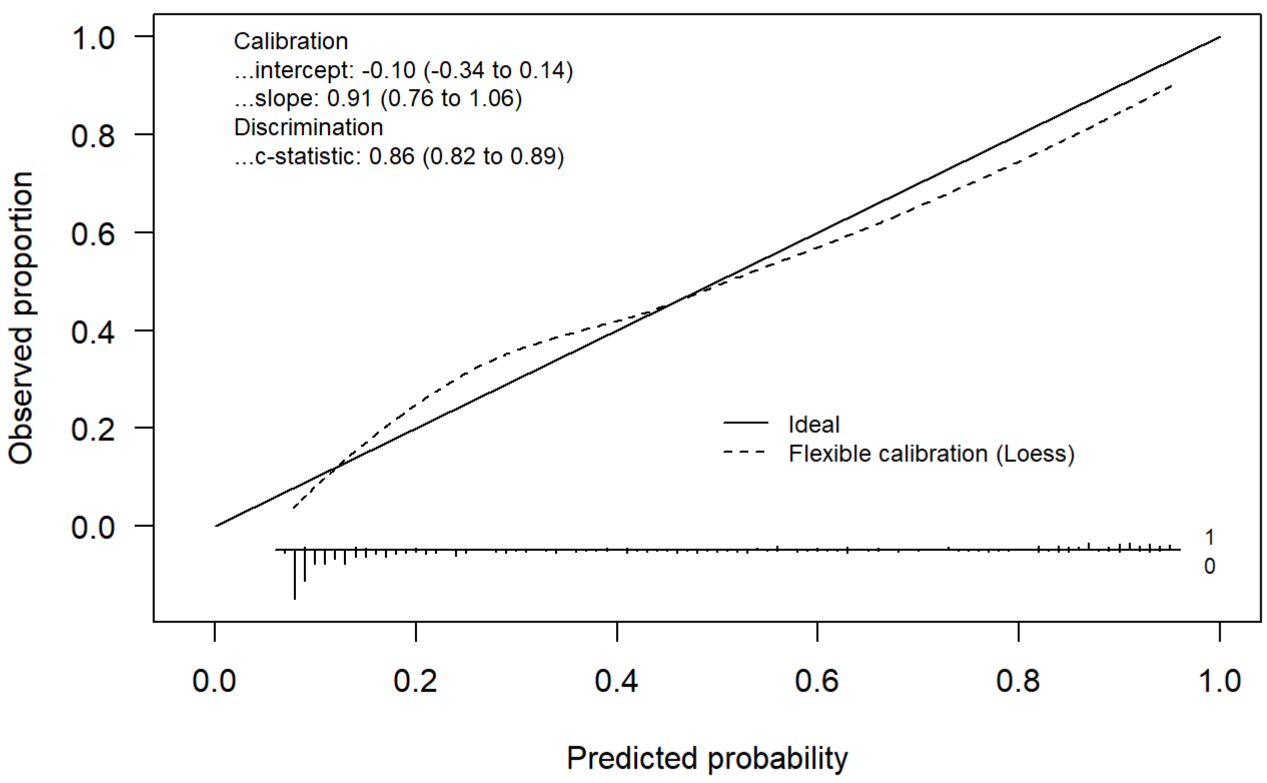


```
## Call:
## CalibrationCurves::val.prob.ci.2(p = pltanycx.imp.valid.pred[,
##     1], y = valid.org.na$Complication10, CL.smooth = FALSE, lty.smooth = 2,
##     col.ideal = "black")
##
## A 95% confidence interval is given for the calibration intercept, calibration slope and c-statist
ic.
##
##           Dxy       C (ROC)            R2             D      D:Chi-sq
##  7.141531e-01  8.570765e-01  4.515979e-01  4.005587e-01  2.004782e+02
##           D:p             U      U:Chi-sq           U:p             Q
##  0.000000e+00 -1.115461e-04  1.944450e+00  3.782405e-01  4.006703e-01
##         Brier     Intercept         Slope          Emax  Brier scaled
##  1.487777e-01 -1.012073e-01  9.105652e-01  4.612215e-02  3.655540e-01
##          Eavg           ECI
##  3.373832e-02  1.464348e-01
```

7 Risk prediction model development: Presence of CVD3 or higher complications

7.1 Original data analysis

Original data analysis includes complete cases only.

```
# Logistic regression model: Enter all input parameters
lrcvd.org.enter <- glm(CVD_23 ~ GENDER
                       + CIA_AMI
                       + CIA_CHF
                       + CIA_PVD
                       + CIA_CEVD
                       + CIA_DEM
                       + CIA_COPD
                       + CIA_PUD
                       + CIA_LD_MLD
                       + CIA_LD_SEV
                       + CIA_DM
                       + CIA_DM_CX
                       + CIA_HEMIPLAEGIA
                       + CIA_CKD
                       + CIA_CA
                       + CIA_CA_MET
                       + PREOP_HB
                       + PREOP_WCC
                       + PREOP_NA
                       + PREOP_K
                       + SURG_SEV
                       + SURG_SCHEDULED_TYPE
                       + PreopICU
                       , data =  train.org.na, family = binomial, na.action = "na.omit")


summary(lrcvd.org.enter)
```

```
##
## Call:
## glm(formula = CVD_23 ~ GENDER + CIA_AMI + CIA_CHF + CIA_PVD +
##     CIA_CEVD + CIA_DEM + CIA_COPD + CIA_PUD + CIA_LD_MLD + CIA_LD_SEV +
##     CIA_DM + CIA_DM_CX + CIA_HEMIPLAEGIA + CIA_CKD + CIA_CA +
##     CIA_CA_MET + PREOP_HB + PREOP_WCC + PREOP_NA + PREOP_K +
##     SURG_SEV + SURG_SCHEDULED_TYPE + PreopICU, family = binomial,
##     data = train.org.na, na.action = "na.omit")
##
## Deviance Residuals:
##     Min       1Q   Median       3Q      Max
## -2.3332  -0.5158  -0.3179  -0.2325   2.7260
##
## Coefficients:
##                       Estimate Std. Error z value Pr(>|z|)
## (Intercept)           -5.11360    3.14279  -1.627  0.10372
## GENDER1               -0.08617    0.16515  -0.522  0.60181
## CIA_AMI1               0.31572    0.90332   0.350  0.72671
## CIA_CHF1               1.86255    0.31116   5.986 2.15e-09 ***
## CIA_PVD1               1.79497    0.35672   5.032 4.86e-07 ***
## CIA_CEVD1              0.89564    0.49457   1.811  0.07015 .
## CIA_DEM1               0.62256    0.28115   2.214  0.02680 *
## CIA_COPD1             -0.35594    0.60589  -0.587  0.55689
## CIA_PUD1               0.34945    0.54635   0.640  0.52243
## CIA_LD_MLD1          -13.55885  560.15883  -0.024  0.98069
## CIA_LD_SEV1          -13.77444  766.43307  -0.018  0.98566
## CIA_DM1               -0.16154    0.32529  -0.497  0.61946
## CIA_DM_CX1             0.17014    0.25263   0.673  0.50063
## CIA_HEMIPLAEGIA1       0.32291    0.73223   0.441  0.65921
## CIA_CKD1               1.40733    0.24932   5.645 1.65e-08 ***
## CIA_CA1                0.64097    0.28689   2.234  0.02547 *
## CIA_CA_MET1            1.81350    0.38807   4.673 2.97e-06 ***
## PREOP_HB              -0.18100    0.04289  -4.220 2.44e-05 ***
## PREOP_WCC              0.03791    0.01660   2.284  0.02236 *
## PREOP_NA               0.02850    0.02118   1.345  0.17849
## PREOP_K               -0.03765    0.15638  -0.241  0.80973
## SURG_SEV1              0.60264    0.18892   3.190  0.00142 **
## SURG_SEV2              1.38902    0.24498   5.670 1.43e-08 ***
## SURG_SCHEDULED_TYPE1   0.96755    0.18647   5.189 2.12e-07 ***
## PreopICU1              0.89592    0.60975   1.469  0.14174
## ---
## Signif. codes:  0 '***' 0.001 '**' 0.01 '*' 0.05 '.' 0.1 ' ' 1
##
## (Dispersion parameter for binomial family taken to be 1)
##
##     Null deviance: 1463.4  on 1570  degrees of freedom
## Residual deviance: 1075.9  on 1546  degrees of freedom
## AIC: 1125.9
##
## Number of Fisher Scoring iterations: 14
```

```
# Stepwise selection method: AIC guided.
# Suppressed output
lrcvd.org.sel <- step(lrcvd.org.enter)
```

```
summary(lrcvd.org.sel)
```

```
##
## Call:
## glm(formula = CVD_23 ~ CIA_CHF + CIA_PVD + CIA_CEVD + CIA_DEM +
##     CIA_CKD + CIA_CA + CIA_CA_MET + PREOP_HB + PREOP_WCC + PREOP_NA +
##     SURG_SEV + SURG_SCHEDULED_TYPE + PreopICU, family = binomial,
##     data = train.org.na, na.action = "na.omit")
##
## Deviance Residuals:
##     Min       1Q   Median       3Q      Max
## -2.2774  -0.5182  -0.3196  -0.2350   2.7196
##
## Coefficients:
##                      Estimate Std. Error z value Pr(>|z|)
## (Intercept)          -5.46601    2.96017  -1.847 0.064817 .
## CIA_CHF1              1.90402    0.30702   6.202 5.59e-10 ***
## CIA_PVD1              1.77671    0.34805   5.105 3.31e-07 ***
## CIA_CEVD1             0.95877    0.41837   2.292 0.021924 *
## CIA_DEM1              0.62777    0.28001   2.242 0.024964 *
## CIA_CKD1              1.44860    0.23960   6.046 1.48e-09 ***
## CIA_CA1               0.62909    0.28546   2.204 0.027540 *
## CIA_CA_MET1           1.79329    0.38367   4.674 2.95e-06 ***
## PREOP_HB             -0.19108    0.04198  -4.552 5.32e-06 ***
## PREOP_WCC             0.03824    0.01648   2.321 0.020308 *
## PREOP_NA              0.03030    0.02091   1.449 0.147228
## SURG_SEV1             0.60413    0.18206   3.318 0.000906 ***
## SURG_SEV2             1.41354    0.23932   5.906 3.49e-09 ***
## SURG_SCHEDULED_TYPE1  0.97594    0.18311   5.330 9.84e-08 ***
## PreopICU1             0.92209    0.59818   1.541 0.123199
## ---
## Signif. codes:  0 '***' 0.001 '**' 0.01 '*' 0.05 '.' 0.1 ' ' 1
##
## (Dispersion parameter for binomial family taken to be 1)
##
##     Null deviance: 1463.4  on 1570  degrees of freedom
## Residual deviance: 1080.6  on 1556  degrees of freedom
## AIC: 1110.6
##
## Number of Fisher Scoring iterations: 5
```

```
# Final logistic regression model with the selected input parameters
lrcvd.org.final <- glm(CVD_23 ~ CIA_CHF
                       + CIA_PVD
                       + CIA_CEVD
                       + CIA_DEM
                       + CIA_CKD
                       + CIA_CA
                       + CIA_CA_MET
                       + PREOP_HB
                       + PREOP_WCC
                       + PREOP_NA
                       + SURG_SEV
                       + SURG_SCHEDULED_TYPE
                       + PreopICU
                       , data =  train.org.na
                       , family = binomial, na.action = "na.omit")

summary(lrcvd.org.final)
```

```
##
## Call:
## glm(formula = CVD_23 ~ CIA_CHF + CIA_PVD + CIA_CEVD + CIA_DEM +
##     CIA_CKD + CIA_CA + CIA_CA_MET + PREOP_HB + PREOP_WCC + PREOP_NA +
##     SURG_SEV + SURG_SCHEDULED_TYPE + PreopICU, family = binomial,
##     data = train.org.na, na.action = "na.omit")
##
## Deviance Residuals:
##     Min       1Q   Median       3Q      Max
## -2.2774  -0.5182  -0.3196  -0.2350   2.7196
##
## Coefficients:
##                      Estimate Std. Error z value Pr(>|z|)
## (Intercept)          -5.46601    2.96017  -1.847 0.064817 .
## CIA_CHF1              1.90402    0.30702   6.202 5.59e-10 ***
## CIA_PVD1              1.77671    0.34805   5.105 3.31e-07 ***
## CIA_CEVD1             0.95877    0.41837   2.292 0.021924 *
## CIA_DEM1              0.62777    0.28001   2.242 0.024964 *
## CIA_CKD1              1.44860    0.23960   6.046 1.48e-09 ***
## CIA_CA1               0.62909    0.28546   2.204 0.027540 *
## CIA_CA_MET1           1.79329    0.38367   4.674 2.95e-06 ***
## PREOP_HB             -0.19108    0.04198  -4.552 5.32e-06 ***
## PREOP_WCC             0.03824    0.01648   2.321 0.020308 *
## PREOP_NA              0.03030    0.02091   1.449 0.147228
## SURG_SEV1             0.60413    0.18206   3.318 0.000906 ***
## SURG_SEV2             1.41354    0.23932   5.906 3.49e-09 ***
## SURG_SCHEDULED_TYPE1  0.97594    0.18311   5.330 9.84e-08 ***
## PreopICU1             0.92209    0.59818   1.541 0.123199
## ---
## Signif. codes:  0 '***' 0.001 '**' 0.01 '*' 0.05 '.' 0.1 ' ' 1
##
## (Dispersion parameter for binomial family taken to be 1)
##
##     Null deviance: 1463.4  on 1570  degrees of freedom
## Residual deviance: 1080.6  on 1556  degrees of freedom
## AIC: 1110.6
##
## Number of Fisher Scoring iterations: 5
```

```
# OR and 95%CI
exp(cbind(OR = coef(lrcvd.org.final), confint(lrcvd.org.final)))
```

```
##                              OR        2.5 %    97.5 %
## (Intercept)          0.004228055 1.194022e-05  1.323836
## CIA_CHF1             6.712810693 3.708550e+00 12.413149
## CIA_PVD1             5.910376384 2.991990e+00 11.794985
## CIA_CEVD1            2.608478975 1.139678e+00  5.951580
## CIA_DEM1             1.873429417 1.073921e+00  3.228361
## CIA_CKD1             4.257149376 2.665046e+00  6.828318
## CIA_CA1              1.875905225 1.049214e+00  3.227985
## CIA_CA_MET1          6.009203211 2.803983e+00 12.754508
## PREOP_HB             0.826066454 7.603352e-01  0.896489
## PREOP_WCC            1.038976458 1.007032e+00  1.074469
## PREOP_NA             1.030765216 9.897539e-01  1.074372
## SURG_SEV1            1.829664019 1.282925e+00  2.621783
## SURG_SEV2            4.110466421 2.574493e+00  6.585644
## SURG_SCHEDULED_TYPE1 2.653663658 1.856314e+00  3.808785
## PreopICU1            2.514537397 7.828449e-01  8.482148
```

```
# ANOVA for individual terms: requires library "car"
car::Anova(lrcvd.org.final, type = "II", test = "Wald")
```

```
## Analysis of Deviance Table (Type II tests)
##
## Response: CVD_23
##                     Df   Chisq Pr(>Chisq)
## CIA_CHF              1 38.4592  5.591e-10 ***
## CIA_PVD              1 26.0583  3.313e-07 ***
## CIA_CEVD             1  5.2518    0.02192 *
## CIA_DEM              1  5.0264    0.02496 *
## CIA_CKD              1 36.5541  1.485e-09 ***
## CIA_CA               1  4.8566    0.02754 *
## CIA_CA_MET           1 21.8473  2.952e-06 ***
## PREOP_HB             1 20.7198  5.316e-06 ***
## PREOP_WCC            1  5.3852    0.02031 *
## PREOP_NA             1  2.1007    0.14723
## SURG_SEV             2 35.1184  2.367e-08 ***
## SURG_SCHEDULED_TYPE  1 28.4056  9.838e-08 ***
## PreopICU             1  2.3762    0.12320
## ---
## Signif. codes:  0 '***' 0.001 '**' 0.01 '*' 0.05 '.' 0.1 ' ' 1
```

```
# Pseudo R sqaured: requires library "rcompanion"
rcompanion::nagelkerke(lrcvd.org.final)
```

```
## $Models
##
## Model: "glm, CVD_23 ~ CIA_CHF + CIA_PVD + CIA_CEVD + CIA_DEM + CIA_CKD + CIA_CA + CIA_CA_MET + PR
EOP_HB + PREOP_WCC + PREOP_NA + SURG_SEV + SURG_SCHEDULED_TYPE + PreopICU, binomial, train.org.na, n
a.omit"
## Null:  "glm, CVD_23 ~ 1, binomial, train.org.na, na.omit"
##
## $Pseudo.R.squared.for.model.vs.null
##                              Pseudo.R.squared
## McFadden                             0.261610
## Cox and Snell (ML)                   0.216277
## Nagelkerke (Cragg and Uhler)         0.356862
##
## $Likelihood.ratio.test
##  Df.diff LogLik.diff  Chisq    p.value
##      -14     -191.43 382.85 5.1653e-73
##
## $Number.of.observations
##
## Model: 1571
## Null:  1571
##
## $Messages
## [1] "Note: For models fit with REML, these statistics are based on refitting with ML"
##
## $Warnings
## [1] "None"
```

```
# Overall p value for model
anova(lrcvd.org.final
      , update(lrcvd.org.final, ~1) # update here produces null model fo comparison
      , test = "Chisq")
```

```
## Analysis of Deviance Table
##
## Model 1: CVD_23 ~ CIA_CHF + CIA_PVD + CIA_CEVD + CIA_DEM + CIA_CKD + CIA_CA +
##     CIA_CA_MET + PREOP_HB + PREOP_WCC + PREOP_NA + SURG_SEV +
##     SURG_SCHEDULED_TYPE + PreopICU
## Model 2: CVD_23 ~ 1
##   Resid. Df Resid. Dev  Df Deviance  Pr(>Chi)
## 1      1556     1080.6
## 2      1570     1463.4 -14  -382.85 < 2.2e-16 ***
## ---
## Signif. codes:  0 '***' 0.001 '**' 0.01 '*' 0.05 '.' 0.1 ' ' 1
```

```
# Standardized residuals
plot(fitted(lrcvd.org.final)
     , rstandard(lrcvd.org.final))
```

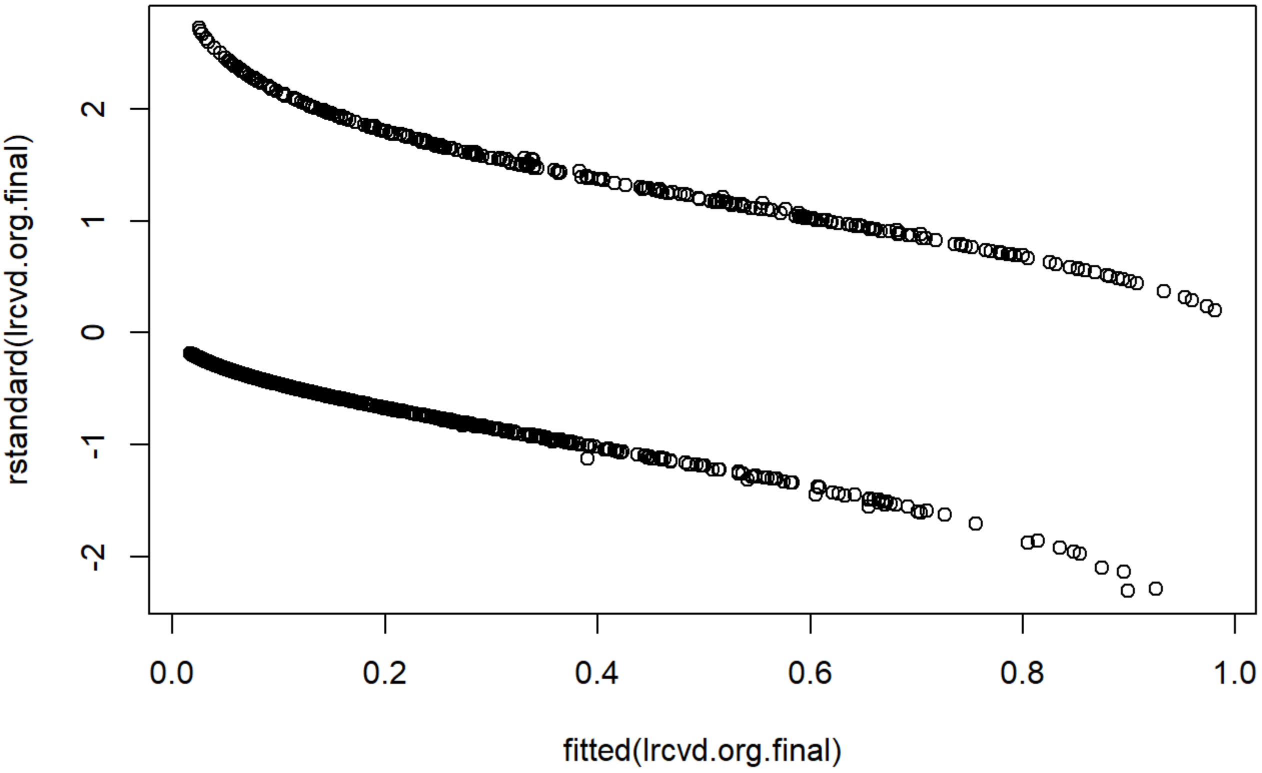


```
plot(lrcvd.org.final)
```

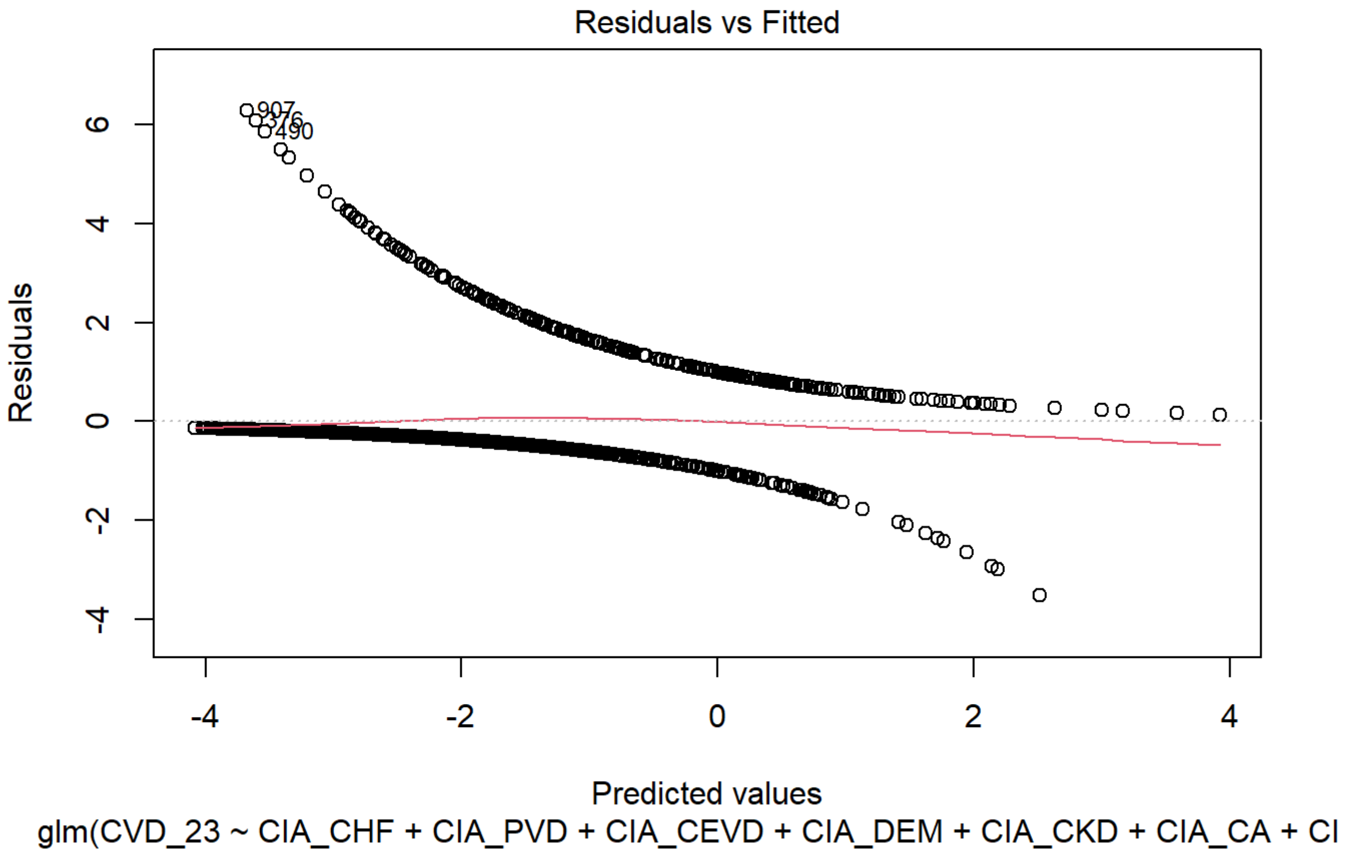
Residuals vs Fitted
Residuals
Predicted values
glm(CVD_23 ~ CIA_CHF + CIA_PVD + CIA_CEVD + CIA_DEM + CIA_CKD + CIA_CA + CI
907
376
490
-4
-2
0
2
4
6

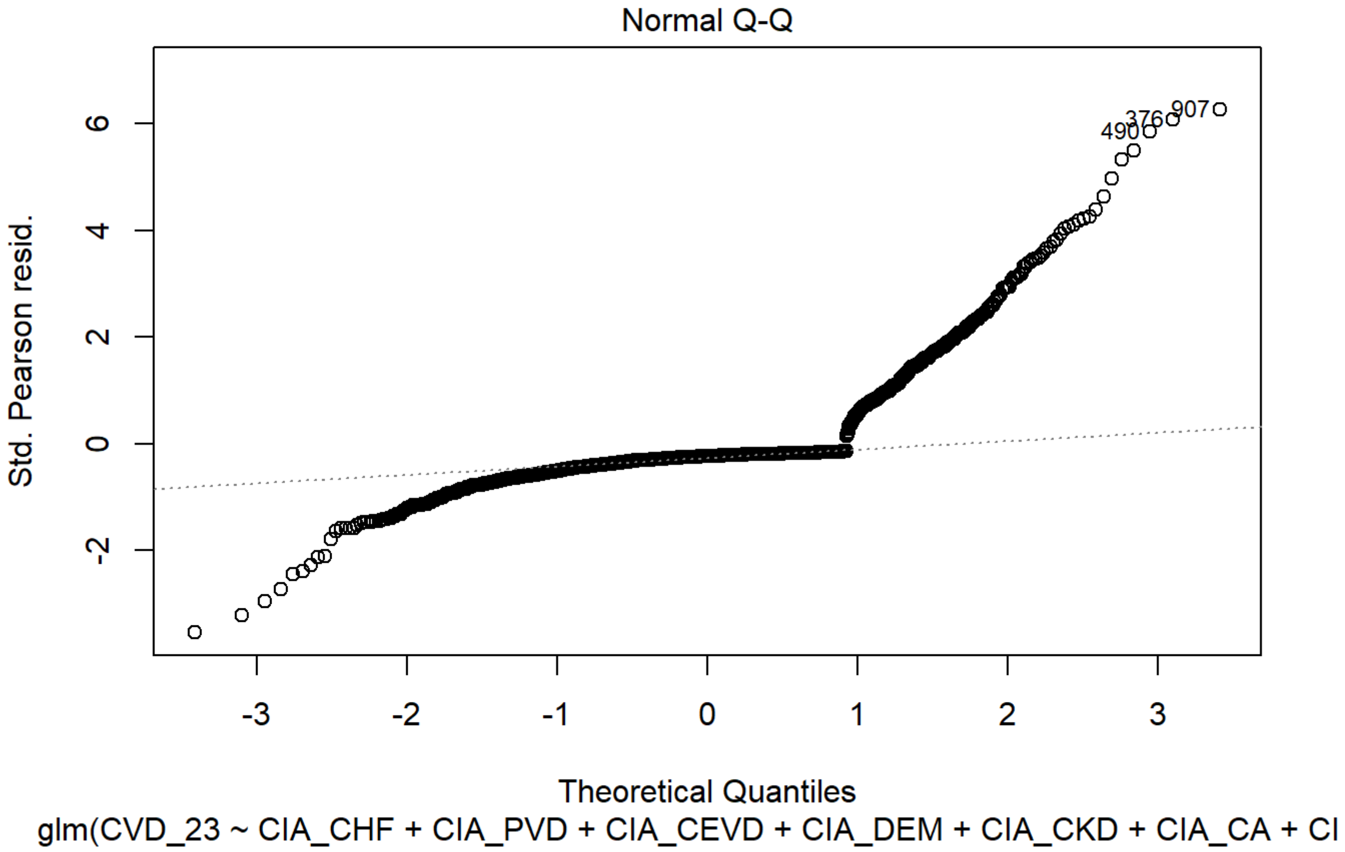
Normal Q-Q
Std. Pearson resid.
Theoretical Quantiles
glm(CVD_23 ~ CIA_CHF + CIA_PVD + CIA_CEVD + CIA_DEM + CIA_CKD + CIA_CA + CI
907
376
490
-3
-2
-1
0
1
2
3
4
6

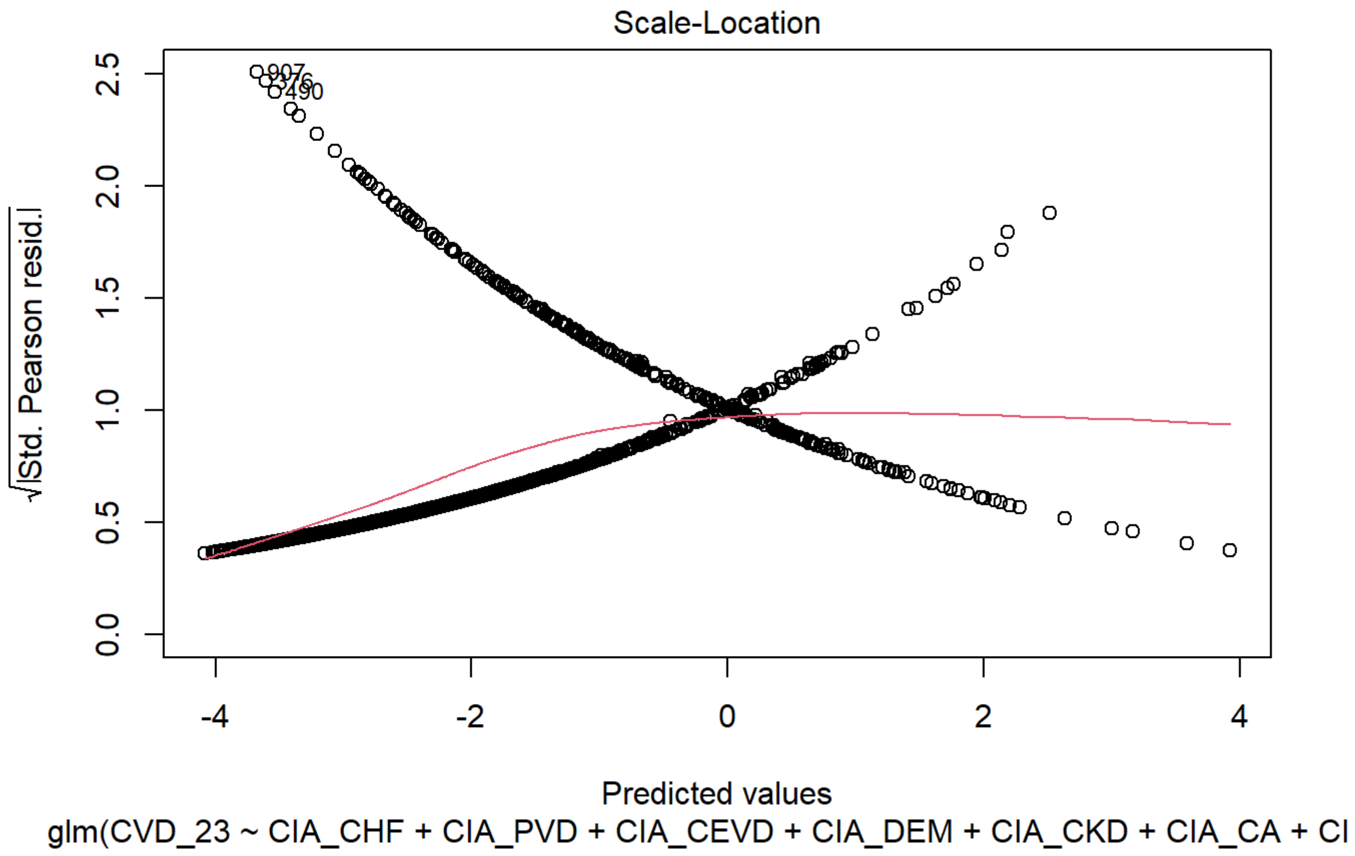
Scale-Location
√|Std. Pearson resid.|
0.0
0.5
1.0
1.5
2.0
2.5
-4
-2
0
2
4
907
376
490
Predicted values
glm(CVD_23 ~ CIA_CHF + CIA_PVD + CIA_CEVD + CIA_DEM + CIA_CKD + CIA_CA + CI

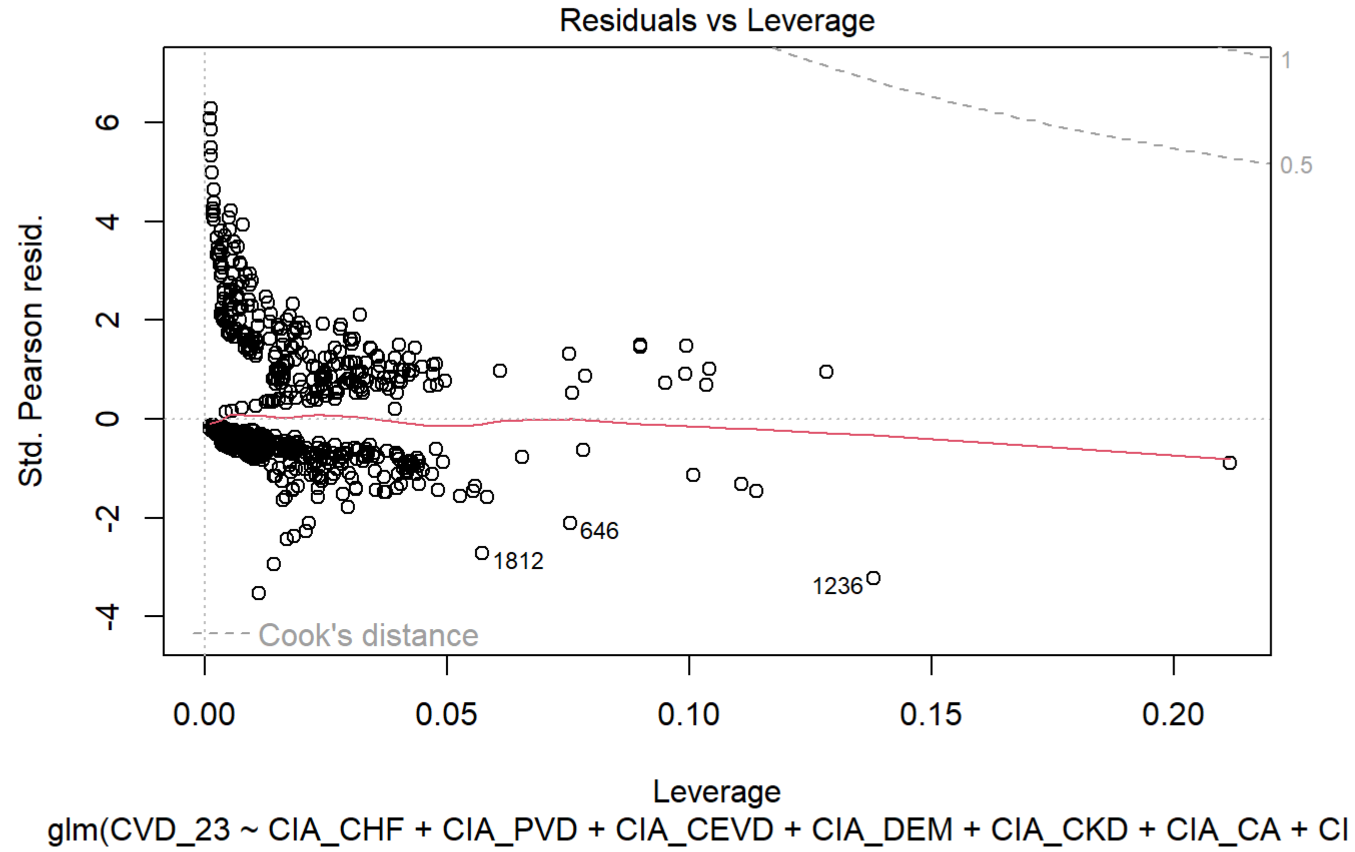
Residuals vs Leverage
Std. Pearson resid.
-4
-2
0
2
4
6
0.00
0.05
0.10
0.15
0.20
1
0.5
1812
646
1236
Cook's distance
Leverage
glm(CVD_23 ~ CIA_CHF + CIA_PVD + CIA_CEVD + CIA_DEM + CIA_CKD + CIA_CA + CI

```
#
#
#
#

#Classification table (confusion matrix)
lrcvd.org.final.pred <- predict(lrcvd.org.final
                                , newdata = train.org.na
                                , type = "response")
lrcvd.org.final.class <- ifelse(lrcvd.org.final.pred>=0.5, 1, 0)
cl_table.lrcvd.org <- table (train.org.na$CVD_23, lrcvd.org.final.class)
rownames(cl_table.lrcvd.org) <- c("CVD12", "CVD345")
colnames(cl_table.lrcvd.org) <- c("CVD12", "CVD345")
addmargins(cl_table.lrcvd.org)
```

```
##         lrcvd.org.final.class
##          CVD12 CVD345  Sum
##   CVD12   1237     57 1294
##   CVD345   175    102  277
##   Sum     1412    159 1571
```

```
#model accuracy (classification accuracy)
mean((train.org.na$CVD_23 == lrcvd.org.final.class))
```

```
## [1] 0.8523234
```

```
#Discriminability

roc.lrcvd.train <- roc(train.org.na$CVD_23
                       ,lrcvd.org.final.pred
                       , add = TRUE)
roc.lrcvd.train$auc
```

```
## Area under the curve: 0.848
```

```
ci(roc.lrcvd.train)
```

```
## 95% CI: 0.8241-0.8718 (DeLong)
```

```
pROC::coords(roc.lrcvd.train,
             "best",
             transpose = TRUE,
             best.method = "youden")
```

```
##   threshold specificity sensitivity
##   0.1431694   0.7349304   0.8194946
```

```
plot.roc(roc.lrcvd.train
         , print.auc = TRUE
         , print.thres = FALSE
         , auc.polygon = TRUE
)
```

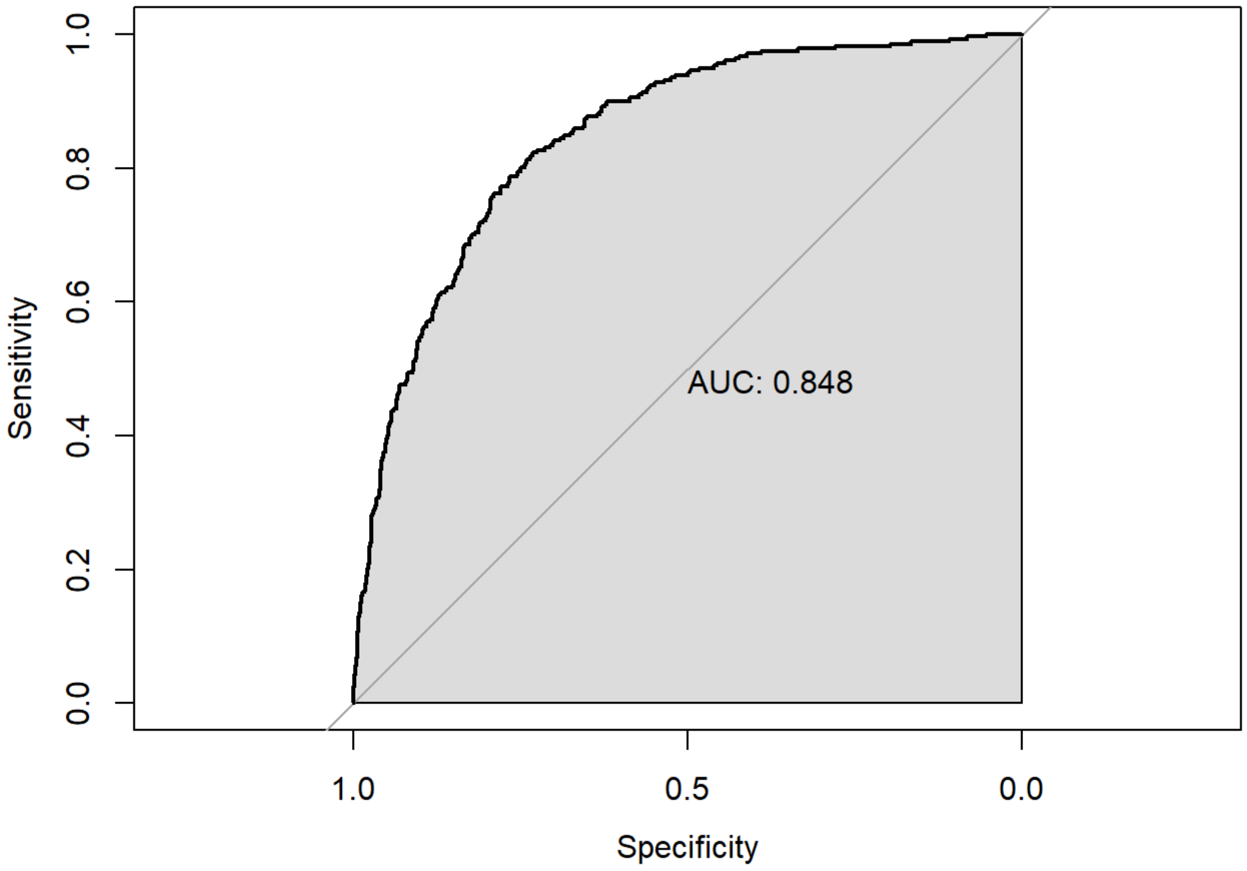

7.2 Imputed data analysis

7.2.1 Pooled logistic regression model

This is a logistic regression model with imputed datasets, results presented as “Pooled” coefficients.

```
# Logistic regression model with imput data: Enter all input parameters
lrcvd.imp.full <- with(data = train.imp
                       , exp = glm(CVD_23 ~ GENDER
                                   + CIA_AMI
                                   + CIA_CHF
                                   + CIA_PVD
                                   + CIA_CEVD
                                   + CIA_DEM
                                   + CIA_COPD
                                   + CIA_PUD
                                   + CIA_LD_MLD
                                   + CIA_LD_SEV
                                   + CIA_DM
                                   + CIA_DM_CX
                                   + CIA_HEMIPLAEGIA
                                   + CIA_CKD
                                   + CIA_CA
                                   + CIA_CA_MET
                                   + PREOP_HB
                                   + PREOP_WCC
                                   + PREOP_NA
                                   + PREOP_K
                                   + SURG_SEV
                                   + SURG_SCHEDULED_TYPE
                                   + PreopICU
                                   , family = "binomial"))

summary(pool(lrcvd.imp.full))
```

```
##                  term     estimate    std.error   statistic        df
## 1         (Intercept)  -5.11435913   3.27009846 -1.56397711  166.4743
## 2             GENDER1  -0.07448724   0.15805091 -0.47128637 1887.9487
## 3            CIA_AMI1   0.02443311   0.84484122  0.02892035 1934.1336
## 4            CIA_CHF1   1.87483705   0.30041179  6.24089047 1900.7257
## 5            CIA_PVD1   1.79175095   0.34550344  5.18591342 1925.7856
## 6           CIA_CEVD1   0.98820297   0.44561643  2.21760892 1911.4777
## 7            CIA_DEM1   0.52901015   0.27320394  1.93631959 1939.6570
## 8           CIA_COPD1  -0.43576935   0.58887950 -0.73999750 1846.4948
## 9            CIA_PUD1   0.40233535   0.53494196  0.75211028 1938.3762
## 10        CIA_LD_MLD1 -13.35727241 559.57233195 -0.02387050 1940.7996
## 11        CIA_LD_SEV1 -13.71500448 760.60407959 -0.01803173 1940.7996
## 12            CIA_DM1  -0.08217609   0.30976853 -0.26528223 1925.1212
## 13         CIA_DM_CX1   0.22167017   0.24446704  0.90674869 1897.7566
## 14    CIA_HEMIPLAEGIA1   0.02168026   0.63435290  0.03417696 1923.7210
## 15           CIA_CKD1   1.30281250   0.24305326  5.36019357 1940.4558
## 16            CIA_CA1   0.56567312   0.28175213  2.00769775 1910.4779
## 17        CIA_CA_MET1   1.95938401   0.38427214  5.09894890 1895.2396
## 18           PREOP_HB  -0.18796130   0.04221422 -4.45255903  705.2145
## 19          PREOP_WCC   0.04455633   0.01698657  2.62303327  830.1878
## 20           PREOP_NA   0.02763020   0.02173952  1.27096615  238.7640
## 21            PREOP_K  -0.04826473   0.16548631 -0.29165389  111.1126
## 22          SURG_SEV1   0.63490341   0.18100551  3.50764682 1918.6406
## 23          SURG_SEV2   1.43098783   0.23785251  6.01628222 1906.3809
## 24 SURG_SCHEDULED_TYPE1   1.14185556   0.18084158  6.31412071 1924.8674
## 25          PreopICU1   0.91934265   0.60673154  1.51523795 1935.9275
##         p.value
## 1  1.197219e-01
## 2  6.374907e-01
## 3  9.769311e-01
## 4  5.350839e-10
## 5  2.375334e-07
## 6  2.669871e-02
## 7  5.297368e-02
## 8  4.593956e-01
## 9  4.520761e-01
## 10 9.809584e-01
## 11 9.856154e-01
## 12 7.908205e-01
## 13 3.646548e-01
## 14 9.727396e-01
## 15 9.303772e-08
## 16 4.481599e-02
## 17 3.756092e-07
## 18 9.862326e-06
## 19 8.874917e-03
## 20 2.049776e-01
## 21 7.710951e-01
## 22 4.624961e-04
## 23 2.134434e-09
## 24 3.364599e-10
## 25 1.298754e-01
```

```r
# Stepwise selection method: AIC guided.
# Suppressed output
lrcvd.imp.sel <- with(data = train.imp
                      , exp = step(glm(CVD_23 ~ GENDER
                                        + CIA_AMI
                                        + CIA_CHF
                                        + CIA_PVD
                                        + CIA_CEVD
                                        + CIA_DEM
                                        + CIA_COPD
                                        + CIA_PUD
                                        + CIA_LD_MLD
                                        + CIA_LD_SEV
                                        + CIA_DM
                                        + CIA_DM_CX
                                        + CIA_HEMIPLAEGIA
                                        + CIA_CKD
                                        + CIA_CA
                                        + CIA_CA_MET
                                        + PREOP_HB
                                        + PREOP_WCC
                                        + PREOP_NA
                                        + PREOP_K
                                        + SURG_SEV
                                        + SURG_SCHEDULED_TYPE
                                        + PreopICU
                                        , family = binomial)))
```

```r
summary(pool(lrcvd.imp.sel))
```

```
##                    term    estimate  std.error statistic         df
## 1          (Intercept) -4.22620285 3.59700817 -1.174922   10.44879
## 2             CIA_CHF1  1.89423920 0.29526145  6.415464 1899.02104
## 3             CIA_PVD1  1.79013631 0.33757014  5.303006 1935.98061
## 4            CIA_CEVD1  0.95663678 0.36561250  2.616532 1876.19161
## 5             CIA_DEM1  0.52496264 0.27130742  1.934937 1949.07227
## 6             CIA_CKD1  1.35604160 0.23314264  5.816360 1933.01738
## 7              CIA_CA1  0.54809464 0.28017833  1.956235 1931.46089
## 8          CIA_CA_MET1  1.94306268 0.37870467  5.130813 1938.13717
## 9             PREOP_HB -0.19722470 0.04160723 -4.740154  591.89669
## 10           PREOP_WCC  0.04407603 0.01707850  2.580790  429.05304
## 11            PREOP_NA  0.03388670 0.02029338  1.669840 1601.09724
## 12           SURG_SEV1  0.63045125 0.17502252  3.602115 1929.65989
## 13           SURG_SEV2  1.43619718 0.23360805  6.147893 1740.24111
## 14 SURG_SCHEDULED_TYPE1  1.14713497 0.17764667  6.457397 1888.18017
## 15           PreopICU1  0.92333973 0.59445764  1.553247 1926.26071
##         p.value
## 1  2.661062e-01
## 2  1.767801e-10
## 3  1.269353e-07
## 4  8.954104e-03
## 5  5.314261e-02
## 6  7.021790e-09
## 7  5.058142e-02
## 8  3.173206e-07
## 9  2.677907e-06
## 10 1.018860e-02
## 11 9.514658e-02
## 12 3.236248e-04
## 13 9.715144e-10
## 14 1.351001e-10
## 15 1.205283e-01
```

```
# Final logistic regression model with the selected input parameters
lrcvd.imp.final <- with(data = train.imp
                        , exp = glm(CVD_23 ~ CIA_CHF
                                    + CIA_PVD
                                    + CIA_CEVD
                                    + CIA_DEM
                                    + CIA_CKD
                                    + CIA_CA
                                    + CIA_CA_MET
                                    + PREOP_HB
                                    + PREOP_WCC
                                    + PREOP_NA
                                    + SURG_SEV
                                    + SURG_SCHEDULED_TYPE
                                    + PreopICU
                                    , family = binomial))
pool(lrcvd.imp.final)
```

```
## Class: mipo    m = 5
##                     term m    estimate         ubar            b            t
## 1           (Intercept) 5 -5.54294404 8.1391667318 6.161914e-01 8.8785964049
## 2              CIA_CHF1 5  1.89113712 0.0867795951 3.404477e-04 0.0871881323
## 3              CIA_PVD1 5  1.78917601 0.1136457906 2.516471e-04 0.1139477672
## 4             CIA_CEVD1 5  0.94758389 0.1330231478 9.215283e-04 0.1341289818
## 5              CIA_DEM1 5  0.52597976 0.0737074705 3.829690e-05 0.0737534268
## 6              CIA_CKD1 5  1.34917552 0.0543766621 1.451470e-05 0.0543940798
## 7               CIA_CA1 5  0.54819083 0.0781347662 2.352767e-04 0.0784170982
## 8           CIA_CA_MET1 5  1.94672170 0.1434774199 3.246336e-04 0.1438669803
## 9              PREOP_HB 5 -0.19812073 0.0016160526 8.556108e-05 0.0017187259
## 10            PREOP_WCC 5  0.04466606 0.0002689849 1.412734e-05 0.0002859377
## 11             PREOP_NA 5  0.02977995 0.0004055687 3.418194e-05 0.0004465871
## 12            SURG_SEV1 5  0.63262960 0.0305333718 1.076517e-04 0.0306625538
## 13            SURG_SEV2 5  1.44773688 0.0540898642 3.225758e-04 0.0544769551
## 14 SURG_SCHEDULED_TYPE1 5  1.15305846 0.0314268698 1.732625e-04 0.0316347848
## 15            PreopICU1 5  0.91244526 0.3533704484 4.827682e-04 0.3539497702
##    dfcom        df          riv       lambda         fmi
## 1   1953  436.0897 0.0908483261 0.0832822711 0.087457808
## 2   1953 1921.3817 0.0047077566 0.0046856975 0.005720122
## 3   1953 1939.2074 0.0026571731 0.0026501313 0.003677158
## 4   1953 1873.3223 0.0083130945 0.0082445568 0.009301684
## 5   1953 1949.4184 0.0006234955 0.0006231070 0.001646839
## 6   1953 1950.2808 0.0003203146 0.0003202121 0.001343803
## 7   1953 1931.8086 0.0036133977 0.0036003880 0.004630360
## 8   1953 1938.8053 0.0027151334 0.0027077815 0.003734962
## 9   1953  695.7590 0.0635333851 0.0597380261 0.062429260
## 10  1953  702.4243 0.0630251173 0.0592884554 0.061955536
## 11  1953  374.0513 0.1011377958 0.0918484464 0.096665571
## 12  1953 1926.1781 0.0042308484 0.0042130238 0.005245367
## 13  1953 1890.9050 0.0071564410 0.0071055903 0.008154106
## 14  1953 1898.4453 0.0066158342 0.0065723526 0.007617271
## 15  1953 1945.2722 0.0016394178 0.0016367346 0.002661605
```

```
lrcvd.imp.final.est <- summary(pool(lrcvd.imp.final))

lrcvd.imp.final.est %>%
  mutate(OR = exp(estimate)
         , Low.CI = exp(estimate - 1.96*std.error)
         , Up.CI = exp(estimate + 1.96*std.error)
         , Sig = ifelse(p.value < 0.05, "*","")
  ) %>%
  select(-c("statistic", "df"))
```

```
##                    term    estimate  std.error      p.value          OR
## 1           (Intercept) -5.54294404 2.97969737 6.352495e-02 0.003914984
## 2              CIA_CHF1  1.89113712 0.29527637 1.890115e-10 6.626899998
## 3              CIA_PVD1  1.78917601 0.33756150 1.287831e-07 5.984519244
## 4             CIA_CEVD1  0.94758389 0.36623624 9.746232e-03 2.579469848
## 5              CIA_DEM1  0.52597976 0.27157582 5.291787e-02 1.692115912
## 6              CIA_CKD1  1.34917552 0.23322538 8.434724e-09 3.854246472
## 7               CIA_CA1  0.54819083 0.28003053 5.041947e-02 1.730120101
## 8           CIA_CA_MET1  1.94672170 0.37929801 3.146258e-07 7.005683128
## 9              PREOP_HB -0.19812073 0.04145752 2.151433e-06 0.820270818
## 10            PREOP_WCC  0.04466606 0.01690969 8.438901e-03 1.045678605
## 11             PREOP_NA  0.02977995 0.02113261 1.596085e-01 1.030227806
## 12            SURG_SEV1  0.63262960 0.17510726 3.106587e-04 1.882554435
## 13            SURG_SEV2  1.44773688 0.23340299 6.798316e-10 4.253477469
## 14 SURG_SCHEDULED_TYPE1  1.15305846 0.17786170 1.143945e-10 3.167866894
## 15            PreopICU1  0.91244526 0.59493678 1.252699e-01 2.490404788
##          Low.CI      Up.CI Sig
## 1  1.138572e-05  1.3461690
## 2  3.715062e+00 11.8210141   *
## 3  3.088098e+00 11.5975811   *
## 4  1.258299e+00  5.2878245   *
## 5  9.937110e-01  2.8813772
## 6  2.440136e+00  6.0878640   *
## 7  9.993312e-01  2.9953188
## 8  3.331083e+00 14.7338239   *
## 9  7.562544e-01  0.8897062   *
## 10 1.011590e+00  1.0809162   *
## 11 9.884275e-01  1.0737958
## 12 1.335652e+00  2.6533947   *
## 13 2.691953e+00  6.7207969   *
## 14 2.235466e+00  4.4891680   *
## 15 7.759782e-01  7.9926418
```

7.2.1.1 Object for the combined-predict probability and model diagnostics

mice::pool function does not provide the object with pooled estimates, we need a small trick to make this object. FYI, CI cannot be calculated with this trick.

```
# Pooled logistic regression model object

# Copy one of the fitted lr models
lrcvd.pool <- lrcvd.imp.final$analyses[1]

# Pooled coefficients
repl.pool <- summary(pool(lrcvd.imp.final))$estimate

# Insert name: named numeric vector objective
names(repl.pool) <- names(lrcvd.pool[[1]]$coefficients)

# Replace the fitted coefficents with the pooled estimates
lrcvd.pool[[1]]$coefficients <- repl.pool
lrcvd.imp <- lrcvd.pool[[1]]

# Check the replaced result

# Coefficient: imputed data 1
lrcvd.imp.final$analyses[1]
```

```
## [[1]]
##
## Call:  glm(formula = CVD_23 ~ CIA_CHF + CIA_PVD + CIA_CEVD + CIA_DEM +
##     CIA_CKD + CIA_CA + CIA_CA_MET + PREOP_HB + PREOP_WCC + PREOP_NA +
##     SURG_SEV + SURG_SCHEDULED_TYPE + PreopICU, family = binomial)
##
## Coefficients:
##         (Intercept)              CIA_CHF1              CIA_PVD1
##            -5.92632               1.87063               1.78894
##           CIA_CEVD1              CIA_DEM1              CIA_CKD1
##             0.97968               0.52624               1.34791
##             CIA_CA1           CIA_CA_MET1              PREOP_HB
##             0.53648               1.91566              -0.18806
##           PREOP_WCC              PREOP_NA             SURG_SEV1
##             0.04756               0.03160               0.61465
##           SURG_SEV2  SURG_SCHEDULED_TYPE1             PreopICU1
##             1.42179               1.16233               0.88206
##
## Degrees of Freedom: 1967 Total (i.e. Null);  1953 Residual
## Null Deviance:       1667
## Residual Deviance: 1198  AIC: 1228
```

```
# Coefficient: pooled model generated
lrcvd.imp
```

```
##
## Call:  glm(formula = CVD_23 ~ CIA_CHF + CIA_PVD + CIA_CEVD + CIA_DEM +
##     CIA_CKD + CIA_CA + CIA_CA_MET + PREOP_HB + PREOP_WCC + PREOP_NA +
##     SURG_SEV + SURG_SCHEDULED_TYPE + PreopICU, family = binomial)
##
## Coefficients:
##         (Intercept)              CIA_CHF1              CIA_PVD1
##            -5.54294               1.89114               1.78918
##           CIA_CEVD1              CIA_DEM1              CIA_CKD1
##             0.94758               0.52598               1.34918
##             CIA_CA1           CIA_CA_MET1              PREOP_HB
##             0.54819               1.94672              -0.19812
##           PREOP_WCC              PREOP_NA             SURG_SEV1
##             0.04467               0.02978               0.63263
##           SURG_SEV2  SURG_SCHEDULED_TYPE1             PreopICU1
##             1.44774               1.15306               0.91245
##
## Degrees of Freedom: 1967 Total (i.e. Null);  1953 Residual
## Null Deviance:       1667
## Residual Deviance: 1198  AIC: 1228
```

```
# Coefficient: estimated pooled model
summary(pool(lrcvd.imp.final))$estimate
```

```
##  [1] -5.54294404  1.89113712  1.78917601  0.94758389  0.52597976  1.34917552
##  [7]  0.54819083  1.94672170 -0.19812073  0.04466606  0.02977995  0.63262960
## [13]  1.44773688  1.15305846  0.91244526
```

Then, we can get the combined-predict probability

```
# Combined-predict probability of training dataset
lrcvd.imp.train.pred <- predict(lrcvd.imp
                                , newdata = train.org.na
                                , type = "response")
lrcvd.imp.train.class <- ifelse(lrcvd.imp.train.pred>=0.5, 1, 0)

# Confusion matrix
cl_table.lrcvd.imp.train <- table (train.org.na$CVD_23, lrcvd.imp.train.class)
rownames(cl_table.lrcvd.imp.train) <- c("CVD12", "CVD345")
colnames(cl_table.lrcvd.imp.train) <- c("CVD12", "CVD345")
addmargins(cl_table.lrcvd.imp.train)
```

```
##         lrcvd.imp.train.class
##          CVD12 CVD345  Sum
##   CVD12   1237     57 1294
##   CVD345   181     96  277
##   Sum     1418    153 1571
```

```
# Sensitivity
round(cl_table.lrcvd.imp.train[2,2]/(cl_table.lrcvd.imp.train[2,1] + cl_table.lrcvd.imp.train[2,2]),
3)
```

```
## [1] 0.347
```

```
# Specificity
round(cl_table.lrcvd.imp.train[1,1]/(cl_table.lrcvd.imp.train[1,1] + cl_table.lrcvd.imp.train[1,2]),
3)
```

```
## [1] 0.956
```

```
# Positive predictive rate
round(cl_table.lrcvd.imp.train[2,2]/(cl_table.lrcvd.imp.train[1,2] + cl_table.lrcvd.imp.train[2,2]),
3)
```

```
## [1] 0.627
```

```
# Negative predictive rate
round(cl_table.lrcvd.imp.train[1,1]/(cl_table.lrcvd.imp.train[1,1] + cl_table.lrcvd.imp.train[2,1]),
3)
```

```
## [1] 0.872
```

```
# Model accuracy (classification accuracy)
mean((train.org.na$CVD_23 == lrcvd.imp.train.class))
```

```
## [1] 0.8485041
```

```
# Discriminability
roc.lrcvd.imp.train <- roc(train.org.na$CVD_23
                           ,lrcvd.imp.train.pred
                           , add = TRUE)
```

```
## Setting levels: control = 0, case = 1
```

```
## Setting direction: controls < cases
```

```
roc.lrcvd.imp.train$auc
```

```
## Area under the curve: 0.8468
```

```
ci(roc.lrcvd.imp.train)
```

```
## 95% CI: 0.8228-0.8708 (DeLong)
```

```
plot.roc(roc.lrcvd.imp.train
        , print.auc = TRUE
        , print.thres = FALSE
        , auc.polygon = TRUE
)
```

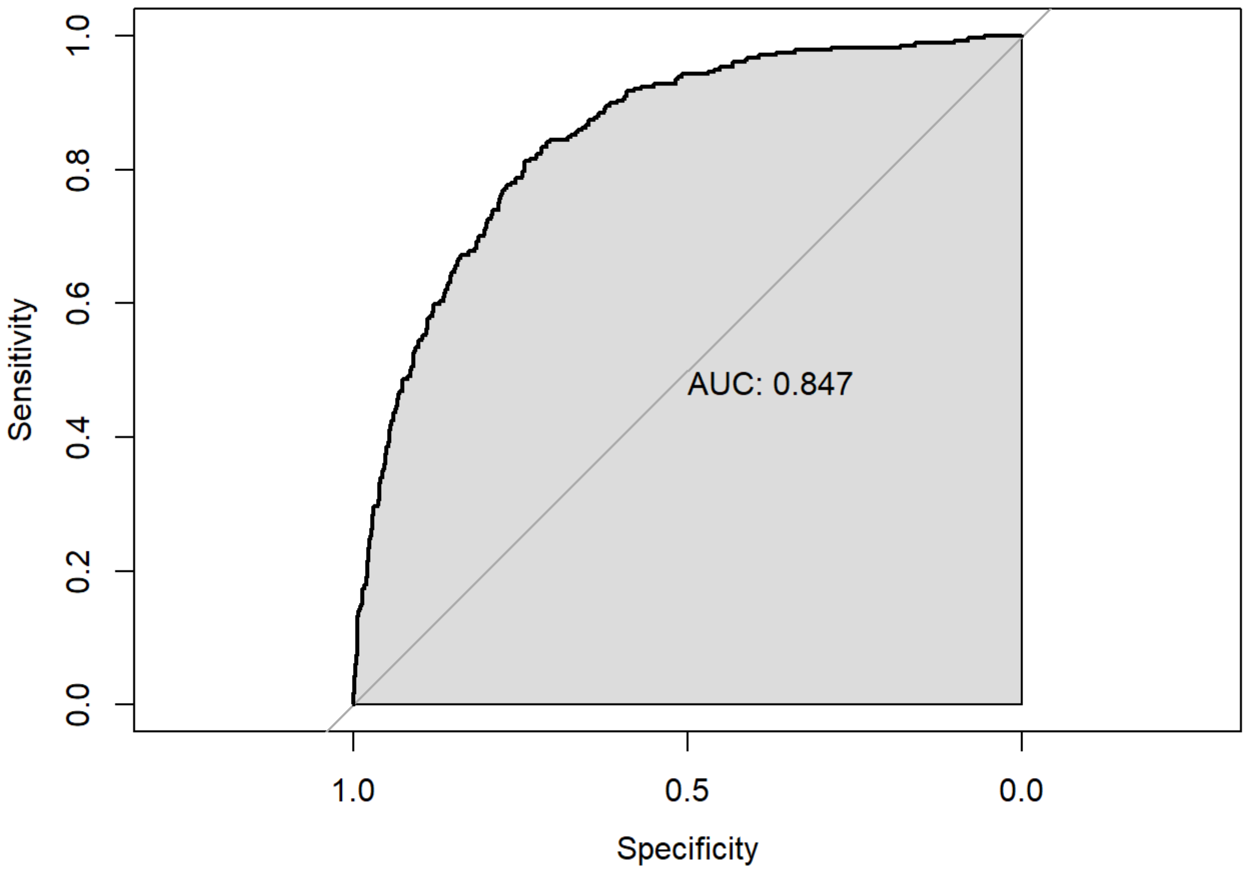


```
pROC::coords(roc.lrcvd.imp.train,
             "best",
             transpose = TRUE,
             best.method = "youden")
```

```
##   threshold specificity sensitivity
##   0.1439794   0.7434312   0.8122744
```

Using the estimated threshold from ROC (0.1439794), make a variable of assisting clinical decision of Yes/No.

```
#Threshold discrimination ability

lrcvd.imp.train.pred.dcs <- ifelse(lrcvd.imp.train.pred > 0.1439794, 1, 0)

roc.lrcvd.imp.train.dcs <- roc(train.org.na$CVD_23
                               ,lrcvd.imp.train.pred.dcs
                               , add = TRUE)
```

```
## Setting levels: control = 0, case = 1
```

```
## Setting direction: controls < cases
```

```
roc.lrcvd.imp.train.dcs$auc
```

```
## Area under the curve: 0.7779
```

```
ci(roc.lrcvd.imp.train.dcs)
```

```
## 95% CI: 0.7519-0.8038 (DeLong)
```

```
plot.roc(roc.lrcvd.imp.train.dcs
         , print.auc = TRUE
         , print.thres = FALSE
         , auc.polygon = TRUE
)
```

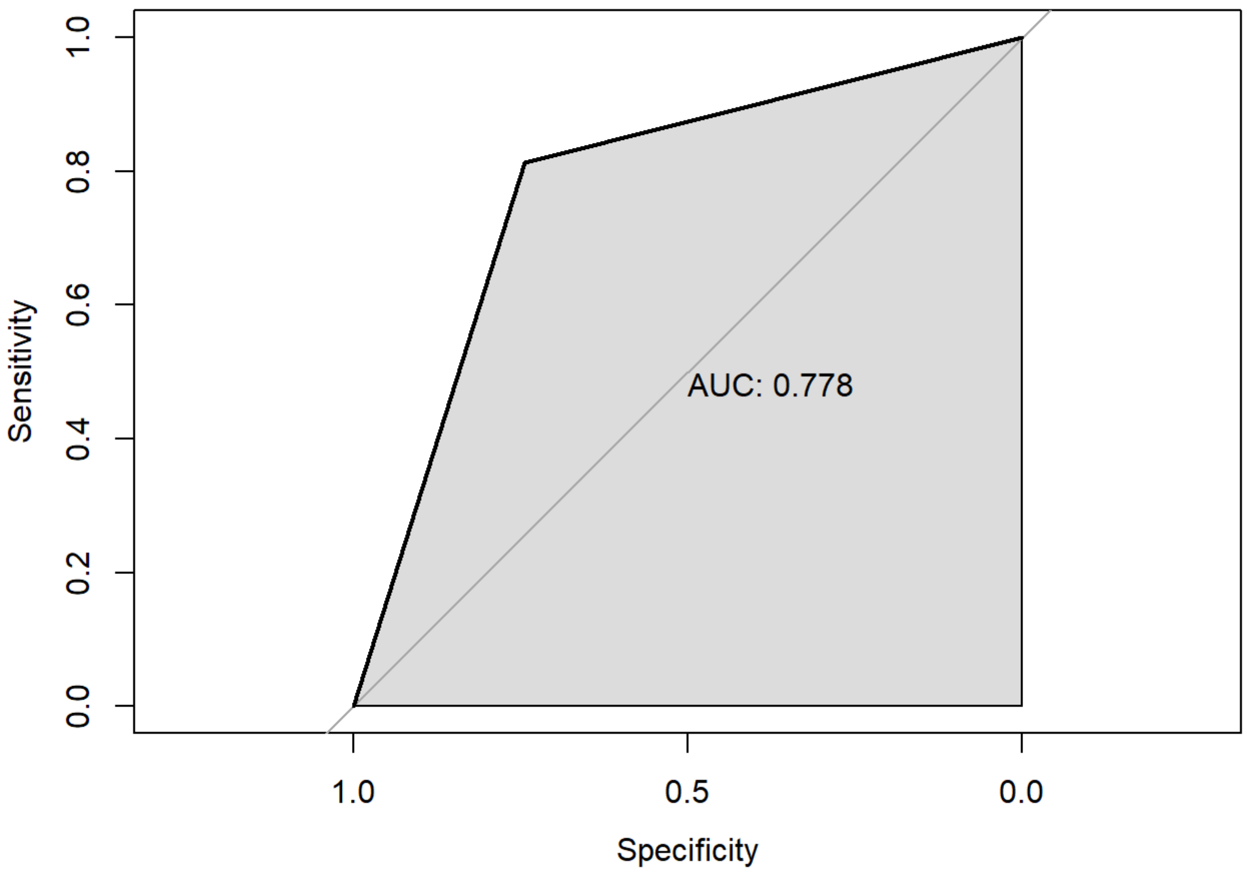

```
pROC::coords(roc.lrcvd.imp.train.dcs,
             "best",
             transpose = TRUE,
             best.method = "youden")
```

```
##   threshold specificity sensitivity
##   0.5000000   0.7434312   0.8122744
```

```
# Calibrations: on the training dataset
cal.lrcvd.imp.train <- val.prob(lrcvd.imp.train.pred
                                , train.org.na$CVD_23
                                , pl=FALSE)

CalibrationCurves::val.prob.ci.2(lrcvd.imp.train.pred, train.org.na$CVD_23
                                 , lty.smooth = 2
                                 , CL.smooth = FALSE
                                 , col.ideal = "black")
```

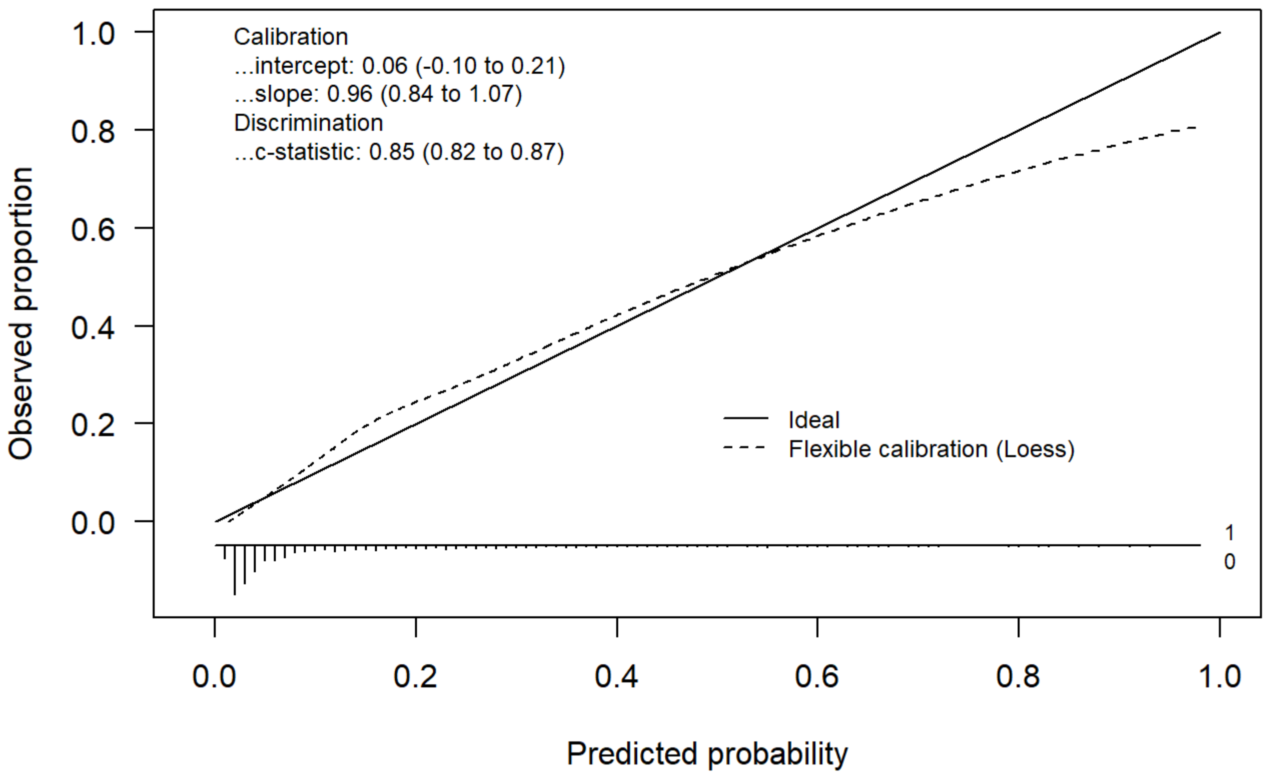

```
## Call:
## CalibrationCurves::val.prob.ci.2(p = lrcvd.imp.train.pred, y = train.org.na$CVD_23,
##     CL.smooth = FALSE, lty.smooth = 2, col.ideal = "black")
##
## A 95% confidence interval is given for the calibration intercept, calibration slope and c-statist
ic.
##
##           Dxy       C (ROC)            R2             D      D:Chi-sq
##  6.936653e-01  8.468326e-01  3.556242e-01  2.421064e-01  3.813491e+02
##           D:p             U      U:Chi-sq           U:p             Q
##  0.000000e+00 -5.940419e-04  1.066760e+00  5.866188e-01  2.427004e-01
##         Brier     Intercept         Slope          Emax  Brier scaled
##  1.076423e-01  5.623651e-02  9.558935e-01  1.050209e-02  2.588240e-01
##          Eavg           ECI
##  1.983920e-02  8.137964e-02
```

```
cal.lrcvd.imp.train
```

```
##           Dxy       C (ROC)            R2             D      D:Chi-sq
##  6.936234e-01  8.468117e-01  3.547449e-01  2.414273e-01  3.802823e+02
##           D:p             U      U:Chi-sq           U:p             Q
##            NA -5.940419e-04  1.066760e+00  5.866188e-01  2.420214e-01
##         Brier     Intercept         Slope          Emax           E90
##  1.076423e-01  2.699021e-03  9.558935e-01  1.141148e-01  3.805832e-02
##          Eavg           S:z           S:p
##  1.796871e-02  1.525485e+00  1.271383e-01
```

```
hosmer_lemeshow(train.org.na$CVD_23, lrcvd.imp.train.pred, 10, 'C')
```

```
## PVALUE 0.09593752
## stat 13.49426
```

```
## [1] 0.09593752
```

```
hosmer_lemeshow(train.org.na$CVD_23, lrcvd.imp.train.pred, 10, 'H')
```

```
## PVALUE 0.04636015
## stat 15.73332
```

```
## [1] 0.04636015
```

7.3 Sensitivity analysis

Using the delta adjusted imputation dataset, compare the estimated ORs.

```
library(finalfit)
```

```
# delta = 0
lrcvd.dlt0 <- with(data = train.imp.delta[[1]]
                   , exp = glm(CVD_23 ~ CIA_CHF
                               + CIA_PVD
                               + CIA_CEVD
                               + CIA_DEM
                               + CIA_CKD
                               + CIA_CA
                               + CIA_CA_MET
                               + PREOP_HB
                               + PREOP_WCC
                               + PREOP_NA
                               + SURG_SEV
                               + SURG_SCHEDULED_TYPE
                               + PreopICU
                               , family = binomial))

lrcvd.dlt0.est <- summary(pool(lrcvd.dlt0)
                          , conf.int = TRUE
                          , exponentiate = TRUE)

# delta = mean + 1*SD
lrcvd.dlt1 <- with(data = train.imp.delta[[2]]
                   , exp = glm(CVD_23 ~ CIA_CHF
                               + CIA_PVD
                               + CIA_CEVD
                               + CIA_DEM
                               + CIA_CKD
                               + CIA_CA
                               + CIA_CA_MET
                               + PREOP_HB
                               + PREOP_WCC
                               + PREOP_NA
                               + SURG_SEV
                               + SURG_SCHEDULED_TYPE
                               + PreopICU
                               , family = binomial))

lrcvd.dlt1.est <- summary(pool(lrcvd.dlt1)
                          , conf.int = TRUE
                          , exponentiate = TRUE)

# delta = mean + 2*SD
lrcvd.dlt2 <- with(data = train.imp.delta[[3]]
                   , exp = glm(CVD_23 ~ CIA_CHF
                               + CIA_PVD
                               + CIA_CEVD
                               + CIA_DEM
                               + CIA_CKD
                               + CIA_CA
                               + CIA_CA_MET
                               + PREOP_HB
                               + PREOP_WCC
                               + PREOP_NA
                               + SURG_SEV
                               + SURG_SCHEDULED_TYPE
                               + PreopICU
                               , family = binomial))
```

```
lrcvd.dlt2.est <- summary(pool(lrcvd.dlt2)
                          , conf.int = TRUE
                          , exponentiate = TRUE)

lrcvd.dlt0.est
```

```
##                    term    estimate  std.error statistic        df      p.value
## 1           (Intercept) 0.003914984 2.97969737 -1.860237  436.0897 6.352495e-02
## 2              CIA_CHF1 6.626899998 0.29527637  6.404634 1921.3817 1.890115e-10
## 3              CIA_PVD1 5.984519244 0.33756150  5.300296 1939.2074 1.287831e-07
## 4             CIA_CEVD1 2.579469848 0.36623624  2.587357 1873.3223 9.746232e-03
## 5              CIA_DEM1 1.692115912 0.27157582  1.936769 1949.4184 5.291787e-02
## 6              CIA_CKD1 3.854246472 0.23322538  5.784857 1950.2808 8.434724e-09
## 7               CIA_CA1 1.730120101 0.28003053  1.957611 1931.8086 5.041947e-02
## 8           CIA_CA_MET1 7.005683128 0.37929801  5.132433 1938.8053 3.146258e-07
## 9              PREOP_HB 0.820270818 0.04145752 -4.778885  695.7590 2.151433e-06
## 10            PREOP_WCC 1.045678605 0.01690969  2.641447  702.4243 8.438901e-03
## 11             PREOP_NA 1.030227806 0.02113261  1.409194  374.0513 1.596085e-01
## 12            SURG_SEV1 1.882554435 0.17510726  3.612812 1926.1781 3.106587e-04
## 13            SURG_SEV2 4.253477469 0.23340299  6.202735 1890.9050 6.798316e-10
## 14 SURG_SCHEDULED_TYPE1 3.167866894 0.17786170  6.482893 1898.4453 1.143945e-10
## 15            PreopICU1 2.490404788 0.59493678  1.533684 1945.2722 1.252699e-01
##           2.5 %     97.5 %
## 1  1.120336e-05  1.3680809
## 2  3.713747e+00 11.8252013
## 3  3.086860e+00 11.6022332
## 4  1.257732e+00  5.2902093
## 5  9.933922e-01  2.8823020
## 6  2.439464e+00  6.0895412
## 7  9.989975e-01  2.9963194
## 8  3.329582e+00 14.7404662
## 9  7.561484e-01  0.8898309
## 10 1.011532e+00  1.0809774
## 11 9.882954e-01  1.0739394
## 12 1.335372e+00  2.6539506
## 13 2.691187e+00  6.7227099
## 14 2.234983e+00  4.4901377
## 15 7.754317e-01  7.9982750
```

```
lrcvd.dlt1.est
```

```
##                    term    estimate  std.error statistic         df
## 1           (Intercept) 0.001883883 3.11342216 -2.015281  104.96956
## 2              CIA_CHF1 6.581156554 0.29677555  6.348941 1940.65444
## 3              CIA_PVD1 6.007269609 0.33543643  5.345187 1929.07084
## 4             CIA_CEVD1 3.062047289 0.36995062  3.024954 1897.67202
## 5              CIA_DEM1 1.676773430 0.26865607  1.923915 1914.69957
## 6              CIA_CKD1 4.095251142 0.23321562  6.045170 1930.53946
## 7               CIA_CA1 1.696037196 0.27797473  1.900513 1950.47518
## 8           CIA_CA_MET1 6.541990339 0.37997202  4.943105 1948.01401
## 9              PREOP_HB 0.912811853 0.02070511 -4.405940 1867.57616
## 10            PREOP_WCC 1.038324540 0.01633317  2.302578   92.43659
## 11             PREOP_NA 1.028302869 0.02213032  1.261154  107.33141
## 12            SURG_SEV1 1.725858861 0.17366086  3.142474 1935.22849
## 13            SURG_SEV2 3.555785428 0.22655484  5.599421 1939.16545
## 14 SURG_SCHEDULED_TYPE1 3.131598178 0.17717375  6.443073 1780.11130
## 15            PreopICU1 2.221070303 0.58969712  1.353219 1925.62005
##         p.value        2.5 %     97.5 %
## 1  4.643117e-02 3.926422e-06  0.9038805
## 2  2.692435e-10 3.677297e+00 11.7781121
## 3  1.010291e-07 3.111529e+00 11.5979274
## 4  2.520083e-03 1.482205e+00  6.3257993
## 5  5.451336e-02 9.900321e-01  2.8398768
## 6  1.786983e-09 2.592045e+00  6.4702129
## 7  5.751320e-02 9.832771e-01  2.9254644
## 8  8.346896e-07 3.105103e+00 13.7829998
## 9  1.113005e-05 8.764873e-01  0.9506418
## 10 2.354674e-02 1.005185e+00  1.0725569
## 11 2.099876e-01 9.841672e-01  1.0744178
## 12 1.700634e-03 1.227700e+00  2.4261530
## 13 2.457366e-08 2.280191e+00  5.5449779
## 14 1.502918e-10 2.212345e+00  4.4328110
## 15 1.761446e-01 6.987077e-01  7.0603967
```

```
lrcvd.dlt2.est
```

```
##                    term     estimate  std.error statistic         df
## 1           (Intercept) 0.0005723142 3.23702505 -2.306384   62.98648
## 2              CIA_CHF1 6.6366449496 0.29715421  6.369106 1942.30147
## 3              CIA_PVD1 6.0752546834 0.33562843  5.375659 1944.75902
## 4             CIA_CEVD1 3.0115515813 0.36922072  2.985898 1938.07032
## 5              CIA_DEM1 1.6734746158 0.26805846  1.920857 1945.87346
## 6              CIA_CKD1 4.1367312272 0.23347278  6.081677 1943.89712
## 7               CIA_CA1 1.6891656965 0.27812574  1.884884 1947.54690
## 8           CIA_CA_MET1 6.5910324017 0.38017371  4.960127 1946.64094
## 9              PREOP_HB 0.9179738622 0.01988570 -4.303914  432.75833
## 10            PREOP_WCC 1.0447848261 0.01563793  2.801583  389.63801
## 11             PREOP_NA 1.0362382802 0.02317760  1.535842   59.48254
## 12            SURG_SEV1 1.7198685712 0.17360025  3.123543 1949.92473
## 13            SURG_SEV2 3.5607303398 0.22743836  5.583780 1909.28915
## 14 SURG_SCHEDULED_TYPE1 3.1240735037 0.17682327  6.442239 1900.92294
## 15            PreopICU1 2.1433534638 0.58938878  1.293495 1946.41793
##         p.value        2.5 %     97.5 %
## 1  2.439145e-02 8.877923e-07  0.3689416
## 2  2.367151e-10 3.705551e+00 11.8862382
## 3  8.548041e-08 3.145568e+00 11.7335611
## 4  2.862897e-03 1.459865e+00  6.2125222
## 5  5.489566e-02 9.892484e-01  2.8309547
## 6  1.428237e-09 2.616984e+00  6.5390327
## 7  5.959428e-02 9.790029e-01  2.9144763
## 8  7.656437e-07 3.127142e+00 13.8918226
## 9  2.077029e-05 8.827874e-01  0.9545629
## 10 5.338911e-03 1.013151e+00  1.0774059
## 11 1.298798e-01 9.892845e-01  1.0854205
## 12 1.813140e-03 1.223587e+00  2.4174408
## 13 2.690703e-08 2.279399e+00  5.5623430
## 14 1.487571e-10 2.208580e+00  4.4190547
## 15 1.959933e-01 6.746725e-01  6.8091766
```

```
explanatory.cvd = c("CIA_CHF", "CIA_PVD", "CIA_CEVD", "CIA_DEM", "CIA_CKD"
                   , "CIA_CA", "CIA_CA_MET", "PREOP_HB", "PREOP_WCC"
                   , "PREOP_NA", "SURG_SEV", "SURG_SCHEDULED_TYPE", "PreopICU")

complete(train.imp.delta[[1]]) %>%
  or_plot("CVD_23", explanatory.cvd
          , glmfit = pool(lrcvd.dlt0)
          , confint_type = "profile"
          , table_text_size = 3
          , title_text_size = 10
          , dependent_label = "Any complication: delta = 0")
```

Any complication: delta = 0: OR (95% CI, p-value)		
CIA_CHF	0	-
	1	6.63 (3.71-11.83, p<0.001)
CIA_PVD	0	-
	1	5.98 (3.09-11.60, p<0.001)
CIA_CEVD	0	-
	1	2.58 (1.26-5.29, p=0.010)
CIA_DEM	0	-
	1	1.69 (0.99-2.88, p=0.053)
CIA_CKD	0	-
	1	3.85 (2.44-6.09, p<0.001)
CIA_CA	0	-
	1	1.73 (1.00-3.00, p=0.050)
CIA_CA_MET	0	-
	1	7.01 (3.33-14.74, p<0.001)
PREOP_HB	[4.3,18.2]	0.82 (0.76-0.89, p<0.001)
PREOP_WCC	[1.4,82.5]	1.05 (1.01-1.08, p=0.008)
PREOP_NA	[120.0,155.0]	1.03 (0.99-1.07, p=0.160)
SURG_SEV	0	-
	1	1.88 (1.34-2.65, p<0.001)
	2	4.25 (2.69-6.72, p<0.001)
SURG_SCHEDULED_TYPE	0	-
	1	3.17 (2.23-4.49, p<0.001)
PreopICU	0	-
	1	2.49 (0.78-8.00, p=0.125)

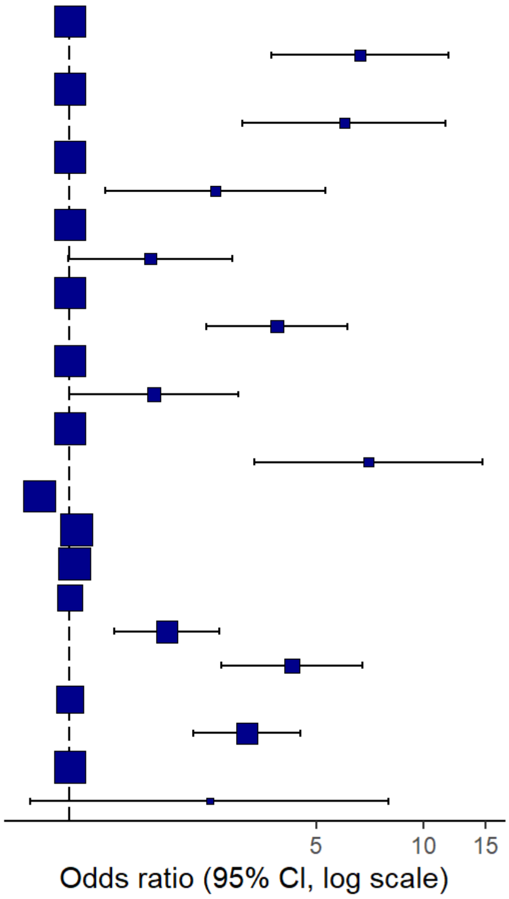


```
complete(train.imp.delta[[2]]) %>%
  or_plot("CVD_23", explanatory.cvd
          , glmfit = pool(lrcvd.dlt1)
          , confint_type = "profile"
          , table_text_size = 3
          , title_text_size = 10
          , dependent_label = "Any complication: delta = m + 1*SD")
```

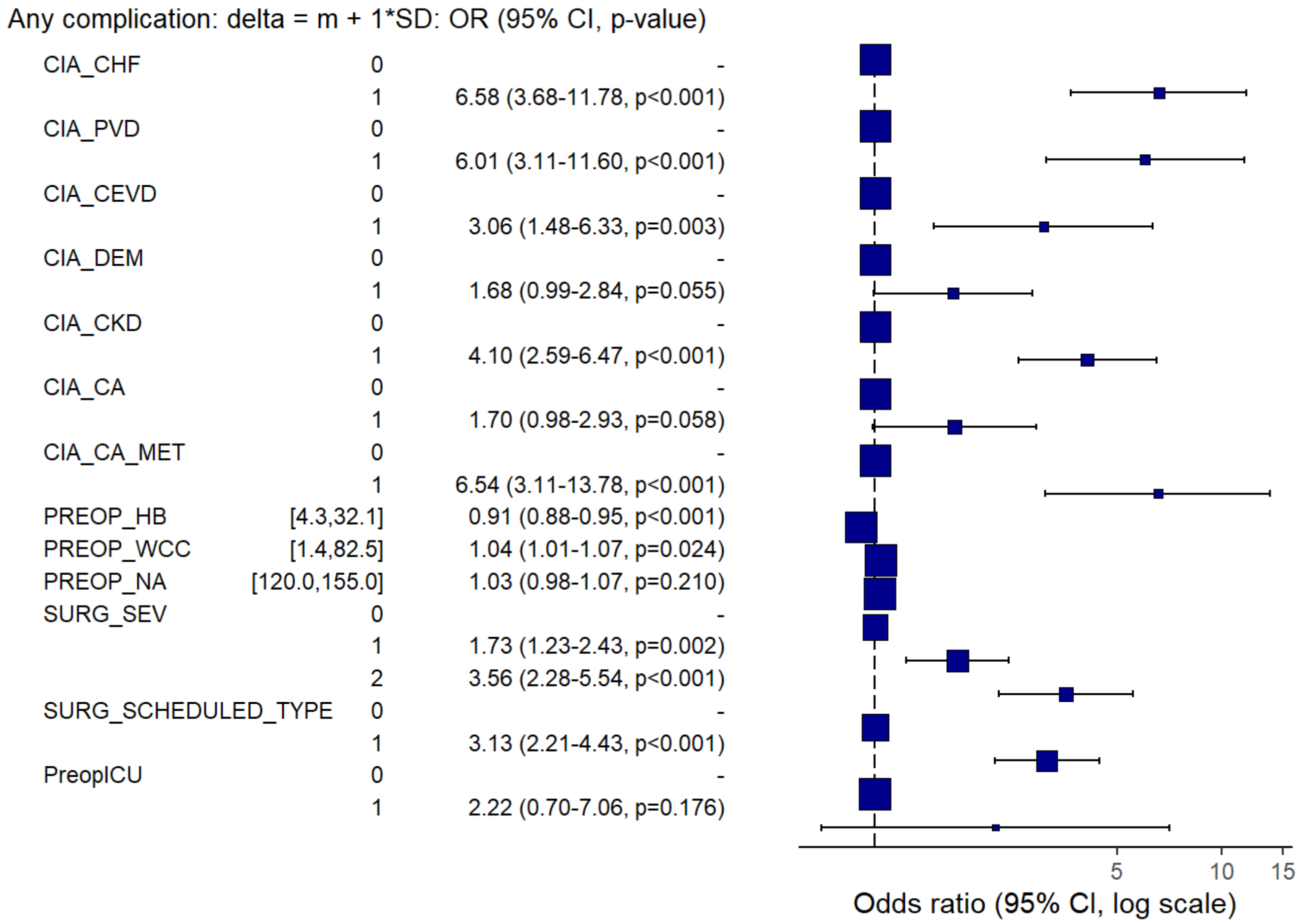


```
complete(train.imp.delta[[3]]) %>%
  or_plot("CVD_23", explanatory.cvd
          , glmfit = pool(lrcvd.dlt2)
          , confint_type = "profile"
          , table_text_size = 3
          , title_text_size = 10
          , dependent_label = "Any complication: delta = m + 2*SD")
```

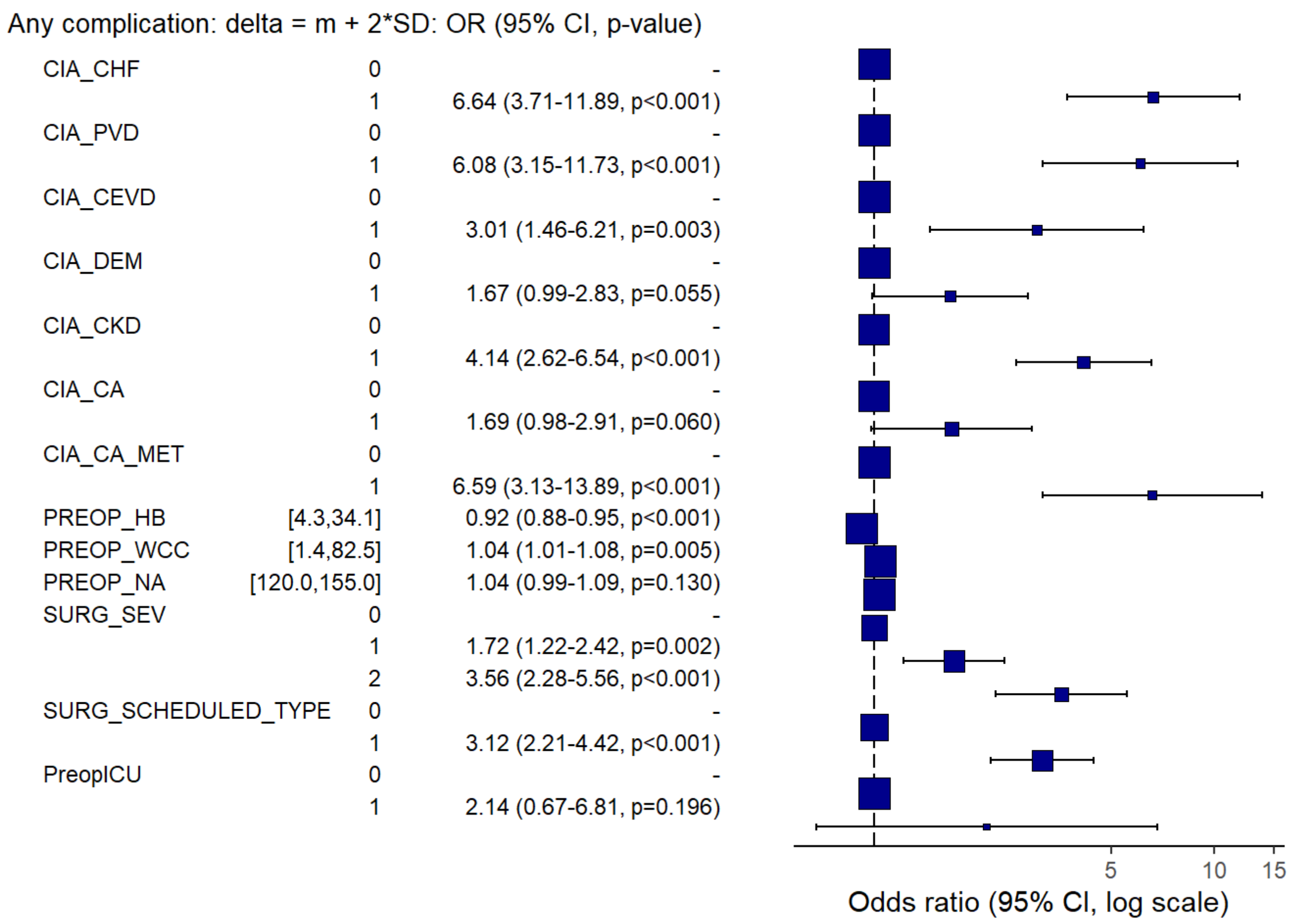

7.4 Model testing

We performed the model testing using the testing dataset for original and pooled imputed models.
Model testing has three components.
First, model discrimination ability using ROC curve analysis.
Second, regarding the threshold estimated from the training dataset, ROC analysis on testing dataset. This process assess the ability of the clinical dichromotous decision making such as “Yes” or “No” risk of complications.
Third, calibrations measurement as usual.

7.4.1 Original model testing

```
# Predicted probability and classification
lrcvd.org.test.pred <- predict(lrcvd.org.final
                               , newdata = test.org.na
                               , type = "response")
lrcvd.org.test.class <- ifelse(lrcvd.org.test.pred>=0.5, 1, 0)

# Confusion matrix
cl_table.lrcvd.org.test <- table (test.org.na$CVD_23, lrcvd.org.test.class)
rownames(cl_table.lrcvd.org.test) <- c("CVD12", "CVD345")
colnames(cl_table.lrcvd.org.test) <- c("CVD12", "CVD345")
addmargins(cl_table.lrcvd.org.test)
```

```
##         lrcvd.org.test.class
##          CVD12 CVD345 Sum
##   CVD12    348      9 357
##   CVD345    43     16  59
##   Sum      391     25 416
```

```
# Model accuracy (classification accuracy)
mean((test.org.na$CVD_23 == lrcvd.org.test.class))
```

```
## [1] 0.875
```

```
# Discriminability
roc.lrcvd.org.test <- roc(test.org.na$CVD_23
                          ,lrcvd.org.test.pred
                          , add = TRUE)
```

```
## Setting levels: control = 0, case = 1
```

```
## Setting direction: controls < cases
```

```
roc.lrcvd.org.test$auc
```

```
## Area under the curve: 0.8811
```

```
ci(roc.lrcvd.org.test)
```

```
## 95% CI: 0.8371-0.9252 (DeLong)
```

```
plot.roc(roc.lrcvd.org.test
         , print.auc = TRUE
         , print.thres = FALSE
         , auc.polygon = TRUE
)
```

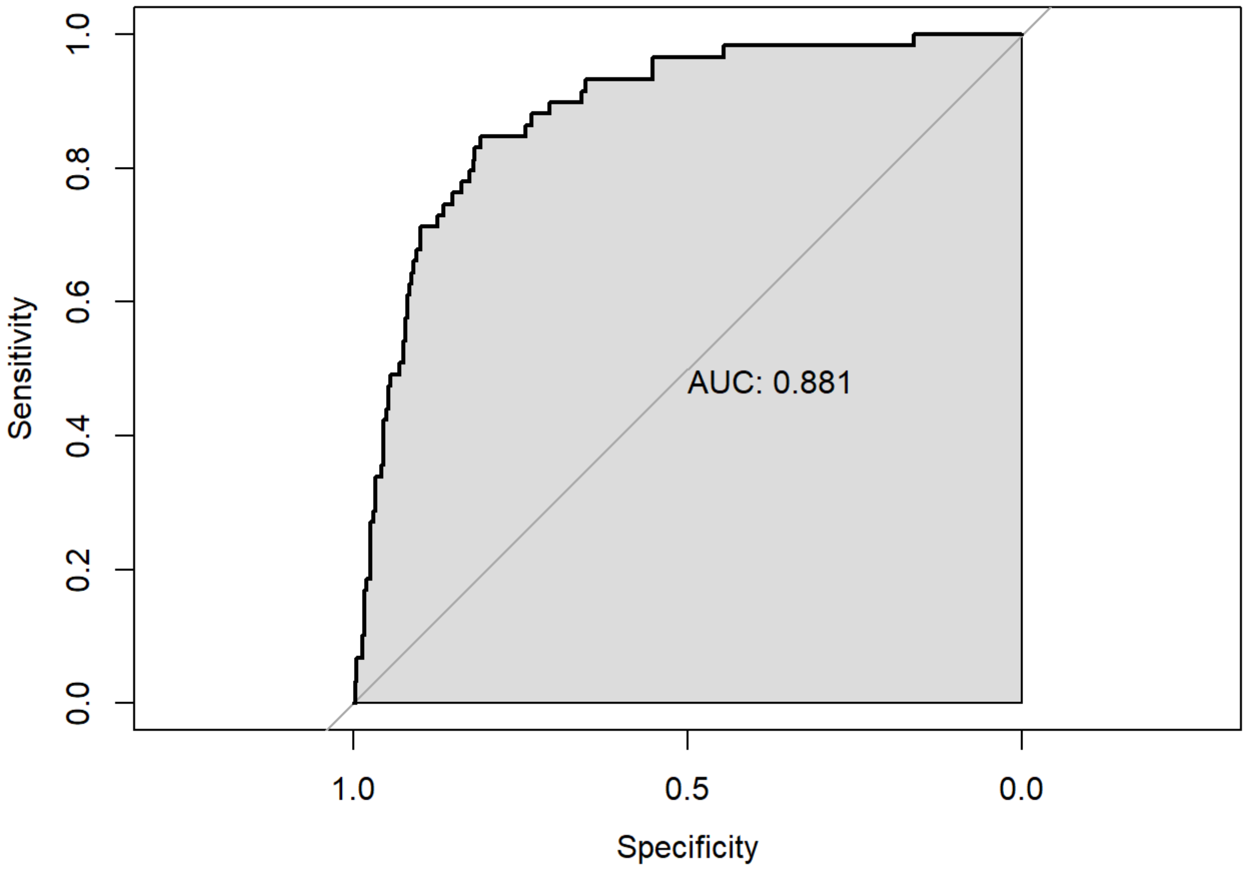


```
pROC::coords(roc.lrcvd.org.test,
             "best",
             transpose = TRUE,
             best.method = "youden")
```

```
##   threshold specificity sensitivity
##   0.1682365   0.8095238   0.8474576
```

```
#Threshold discrimination ability

lrcvd.org.test.pred.dcs <- ifelse(lrcvd.org.test.pred > 0.1439794, 1, 0)

roc.lrcvd.org.test.dcs <- roc(test.org.na$CVD_23
                              ,lrcvd.org.test.pred.dcs
                              , add = TRUE)
```

```
## Setting levels: control = 0, case = 1
```

```
## Setting direction: controls < cases
```

```
roc.lrcvd.org.test.dcs$auc
```

```
## Area under the curve: 0.8103
```

```
ci(roc.lrcvd.org.test.dcs)
```

```
## 95% CI: 0.7592-0.8614 (DeLong)
```

```
plot.roc(roc.lrcvd.org.test.dcs
         , print.auc = TRUE
         , print.thres = FALSE
         , auc.polygon = TRUE
)
```

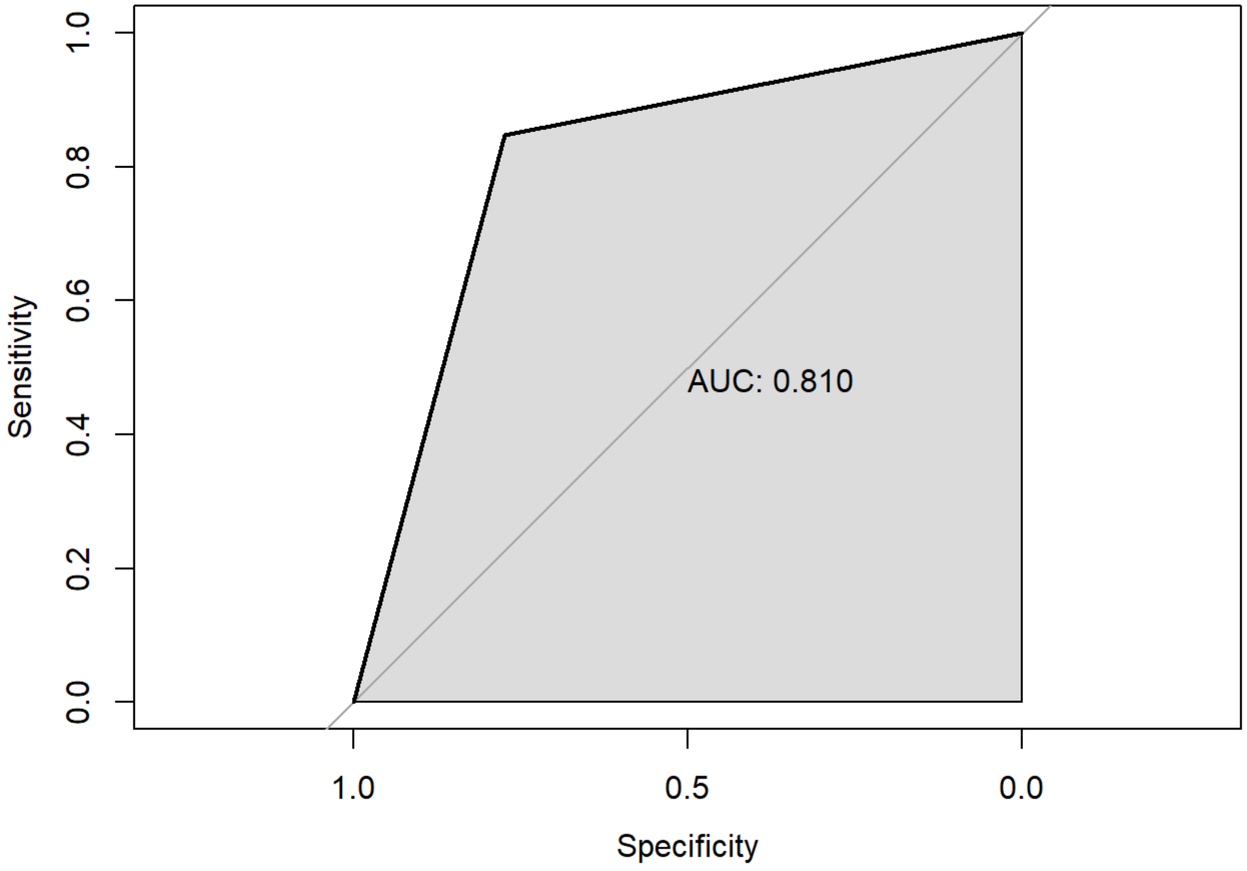


```
pROC::coords(roc.lrcvd.org.test.dcs,
             "best",
             transpose = TRUE,
             best.method = "youden")
```

```
##   threshold specificity sensitivity
##   0.5000000   0.7731092   0.8474576
```

7.4.2 Imputed model Testing

7.4.2.1 Testing with the imputted result.

```
# Predicted probability and classification: pooled model
lrcvd.imp.test.pred <- as.data.frame(predict(lrcvd.imp, test.org.na, type = "response"))
lrcvd.imp.test.class <- ifelse(lrcvd.imp.test.pred>=0.5, 1, 0)

# Confusion matrix
cl_table.lrcvd.imp.test <- table (test.org.na$CVD_23, lrcvd.imp.test.class)
rownames(cl_table.lrcvd.imp.test) <- c("CVD12", "CVD345")
colnames(cl_table.lrcvd.imp.test) <- c("CVD12", "CVD345")
addmargins(cl_table.lrcvd.imp.test)
```

```
##         lrcvd.imp.test.class
##          CVD12 CVD345 Sum
##   CVD12    348      9 357
##   CVD345    43     16  59
##   Sum      391     25 416
```

```
# Sensitivity
round(cl_table.lrcvd.imp.test[2,2]/(cl_table.lrcvd.imp.test[2,1] + cl_table.lrcvd.imp.test[2,2]),3)
```

```
## [1] 0.271
```

```
# Specificity
round(cl_table.lrcvd.imp.test[1,1]/(cl_table.lrcvd.imp.test[1,1] + cl_table.lrcvd.imp.test[1,2]),3)
```

```
## [1] 0.975
```

```
# Positive predictive rate
round(cl_table.lrcvd.imp.test[2,2]/(cl_table.lrcvd.imp.test[1,2] + cl_table.lrcvd.imp.test[2,2]),3)
```

```
## [1] 0.64
```

```
# Negative predictive rate
round(cl_table.lrcvd.imp.test[1,1]/(cl_table.lrcvd.imp.test[1,1] + cl_table.lrcvd.imp.test[2,1]),3)
```

```
## [1] 0.89
```

```
#model accuracy (classification accuracy)
mean((test.org.na$CVD_23 == lrcvd.imp.test.class))
```

```
## [1] 0.875
```

```
#Discriminability

roc.lrcvd.imp.test <- roc(test.org.na$CVD_23
                         , lrcvd.imp.test.pred[,1]
                         , add = TRUE)
```

```
## Setting levels: control = 0, case = 1
```

```
## Setting direction: controls < cases
```

```
roc.lrcvd.imp.test$auc
```

```
## Area under the curve: 0.8812
```

```
ci(roc.lrcvd.imp.test)
```

```
## 95% CI: 0.8369-0.9255 (DeLong)
```

```
pROC::coords(roc.lrcvd.imp.test
             , "best"
             , transpose = TRUE
             , best.method="youden"
)
```

```
##   threshold specificity sensitivity
##   0.1608411   0.8011204   0.8474576
```

```
plot.roc(roc.lrcvd.imp.test
         , print.auc = TRUE
         , print.thres = FALSE
         , auc.polygon = TRUE
)
```

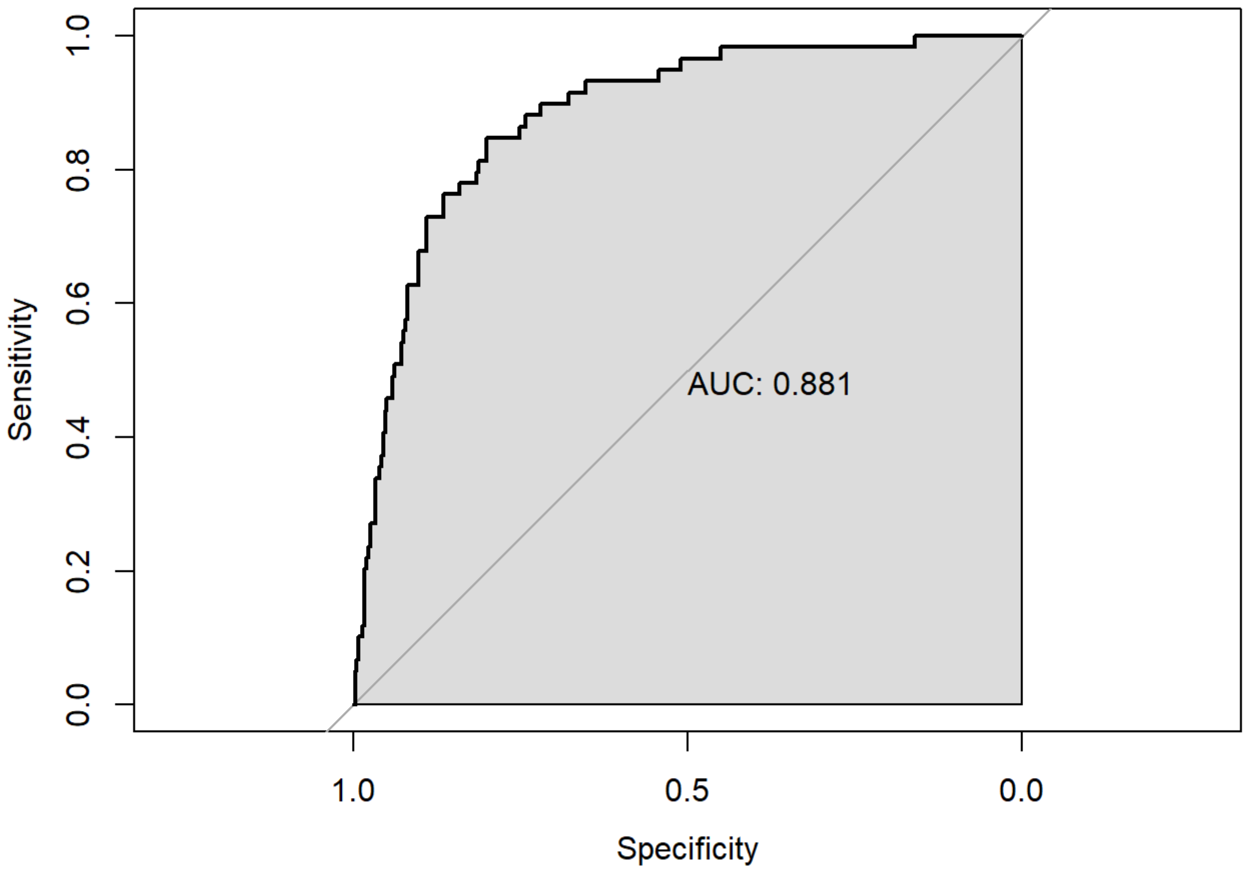


```
# Threshold discrimination ability

lrcvd.imp.test.pred.dcs <- ifelse(lrcvd.imp.test.pred[,1] > 0.1439794, 1, 0)

roc.lrcvd.imp.test.dcs <- roc(test.org.na$CVD_23
                              ,lrcvd.imp.test.pred.dcs
                              , add = TRUE)
```

```
## Setting levels: control = 0, case = 1
## Setting direction: controls < cases
```

```
roc.lrcvd.imp.test.dcs$auc
```

```
## Area under the curve: 0.8117
```

```
ci(roc.lrcvd.imp.test.dcs)
```

```
## 95% CI: 0.7606-0.8628 (DeLong)
```

```
plot.roc(roc.lrcvd.imp.test.dcs
         , print.auc = TRUE
         , print.thres = FALSE
         , auc.polygon = TRUE
)
```

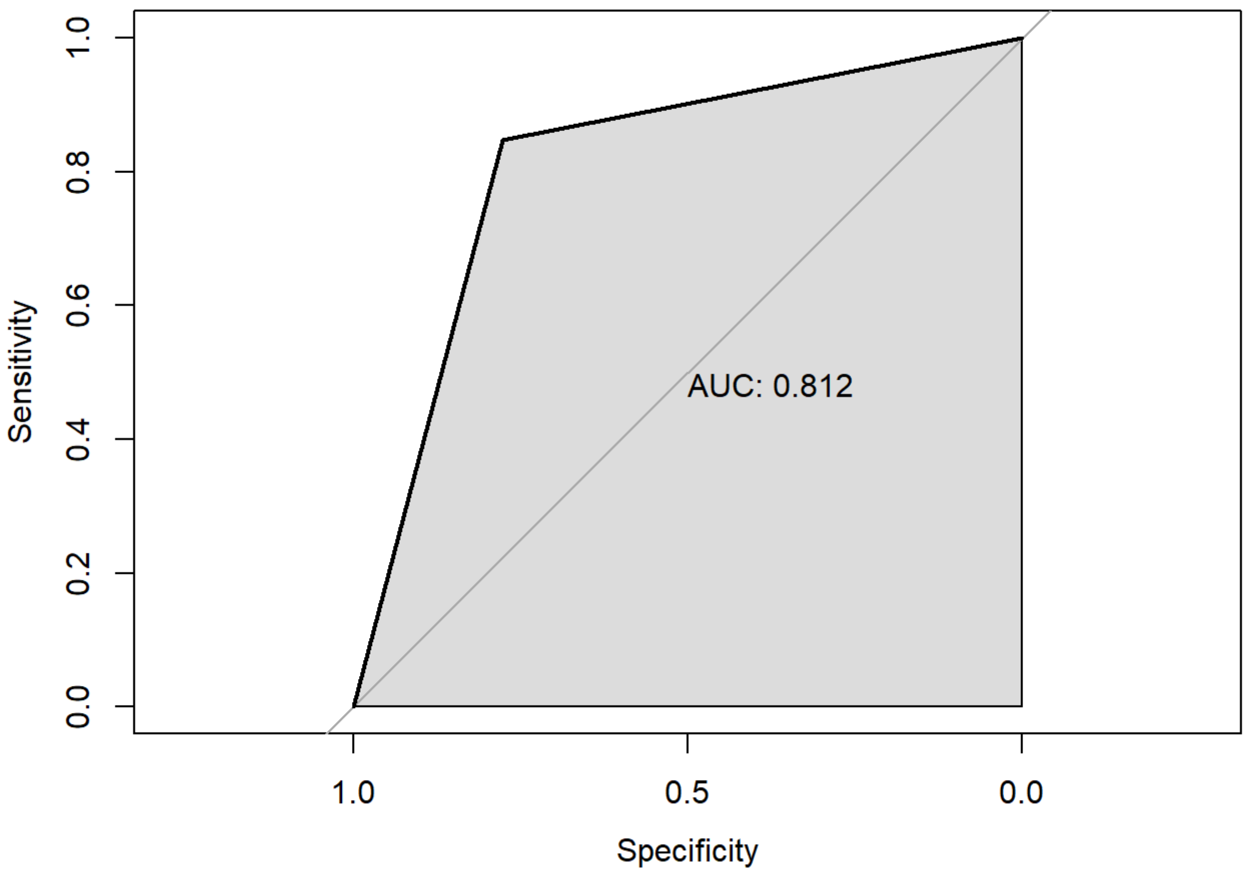


```
pROC::coords(roc.lrcvd.imp.test.dcs,
             "best",
             transpose = TRUE,
             best.method = "youden")
```

```
##   threshold specificity sensitivity
##   0.5000000   0.7759104   0.8474576
```

```
# Calibrations: on the testing dataset
cal.lrcvd.imp.test <- val.prob(lrcvd.imp.test.pred[,1]
                                , test.org.na$CVD_23
                                , pl = FALSE)

cal.lrcvd.imp.test
```

```
##          Dxy      C (ROC)           R2            D     D:Chi-sq          D:p
##  0.762521958  0.881260979  0.378371307  0.234769788 98.664231709           NA
##            U     U:Chi-sq          U:p            Q        Brier    Intercept
## -0.002178069  1.093923483  0.578705399  0.236947856  0.088624385  0.157154318
##        Slope         Emax          E90         Eavg          S:z          S:p
##  1.141956118  0.228111407  0.082673083  0.031001952 -0.515591152  0.606139991
```

```
hosmer_lemeshow(test.org.na$CVD_23, lrcvd.imp.test.pred[,1], 10, 'C')
```

```
## PVALUE 0.555794
## stat 6.823464
```

```
## [1] 0.555794
```

```
hosmer_lemeshow(test.org.na$CVD_23, lrcvd.imp.test.pred[,1], 10, 'H')
```

```
## PVALUE 3.022748e-06
## stat 40.13553
```

```
## [1] 3.022748e-06
```

Calibration interpretations & decision of re-calibration requirement

Brier score: 0.089 <<1
Spiegelhalter Z-test P = 0.606: Fail to reject the null hypothesis
Hosmer-Lemeshow C & H test P values = 0.556 & <0.001 : appropriate for C, not for H (misscalibrated).

I try out the re-calibration with testing dataset to see how it works and to gain experience.

7.5 Recalibrations using testing dataset

Recalibration: Isotonic regression model

```
# predicted probability testing dataset with pooled logistic regression
lrcvd.imp.test.pred.prob <- predict(lrcvd.imp,
                                    test.org.na[,-length(test.org.na)],
                                    type = "response")
lrcvd.imp.iso.data = cbind(y=test.org.na$CVD_23
                          , yhat=lrcvd.imp.test.pred.prob)
lrcvd.imp.iso.data.order = lrcvd.imp.iso.data[order(lrcvd.imp.iso.data[,2]),]

# create calibration model: isocvd.imp
isocvd.imp.wostepfunc <- isoreg(lrcvd.imp.iso.data.order[,2], lrcvd.imp.iso.data.order[,1])
isocvd.imp.stepf_data = cbind(isocvd.imp.wostepfunc$x, isocvd.imp.wostepfunc$yf)
isocvd.imp = stepfun(isocvd.imp.stepf_data[,1], c(0,isocvd.imp.stepf_data[,2]))

# Predicted probability of isotonic regression on testing dataset. Imputed model

isocvd.imp.pred <- isocvd.imp(as.data.frame(lrcvd.imp.test.pred.prob)[,1])
```

Recalibration: Platt scailing

```
pltcvd.imp <- glm(y~yhat, as.data.frame(lrcvd.imp.iso.data), family=binomial)
ygrid_norm = as.data.frame(lrcvd.imp.test.pred)
colnames(lrcvd.imp.test.pred) <- c("yhat")



# Predicted probability of platt scailing
pltcvd.imp.pred <- as.data.frame(predict(pltcvd.imp, lrcvd.imp.test.pred, type = "response" ))
```

7.5.1 Discriminations & Calibration measurements of recalibrated models

7.5.1.1 Discrimination

```
# Discrimination ability: Isoregression model

roc.isocvd.test.imp <- roc(test.org.na$CVD_23
                           , isocvd.imp.pred
                           , add = TRUE)
```

```
## Setting levels: control = 0, case = 1
```

```
## Setting direction: controls < cases
```

```
roc.isocvd.test.imp$auc
```

```
## Area under the curve: 0.8936
```

```
ci(roc.isocvd.test.imp)
```

```
## 95% CI: 0.8542-0.933 (DeLong)
```

```
pROC::coords(roc.isocvd.test.imp
             , "best"
             , transpose = TRUE
             , best.method="youden"
)
```

```
##   threshold specificity sensitivity
##   0.1361607   0.8011204   0.8474576
```

```
plot.roc(roc.isocvd.test.imp
         , print.auc = TRUE
         , print.thres = FALSE
         , auc.polygon = TRUE
)
```

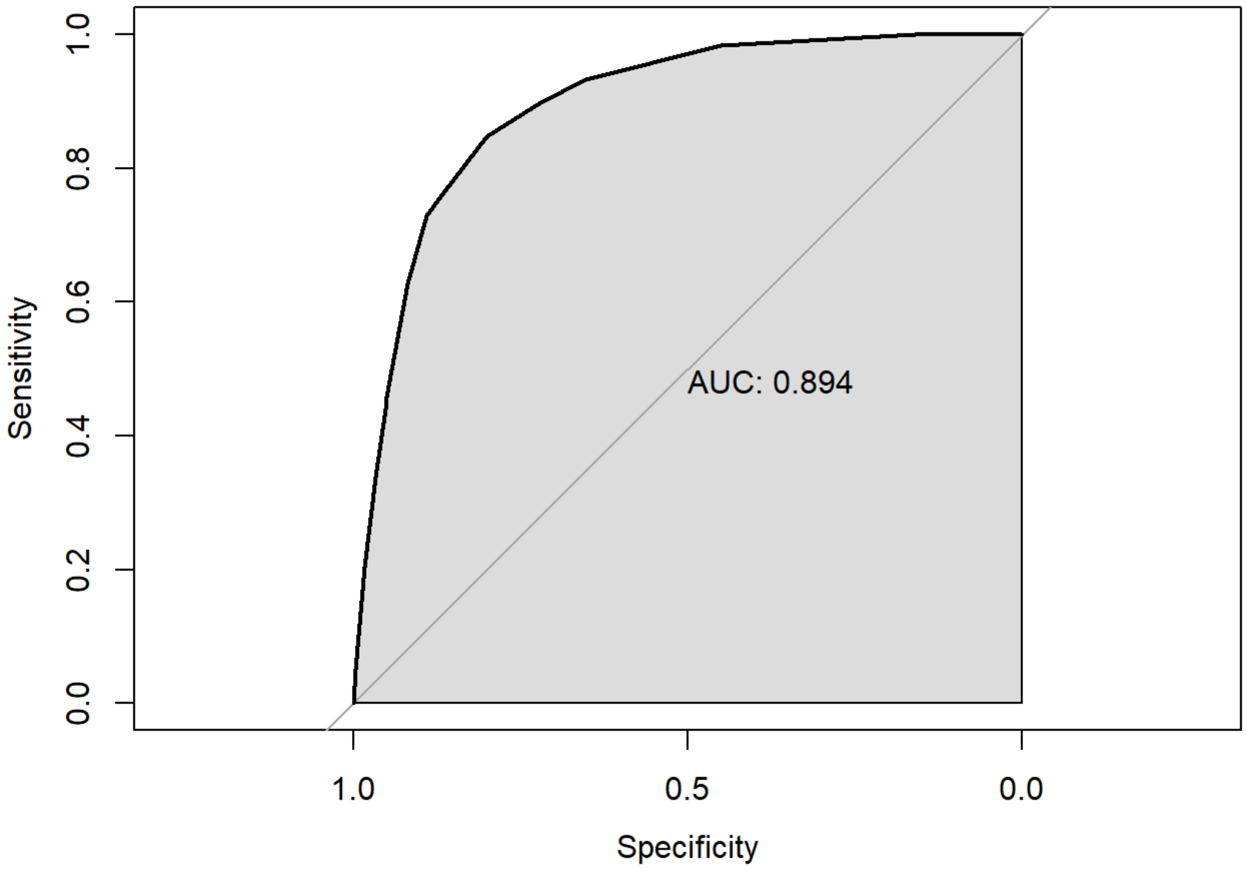


```
# Discrimination ability: Platt Scaling model
roc.pltcvd.test.imp <- roc(test.org.na$CVD_23
                           , pltcvd.imp.pred[,1]
                           , add = TRUE)
```

```
## Setting levels: control = 0, case = 1
## Setting direction: controls < cases
```

```
roc.pltcvd.test.imp$auc
```

```
## Area under the curve: 0.8812
```

```
ci(roc.pltcvd.test.imp)
```

```
## 95% CI: 0.8369-0.9255 (DeLong)
```

```
pROC::coords(roc.pltcvd.test.imp
             , "best"
             , transpose = TRUE
             , best.method="youden"
)
```

```
##   threshold specificity sensitivity
##   0.1092775   0.8011204   0.8474576
```

```
plot.roc(roc.pltcvd.test.imp
         , print.auc = TRUE
         , print.thres = FALSE
         , auc.polygon = TRUE
)
```

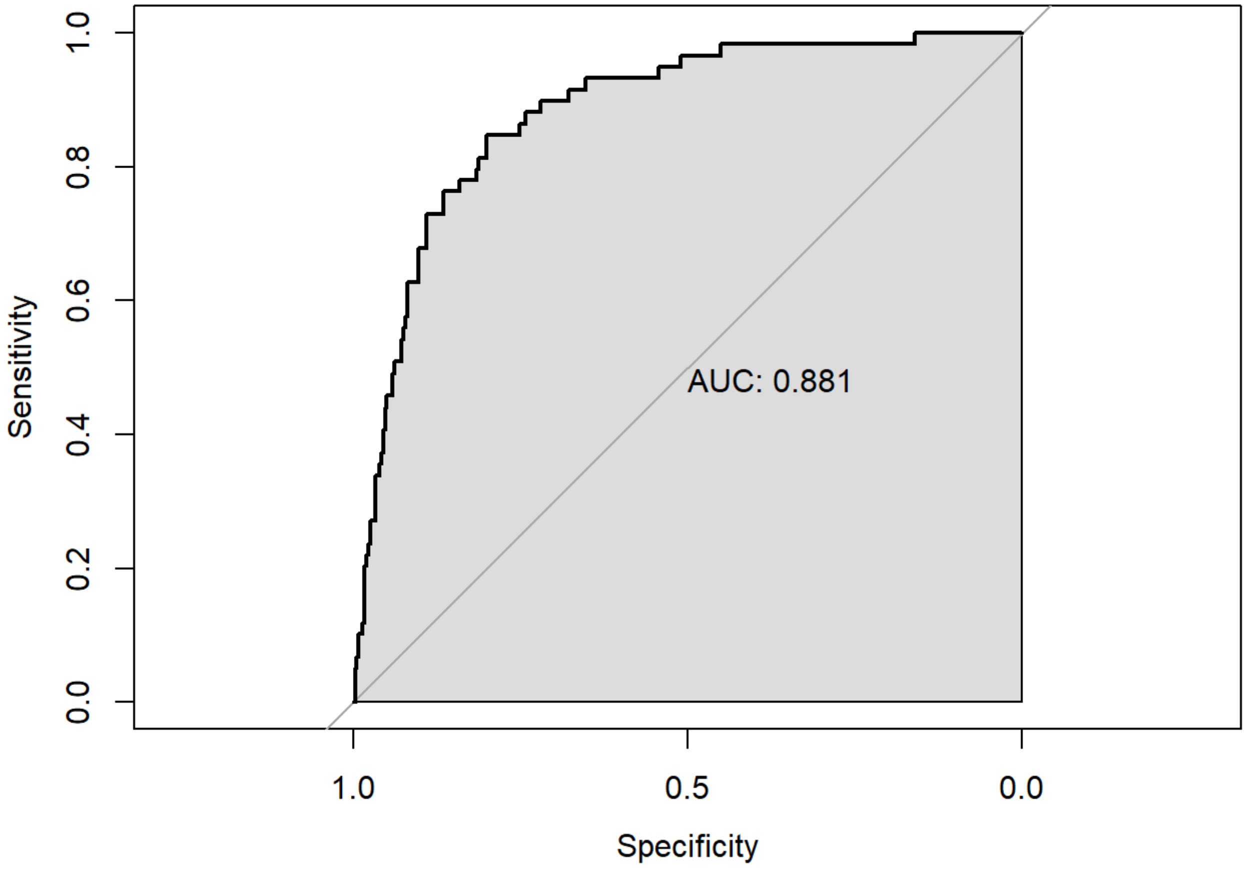

7.5.2 Threshold discrimination ability: Treshold from lrmodel with training data

```
# Isoregression model

isocvd.imp.test.pred.dcs <- ifelse(isocvd.imp.pred > 0.1439794, 1, 0)

roc.isocvd.imp.test.dcs <- roc(test.org.na$CVD_23
                              ,isocvd.imp.test.pred.dcs
                              , add = TRUE)
```

```
## Setting levels: control = 0, case = 1
```

```
## Setting direction: controls < cases
```

```
roc.isocvd.imp.test.dcs$auc
```

```
## Area under the curve: 0.8243
```

```
ci(roc.isocvd.imp.test.dcs)
```

```
## 95% CI: 0.7736-0.875 (DeLong)
```

```
plot.roc(roc.isocvd.imp.test.dcs
         , print.auc = TRUE
         , print.thres = FALSE
         , auc.polygon = TRUE
)
```

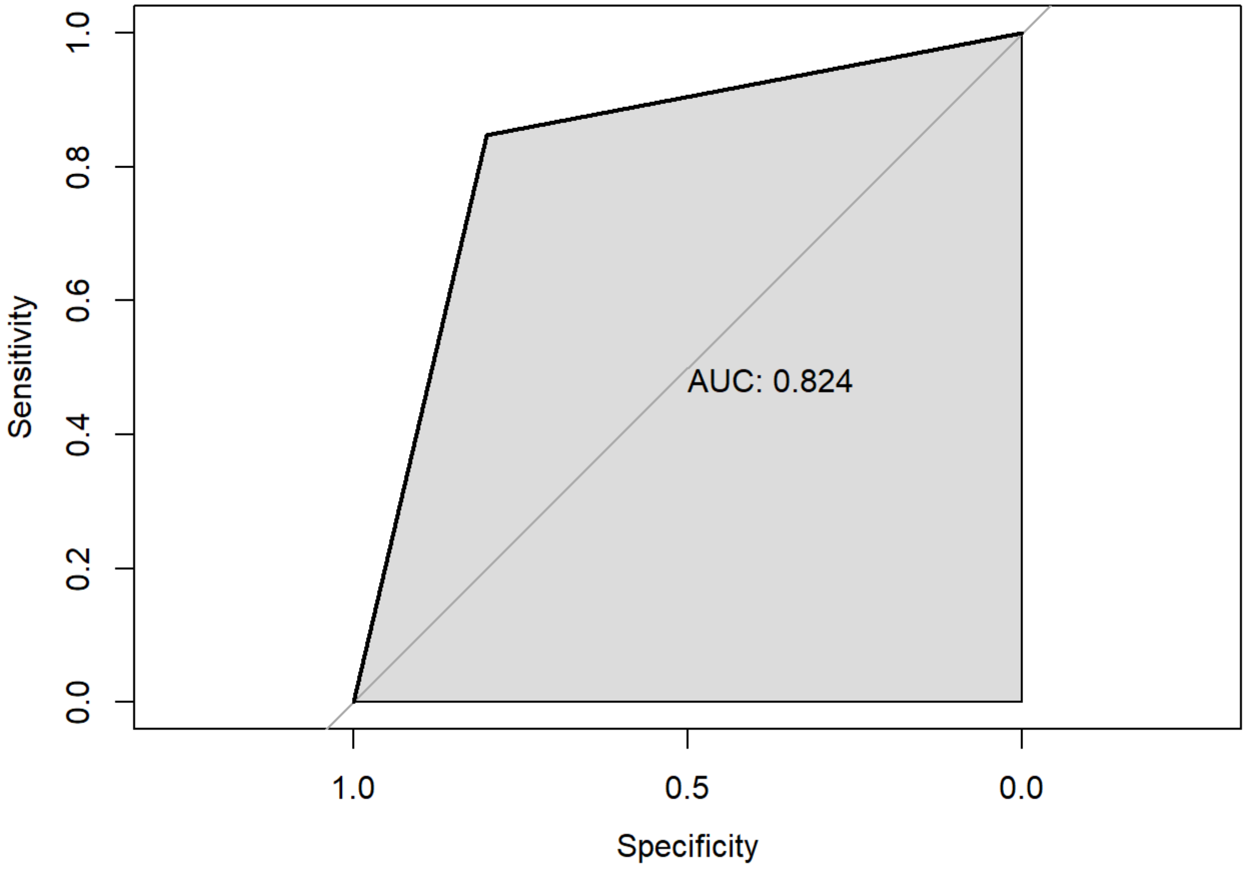


```
pROC::coords(roc.isocvd.imp.test.dcs,
             "best",
             transpose = TRUE,
             best.method = "youden")
```

```
##   threshold specificity sensitivity
##   0.5000000   0.8011204   0.8474576
```

```
# Platt Scailing model


pltcvd.imp.test.pred.dcs <- ifelse(pltcvd.imp.pred > 0.1439794, 1, 0)

roc.pltcvd.imp.test.dcs <- roc(test.org.na$CVD_23
                               ,pltcvd.imp.test.pred.dcs
                               , add = TRUE)
```

```
## Setting levels: control = 0, case = 1
## Setting direction: controls < cases
```

```
roc.pltcvd.imp.test.dcs$auc
```

```
## Area under the curve: 0.8015
```

```
ci(roc.pltcvd.imp.test.dcs)
```

```
## 95% CI: 0.7436-0.8595 (DeLong)
```

```
plot.roc(roc.pltcvd.imp.test.dcs
         , print.auc = TRUE
         , print.thres = FALSE
         , auc.polygon = TRUE
)
```

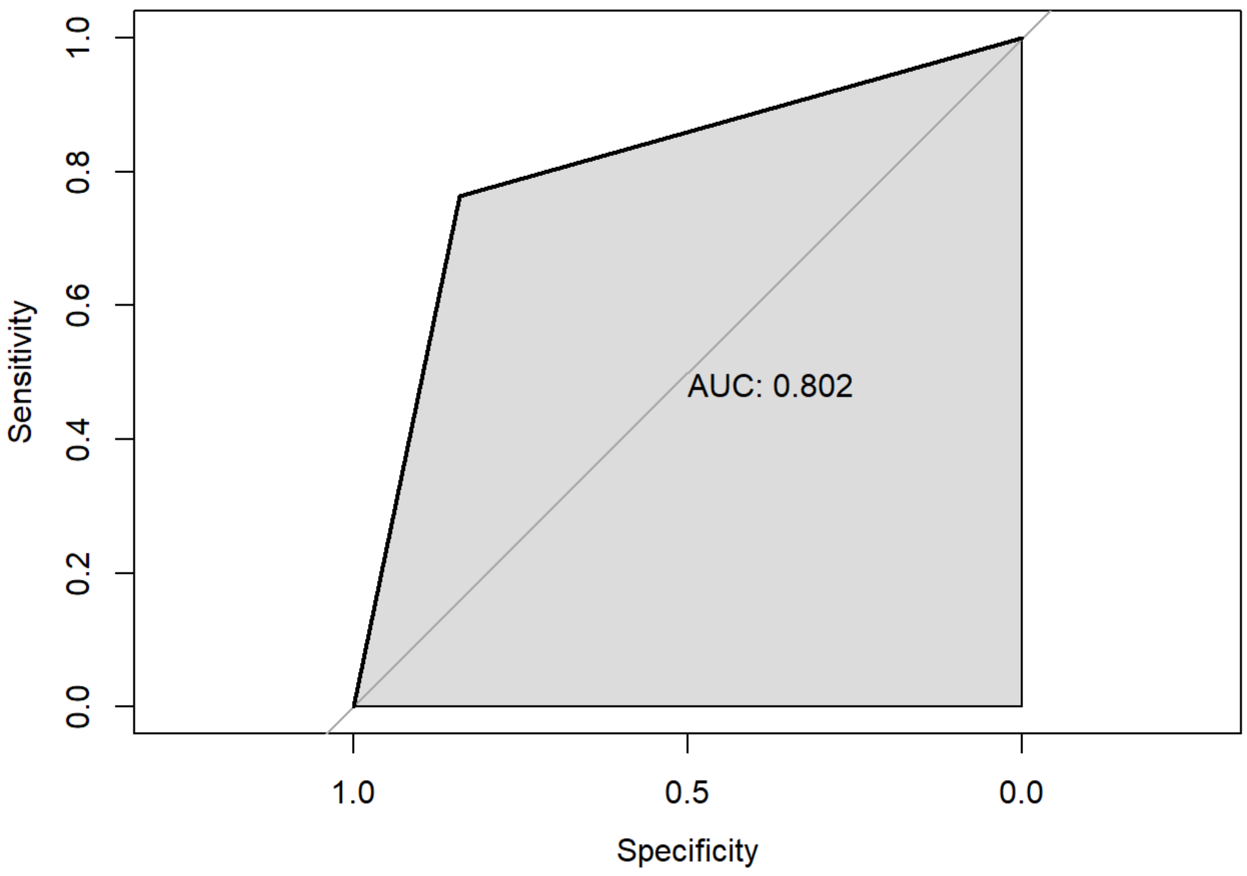


```
pROC::coords(roc.pltcvd.imp.test.dcs,
             "best",
             transpose = TRUE,
             best.method = "youden")
```

```
##   threshold specificity sensitivity
##   0.5000000   0.8403361   0.7627119
```

7.5.3 Calibrations of recalibrated models

```
cal.isocvd.imp.test <- val.prob(isocvd.imp.pred
                               , test.org.na$CVD_23
                               , pl = FALSE)


cal.pltcvd.imp.test <- val.prob(pltcvd.imp.pred[,1]
                               , test.org.na$CVD_23
                               , pl = FALSE)


cbind(cal.lrcvd.imp.test, cal.isocvd.imp.test, cal.pltcvd.imp.test)
```

```
##           cal.lrcvd.imp.test cal.isocvd.imp.test cal.pltcvd.imp.test
## Dxy              0.762521958        7.467232e-01        7.623321e-01
## C (ROC)          0.881260979        8.733616e-01        8.811660e-01
## R2               0.378371307        4.241360e-01        3.399381e-01
## D                0.234769788        2.856836e-01        2.079450e-01
## D:Chi-sq        98.664231709        1.035604e+02        8.750514e+01
## D:p                       NA                  NA                  NA
## U               -0.002178069       -5.571031e-03       -4.807692e-03
## U:Chi-sq         1.093923483       -1.250555e-12        0.000000e+00
## U:p              0.578705399        1.000000e+00        1.000000e+00
## Q                0.236947856        2.912546e-01        2.127527e-01
## Brier            0.088624385        8.121902e-02        9.168694e-02
## Intercept        0.157154318       -1.591813e-08       -9.789013e-11
## Slope            1.141956118        9.999999e-01        1.000000e+00
## Emax             0.228111407        4.218847e-15        3.101143e-01
## E90              0.082673083        8.743006e-16        1.369336e-01
## Eavg             0.031001952        3.649091e-16        5.243355e-02
## S:z             -0.515591152       -3.461175e-17        4.692902e-01
## S:p              0.606139991        1.000000e+00        6.388623e-01
```

```
hosmer_lemeshow(test.org.na$CVD_23, lrcvd.imp.test.pred[,1], 10, 'C')
```

```
## PVALUE 0.555794
## stat 6.823464
```

```
## [1] 0.555794
```

```
hosmer_lemeshow(test.org.na$CVD_23, isocvd.imp.pred, 10, 'C')
```

```
## PVALUE 0.975992
## stat 2.15205
```

```
## [1] 0.975992
```

```
hosmer_lemeshow(test.org.na$CVD_23, pltcvd.imp.pred[,1], 10, 'C')
```

```
## PVALUE 0.1378644
## stat 12.31084
```

```
## [1] 0.1378644
```

```
hosmer_lemeshow(test.org.na$CVD_23, lrcvd.imp.test.pred[,1], 10, 'H')
```

```
## PVALUE 3.022748e-06
## stat 40.13553
```

```
## [1] 3.022748e-06
```

```
hosmer_lemeshow(test.org.na$CVD_23, isocvd.imp.pred, 10, 'H')
```

```
## PVALUE 1
## stat 1.577722e-31
```

```
## [1] 1
```

```
hosmer_lemeshow(test.org.na$CVD_23, pltcvd.imp.pred[,1], 10, 'H')
```

```
## PVALUE 0.0003376886
## stat 28.84421
```

```
## [1] 0.0003376886
```

```
CalibrationCurves::val.prob.ci.2(lrcvd.imp.test.pred[,1], test.org.na$CVD_23
                                 , lty.smooth = 2
                                 , CL.smooth = FALSE
                                 , col.ideal = "black")
```

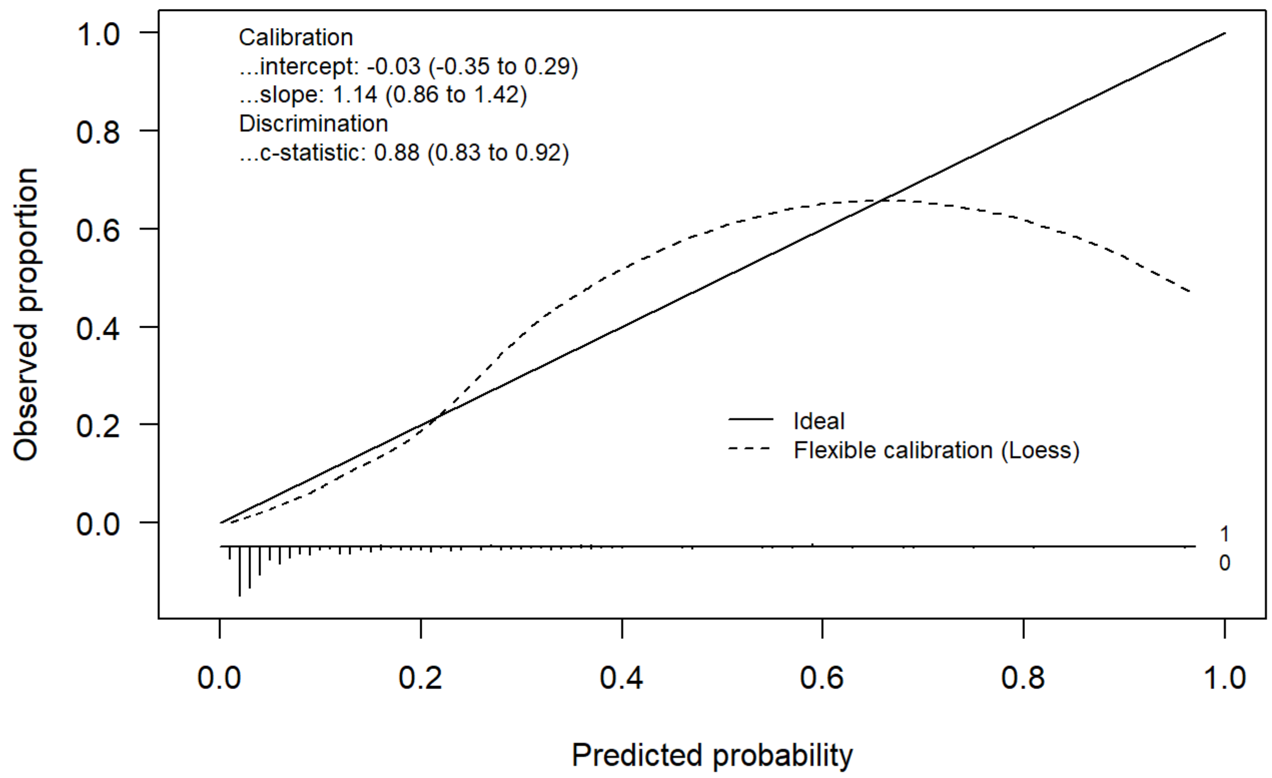


```
## Call:
## CalibrationCurves::val.prob.ci.2(p = lrcvd.imp.test.pred[, 1],
##     y = test.org.na$CVD_23, CL.smooth = FALSE, lty.smooth = 2,
##     col.ideal = "black")
##
## A 95% confidence interval is given for the calibration intercept, calibration slope and c-statist
ic.
##
##           Dxy      C (ROC)           R2            D     D:Chi-sq          D:p
##  0.762474481  0.881237241  0.382083714  0.237399412 99.758155192  0.000000000
##            U      U:Chi-sq          U:p            Q        Brier    Intercept
## -0.002178069  1.093923483  0.578705399  0.239577480  0.088624385 -0.030300787
##        Slope          Emax Brier scaled         Eavg          ECI
##  1.141956118  0.054887484  0.271851985  0.036163812  0.330379413
```

```
CalibrationCurves::val.prob.ci.2(isocvd.imp.pred, test.org.na$CVD_23
                                 , lty.smooth = 2
                                 , CL.smooth = FALSE
                                 , col.ideal = "black")
```

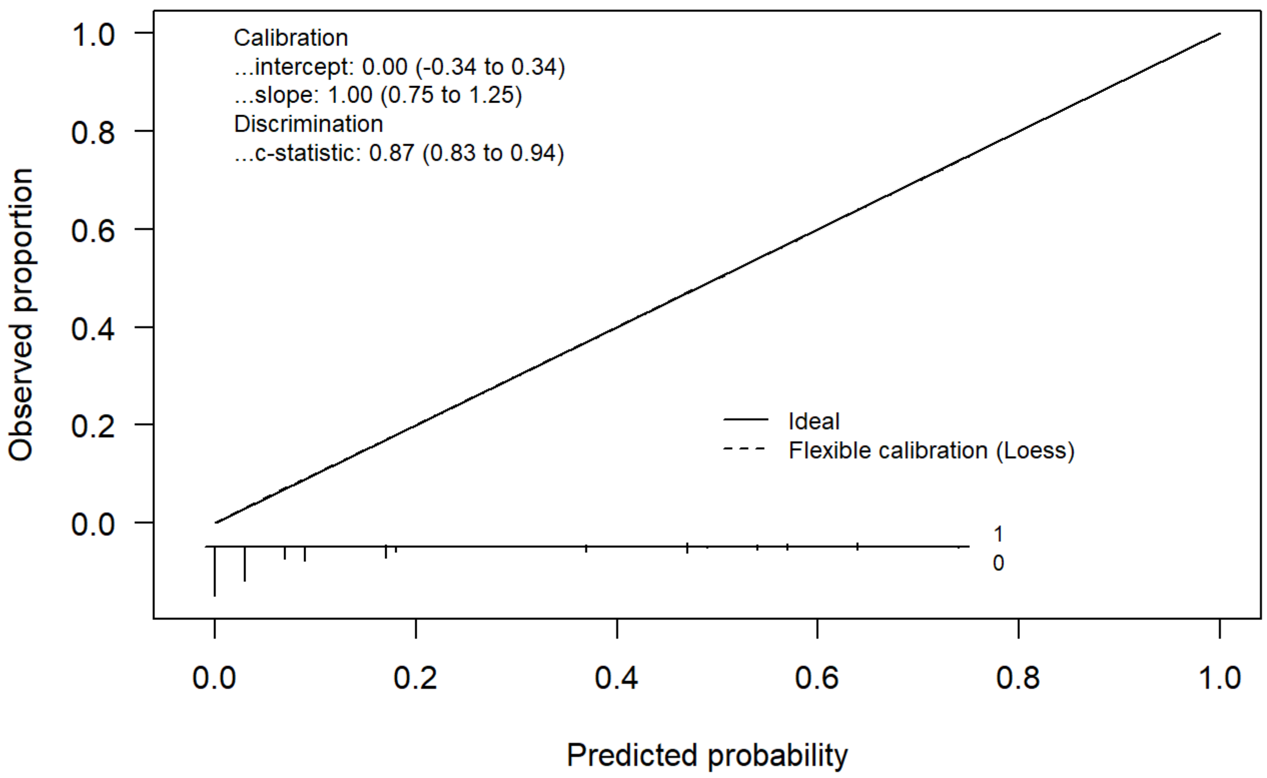


```
## Call:
## CalibrationCurves::val.prob.ci.2(p = isocvd.imp.pred, y = test.org.na$CVD_23,
##     CL.smooth = FALSE, lty.smooth = 2, col.ideal = "black")
##
## A 95% confidence interval is given for the calibration intercept, calibration slope and c-statist
ic.
##
##           Dxy       C (ROC)            R2             D      D:Chi-sq
##  7.467232e-01  8.733616e-01  4.241360e-01  2.856836e-01  1.035604e+02
##           D:p             U      U:Chi-sq           U:p             Q
##  0.000000e+00 -5.571031e-03 -1.080025e-12  1.000000e+00  2.912546e-01
##         Brier     Intercept         Slope          Emax  Brier scaled
##  9.411452e-02  7.609752e-15  9.999999e-01  2.933630e-08  2.267444e-01
##          Eavg           ECI
##  7.854232e-16  3.624594e-28
```

```
CalibrationCurves::val.prob.ci.2(pltcvd.imp.pred[,1], test.org.na$CVD_23
                                  , lty.smooth = 2
                                  , CL.smooth = FALSE
                                  , col.ideal = "black")
```

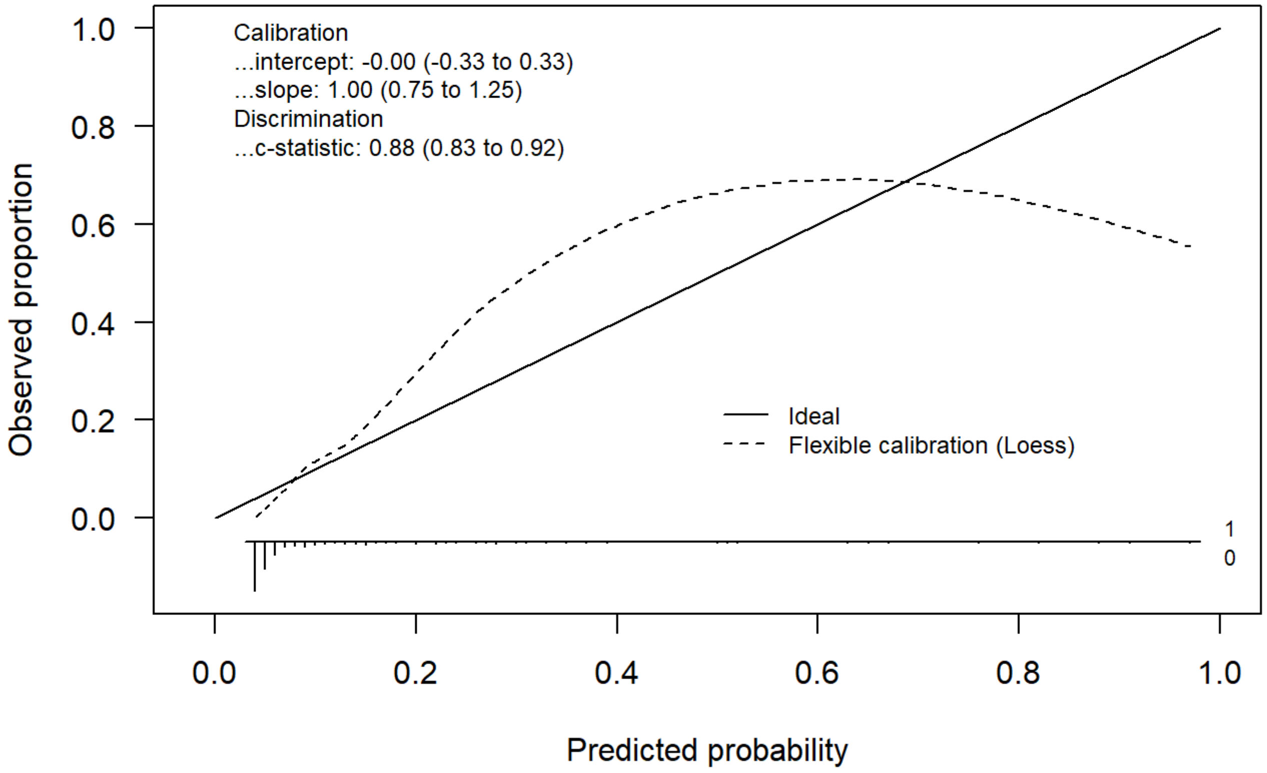


```
## Call:
## CalibrationCurves::val.prob.ci.2(p = pltcvd.imp.pred[, 1], y = test.org.na$CVD_23,
##     CL.smooth = FALSE, lty.smooth = 2, col.ideal = "black")
##
## A 95% confidence interval is given for the calibration intercept, calibration slope and c-statist
ic.
##
##           Dxy       C (ROC)            R2             D      D:Chi-sq
##  7.623321e-01  8.811660e-01  3.399381e-01  2.079450e-01  8.750514e+01
##           D:p             U      U:Chi-sq           U:p             Q
##  0.000000e+00 -4.807692e-03 -1.705303e-13  1.000000e+00  2.127527e-01
##         Brier     Intercept         Slope          Emax  Brier scaled
##  9.168694e-02 -6.238194e-11  1.000000e+00  2.768408e-11  2.466897e-01
##          Eavg           ECI
##  5.379058e-02  6.935004e-01
```

Isoregression model shows perfect fit?!
Going on to the validation

7.6 Validation

7.6.1 Original data analysis: Validation

```
# Remove NA
valid.org.na <- na.omit(valid.org)

# Predicted probability and classification
lrcvd.org.valid.pred <- predict(lrcvd.org.final
                                , newdata = valid.org.na
                                , type = "response")

lrcvd.org.valid.class <- ifelse(lrcvd.org.valid.pred>=0.5, 1, 0)

# Confusion matrix
cl_table.lrcvd.org.valid <- table (valid.org.na$CVD_23, lrcvd.org.valid.class)
rownames(cl_table.lrcvd.org.valid) <- c("CVD12", "CVD345")
colnames(cl_table.lrcvd.org.valid) <- c("CVD12", "CVD345")
addmargins(cl_table.lrcvd.org.valid)
```

```
##         lrcvd.org.valid.class
##          CVD12 CVD345 Sum
##   CVD12    398     18 416
##   CVD345    60     22  82
##   Sum      458     40 498
```

```
# Model accuracy (classification accuracy)
mean((valid.org.na$CVD_23 == lrcvd.org.valid.class))
```

```
## [1] 0.8433735
```

```
# Discriminability
roc.lrcvd.org.valid <- roc(valid.org.na$CVD_23
                           ,lrcvd.org.valid.pred
                           , add = TRUE)
```

```
## Setting levels: control = 0, case = 1
```

```
## Setting direction: controls < cases
```

```
roc.lrcvd.org.valid$auc
```

```
## Area under the curve: 0.831
```

```
ci(roc.lrcvd.org.valid)
```

```
## 95% CI: 0.7895-0.8726 (DeLong)
```

```
pROC::coords(roc.lrcvd.org.valid,
             "best",
             transpose = TRUE,
             best.method = "youden")
```

```
##   threshold specificity sensitivity
##   0.1678527   0.7451923   0.8170732
```

```
plot.roc(roc.lrcvd.org.valid
         , print.auc = TRUE
         , print.thres = FALSE
         , auc.polygon = TRUE
)
```

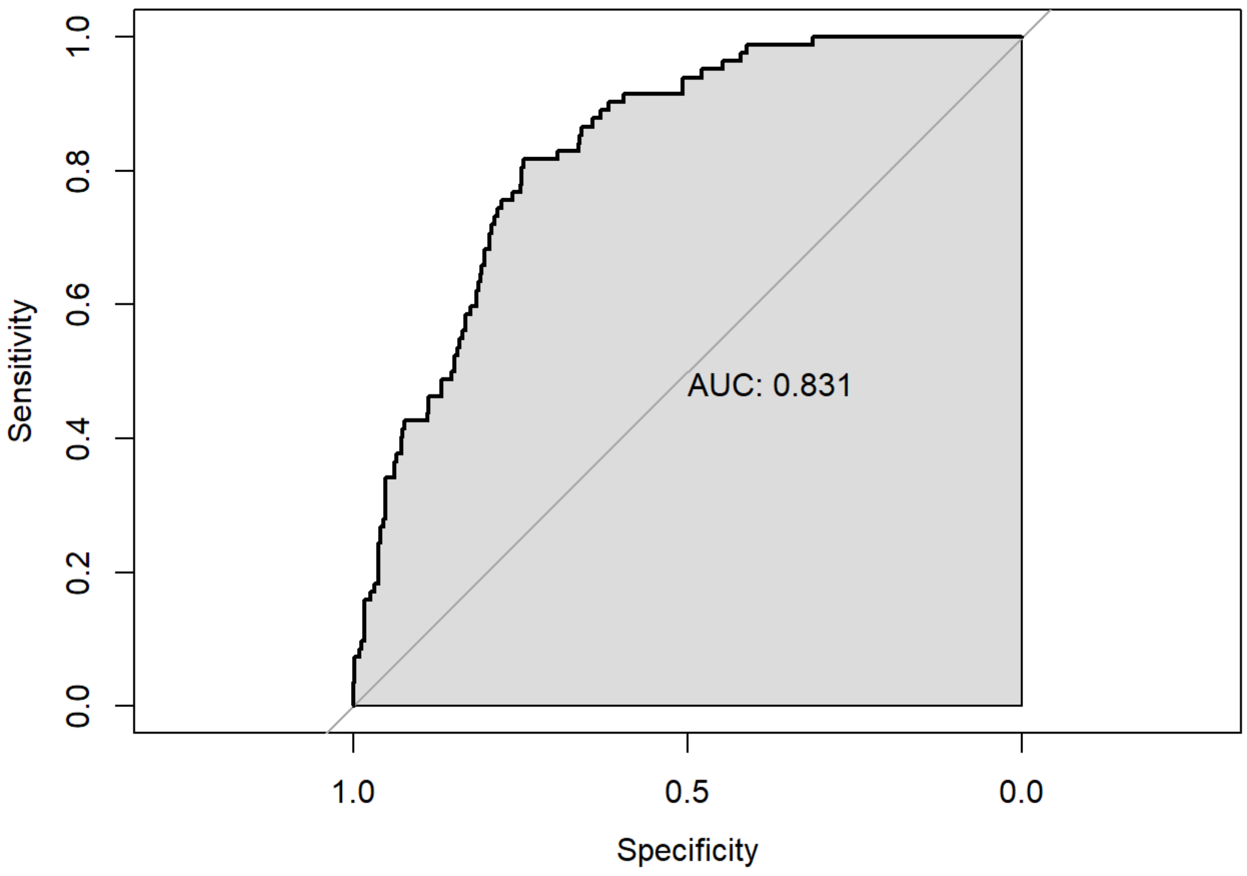


```
#Threshold discrimination ability

lrcvd.org.valid.pred.dcs <- ifelse(lrcvd.org.valid.pred > 0.1439794, 1, 0)

roc.lrcvd.org.valid.dcs <- roc(valid.org.na$CVD_23
                              ,lrcvd.org.valid.pred.dcs
                              , add = TRUE)
```

```
## Setting levels: control = 0, case = 1
## Setting direction: controls < cases
```

```
roc.lrcvd.org.valid.dcs$auc
```

```
## Area under the curve: 0.7607
```

```
ci(roc.lrcvd.org.valid.dcs)
```

```
## 95% CI: 0.7132-0.8082 (DeLong)
```

```
pROC::coords(roc.lrcvd.org.valid.dcs,
             "best",
             transpose = TRUE,
             best.method = "youden")
```

```
##   threshold specificity sensitivity
##   0.5000000   0.7043269   0.8170732
```

```
plot.roc(roc.lrcvd.org.valid.dcs
         , print.auc = TRUE
         , print.thres = FALSE
         , auc.polygon = TRUE
)
```

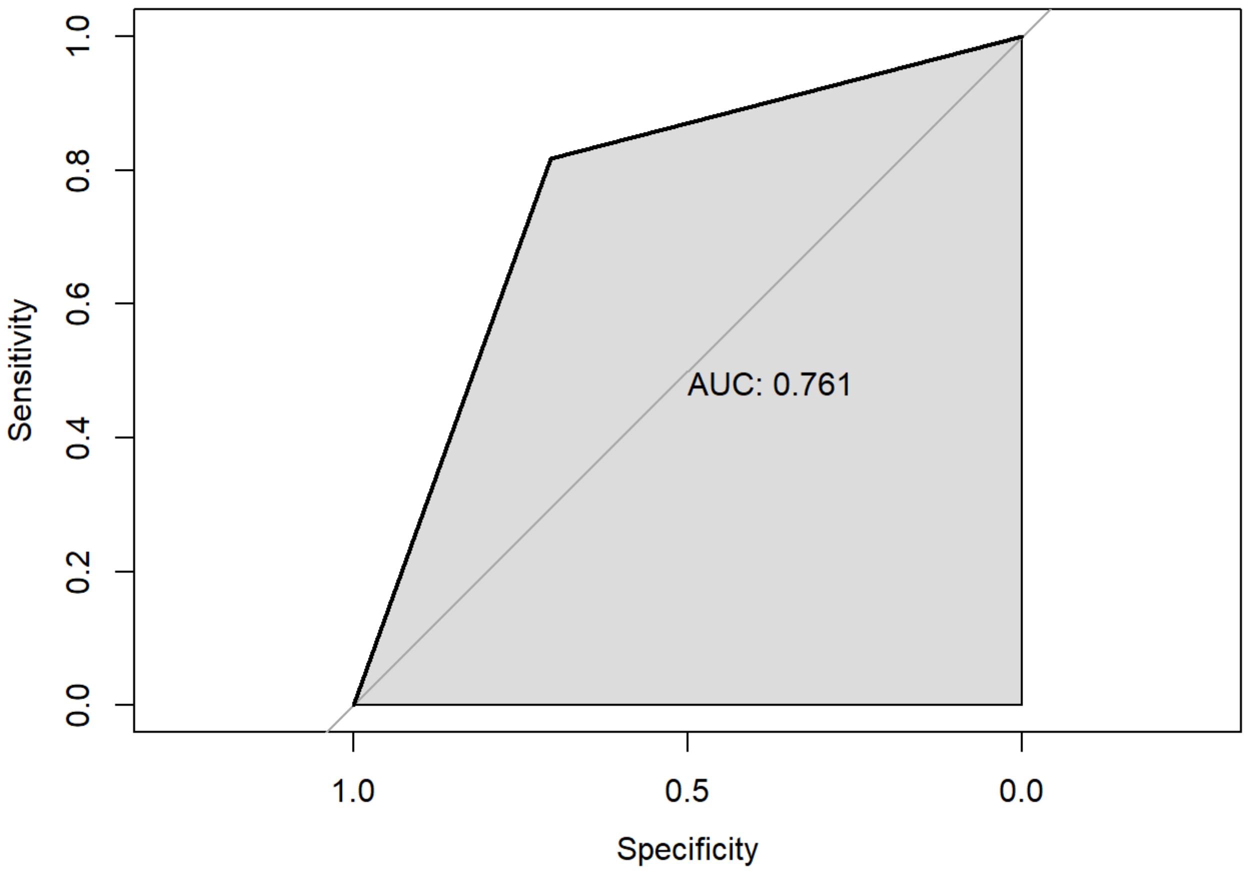

7.6.2 Imputted data analaysis: Validation

```
# Predicted probability and classification: pooled model
lrcvd.imp.valid.pred <- as.data.frame(predict(lrcvd.imp, valid.org.na, type = "response"))
lrcvd.imp.valid.class <- ifelse(lrcvd.imp.valid.pred>=0.5, 1, 0)

# Confusion matrix
cl_table.lrcvd.imp.valid <- table (valid.org.na$CVD_23, lrcvd.imp.valid.class)
rownames(cl_table.lrcvd.imp.valid) <- c("CVD12", "CVD345")
colnames(cl_table.lrcvd.imp.valid) <- c("CVD12", "CVD345")
addmargins(cl_table.lrcvd.imp.valid)
```

```
##         lrcvd.imp.valid.class
##          CVD12 CVD345 Sum
##   CVD12    398     18 416
##   CVD345    62     20  82
##   Sum      460     38 498
```

```
# Sensitivity
round(cl_table.lrcvd.imp.valid[2,2]/(cl_table.lrcvd.imp.valid[2,1] + cl_table.lrcvd.imp.valid[2,2]),
3)
```

```
## [1] 0.244
```

```
# Specificity
round(cl_table.lrcvd.imp.valid[1,1]/(cl_table.lrcvd.imp.valid[1,1] + cl_table.lrcvd.imp.valid[1,2]),
3)
```

```
## [1] 0.957
```

```
# Positive predictive rate
round(cl_table.lrcvd.imp.valid[2,2]/(cl_table.lrcvd.imp.valid[1,2] + cl_table.lrcvd.imp.valid[2,2]),
3)
```

```
## [1] 0.526
```

```
# Negative predictive rate
round(cl_table.lrcvd.imp.valid[1,1]/(cl_table.lrcvd.imp.valid[1,1] + cl_table.lrcvd.imp.valid[2,1]),
3)
```

```
## [1] 0.865
```

```
#model accuracy (classification accuracy)
mean((valid.org.na$CVD_23 == lrcvd.imp.valid.class))
```

```
## [1] 0.8393574
```

```
#Discriminability

roc.lrcvd.imp.valid <- roc(valid.org.na$CVD_23
                          , lrcvd.imp.valid.pred[,1]
                          , add = TRUE)
```

```
## Setting levels: control = 0, case = 1
```

```
## Setting direction: controls < cases
```

```
roc.lrcvd.imp.valid$auc
```

```
## Area under the curve: 0.8332
```

```
ci(roc.lrcvd.imp.valid)
```

```
## 95% CI: 0.7925-0.8739 (DeLong)
```

```
pROC::coords(roc.lrcvd.imp.valid
             , "best"
             , transpose = TRUE
             , best.method="youden"
)
```

```
##   threshold specificity sensitivity
##   0.1682498   0.7596154   0.7926829
```

```
plot.roc(roc.lrcvd.imp.valid
         , print.auc = TRUE
         , print.thres = FALSE
         , auc.polygon = TRUE
)
```

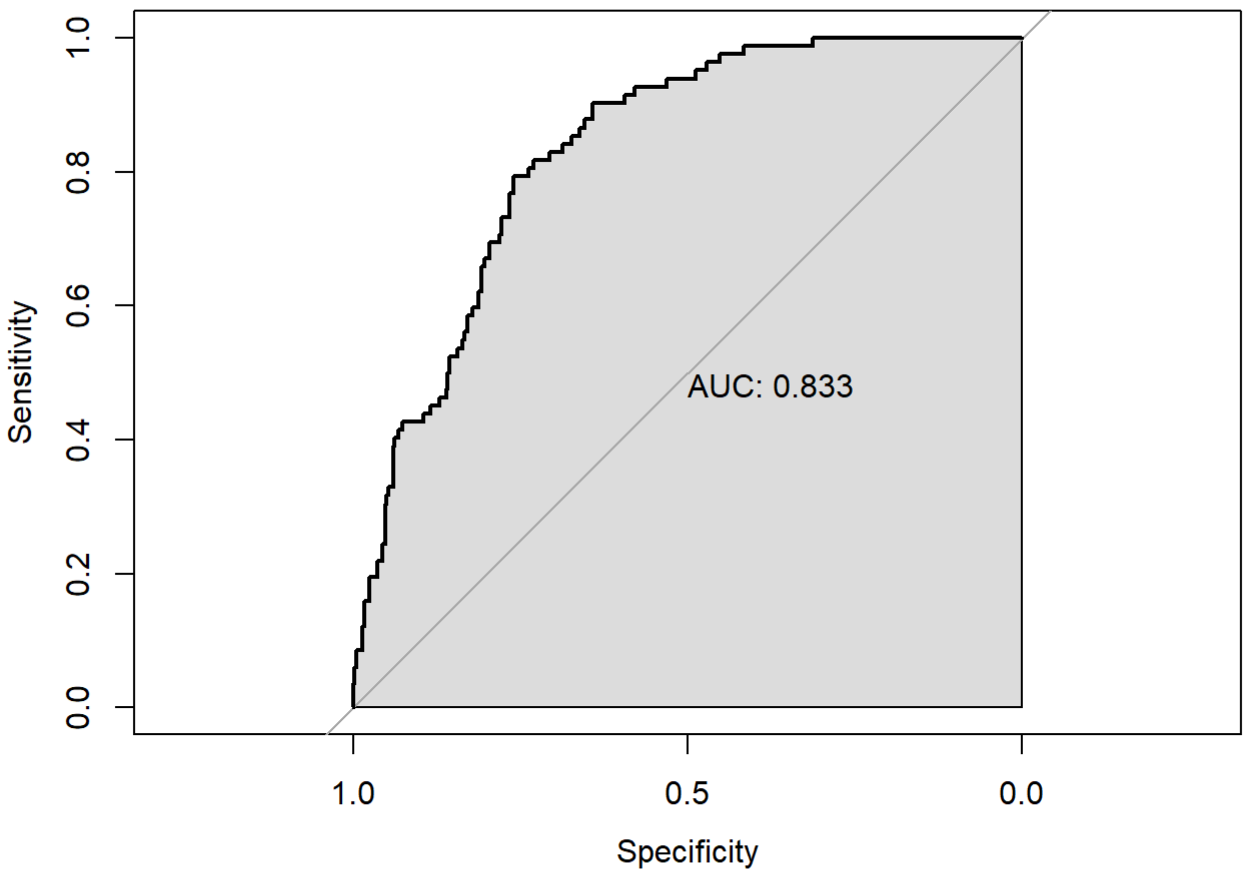


```
# Threshold discrimination ability

lrcvd.imp.valid.pred.dcs <- ifelse(lrcvd.imp.valid.pred[,1] > 0.1439794, 1, 0)

roc.lrcvd.imp.valid.dcs <- roc(valid.org.na$CVD_23
                               ,lrcvd.imp.valid.pred.dcs
                               , add = TRUE)
```

```
## Setting levels: control = 0, case = 1
## Setting direction: controls < cases
```

```
roc.lrcvd.imp.valid.dcs$auc
```

```
## Area under the curve: 0.7631
```

```
ci(roc.lrcvd.imp.valid.dcs)
```

```
## 95% CI: 0.7157-0.8105 (DeLong)
```

```
pROC::coords(roc.lrcvd.imp.valid.dcs,
             "best",
             transpose = TRUE,
             best.method = "youden")
```

```
##   threshold specificity sensitivity
##   0.5000000   0.7091346   0.8170732
```

```
plot.roc(roc.lrcvd.imp.valid.dcs
         , print.auc = TRUE
         , print.thres = FALSE
         , auc.polygon = TRUE
)
```

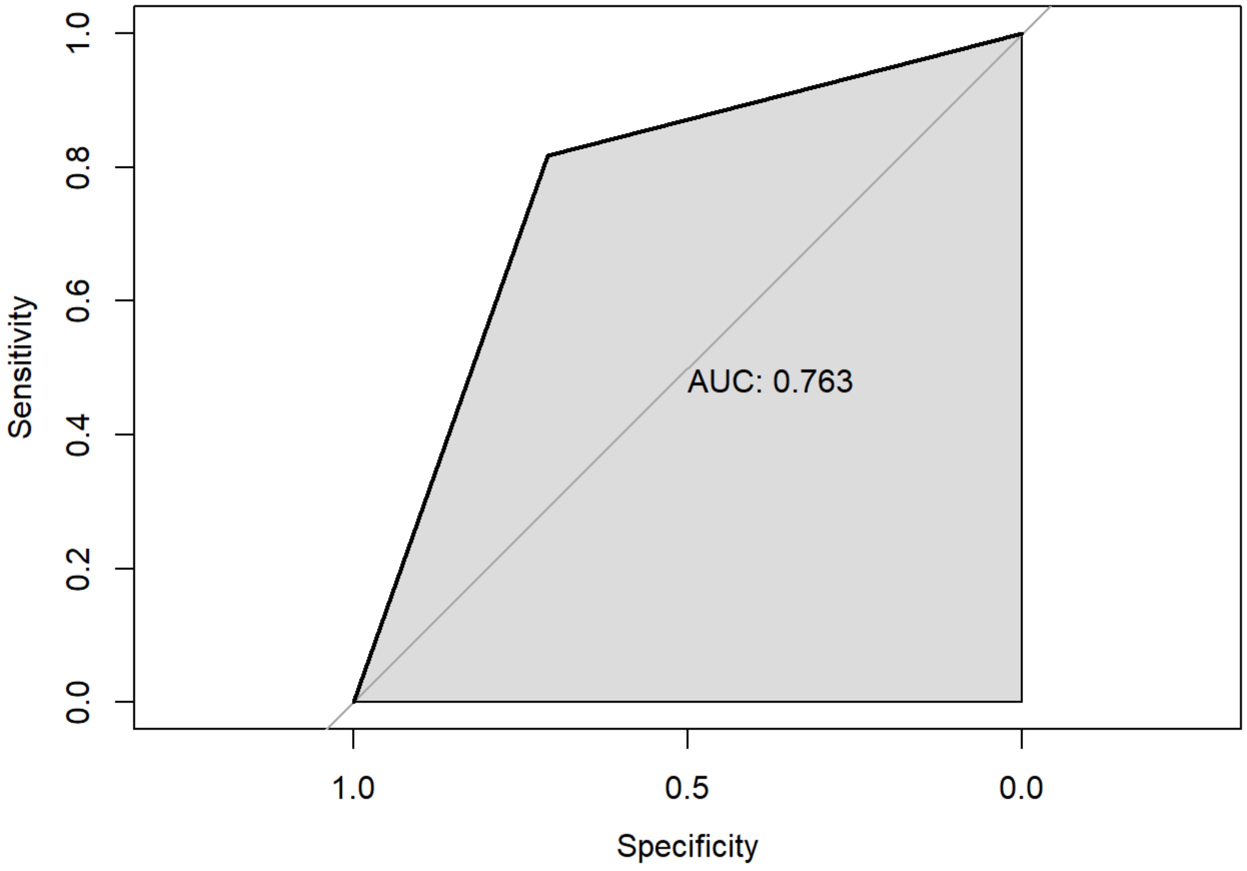

```
# Calibrations: on the validing dataset
cal.lrcvd.imp.valid <- val.prob(lrcvd.imp.valid.pred[,1]
                                , valid.org.na$CVD_23
                                , pl = FALSE)

cal.lrcvd.imp.valid
```

```
##          Dxy      C (ROC)           R2            D     D:Chi-sq          D:p
##  0.666422373  0.833211187  0.301520906  0.194337247 97.779949236           NA
##            U     U:Chi-sq          U:p            Q        Brier    Intercept
## -0.002632836  0.688847723  0.708628504  0.196970083  0.111497962 -0.119957738
##        Slope         Emax          E90         Eavg          S:z          S:p
##  0.909131477  0.255577200  0.072467384  0.033698098  1.143352905  0.252892098
```

```
hosmer_lemeshow(valid.org.na$CVD_23, lrcvd.imp.valid.pred[,1], 10, 'C')
```

```
## PVALUE 0.2526622
## stat 10.17936
```

```
## [1] 0.2526622
```

```
hosmer_lemeshow(valid.org.na$CVD_23, lrcvd.imp.valid.pred[,1], 10, 'H')
```

```
## PVALUE 0.01073413
## stat 19.89667
```

```
## [1] 0.01073413
```

```
CalibrationCurves::val.prob.ci.2(lrcvd.imp.valid.pred[,1], valid.org.na$CVD_23
                                 , lty.smooth = 2
                                 , CL.smooth = FALSE
                                 , col.ideal = "black")
```

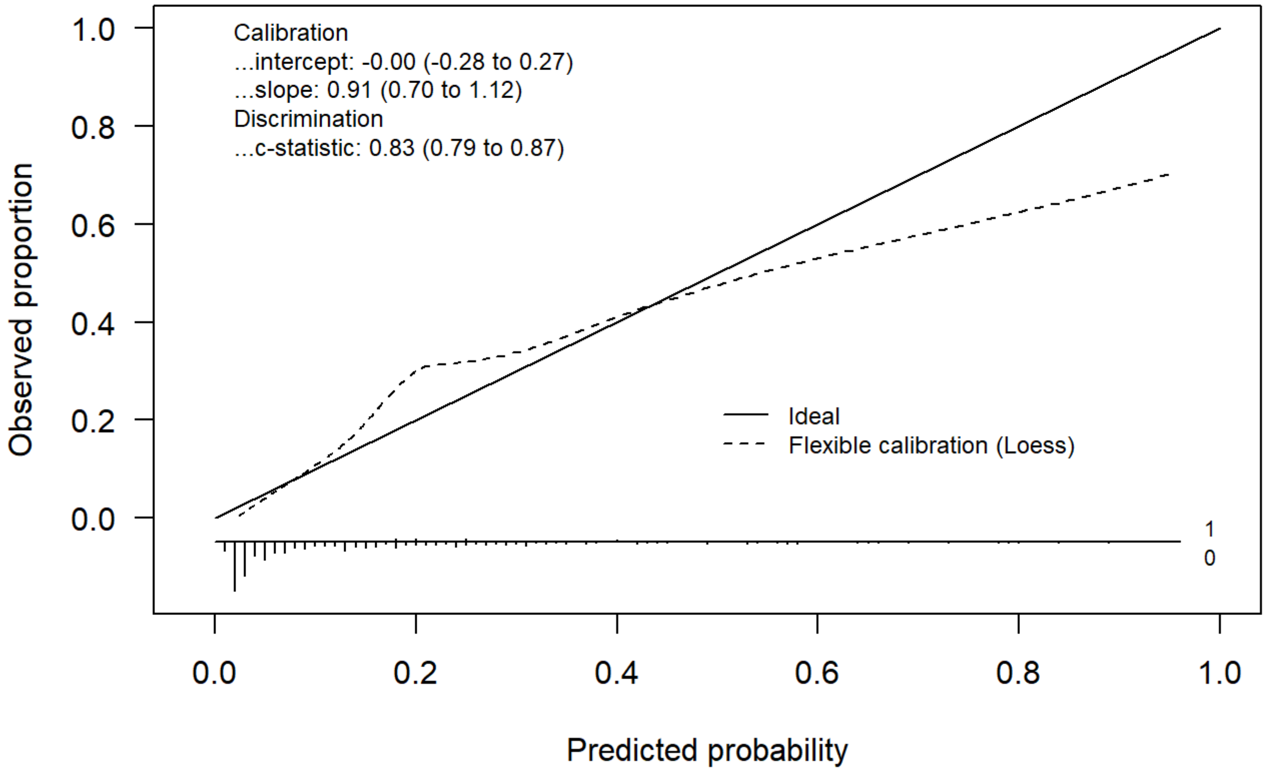


```
## Call:
## CalibrationCurves::val.prob.ci.2(p = lrcvd.imp.valid.pred[, 1],
##     y = valid.org.na$CVD_23, CL.smooth = FALSE, lty.smooth = 2,
##     col.ideal = "black")
##
## A 95% confidence interval is given for the calibration intercept, calibration slope and c-statist
ic.
##
##          Dxy      C (ROC)           R2            D     D:Chi-sq          D:p
##  0.666539634  0.833269817  0.303442039  0.195720476 98.468796959  0.000000000
##            U     U:Chi-sq          U:p            Q        Brier    Intercept
## -0.002632836  0.688847723  0.708628504  0.198353312  0.111497962 -0.003075474
##        Slope         Emax Brier scaled         Eavg          ECI
##  0.909131477  0.043442259  0.189377917  0.035898167  0.291055034
```

7.6.3 Isoregression model validation

```
# Predicted probability and classification: isoregression model
isocvd.imp.valid.pred.prob <- predict(lrcvd.imp,
                                      valid.org.na[,-length(valid.org.na)],
                                      type = "response")
isocvd.imp.valid.pred <- isocvd.imp(as.data.frame(isocvd.imp.valid.pred.prob)[,1])
isocvd.imp.valid.class <- ifelse(isocvd.imp.valid.pred>=0.5, 1, 0)

# Confusion matrix
cl_table.isocvd.imp.valid <- table (valid.org.na$CVD_23, isocvd.imp.valid.class)
rownames(cl_table.isocvd.imp.valid) <- c("CVD12", "CVD345")
colnames(cl_table.isocvd.imp.valid) <- c("CVD12", "CVD345")
addmargins(cl_table.isocvd.imp.valid)
```

```
##         isocvd.imp.valid.class
##          CVD12 CVD345 Sum
##   CVD12    388     28 416
##   CVD345    49     33  82
##   Sum      437     61 498
```

```
# Sensitivity
round(cl_table.isocvd.imp.valid[2,2]/(cl_table.isocvd.imp.valid[2,1] + cl_table.isocvd.imp.valid[2,
2]),3)
```

```
## [1] 0.402
```

```
# Specificity
round(cl_table.isocvd.imp.valid[1,1]/(cl_table.isocvd.imp.valid[1,1] + cl_table.isocvd.imp.valid[1,
2]),3)
```

```
## [1] 0.933
```

```
# Positive predictive rate
round(cl_table.isocvd.imp.valid[2,2]/(cl_table.isocvd.imp.valid[1,2] + cl_table.isocvd.imp.valid[2,
2]),3)
```

```
## [1] 0.541
```

```
# Negative predictive rate
round(cl_table.isocvd.imp.valid[1,1]/(cl_table.isocvd.imp.valid[1,1] + cl_table.isocvd.imp.valid[2,
1]),3)
```

```
## [1] 0.888
```

```
#model accuracy (classification accuracy)
mean((valid.org.na$CVD_23 == isocvd.imp.valid.class))
```

```
## [1] 0.8453815
```

```
#Discriminability

roc.isocvd.imp.valid <- roc(valid.org.na$CVD_23
                            , isocvd.imp.valid.pred
                            , add = TRUE)
```

```
## Setting levels: control = 0, case = 1
```

```
## Setting direction: controls < cases
```

```
roc.isocvd.imp.valid$auc
```

```
## Area under the curve: 0.8338
```

```
ci(roc.isocvd.imp.valid)
```

```
## 95% CI: 0.7933-0.8743 (DeLong)
```

```
pROC::coords(roc.isocvd.imp.valid
             , "best"
             , transpose = TRUE
             , best.method="youden"
)
```

```
##   threshold specificity sensitivity
##   0.1361607   0.7427885   0.7926829
```

```
plot.roc(roc.isocvd.imp.valid
         , print.auc = TRUE
         , print.thres = FALSE
         , auc.polygon = TRUE
)
```

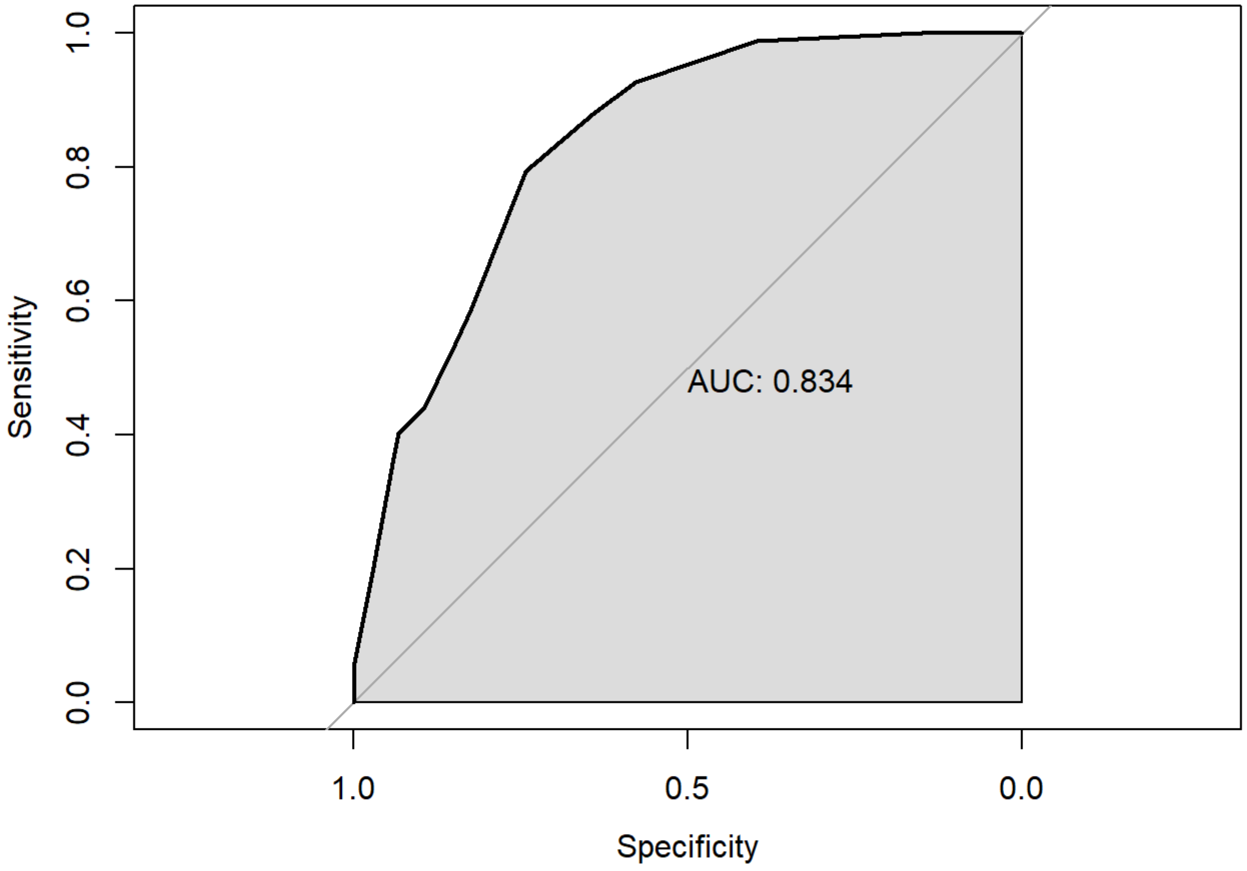


```
# Threshold discrimination ability

isocvd.imp.valid.pred.dcs <- ifelse(isocvd.imp.valid.pred > 0.1439794, 1, 0)

roc.isocvd.imp.valid.dcs <- roc(valid.org.na$CVD_23
                                ,isocvd.imp.valid.pred.dcs
                                , add = TRUE)
```

```
## Setting levels: control = 0, case = 1
## Setting direction: controls < cases
```

```
roc.lrcvd.imp.valid.dcs$auc
```

```
## Area under the curve: 0.7631
```

```
ci(roc.lrcvd.imp.valid.dcs)
```

```
## 95% CI: 0.7157-0.8105 (DeLong)
```

```
pROC::coords(roc.lrcvd.imp.valid.dcs,
             "best",
             transpose = TRUE,
             best.method = "youden")
```

```
##   threshold specificity sensitivity
##   0.5000000   0.7091346   0.8170732
```

```
plot.roc(roc.lrcvd.imp.valid.dcs
         , print.auc = TRUE
         , print.thres = FALSE
         , auc.polygon = TRUE
)
```

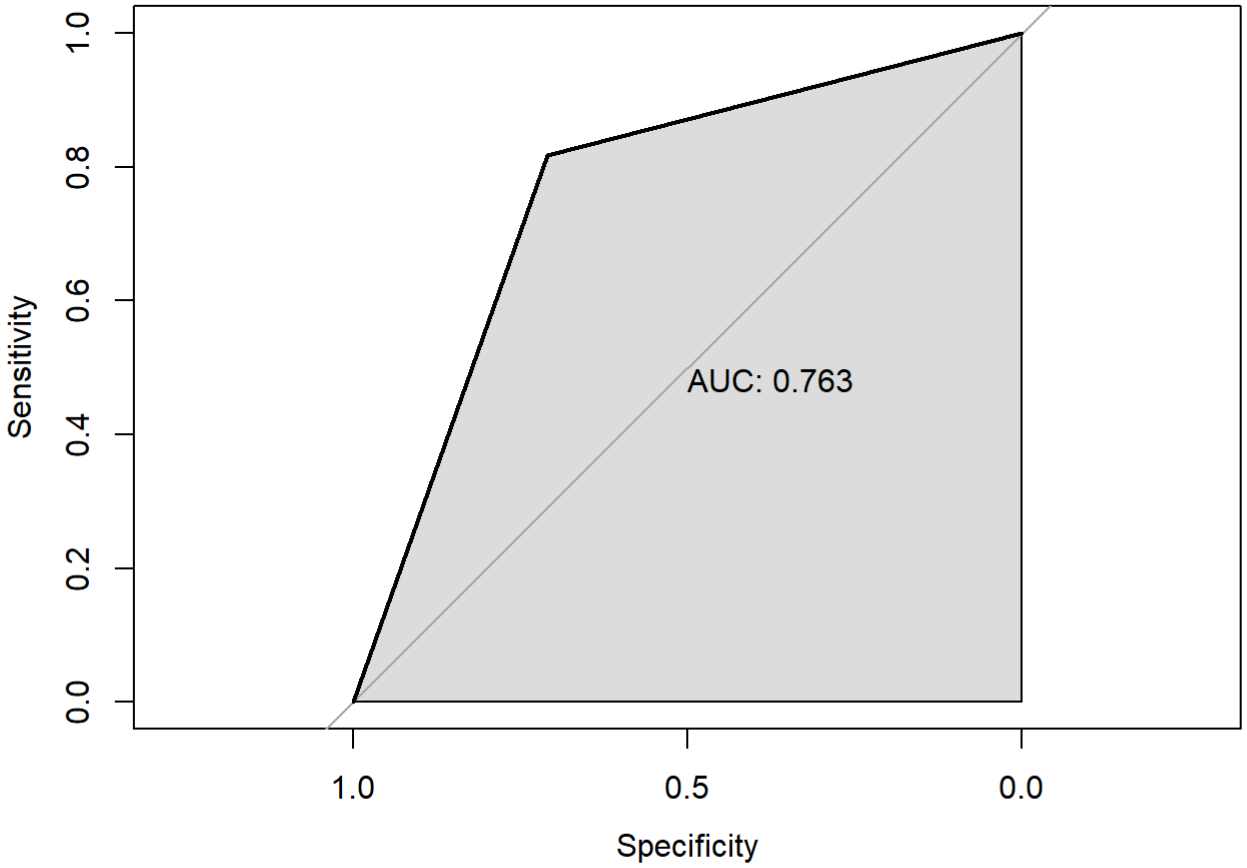

```
# Calibrations: on the validing dataset
cal.isocvd.imp.valid <- val.prob(isocvd.imp.valid.pred
                                 , valid.org.na$CVD_23
                                 , pl = FALSE)

cal.isocvd.imp.valid
```

```
##         Dxy     C (ROC)          R2           D    D:Chi-sq         D:p
##  0.60941160  0.80470580  0.24858223  0.16500163 72.94071151          NA
##           U    U:Chi-sq         U:p           Q       Brier   Intercept
##  0.01450017  8.32207431  0.01559138  0.15050146  0.11331766 -0.22807538
##       Slope        Emax         E90        Eavg         S:z         S:p
##  0.72137558  0.14487202  0.14168764  0.05189295  3.12962093  0.00175032
```

```
hosmer_lemeshow(valid.org.na$CVD_23, isocvd.imp.valid.pred, 10, 'C')
```

```
## PVALUE 0.001814206
## stat 24.60333
```

```
## [1] 0.001814206
```

```
hosmer_lemeshow(valid.org.na$CVD_23, isocvd.imp.valid.pred, 10, 'H')
```

```
## PVALUE 0.002558882
## stat 23.714
```

```
## [1] 0.002558882
```

```
CalibrationCurves::val.prob.ci.2(isocvd.imp.valid.pred, valid.org.na$CVD_23
                                 , lty.smooth = 2
                                 , CL.smooth = FALSE
                                 , col.ideal = "black")
```

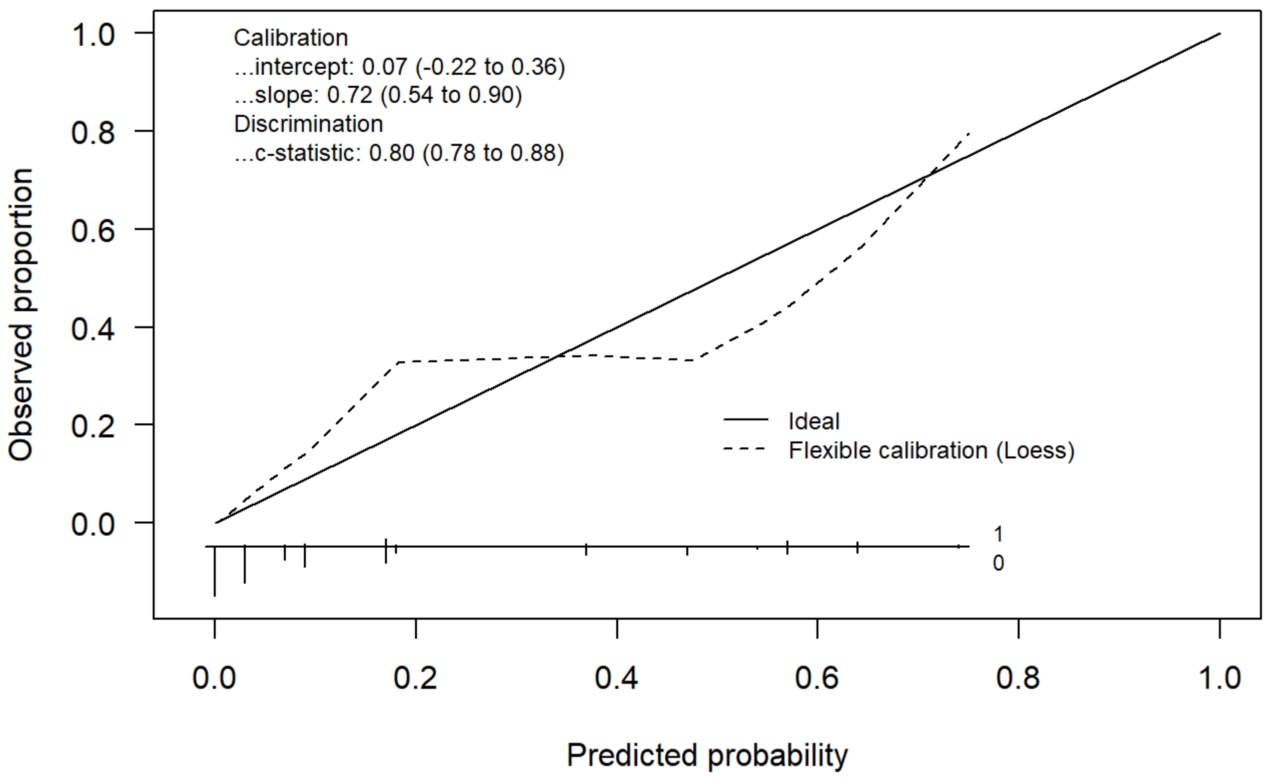


```
## Call:
## CalibrationCurves::val.prob.ci.2(p = isocvd.imp.valid.pred, y = valid.org.na$CVD_23,
##     CL.smooth = FALSE, lty.smooth = 2, col.ideal = "black")
##
## A 95% confidence interval is given for the calibration intercept, calibration slope and c-statist
ic.
##
##          Dxy      C (ROC)           R2            D     D:Chi-sq          D:p
##   0.60941160   0.80470580   0.27439053   0.18408896  81.26278581   0.00000000
##            U     U:Chi-sq          U:p            Q        Brier    Intercept
##   0.01450017   8.32207431   0.01559138   0.16958879   0.12943164   0.07113985
##        Slope         Emax Brier scaled         Eavg          ECI
##   0.72137558   0.11620798   0.05899494   0.05224899   0.55734784
```

7.6.4 Platt scaling model validation

```
# Predicted probability and classification: Platt scaling model
colnames(lrcvd.imp.valid.pred) <- c("yhat")
pltcvd.imp.valid.pred <- as.data.frame(predict(pltcvd.imp, lrcvd.imp.valid.pred, type = "response"
))
pltcvd.imp.valid.class <- ifelse(pltcvd.imp.valid.pred>=0.5, 1, 0)



# Confusion matrix
cl_table.pltcvd.imp.valid <- table (valid.org.na$CVD_23, pltcvd.imp.valid.class)
rownames(cl_table.pltcvd.imp.valid) <- c("CVD12", "CVD345")
colnames(cl_table.pltcvd.imp.valid) <- c("CVD12", "CVD345")
addmargins(cl_table.pltcvd.imp.valid)
```

```
##         pltcvd.imp.valid.class
##          CVD12 CVD345 Sum
##   CVD12    396     20 416
##   CVD345    57     25  82
##   Sum      453     45 498
```

```
# Sensitivity
round(cl_table.pltcvd.imp.valid[2,2]/(cl_table.pltcvd.imp.valid[2,1] + cl_table.pltcvd.imp.valid[2,
2]),3)
```

```
## [1] 0.305
```

```
# Specificity
round(cl_table.pltcvd.imp.valid[1,1]/(cl_table.pltcvd.imp.valid[1,1] + cl_table.pltcvd.imp.valid[1,
2]),3)
```

```
## [1] 0.952
```

```
# Positive predictive rate
round(cl_table.pltcvd.imp.valid[2,2]/(cl_table.pltcvd.imp.valid[1,2] + cl_table.pltcvd.imp.valid[2,
2]),3)
```

```
## [1] 0.556
```

```
# Negative predictive rate
round(cl_table.pltcvd.imp.valid[1,1]/(cl_table.pltcvd.imp.valid[1,1] + cl_table.pltcvd.imp.valid[2,
1]),3)
```

```
## [1] 0.874
```

```
#model accuracy (classification accuracy)
mean((valid.org.na$CVD_23 == pltcvd.imp.valid.class))
```

```
## [1] 0.8453815
```

```
#Discriminability

roc.pltcvd.imp.valid <- roc(valid.org.na$CVD_23
                            , pltcvd.imp.valid.pred[,1]
                            , add = TRUE)
```

```
## Setting levels: control = 0, case = 1
```

```
## Setting direction: controls < cases
```

```
roc.pltcvd.imp.valid$auc
```

```
## Area under the curve: 0.8332
```

```
ci(roc.pltcvd.imp.valid)
```

```
## 95% CI: 0.7925-0.8739 (DeLong)
```

```
pROC::coords(roc.pltcvd.imp.valid
             , "best"
             , transpose = TRUE
             , best.method="youden"
)
```

```
##   threshold specificity sensitivity
##   0.1143724   0.7596154   0.7926829
```

```
plot.roc(roc.pltcvd.imp.valid
         , print.auc = TRUE
         , print.thres = FALSE
         , auc.polygon = TRUE
)
```

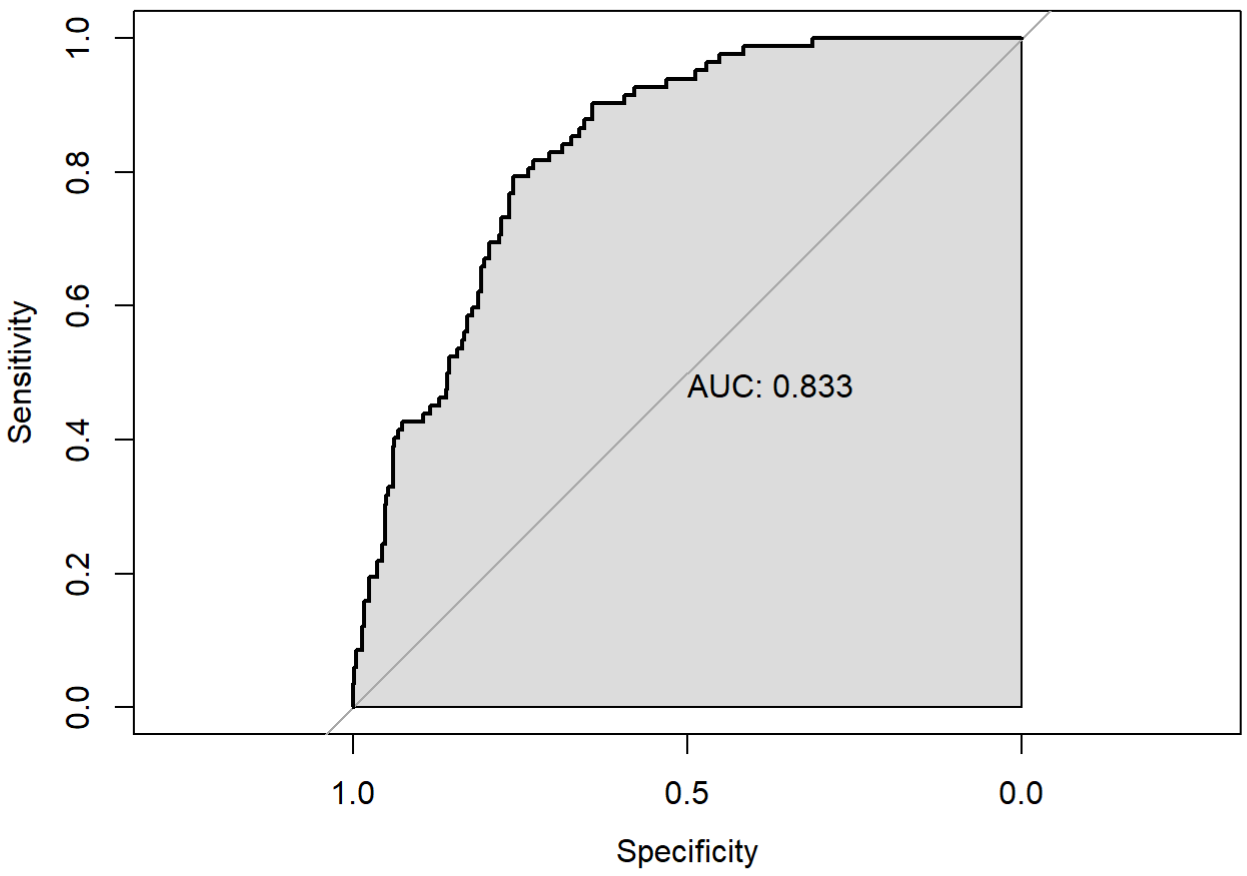


```
# Threshold discrimination ability

pltcvd.imp.valid.pred.dcs <- ifelse(pltcvd.imp.valid.pred > 0.1439794, 1, 0)

roc.pltcvd.imp.valid.dcs <- roc(valid.org.na$CVD_23
                                ,pltcvd.imp.valid.pred.dcs[,1]
                                , add = TRUE)
```

```
## Setting levels: control = 0, case = 1
## Setting direction: controls < cases
```

```
roc.lrcvd.imp.valid.dcs$auc
```

```
## Area under the curve: 0.7631
```

```
ci(roc.lrcvd.imp.valid.dcs)
```

```
## 95% CI: 0.7157-0.8105 (DeLong)
```

```
pROC::coords(roc.lrcvd.imp.valid.dcs,
             "best",
             transpose = TRUE,
             best.method = "youden")
```

```
##   threshold specificity sensitivity
##   0.5000000   0.7091346   0.8170732
```

```
plot.roc(roc.lrcvd.imp.valid.dcs
         , print.auc = TRUE
         , print.thres = FALSE
         , auc.polygon = TRUE
)
```

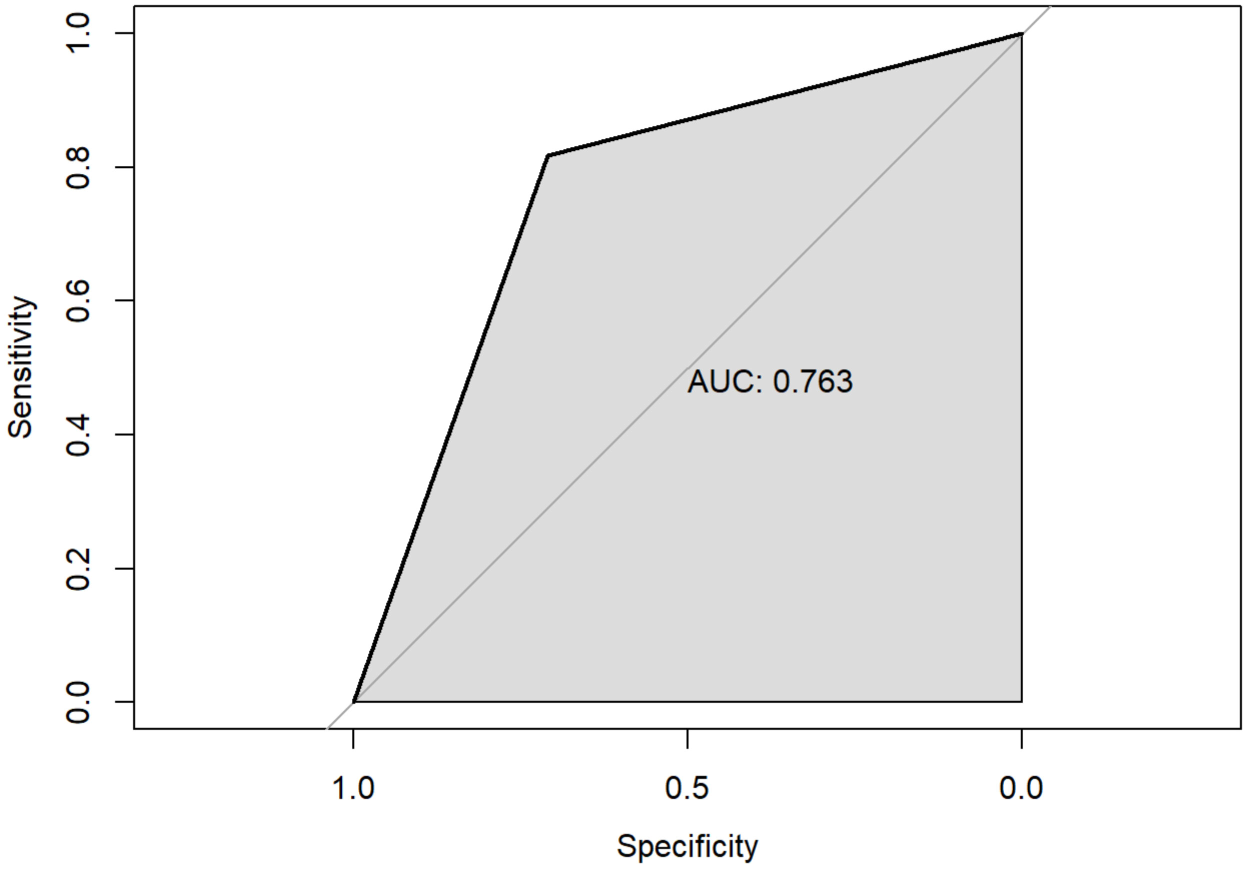

```
# Calibrations: on the validing dataset
cal.pltcvd.imp.valid <- val.prob(pltcvd.imp.valid.pred[,1]
                                 , valid.org.na$CVD_23
                                 , pl = FALSE)

cal.pltcvd.imp.valid
```

```
##          Dxy      C (ROC)           R2            D     D:Chi-sq          D:p
##  0.666686210  0.833343105  0.219856175  0.137240240 69.345639394           NA
##            U     U:Chi-sq          U:p            Q        Brier    Intercept
##  0.012884557  8.416509437  0.014872302  0.124355683  0.118129241 -0.375059911
##        Slope         Emax          E90         Eavg          S:z          S:p
##  0.723880147  0.350631802  0.153899842  0.065200527  2.911274656  0.003599574
```

```
hosmer_lemeshow(valid.org.na$CVD_23, pltcvd.imp.valid.pred[,1], 10, 'C')
```

```
## PVALUE 0.0003296174
## stat 28.90414
```

```
## [1] 0.0003296174
```

```
hosmer_lemeshow(valid.org.na$CVD_23, pltcvd.imp.valid.pred[,1], 10, 'H')
```

```
## PVALUE 2.647237e-10
## stat 61.25105
```

```
## [1] 2.647237e-10
```

```
CalibrationCurves::val.prob.ci.2(pltcvd.imp.valid.pred[,1], valid.org.na$CVD_23
                                 , lty.smooth = 2
                                 , CL.smooth = FALSE
                                 , col.ideal = "black")
```

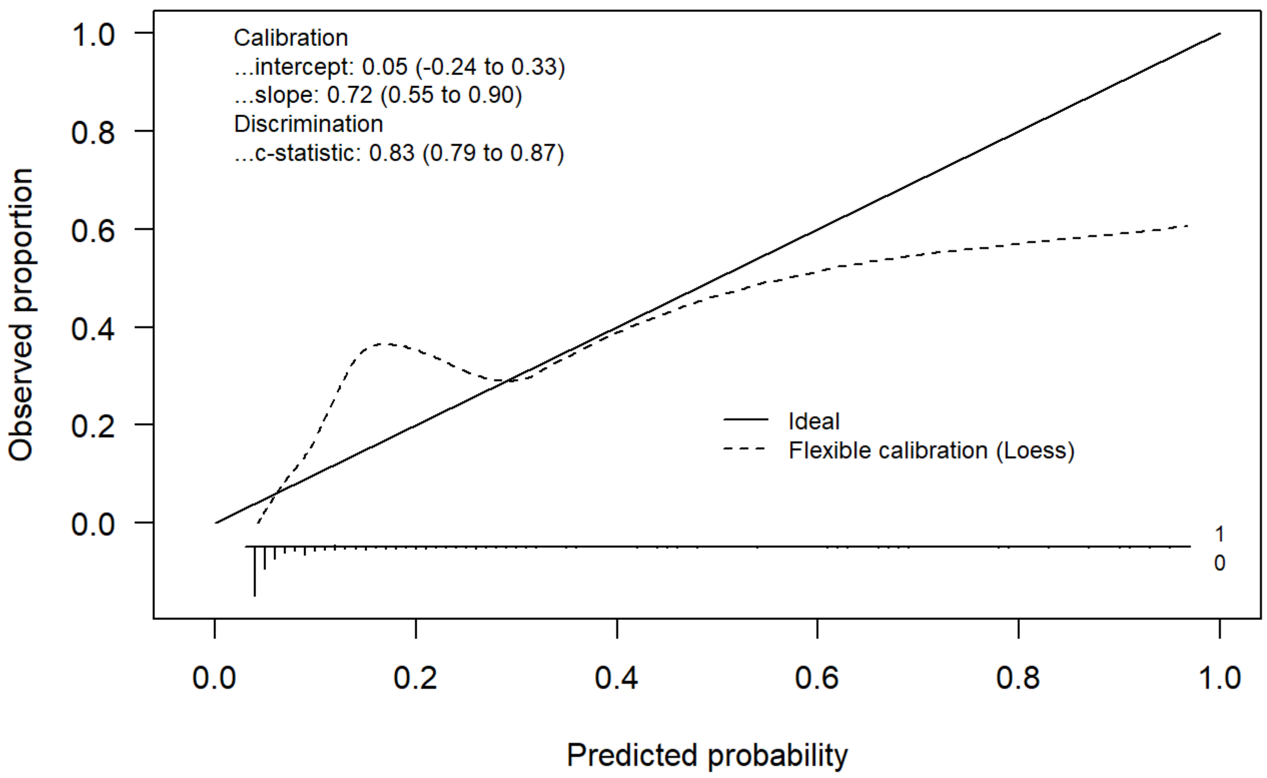


```
## Call:
## CalibrationCurves::val.prob.ci.2(p = pltcvd.imp.valid.pred[,
##     1], y = valid.org.na$CVD_23, CL.smooth = FALSE, lty.smooth = 2,
##     col.ideal = "black")
##
## A 95% confidence interval is given for the calibration intercept, calibration slope and c-statist
ic.
##
##          Dxy       C (ROC)            R2             D      D:Chi-sq           D:p
##   0.66659826    0.83329913    0.24451649    0.15414086   77.76214883    0.00000000
##            U      U:Chi-sq           U:p             Q         Brier     Intercept
##   0.01288456    8.41650944    0.01487230    0.14125630    0.11812924    0.04695932
##        Slope          Emax  Brier scaled          Eavg           ECI
##   0.72388015    0.14800820    0.14116662    0.07057692    1.09353833
```

8 Risk prediction model development: In-hospital death

8.1 Original data analysis

Original data analysis includes complete cases only.

```
# Remove cases with NA values
train.org.na <- na.omit(train.org)

# Logistic regression model: Enter all input parameters
lrdeathih.org.enter <- glm(DEATH_INHOSPITAL ~ GENDER
                       + CIA_AMI
                       + CIA_CHF
                       + CIA_PVD
                       + CIA_CEVD
                       + CIA_DEM
                       + CIA_COPD
                       + CIA_PUD
                       + CIA_LD_MLD
                       + CIA_LD_SEV
                       + CIA_DM
                       + CIA_DM_CX
                       + CIA_HEMIPLAEGIA
                       + CIA_CKD
                       + CIA_CA
                       + CIA_CA_MET
                       + PREOP_HB
                       + PREOP_WCC
                       + PREOP_NA
                       + PREOP_K
                       + SURG_SEV
                       + SURG_SCHEDULED_TYPE
                       + PreopICU
                       , data =  train.org.na, family = binomial, na.action = "na.omit")


summary(lrdeathih.org.enter)
```

```
##
## Call:
## glm(formula = DEATH_INHOSPITAL ~ GENDER + CIA_AMI + CIA_CHF +
##     CIA_PVD + CIA_CEVD + CIA_DEM + CIA_COPD + CIA_PUD + CIA_LD_MLD +
##     CIA_LD_SEV + CIA_DM + CIA_DM_CX + CIA_HEMIPLAEGIA + CIA_CKD +
##     CIA_CA + CIA_CA_MET + PREOP_HB + PREOP_WCC + PREOP_NA + PREOP_K +
##     SURG_SEV + SURG_SCHEDULED_TYPE + PreopICU, family = binomial,
##     data = train.org.na, na.action = "na.omit")
##
## Deviance Residuals:
##     Min       1Q   Median       3Q      Max
## -2.0615  -0.2468  -0.1252  -0.0890   3.3133
##
## Coefficients:
##                       Estimate Std. Error z value Pr(>|z|)
## (Intercept)          -13.15899    5.88645  -2.235  0.02539 *
## GENDER1               -0.11995    0.30791  -0.390  0.69685
## CIA_AMI1             -15.05096 1267.22290  -0.012  0.99052
## CIA_CHF1               1.01825    0.44265   2.300  0.02143 *
## CIA_PVD1               0.98181    0.65528   1.498  0.13405
## CIA_CEVD1              0.63177    0.77482   0.815  0.41486
## CIA_DEM1              -1.45729    0.79786  -1.826  0.06778 .
## CIA_COPD1              0.20790    0.82794   0.251  0.80173
## CIA_PUD1              -1.31764    1.23922  -1.063  0.28765
## CIA_LD_MLD1          -13.51023 1483.78767  -0.009  0.99274
## CIA_LD_SEV1          -13.23001 2179.55526  -0.006  0.99516
## CIA_DM1                0.27783    0.57616   0.482  0.62966
## CIA_DM_CX1            -0.43939    0.45050  -0.975  0.32940
## CIA_HEMIPLAEGIA1       0.53110    1.03971   0.511  0.60948
## CIA_CKD1               1.61952    0.38022   4.259 2.05e-05 ***
## CIA_CA1                1.58827    0.45904   3.460  0.00054 ***
## CIA_CA_MET1            1.92197    0.61713   3.114  0.00184 **
## PREOP_HB              -0.20513    0.07651  -2.681  0.00733 **
## PREOP_WCC              0.05954    0.02258   2.637  0.00838 **
## PREOP_NA               0.07356    0.03903   1.885  0.05945 .
## PREOP_K               -0.07746    0.28027  -0.276  0.78226
## SURG_SEV1             -0.04385    0.36094  -0.121  0.90331
## SURG_SEV2              0.79264    0.43165   1.836  0.06632 .
## SURG_SCHEDULED_TYPE1   1.67458    0.41778   4.008 6.12e-05 ***
## PreopICU1              1.48088    0.72664   2.038  0.04155 *
## ---
## Signif. codes:  0 '***' 0.001 '**' 0.01 '*' 0.05 '.' 0.1 ' ' 1
##
## (Dispersion parameter for binomial family taken to be 1)
##
##     Null deviance: 515.93  on 1570  degrees of freedom
## Residual deviance: 377.50  on 1546  degrees of freedom
## AIC: 427.5
##
## Number of Fisher Scoring iterations: 16
```

```
# Stepwise selection method: AIC guided.
# Suppressed output
lrdeathih.org.sel <- step(lrdeathih.org.enter)
```

```
summary(lrdeathih.org.sel)
```

```
##
## Call:
## glm(formula = DEATH_INHOSPITAL ~ CIA_CHF + CIA_CEVD + CIA_DEM +
##     CIA_CKD + CIA_CA + CIA_CA_MET + PREOP_HB + PREOP_WCC + PREOP_NA +
##     SURG_SEV + SURG_SCHEDULED_TYPE + PreopICU, family = binomial,
##     data = train.org.na, na.action = "na.omit")
##
## Deviance Residuals:
##     Min       1Q   Median       3Q      Max
## -2.0050  -0.2568  -0.1269  -0.0912   3.2607
##
## Coefficients:
##                       Estimate Std. Error z value Pr(>|z|)
## (Intercept)          -13.08208    5.41682  -2.415 0.015732 *
## CIA_CHF1               0.99992    0.43477   2.300 0.021455 *
## CIA_CEVD1              0.93809    0.59645   1.573 0.115771
## CIA_DEM1              -1.38100    0.77743  -1.776 0.075673 .
## CIA_CKD1               1.39082    0.34676   4.011 6.05e-05 ***
## CIA_CA1                1.60339    0.45288   3.540 0.000399 ***
## CIA_CA_MET1            1.94517    0.60051   3.239 0.001199 **
## PREOP_HB              -0.19698    0.07408  -2.659 0.007834 **
## PREOP_WCC              0.05719    0.02176   2.628 0.008589 **
## PREOP_NA               0.06923    0.03795   1.824 0.068120 .
## SURG_SEV1              0.11922    0.34773   0.343 0.731703
## SURG_SEV2              0.88494    0.42154   2.099 0.035791 *
## SURG_SCHEDULED_TYPE1   1.66883    0.41387   4.032 5.52e-05 ***
## PreopICU1              1.28998    0.68651   1.879 0.060239 .
## ---
## Signif. codes:  0 '***' 0.001 '**' 0.01 '*' 0.05 '.' 0.1 ' ' 1
##
## (Dispersion parameter for binomial family taken to be 1)
##
##     Null deviance: 515.93  on 1570  degrees of freedom
## Residual deviance: 384.31  on 1557  degrees of freedom
## AIC: 412.31
##
## Number of Fisher Scoring iterations: 7
```

```
# Final logistic regression model with the selected input parameters
lrdeathih.org.final <- glm(DEATH_INHOSPITAL ~ CIA_CHF
                           + CIA_CEVD
                           + CIA_DEM
                           + CIA_CKD
                           + CIA_CA
                           + CIA_CA_MET
                           + PREOP_HB
                           + PREOP_WCC
                           + PREOP_NA
                           + SURG_SEV
                           + SURG_SCHEDULED_TYPE
                           + PreopICU
                       , data =  train.org.na
                       , family = binomial, na.action = "na.omit")

summary(lrdeathih.org.final)
```

```
##
## Call:
## glm(formula = DEATH_INHOSPITAL ~ CIA_CHF + CIA_CEVD + CIA_DEM +
##     CIA_CKD + CIA_CA + CIA_CA_MET + PREOP_HB + PREOP_WCC + PREOP_NA +
##     SURG_SEV + SURG_SCHEDULED_TYPE + PreopICU, family = binomial,
##     data = train.org.na, na.action = "na.omit")
##
## Deviance Residuals:
##     Min       1Q   Median       3Q      Max
## -2.0050  -0.2568  -0.1269  -0.0912   3.2607
##
## Coefficients:
##                      Estimate Std. Error z value Pr(>|z|)
## (Intercept)          -13.08208    5.41682  -2.415 0.015732 *
## CIA_CHF1               0.99992    0.43477   2.300 0.021455 *
## CIA_CEVD1              0.93809    0.59645   1.573 0.115771
## CIA_DEM1              -1.38100    0.77743  -1.776 0.075673 .
## CIA_CKD1               1.39082    0.34676   4.011 6.05e-05 ***
## CIA_CA1                1.60339    0.45288   3.540 0.000399 ***
## CIA_CA_MET1            1.94517    0.60051   3.239 0.001199 **
## PREOP_HB              -0.19698    0.07408  -2.659 0.007834 **
## PREOP_WCC              0.05719    0.02176   2.628 0.008589 **
## PREOP_NA               0.06923    0.03795   1.824 0.068120 .
## SURG_SEV1              0.11922    0.34773   0.343 0.731703
## SURG_SEV2              0.88494    0.42154   2.099 0.035791 *
## SURG_SCHEDULED_TYPE1   1.66883    0.41387   4.032 5.52e-05 ***
## PreopICU1              1.28998    0.68651   1.879 0.060239 .
## ---
## Signif. codes:  0 '***' 0.001 '**' 0.01 '*' 0.05 '.' 0.1 ' ' 1
##
## (Dispersion parameter for binomial family taken to be 1)
##
##     Null deviance: 515.93  on 1570  degrees of freedom
## Residual deviance: 384.31  on 1557  degrees of freedom
## AIC: 412.31
##
## Number of Fisher Scoring iterations: 7
```

```
# OR and 95%CI
exp(cbind(OR = coef(lrdeathih.org.final), confint(lrdeathih.org.final)))
```

```
##                                OR        2.5 %      97.5 %
## (Intercept)          2.082206e-06 3.860193e-11  0.06774073
## CIA_CHF1             2.718051e+00 1.110371e+00  6.17394049
## CIA_CEVD1            2.555093e+00 7.184605e-01  7.70149763
## CIA_DEM1             2.513274e-01 3.806734e-02  0.92384737
## CIA_CKD1             4.018162e+00 2.014114e+00  7.88934637
## CIA_CA1              4.969856e+00 1.949554e+00 11.75436099
## CIA_CA_MET1          6.994795e+00 1.878990e+00 20.90930168
## PREOP_HB             8.212051e-01 7.083688e-01  0.94773127
## PREOP_WCC            1.058855e+00 1.016937e+00  1.10763144
## PREOP_NA             1.071683e+00 9.963073e-01  1.15655146
## SURG_SEV1            1.126621e+00 5.706878e-01  2.24886757
## SURG_SEV2            2.422828e+00 1.055064e+00  5.55506995
## SURG_SCHEDULED_TYPE1 5.305949e+00 2.445200e+00 12.58579783
## PreopICU1            3.632720e+00 8.625585e-01 13.34566104
```

```
# ANOVA for individual terms: requires library "car"
car::Anova(lrdeathih.org.final, type = "II", test = "Wald")
```

```
## Analysis of Deviance Table (Type II tests)
##
## Response: DEATH_INHOSPITAL
##                     Df   Chisq Pr(>Chisq)
## CIA_CHF              1  5.2894  0.0214552 *
## CIA_CEVD             1  2.4736  0.1157711
## CIA_DEM              1  3.1555  0.0756735 .
## CIA_CKD              1 16.0871  6.050e-05 ***
## CIA_CA               1 12.5348  0.0003994 ***
## CIA_CA_MET           1 10.4922  0.0011988 **
## PREOP_HB             1  7.0710  0.0078339 **
## PREOP_WCC            1  6.9063  0.0085892 **
## PREOP_NA             1  3.3277  0.0681204 .
## SURG_SEV             2  5.1916  0.0745865 .
## SURG_SCHEDULED_TYPE  1 16.2594  5.523e-05 ***
## PreopICU             1  3.5308  0.0602389 .
## ---
## Signif. codes:  0 '***' 0.001 '**' 0.01 '*' 0.05 '.' 0.1 ' ' 1
```

```
# Pseudo R sqaured: requires library "rcompanion"
rcompanion::nagelkerke(lrdeathih.org.final)
```

```
## $Models
##
## Model: "glm, DEATH_INHOSPITAL ~ CIA_CHF + CIA_CEVD + CIA_DEM + CIA_CKD + CIA_CA + CIA_CA_MET + PR
EOP_HB + PREOP_WCC + PREOP_NA + SURG_SEV + SURG_SCHEDULED_TYPE + PreopICU, binomial, train.org.na, n
a.omit"
## Null:  "glm, DEATH_INHOSPITAL ~ 1, binomial, train.org.na, na.omit"
##
## $Pseudo.R.squared.for.model.vs.null
##                              Pseudo.R.squared
## McFadden                            0.2551090
## Cox and Snell (ML)                  0.0803662
## Nagelkerke (Cragg and Uhler)        0.2870930
##
## $Likelihood.ratio.test
##  Df.diff LogLik.diff  Chisq    p.value
##      -13     -65.809 131.62 9.9574e-22
##
## $Number.of.observations
##
## Model: 1571
## Null:  1571
##
## $Messages
## [1] "Note: For models fit with REML, these statistics are based on refitting with ML"
##
## $Warnings
## [1] "None"
```

```
# Overall p value for model
anova(lrdeathih.org.final
      , update(lrdeathih.org.final, ~1) # update here produces null model fo comparison
      , test = "Chisq")
```

```
## Analysis of Deviance Table
##
## Model 1: DEATH_INHOSPITAL ~ CIA_CHF + CIA_CEVD + CIA_DEM + CIA_CKD + CIA_CA +
##     CIA_CA_MET + PREOP_HB + PREOP_WCC + PREOP_NA + SURG_SEV +
##     SURG_SCHEDULED_TYPE + PreopICU
## Model 2: DEATH_INHOSPITAL ~ 1
##   Resid. Df Resid. Dev  Df Deviance  Pr(>Chi)
## 1      1557     384.31
## 2      1570     515.93 -13  -131.62 < 2.2e-16 ***
## ---
## Signif. codes:  0 '***' 0.001 '**' 0.01 '*' 0.05 '.' 0.1 ' ' 1
```

```
# Standardized residuals
plot(fitted(lrdeathih.org.final)
     , rstandard(lrdeathih.org.final))
```

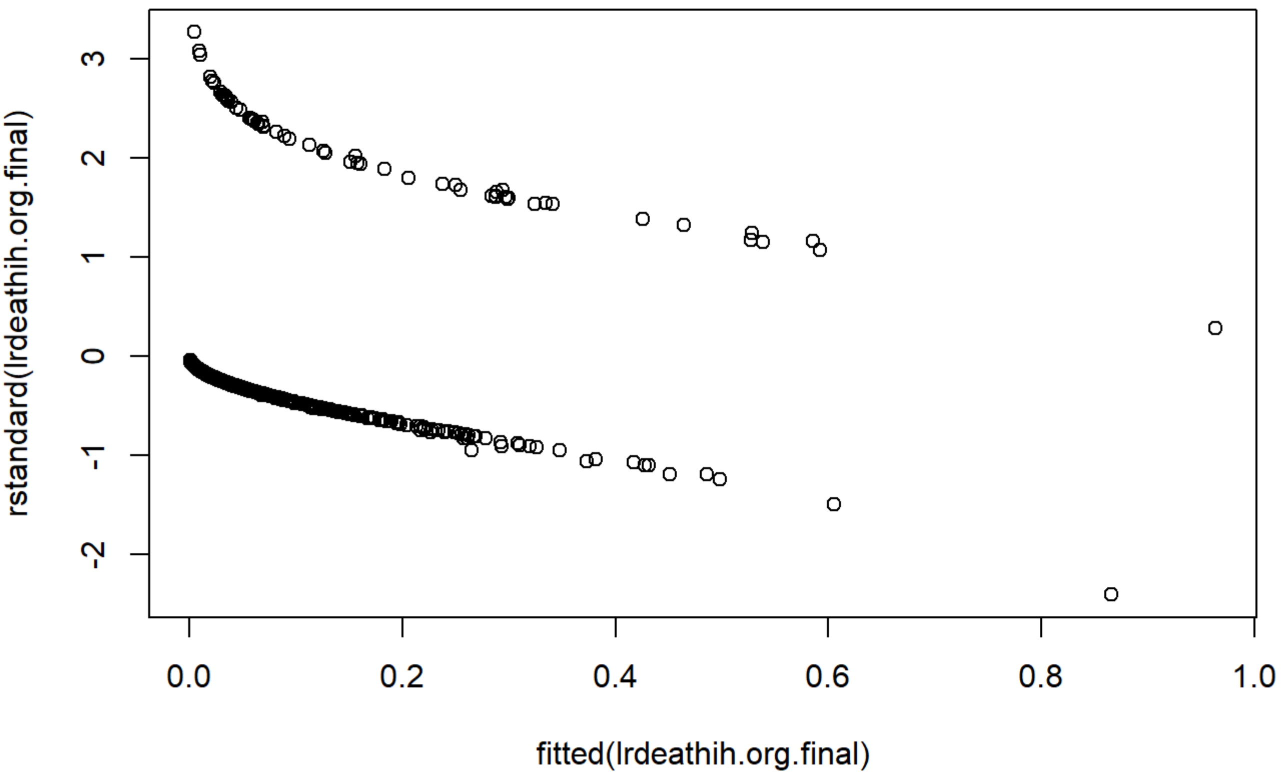


```
plot(lrdeathih.org.final)
```

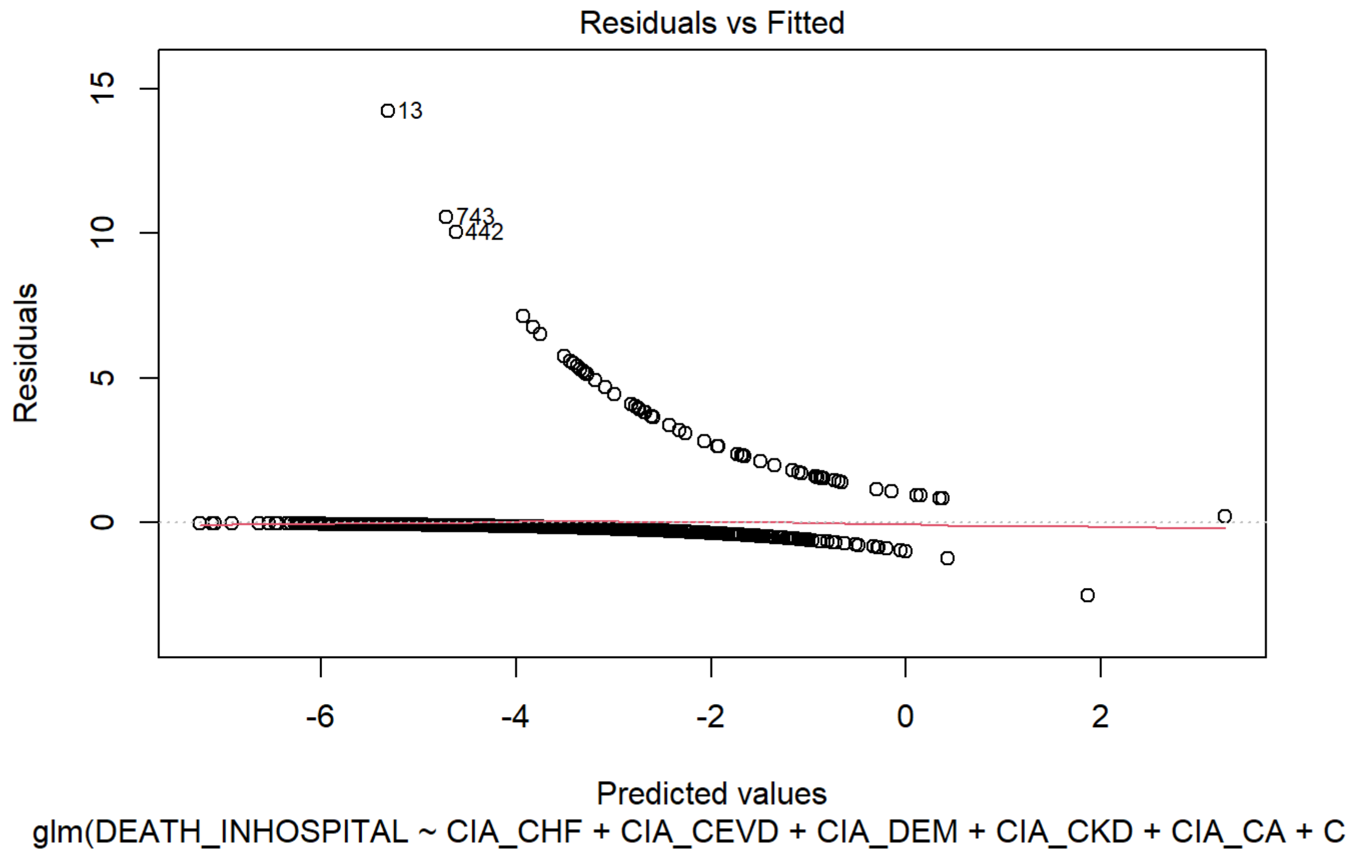
Residuals vs Fitted
Residuals
15
10
5
0
13
743
442
-6
-4
-2
0
2
Predicted values
glm(DEATH_INHOSPITAL ~ CIA_CHF + CIA_CEVD + CIA_DEM + CIA_CKD + CIA_CA + C

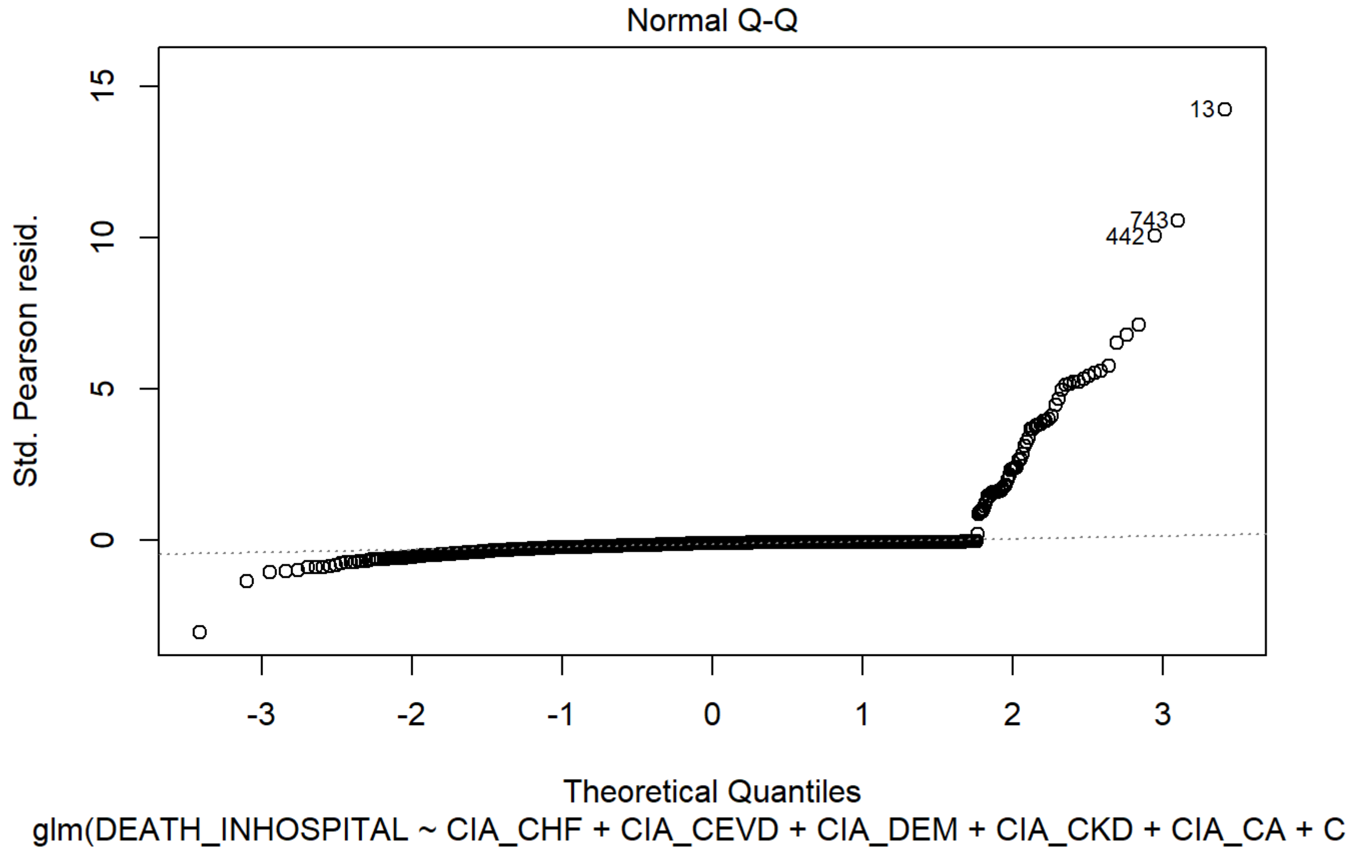
Normal Q-Q
Std. Pearson resid.
15
10
5
0
13
743
442
-3
-2
-1
0
1
2
3
Theoretical Quantiles
glm(DEATH_INHOSPITAL ~ CIA_CHF + CIA_CEVD + CIA_DEM + CIA_CKD + CIA_CA + C

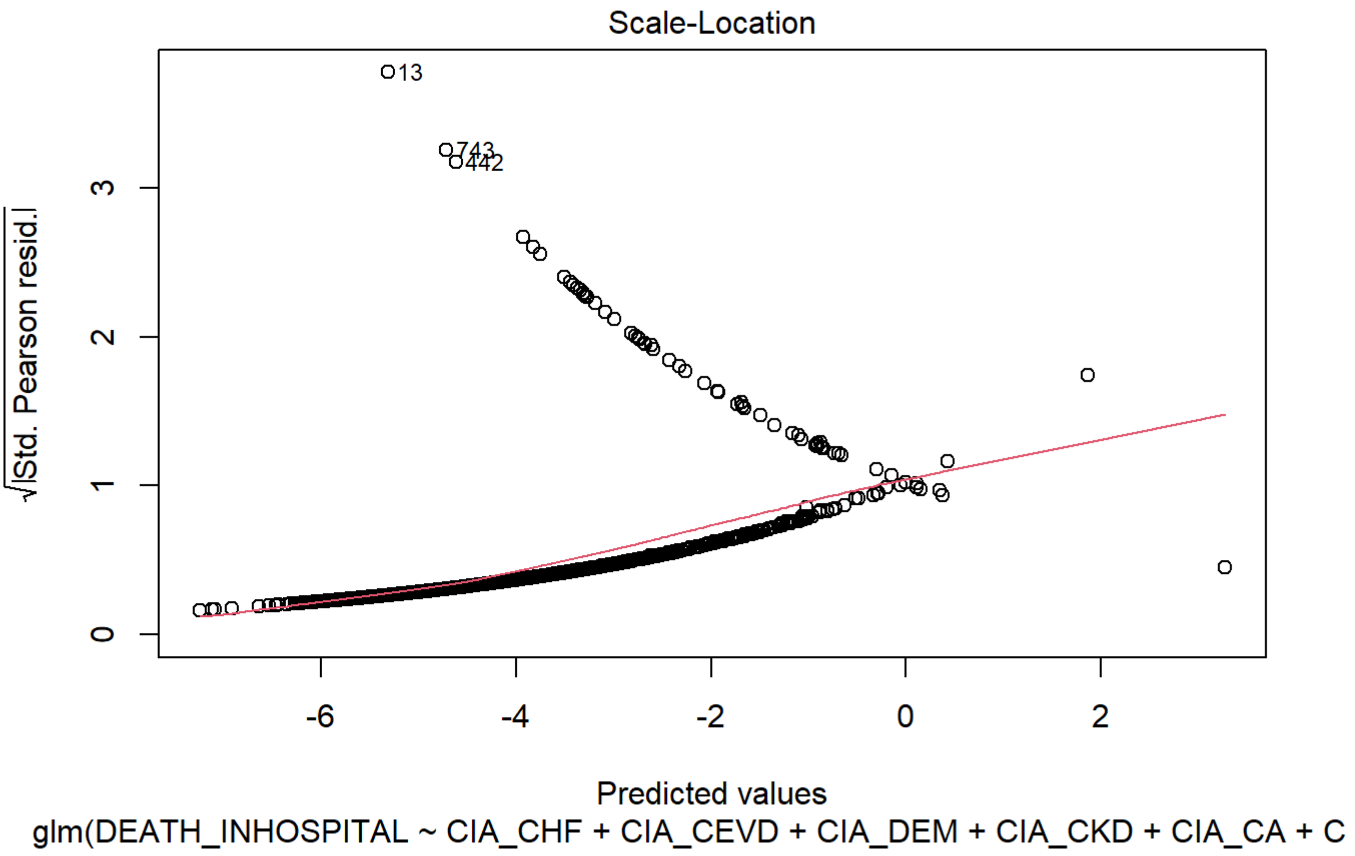
Scale-Location
13
743
442
√|Std. Pearson resid.|
Predicted values
glm(DEATH_INHOSPITAL ~ CIA_CHF + CIA_CEVD + CIA_DEM + CIA_CKD + CIA_CA + C

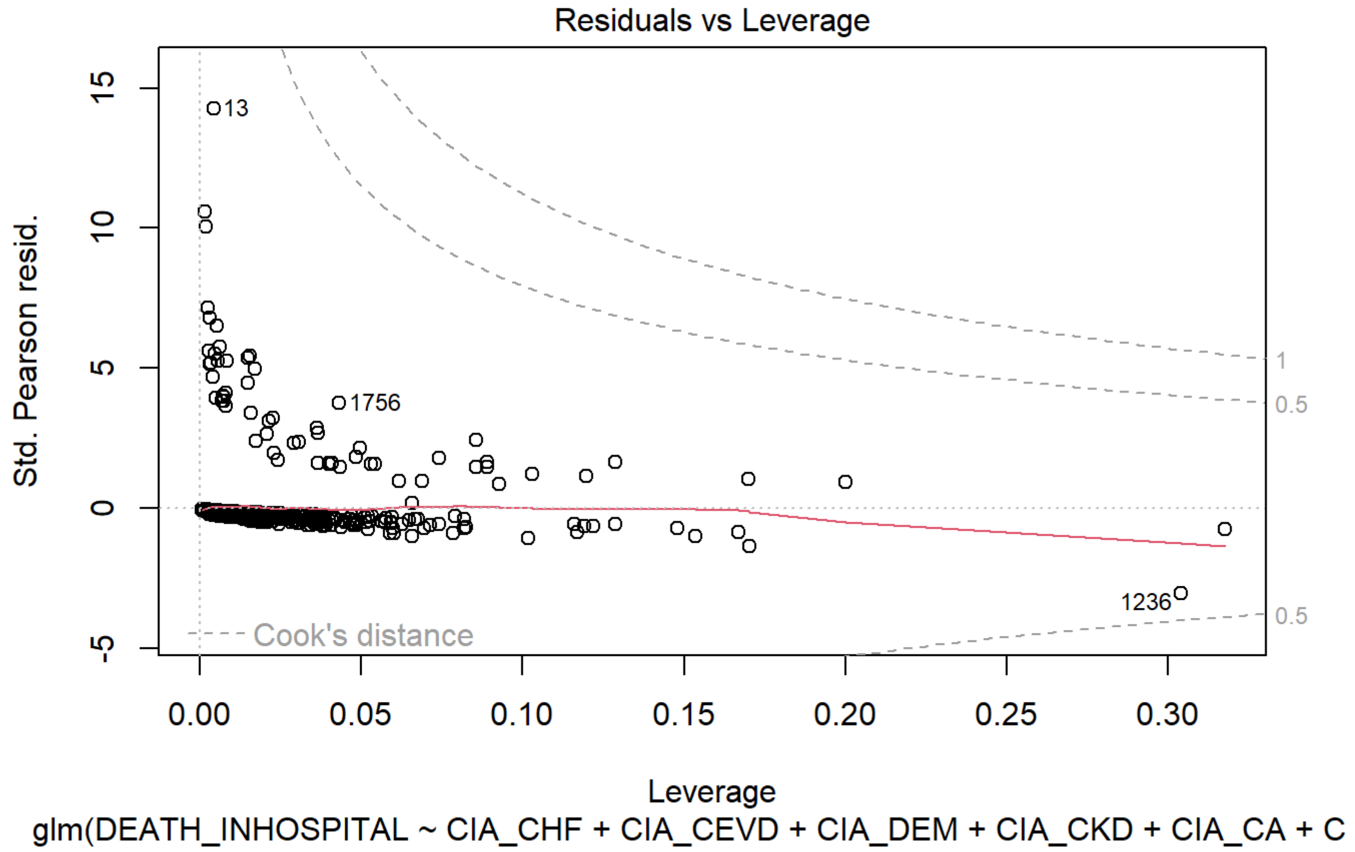
Residuals vs Leverage
13
1756
1236
Cook's distance
Std. Pearson resid.
Leverage
glm(DEATH_INHOSPITAL ~ CIA_CHF + CIA_CEVD + CIA_DEM + CIA_CKD + CIA_CA + C

```
#
#
#
#

#Classification table (confusion matrix)
lrdeathih.org.final.pred <- predict(lrdeathih.org.final
                                , newdata = train.org.na
                                , type = "response")
lrdeathih.org.final.class <- ifelse(lrdeathih.org.final.pred>=0.5, 1, 0)
cl_table.lrdeathih.org <- table (train.org.na$DEATH_INHOSPITAL, lrdeathih.org.final.class)
rownames(cl_table.lrdeathih.org) <- c("Alive", "Death in-hospital")
colnames(cl_table.lrdeathih.org) <- c("Alive", "Death in-hospital")
addmargins(cl_table.lrdeathih.org)
```

```
##                    lrdeathih.org.final.class
##                     Alive Death in-hospital  Sum
##   Alive              1508                 2 1510
##   Death in-hospital    55                 6   61
##   Sum                1563                 8 1571
```

```
#model accuracy (classification accuracy)
mean((train.org.na$DEATH_INHOSPITAL == lrdeathih.org.final.class))
```

```
## [1] 0.9637174
```

```
#Discriminability

roc.lrdeathih.train <- roc(train.org.na$DEATH_INHOSPITAL
                        ,lrdeathih.org.final.pred
                        , add = TRUE)
roc.lrdeathih.train$auc
```

```
## Area under the curve: 0.8742
```

```
ci(roc.lrdeathih.train)
```

```
## 95% CI: 0.8362-0.9121 (DeLong)
```

```
plot.roc(roc.lrdeathih.train
         , print.auc = TRUE
         , print.thres = FALSE
         , auc.polygon = TRUE
)
```

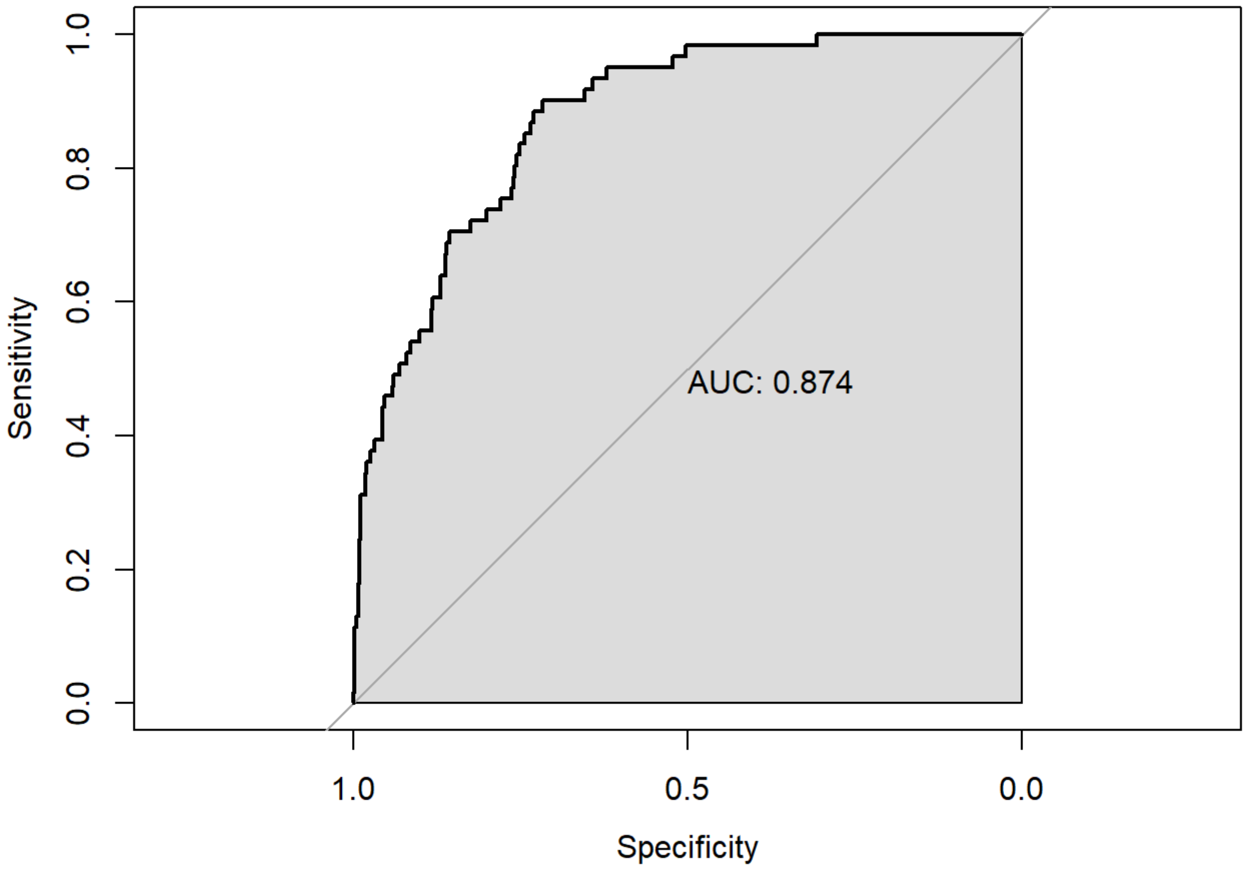


```
pROC::coords(roc.lrdeathih.train,
             "best",
             transpose = TRUE,
             best.method = "youden")
```

```
##   threshold specificity sensitivity
##  0.02924449  0.71655629  0.90163934
```

8.2 Imputed data analysis

8.2.1 Pooled logistic regression model

This is a logistic regression model with imputed datasets, results presented as “Pooled” coefficients.

```
# Logistic regression model with imput data: Enter all input parameters
lrdeathih.imp.full <- with(data = train.imp
                       , exp = glm(DEATH_INHOSPITAL ~ GENDER
                                   + CIA_AMI
                                   + CIA_CHF
                                   + CIA_PVD
                                   + CIA_CEVD
                                   + CIA_DEM
                                   + CIA_COPD
                                   + CIA_PUD
                                   + CIA_LD_MLD
                                   + CIA_LD_SEV
                                   + CIA_DM
                                   + CIA_DM_CX
                                   + CIA_HEMIPLAEGIA
                                   + CIA_CKD
                                   + CIA_CA
                                   + CIA_CA_MET
                                   + PREOP_HB
                                   + PREOP_WCC
                                   + PREOP_NA
                                   + PREOP_K
                                   + SURG_SEV
                                   + SURG_SCHEDULED_TYPE
                                   + PreopICU
                                   , family = "binomial"))

summary(pool(lrdeathih.imp.full))
```

```
##                   term     estimate    std.error    statistic         df
## 1          (Intercept) -12.62961929 5.635928e+00 -2.240911999  693.27938
## 2              GENDER1  -0.18376546 2.868971e-01 -0.640527495 1905.50320
## 3             CIA_AMI1 -15.17279347 1.213071e+03 -0.012507754 1940.79957
## 4             CIA_CHF1   0.95001535 4.352330e-01  2.182774139 1827.91157
## 5             CIA_PVD1   0.79931613 6.466452e-01  1.236096958 1932.71835
## 6            CIA_CEVD1   1.14485908 6.152774e-01  1.860720123 1914.29088
## 7             CIA_DEM1  -1.05103282 6.607579e-01 -1.590647311 1940.08439
## 8            CIA_COPD1   0.15204317 8.089016e-01  0.187962502 1902.36462
## 9             CIA_PUD1  -1.20578876 1.198262e+00 -1.006281092 1903.50717
## 10         CIA_LD_MLD1 -13.29535527 1.504412e+03 -0.008837576 1940.79957
## 11         CIA_LD_SEV1 -13.15910347 2.226768e+03 -0.005909508 1940.79957
## 12             CIA_DM1   0.26022977 5.237784e-01  0.496831779 1910.70488
## 13          CIA_DM_CX1  -0.26964128 4.246822e-01 -0.634924887 1934.11634
## 14     CIA_HEMIPLAEGIA1   0.20986678 8.547649e-01  0.245525733 1906.28469
## 15            CIA_CKD1   1.41465457 3.664362e-01  3.860575698 1932.10560
## 16             CIA_CA1   1.41340885 4.437442e-01  3.185188240 1889.37385
## 17         CIA_CA_MET1   2.09737924 5.683954e-01  3.690000466 1647.85288
## 18            PREOP_HB  -0.23089534 8.193358e-02 -2.818079484   67.90338
## 19           PREOP_WCC   0.06972706 2.520327e-02  2.766587728  119.16019
## 20            PREOP_NA   0.07274125 3.791429e-02  1.918570736  494.64929
## 21             PREOP_K  -0.10877573 2.836323e-01 -0.383509605  181.58443
## 22           SURG_SEV1   0.06004308 3.392541e-01  0.176985583 1924.54933
## 23           SURG_SEV2   0.92225784 4.100522e-01  2.249122826 1743.64886
## 24 SURG_SCHEDULED_TYPE1   1.60196371 3.814127e-01  4.200079460 1602.15336
## 25           PreopICU1   1.37180220 7.167238e-01  1.913990081 1914.80649
##         p.value
## 1  2.534759e-02
## 2  5.219067e-01
## 3  9.900218e-01
## 4  2.917931e-02
## 5  2.165727e-01
## 6  6.293694e-02
## 7  1.118519e-01
## 8  8.509261e-01
## 9  3.144083e-01
## 10 9.929496e-01
## 11 9.952855e-01
## 12 6.193648e-01
## 13 5.255525e-01
## 14 8.060758e-01
## 15 1.168399e-04
## 16 1.470191e-03
## 17 2.315444e-04
## 18 6.322927e-03
## 19 6.568485e-03
## 20 5.561392e-02
## 21 7.017906e-01
## 22 8.595383e-01
## 23 2.462904e-02
## 24 2.815058e-05
## 25 5.577035e-02
```

```
# Stepwise selection method: AIC guided.
# Suppressed output
lrdeathih.imp.sel <- with(data = train.imp
                      , exp = step(glm(DEATH_INHOSPITAL ~ GENDER
                                       + CIA_AMI
                                       + CIA_CHF
                                       + CIA_PVD
                                       + CIA_CEVD
                                       + CIA_DEM
                                       + CIA_COPD
                                       + CIA_PUD
                                       + CIA_LD_MLD
                                       + CIA_LD_SEV
                                       + CIA_DM
                                       + CIA_DM_CX
                                       + CIA_HEMIPLAEGIA
                                       + CIA_CKD
                                       + CIA_CA
                                       + CIA_CA_MET
                                       + PREOP_HB
                                       + PREOP_WCC
                                       + PREOP_NA
                                       + PREOP_K
                                       + SURG_SEV
                                       + SURG_SCHEDULED_TYPE
                                       + PreopICU
                                       , family = binomial)))
```

```
summary(pool(lrdeathih.imp.sel))
```

```
##                    term     estimate    std.error   statistic         df
## 1           (Intercept) -12.72458835   5.29669085 -2.40236568  453.69629
## 2              CIA_AMI1 -14.37731164 739.90407515 -0.01943132 1950.78894
## 3              CIA_CHF1   0.95366513   0.42764685  2.23002957 1913.05236
## 4             CIA_CEVD1   1.26840983   0.49624638  2.55600825 1862.29649
## 5              CIA_DEM1  -1.04243803   0.64670038 -1.61193353 1949.44702
## 6              CIA_CKD1   1.25071963   0.33881948  3.69140417 1923.60418
## 7               CIA_CA1   1.43847480   0.43924472  3.27488237 1919.62190
## 8           CIA_CA_MET1   2.10565262   0.55063407  3.82405073 1850.31475
## 9              PREOP_HB  -0.22577555   0.08026700 -2.81280655   60.94071
## 10            PREOP_WCC   0.06747883   0.02491480  2.70838347   94.98627
## 11             PREOP_NA   0.06871595   0.03707833  1.85326422  495.06361
## 12            SURG_SEV1   0.19554444   0.32995991  0.59263090 1806.80135
## 13            SURG_SEV2   1.01993895   0.40396921  2.52479378 1530.04219
## 14 SURG_SCHEDULED_TYPE1   1.61412129   0.37953910  4.25284587 1304.04886
## 15            PreopICU1   1.16730182   0.68712538  1.69881924 1930.18089
##         p.value
## 1  1.669030e-02
## 2  9.844990e-01
## 3  2.586110e-02
## 4  1.066683e-02
## 5  1.071383e-01
## 6  2.292301e-04
## 7  1.075869e-03
## 8  1.356259e-04
## 9  6.600253e-03
## 10 8.019899e-03
## 11 6.443927e-02
## 12 5.535024e-01
## 13 1.167700e-02
## 14 2.261253e-05
## 15 8.951440e-02
```

```
# Final logistic regression model with the selected input parameters
lrdeathih.imp.final <- with(data = train.imp
                        , exp = glm(DEATH_INHOSPITAL ~ CIA_AMI
                                    + CIA_CHF
                                    + CIA_CEVD
                                    + CIA_DEM
                                    + CIA_CKD
                                    + CIA_CA
                                    + CIA_CA_MET
                                    + PREOP_HB
                                    + PREOP_WCC
                                    + PREOP_NA
                                    + SURG_SEV
                                    + SURG_SCHEDULED_TYPE
                                    + PreopICU
                                    , family = binomial))
pool(lrdeathih.imp.final)
```

```
## Class: mipo    m = 5
##                    term m     estimate         ubar            b            t
## 1           (Intercept) 5 -12.57575131 2.574565e+01 2.043534e+00 2.819789e+01
## 2              CIA_AMI1 5 -14.36215963 5.476270e+05 7.699101e-04 5.476270e+05
## 3              CIA_CHF1 5   0.94934713 1.828569e-01 9.299564e-04 1.839728e-01
## 4             CIA_CEVD1 5   1.26188213 2.445011e-01 2.261720e-03 2.472152e-01
## 5              CIA_DEM1 5  -1.05310862 4.184874e-01 2.273827e-04 4.187603e-01
## 6              CIA_CKD1 5   1.26509310 1.149888e-01 3.381717e-04 1.153946e-01
## 7               CIA_CA1 5   1.43079037 1.922181e-01 8.820879e-04 1.932767e-01
## 8           CIA_CA_MET1 5   2.10252930 3.002496e-01 2.382872e-03 3.031090e-01
## 9              PREOP_HB 5  -0.22557853 4.822770e-03 1.309044e-03 6.393622e-03
## 10            PREOP_WCC 5   0.06804795 5.000192e-04 9.716816e-05 6.166210e-04
## 11             PREOP_NA 5   0.06767241 1.268278e-03 9.516885e-05 1.382481e-03
## 12            SURG_SEV1 5   0.18123910 1.080738e-01 4.189412e-04 1.085766e-01
## 13            SURG_SEV2 5   1.01129062 1.599279e-01 2.767196e-03 1.632486e-01
## 14 SURG_SCHEDULED_TYPE1 5   1.61756244 1.395373e-01 3.699970e-03 1.439773e-01
## 15            PreopICU1 5   1.18083793 4.753842e-01 1.932027e-03 4.777026e-01
##    dfcom         df          riv       lambda         fmi
## 1   1953  407.80999 9.524874e-02 8.696540e-02 0.091410444
## 2   1953 1950.79845 1.687083e-09 1.687083e-09 0.001023649
## 3   1953 1905.18474 6.102847e-03 6.065828e-03 0.007107587
## 4   1953 1823.55772 1.110042e-02 1.097855e-02 0.012061484
## 5   1953 1949.32841 6.520131e-04 6.515882e-04 0.001675339
## 6   1953 1932.52595 3.529090e-03 3.516679e-03 0.004546357
## 7   1953 1912.49281 5.506793e-03 5.476634e-03 0.006515034
## 8   1953 1852.92589 9.523563e-03 9.433720e-03 0.010501183
## 9   1953   63.40965 3.257159e-01 2.456906e-01 0.268407455
## 10  1953  104.47579 2.331946e-01 1.890980e-01 0.204187941
## 11  1953  441.56136 9.004542e-02 8.260704e-02 0.086734220
## 12  1953 1921.96521 4.651722e-03 4.630184e-03 0.005664353
## 13  1953 1595.81766 2.076332e-02 2.034097e-02 0.021566450
## 14  1953 1304.44294 3.181919e-02 3.083795e-02 0.032320479
## 15  1953 1919.58769 4.876966e-03 4.853297e-03 0.005888513
```

```
lrdeathih.imp.final.est <- summary(pool(lrdeathih.imp.final))

lrdeathih.imp.final.est %>%
  mutate(OR = exp(estimate)
         , Low.CI = exp(estimate - 1.96*std.error)
         , Up.CI = exp(estimate + 1.96*std.error)
         , Sig = ifelse(p.value < 0.05, "*","")
  ) %>%
  select(-c("statistic", "df"))
```

```
##                    term     estimate    std.error      p.value           OR
## 1           (Intercept) -12.57575131   5.31016900 1.833847e-02 3.454782e-06
## 2              CIA_AMI1 -14.36215963 740.01822829 9.845177e-01 5.788864e-07
## 3              CIA_CHF1   0.94934713   0.42892056 2.699229e-02 2.584022e+00
## 4             CIA_CEVD1   1.26188213   0.49720740 1.123335e-02 3.532063e+00
## 5              CIA_DEM1  -1.05310862   0.64711691 1.038169e-01 3.488516e-01
## 6              CIA_CKD1   1.26509310   0.33969788 2.015748e-04 3.543423e+00
## 7               CIA_CA1   1.43079037   0.43963241 1.155682e-03 4.182003e+00
## 8           CIA_CA_MET1   2.10252930   0.55055340 1.384386e-04 8.186851e+00
## 9              PREOP_HB  -0.22557853   0.07996013 6.381614e-03 7.980544e-01
## 10            PREOP_WCC   0.06804795   0.02483185 7.219697e-03 1.070417e+00
## 11             PREOP_NA   0.06767241   0.03718172 6.942904e-02 1.070015e+00
## 12            SURG_SEV1   0.18123910   0.32950958 5.823649e-01 1.198702e+00
## 13            SURG_SEV2   1.01129062   0.40404032 1.241576e-02 2.749147e+00
## 14 SURG_SCHEDULED_TYPE1   1.61756244   0.37944338 2.162292e-05 5.040788e+00
## 15            PreopICU1   1.18083793   0.69116033 8.770782e-02 3.257102e+00
##          Low.CI      Up.CI Sig
## 1  1.043070e-10  0.1144268   *
## 2  0.000000e+00        Inf
## 3  1.114786e+00  5.9896407   *
## 4  1.332898e+00  9.3596570   *
## 5  9.813043e-02  1.2401602
## 6  1.820817e+00  6.8957201   *
## 7  1.766696e+00  9.8993578   *
## 8  2.782764e+00 24.0855954   *
## 9  6.822901e-01  0.9334605   *
## 10 1.019566e+00  1.1238029   *
## 11 9.948097e-01  1.1509050
## 12 6.283866e-01  2.2866272
## 13 1.245294e+00  6.0690959   *
## 14 2.396126e+00 10.6044260   *
## 15 8.404344e-01 12.6228945
```

8.2.1.1 Object for the combined-predict probability and model diagnostics

```
# Pooled logistic regression model object

# Copy one of the fitted lr models
lrdeathih.pool <- lrdeathih.imp.final$analyses[1]

# Pooled coefficients
repl.pool <- summary(pool(lrdeathih.imp.final))$estimate

# Insert name: named numeric vector objective
names(repl.pool) <- names(lrdeathih.pool[[1]]$coefficients)

# Replace the fitted coefficents with the pooled estimates
lrdeathih.pool[[1]]$coefficients <- repl.pool
lrdeathih.imp <- lrdeathih.pool[[1]]

# Check the replaced result

# Coefficient: imputed data 1
lrdeathih.imp.final$analyses[1]
```

```
## [[1]]
## 
## Call:  glm(formula = DEATH_INHOSPITAL ~ CIA_AMI + CIA_CHF + CIA_CEVD + 
##     CIA_DEM + CIA_CKD + CIA_CA + CIA_CA_MET + PREOP_HB + PREOP_WCC + 
##     PREOP_NA + SURG_SEV + SURG_SCHEDULED_TYPE + PreopICU, family = binomial)
## 
## Coefficients:
##          (Intercept)              CIA_AMI1              CIA_CHF1  
##            -14.22859             -14.36870               0.93111  
##            CIA_CEVD1              CIA_DEM1              CIA_CKD1  
##              1.26532              -1.03407               1.25721  
##              CIA_CA1           CIA_CA_MET1              PREOP_HB  
##              1.42222               2.03876              -0.20356  
##            PREOP_WCC              PREOP_NA             SURG_SEV1  
##              0.06649               0.07776               0.16491  
##            SURG_SEV2  SURG_SCHEDULED_TYPE1             PreopICU1  
##              0.98179               1.66728               1.14107  
## 
## Degrees of Freedom: 1967 Total (i.e. Null);  1953 Residual
## Null Deviance:       604.6 
## Residual Deviance: 445.2     AIC: 475.2
```

```
# Coefficient: pooled model generated
lrdeathih.imp
```

```
## 
## Call:  glm(formula = DEATH_INHOSPITAL ~ CIA_AMI + CIA_CHF + CIA_CEVD + 
##     CIA_DEM + CIA_CKD + CIA_CA + CIA_CA_MET + PREOP_HB + PREOP_WCC + 
##     PREOP_NA + SURG_SEV + SURG_SCHEDULED_TYPE + PreopICU, family = binomial)
## 
## Coefficients:
##          (Intercept)              CIA_AMI1              CIA_CHF1  
##            -12.57575             -14.36216               0.94935  
##            CIA_CEVD1              CIA_DEM1              CIA_CKD1  
##              1.26188              -1.05311               1.26509  
##              CIA_CA1           CIA_CA_MET1              PREOP_HB  
##              1.43079               2.10253              -0.22558  
##            PREOP_WCC              PREOP_NA             SURG_SEV1  
##              0.06805               0.06767               0.18124  
##            SURG_SEV2  SURG_SCHEDULED_TYPE1             PreopICU1  
##              1.01129               1.61756               1.18084  
## 
## Degrees of Freedom: 1967 Total (i.e. Null);  1953 Residual
## Null Deviance:       604.6 
## Residual Deviance: 445.2     AIC: 475.2
```

```
# Coefficient: estimated pooled model
summary(pool(lrdeathih.imp.final))$estimate
```

```
##  [1] -12.57575131 -14.36215963   0.94934713   1.26188213  -1.05310862
##  [6]   1.26509310   1.43079037   2.10252930  -0.22557853   0.06804795
## [11]   0.06767241   0.18123910   1.01129062   1.61756244   1.18083793
```

Then, we can get the combined-predict probability

```
# Combined-predict probability of training dataset
lrdeathih.imp.train.pred <- predict(lrdeathih.imp
                                , newdata = train.org.na
                                , type = "response")
lrdeathih.imp.train.class <- ifelse(lrdeathih.imp.train.pred>=0.5, 1, 0)

# Confusion matrix
cl_table.lrdeathih.imp.train <- table (train.org.na$DEATH_INHOSPITAL, lrdeathih.imp.train.class)
rownames(cl_table.lrdeathih.imp.train) <- c("Alive", "Death in-hospital")
colnames(cl_table.lrdeathih.imp.train) <- c("Alive", "Death in-hospital")
addmargins(cl_table.lrdeathih.imp.train)
```

```
##                    lrdeathih.imp.train.class
##                     Alive Death in-hospital  Sum
##   Alive              1506                 4 1510
##   Death in-hospital    56                 5   61
##   Sum                1562                 9 1571
```

```
# Sensitivity
round(cl_table.lrdeathih.imp.train[2,2]/(cl_table.lrdeathih.imp.train[2,1] + cl_table.lrdeathih.imp.
train[2,2]),3)
```

```
## [1] 0.082
```

```
# Specificity
round(cl_table.lrdeathih.imp.train[1,1]/(cl_table.lrdeathih.imp.train[1,1] + cl_table.lrdeathih.imp.
train[1,2]),3)
```

```
## [1] 0.997
```

```
# Positive predictive rate
round(cl_table.lrdeathih.imp.train[2,2]/(cl_table.lrdeathih.imp.train[1,2] + cl_table.lrdeathih.imp.
train[2,2]),3)
```

```
## [1] 0.556
```

```
# Negative predictive rate
round(cl_table.lrdeathih.imp.train[1,1]/(cl_table.lrdeathih.imp.train[1,1] + cl_table.lrdeathih.imp.
train[2,1]),3)
```

```
## [1] 0.964
```

```
# Model accuracy (classification accuracy)
mean((train.org.na$DEATH_INHOSPITAL == lrdeathih.imp.train.class))
```

```
## [1] 0.9618078
```

```
# Discriminability
roc.lrdeathih.imp.train <- roc(train.org.na$DEATH_INHOSPITAL
                           ,lrdeathih.imp.train.pred
                           , add = TRUE)
```

```
## Setting levels: control = 0, case = 1
```

```
## Setting direction: controls < cases
```

```
roc.lrdeathih.imp.train$auc
```

```
## Area under the curve: 0.8795
```

```
ci(roc.lrdeathih.imp.train)
```

```
## 95% CI: 0.8443-0.9146 (DeLong)
```

```
plot.roc(roc.lrdeathih.imp.train
         , print.auc = TRUE
         , print.thres = FALSE
         , auc.polygon = TRUE
)
```

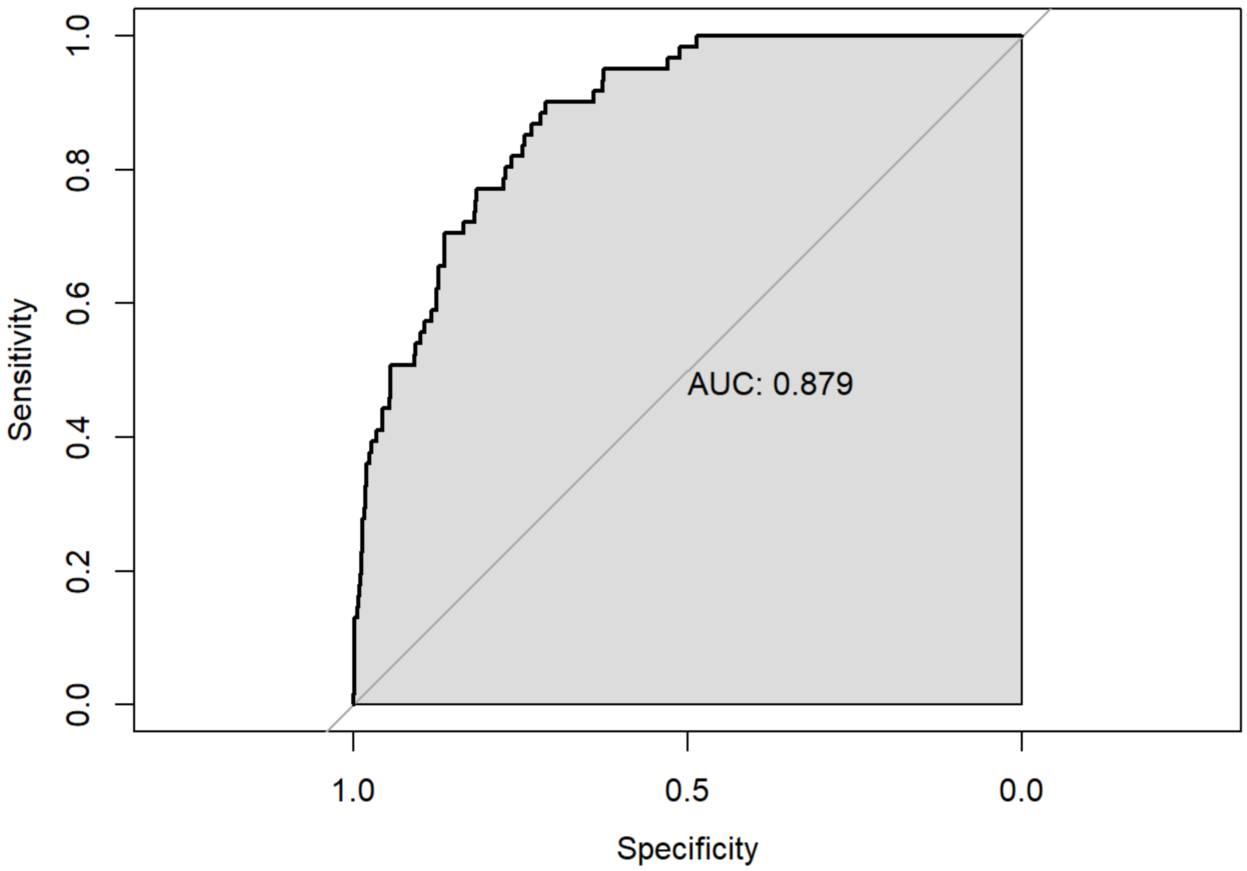


```
pROC::coords(roc.lrdeathih.imp.train,
             "best",
             transpose = TRUE,
             best.method = "youden")
```

```
##   threshold specificity sensitivity
##  0.02957133  0.71258278  0.90163934
```

Using the estimated threshold from ROC (0.02957133), make a variable of assisting clinical decision of Yes/No.

```
#Threshold discrimination ability

lrdeathih.imp.train.pred.dcs <- ifelse(lrdeathih.imp.train.pred > 0.02957133, 1, 0)

roc.lrdeathih.imp.train.dcs <- roc(train.org.na$DEATH_INHOSPITAL
                              ,lrdeathih.imp.train.pred.dcs
                              , add = TRUE)
```

```
## Setting levels: control = 0, case = 1
```

```
## Setting direction: controls < cases
```

```
roc.lrdeathih.imp.train.dcs$auc
```

```
## Area under the curve: 0.8071
```

```
ci(roc.lrdeathih.imp.train.dcs)
```

```
## 95% CI: 0.7677-0.8465 (DeLong)
```

```
plot.roc(roc.lrdeathih.imp.train.dcs
         , print.auc = TRUE
         , print.thres = FALSE
         , auc.polygon = TRUE
)
```

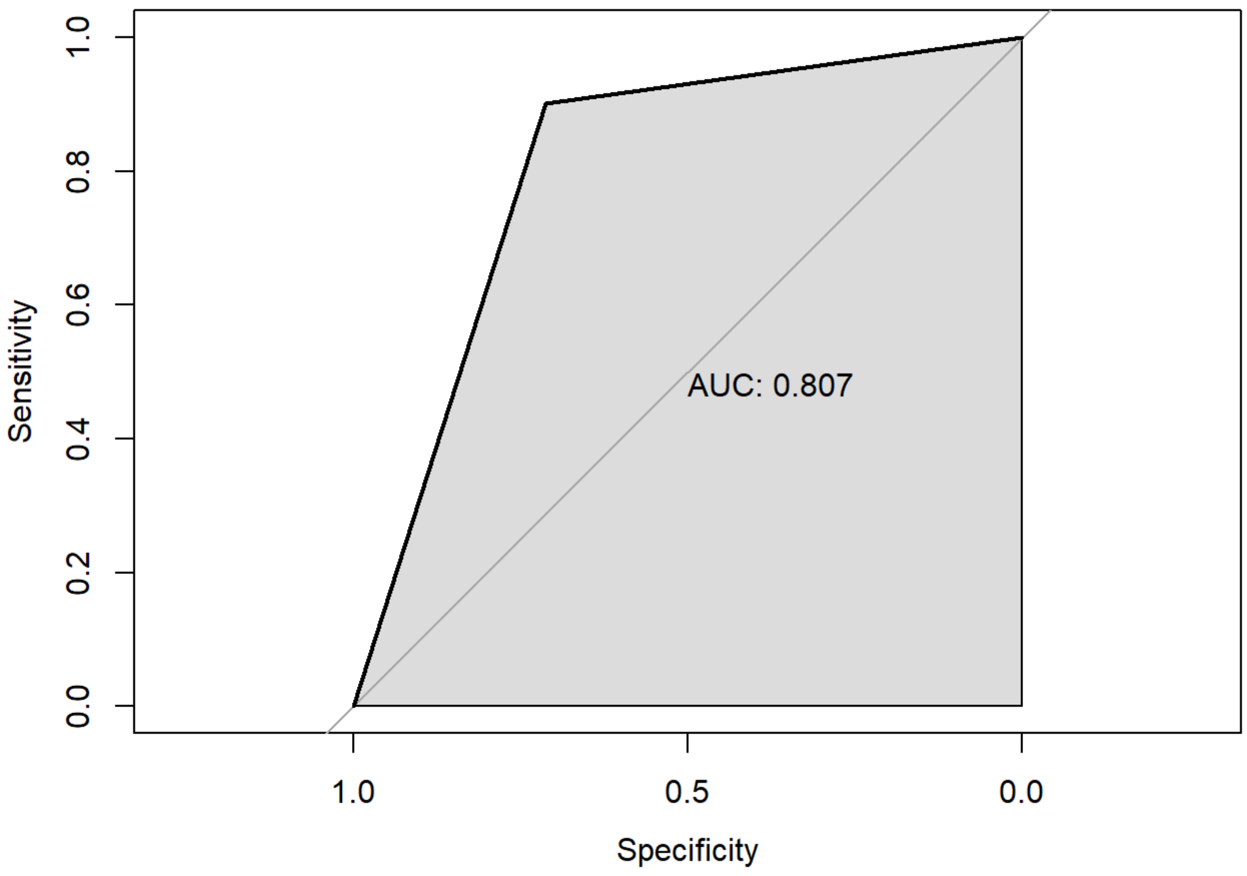

```
pROC::coords(roc.lrdeathih.imp.train.dcs,
             "best",
             transpose = TRUE,
             best.method = "youden")
```

```
##   threshold specificity sensitivity
##   0.5000000   0.7125828   0.9016393
```

```
# Calibrations: on the training dataset
cal.lrdeathih.imp.train <- val.prob(lrdeathih.imp.train.pred#[,1]
                                , train.org.na$DEATH_INHOSPITAL
                                , pl = FALSE)

CalibrationCurves::val.prob.ci.2(lrdeathih.imp.train.pred, train.org.na$DEATH_INHOSPITAL
                                 , lty.smooth = 2
                                 , CL.smooth = FALSE
                                 , col.ideal = "black")
```

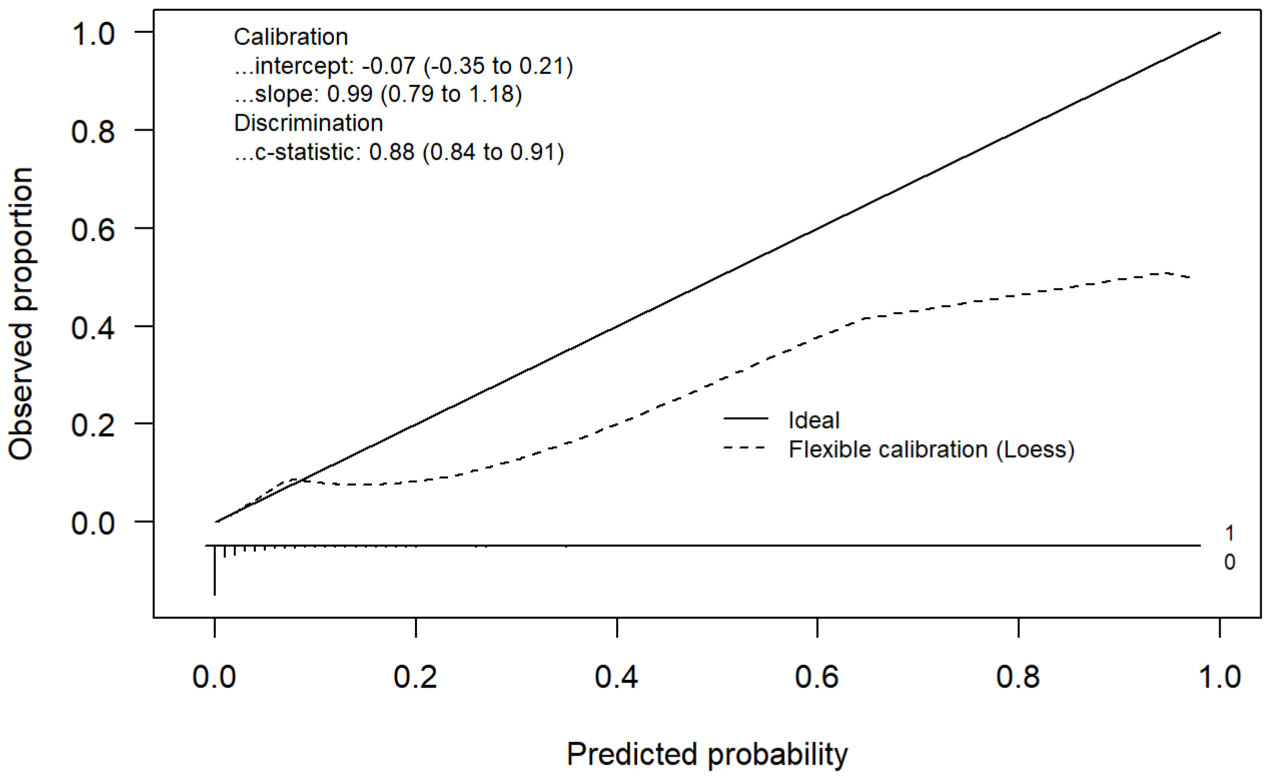

```
## Call:
## CalibrationCurves::val.prob.ci.2(p = lrdeathih.imp.train.pred,
##     y = train.org.na$DEATH_INHOSPITAL, CL.smooth = FALSE, lty.smooth = 2,
##     col.ideal = "black")
##
## A 95% confidence interval is given for the calibration intercept, calibration slope and c-statist
ic.
##
##          Dxy       C (ROC)           R2             D      D:Chi-sq
##   0.758853545   0.879426772   0.287574027   0.083289557 131.847893385
##          D:p             U      U:Chi-sq           U:p             Q
##   0.000000000  -0.001113844   0.250150579   0.882430462   0.084403401
##        Brier     Intercept         Slope          Emax  Brier scaled
##   0.032058891  -0.068314701   0.986421861   0.025447243   0.140998191
##         Eavg           ECI
##   0.014898561   0.187157664
```

```
cal.lrdeathih.imp.train
```

```
##          Dxy       C (ROC)           R2             D      D:Chi-sq
##   0.759081533   0.879540766   0.287050956   0.083130326 131.597742806
##          D:p             U      U:Chi-sq           U:p             Q
##           NA  -0.001113844   0.250150579   0.882430462   0.084244171
##        Brier     Intercept         Slope          Emax           E90
##   0.032058891  -0.099306842   0.986421861   0.271547772   0.015019801
##         Eavg           S:z           S:p
##   0.006311574  -0.189677992   0.849561468
```

```
hosmer_lemeshow(train.org.na$DEATH_INHOSPITAL, lrdeathih.imp.train.pred, 10, 'C')
```

```
## PVALUE 0.6114515
## stat 6.31985
```

```
## [1] 0.6114515
```

```
hosmer_lemeshow(train.org.na$DEATH_INHOSPITAL, lrdeathih.imp.train.pred, 10, 'H')
```

```
## PVALUE 0.02238396
## stat 17.84945
```

```
## [1] 0.02238396
```

8.3 Sensitivity analysis

Using the delta adjusted imputation dataset, compare the estimated ORs.

```
library(finalfit)
```

```
# delta = 0
lrdeathih.dlt0 <- with(data = train.imp.delta[[1]]
                  , exp = glm(DEATH_INHOSPITAL ~ CIA_AMI
                              + CIA_CHF
                              + CIA_CEVD
                              + CIA_DEM
                              + CIA_CKD
                              + CIA_CA
                              + CIA_CA_MET
                              + PREOP_HB
                              + PREOP_WCC
                              + PREOP_NA
                              + SURG_SEV
                              + SURG_SCHEDULED_TYPE
                              + PreopICU
                              , family = binomial))

lrdeathih.dlt0.est <- summary(pool(lrdeathih.dlt0)
                         , conf.int = TRUE
                         , exponentiate = TRUE)

# delta = mean + 1*SD
lrdeathih.dlt1 <- with(data = train.imp.delta[[2]]
                  , exp = glm(DEATH_INHOSPITAL ~ CIA_AMI
                              + CIA_CHF
                              + CIA_CEVD
                              + CIA_DEM
                              + CIA_CKD
                              + CIA_CA
                              + CIA_CA_MET
                              + PREOP_HB
                              + PREOP_WCC
                              + PREOP_NA
                              + SURG_SEV
                              + SURG_SCHEDULED_TYPE
                              + PreopICU
                              , family = binomial))

lrdeathih.dlt1.est <- summary(pool(lrdeathih.dlt1)
                         , conf.int = TRUE
                         , exponentiate = TRUE)

# delta = mean + 2*SD
lrdeathih.dlt2 <- with(data = train.imp.delta[[3]]
                  , exp = glm(DEATH_INHOSPITAL ~ CIA_AMI
                              + CIA_CHF
                              + CIA_CEVD
                              + CIA_DEM
                              + CIA_CKD
                              + CIA_CA
                              + CIA_CA_MET
                              + PREOP_HB
                              + PREOP_WCC
                              + PREOP_NA
                              + SURG_SEV
                              + SURG_SCHEDULED_TYPE
                              + PreopICU
                              , family = binomial))
```

```
lrdeathih.dlt2.est <- summary(pool(lrdeathih.dlt2)
                         , conf.int = TRUE
                         , exponentiate = TRUE)

lrdeathih.dlt0.est
```

```
##                    term     estimate    std.error   statistic         df
## 1           (Intercept) 3.454782e-06   5.31016900 -2.36823937  407.80999
## 2              CIA_AMI1 5.788864e-07 740.01822829 -0.01940785 1950.79845
## 3              CIA_CHF1 2.584022e+00   0.42892056  2.21334024 1905.18474
## 4             CIA_CEVD1 3.532063e+00   0.49720740  2.53793914 1823.55772
## 5              CIA_DEM1 3.488516e-01   0.64711691 -1.62738541 1949.32841
## 6              CIA_CKD1 3.543423e+00   0.33969788  3.72417133 1932.52595
## 7               CIA_CA1 4.182003e+00   0.43963241  3.25451526 1912.49281
## 8           CIA_CA_MET1 8.186851e+00   0.55055340  3.81893799 1852.92589
## 9              PREOP_HB 7.980544e-01   0.07996013 -2.82113764   63.40965
## 10            PREOP_WCC 1.070417e+00   0.02483185  2.74034922  104.47579
## 11             PREOP_NA 1.070015e+00   0.03718172  1.82004491  441.56136
## 12            SURG_SEV1 1.198702e+00   0.32950958  0.55002678 1921.96521
## 13            SURG_SEV2 2.749147e+00   0.40404032  2.50294477 1595.81766
## 14 SURG_SCHEDULED_TYPE1 5.040788e+00   0.37944338  4.26298761 1304.44294
## 15            PreopICU1 3.257102e+00   0.69116033  1.70848626 1919.58769
##         p.value        2.5 %     97.5 %
## 1  1.833847e-02 1.011445e-10  0.1180047
## 2  9.845177e-01 0.000000e+00        Inf
## 3  2.699229e-02 1.114208e+00  5.9927499
## 4  1.123335e-02 1.332060e+00  9.3655492
## 5  1.038169e-01 9.805542e-02  1.2411089
## 6  2.015748e-04 1.820080e+00  6.8985135
## 7  1.155682e-03 1.765760e+00  9.9046042
## 8  1.384386e-04 2.780857e+00 24.1021115
## 9  6.381614e-03 6.802153e-01  0.9363077
## 10 7.219697e-03 1.018986e+00  1.1244430
## 11 6.942904e-02 9.946118e-01  1.1511340
## 12 5.823649e-01 6.281384e-01  2.2875308
## 13 1.241576e-02 1.244564e+00  6.0726566
## 14 2.162292e-05 2.394505e+00 10.6116079
## 15 8.770782e-02 8.397374e-01 12.6333732
```

```
lrdeathih.dlt1.est
```

```
##                    term     estimate    std.error   statistic         df
## 1           (Intercept) 4.454744e-07   5.87158391 -2.49066118   47.65947
## 2              CIA_AMI1 6.234830e-07 742.08984533 -0.01925366 1950.79845
## 3              CIA_CHF1 2.409443e+00   0.42777181  2.05575877 1805.92415
## 4             CIA_CEVD1 3.439757e+00   0.49053207  2.51849147 1920.01826
## 5              CIA_DEM1 3.390202e-01   0.64948009 -1.66547904 1525.10538
## 6              CIA_CKD1 4.133494e+00   0.33991494  4.17493610 1865.39220
## 7               CIA_CA1 4.015608e+00   0.43251302  3.21421246 1945.58933
## 8           CIA_CA_MET1 7.666970e+00   0.55019373  3.70218954 1947.31443
## 9              PREOP_HB 9.848205e-01   0.03219815 -0.47505430 1218.00258
## 10            PREOP_WCC 1.054470e+00   0.01842578  2.87845726  217.99601
## 11             PREOP_NA 1.067855e+00   0.04191089  1.56646852   44.05635
## 12            SURG_SEV1 1.082587e+00   0.32649397  0.24304663 1854.95765
## 13            SURG_SEV2 2.055691e+00   0.38630295  1.86540656 1739.75005
## 14 SURG_SCHEDULED_TYPE1 6.701146e+00   0.38399653  4.95389497 1778.10114
## 15            PreopICU1 2.989256e+00   0.67199803  1.62950580 1940.02063
##         p.value        2.5 %      97.5 %
## 1  1.628928e-02 3.317146e-12  0.05982475
## 2  9.846407e-01 0.000000e+00         Inf
## 3  3.994925e-02 1.041244e+00  5.57546252
## 4  1.186657e-02 1.314385e+00  9.00187123
## 5  9.602260e-02 9.483051e-02  1.21200153
## 6  3.117966e-05 2.122235e+00  8.05084247
## 7  1.329450e-03 1.719358e+00  9.37856143
## 8  2.196916e-04 2.606194e+00 22.55489323
## 9  6.348334e-01 9.245336e-01  1.04903859
## 10 4.394004e-03 1.016863e+00  1.09346693
## 11 1.243944e-01 9.813651e-01  1.16196772
## 12 8.079961e-01 5.706490e-01  2.05379128
## 13 6.229265e-02 9.636232e-01  4.38539214
## 14 7.964510e-07 3.155498e+00 14.23082696
## 15 1.033683e-01 8.002033e-01 11.16673037
```

```
lrdeathih.dlt2.est
```

```
##                    term     estimate    std.error  statistic        df
## 1           (Intercept) 3.146213e-07   5.30222622 -2.8237000  211.6408
## 2              CIA_AMI1 6.109847e-07 741.34199144 -0.0193004 1950.7985
## 3              CIA_CHF1 2.417472e+00   0.42675148  2.0684695 1931.4922
## 4             CIA_CEVD1 3.402620e+00   0.49206764  2.4885722 1890.4792
## 5              CIA_DEM1 3.480592e-01   0.63794755 -1.6543407 1946.0882
## 6              CIA_CKD1 4.191575e+00   0.33949098  4.2212507 1941.1074
## 7               CIA_CA1 4.041315e+00   0.43431695  3.2155550 1927.1296
## 8           CIA_CA_MET1 7.805079e+00   0.55054551  3.7322521 1947.2710
## 9              PREOP_HB 9.840861e-01   0.03002309 -0.5343192  622.0301
## 10            PREOP_WCC 1.059925e+00   0.01775402  3.2780101 1649.0622
## 11             PREOP_NA 1.070142e+00   0.03775493  1.7955565  188.9897
## 12            SURG_SEV1 1.077826e+00   0.32557522  0.2301954 1947.8451
## 13            SURG_SEV2 2.063090e+00   0.38583036  1.8770033 1893.9670
## 14 SURG_SCHEDULED_TYPE1 6.672898e+00   0.38282514  4.9580188 1947.5099
## 15            PreopICU1 2.949931e+00   0.67349710  1.6062157 1942.9867
##         p.value        2.5 %      97.5 %
## 1  5.200545e-03 9.090089e-12  0.01088951
## 2  9.846034e-01 0.000000e+00         Inf
## 3  3.872870e-02 1.046845e+00  5.58265472
## 4  1.291121e-02 1.296273e+00  8.93162674
## 5  9.821957e-02 9.960778e-02  1.21622251
## 6  2.541650e-05 2.153881e+00  8.15704332
## 7  1.323485e-03 1.724245e+00  9.47209945
## 8  1.952183e-04 2.651311e+00 22.97703187
## 9  5.933116e-01 9.277427e-01  1.04385123
## 10 1.067167e-03 1.023650e+00  1.09748441
## 11 7.416267e-02 9.933382e-01  1.15288363
## 12 8.179641e-01 5.691755e-01  2.04103777
## 13 6.067125e-02 9.680299e-01  4.39691003
## 14 7.738643e-07 3.149564e+00 14.13769022
## 15 1.083890e-01 7.873588e-01 11.05225500
```

```
explanatory.cvd = c("CIA_AMI", "CIA_CHF", "CIA_CEVD", "CIA_DEM", "CIA_CKD"
                    , "CIA_CA", "CIA_CA_MET", "PREOP_HB", "PREOP_WCC"
                    , "PREOP_NA", "SURG_SEV", "SURG_SCHEDULED_TYPE", "PreopICU")

complete(train.imp.delta[[1]]) %>%
  or_plot("DEATH_INHOSPITAL", explanatory.cvd
          , glmfit = pool(lrdeathih.dlt0)
          , confint_type = "profile"
          , table_text_size = 3
          , title_text_size = 10
          , dependent_label = "Any complication: delta = 0")
```

Any complication: delta = 0: OR (95% CI, p-value)

CIA_AMI	0	-
	1	0.00 (0.00-Inf, p=0.985)
CIA_CHF	0	-
	1	2.58 (1.11-5.99, p=0.027)
CIA_CEVD	0	-
	1	3.53 (1.33-9.37, p=0.011)
CIA_DEM	0	-
	1	0.35 (0.10-1.24, p=0.104)
CIA_CKD	0	-
	1	3.54 (1.82-6.90, p<0.001)
CIA_CA	0	-
	1	4.18 (1.77-9.90, p=0.001)
CIA_CA_MET	0	-
	1	8.19 (2.78-24.10, p<0.001)
PREOP_HB	[4.3,18.2]	0.80 (0.68-0.94, p=0.006)
PREOP_WCC	[1.4,82.5]	1.07 (1.02-1.12, p=0.007)
PREOP_NA	[120.0,155.0]	1.07 (0.99-1.15, p=0.069)
SURG_SEV	0	-
	1	1.20 (0.63-2.29, p=0.582)
	2	2.75 (1.24-6.07, p=0.012)
SURG_SCHEDULED_TYPE	0	-
	1	5.04 (2.39-10.61, p<0.001)
PreopICU	0	-
	1	3.26 (0.84-12.63, p=0.088)

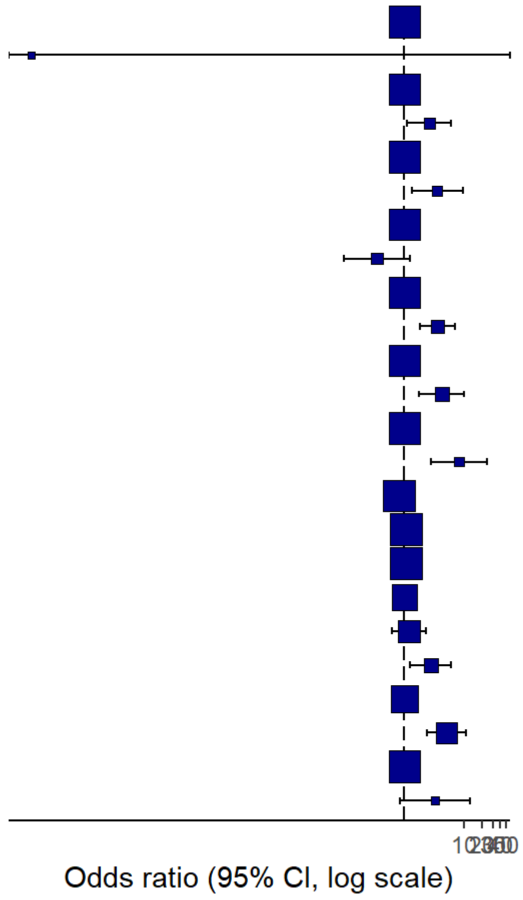


```
complete(train.imp.delta[[2]]) %>%
  or_plot("DEATH_INHOSPITAL", explanatory.cvd
          , glmfit = pool(lrdeathih.dlt1)
          , confint_type = "profile"
          , table_text_size = 3
          , title_text_size = 10
          , dependent_label = "Any complication: delta = m + 1*SD")
```

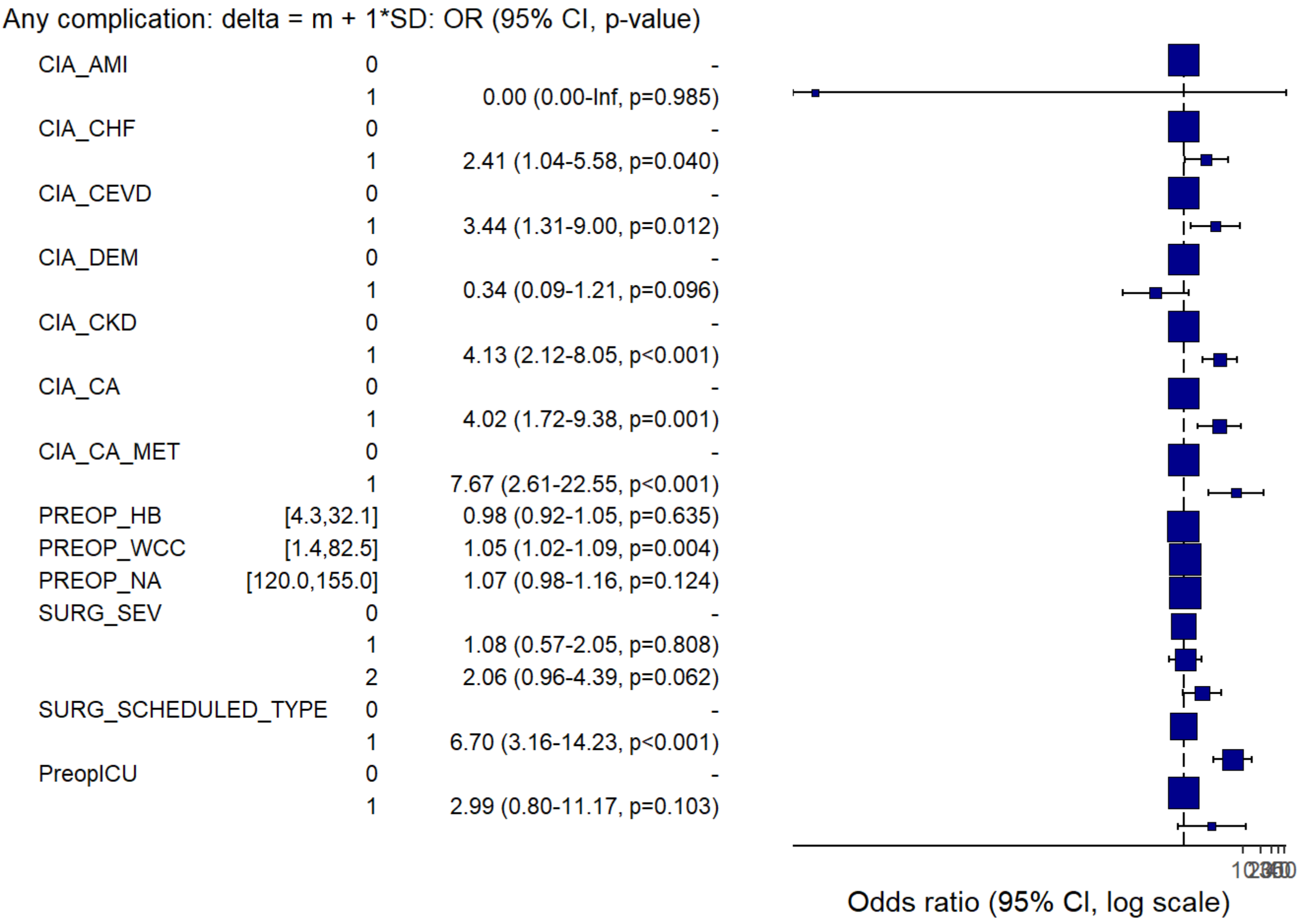


```
complete(train.imp.delta[[3]]) %>%
  or_plot("DEATH_INHOSPITAL", explanatory.cvd
          , glmfit = pool(lrdeathih.dlt2)
          , confint_type = "profile"
          , table_text_size = 3
          , title_text_size = 10
          , dependent_label = "Any complication: delta = m + 2*SD")
```

Any complication: delta = m + 2*SD: OR (95% CI, p-value)

CIA_AMI	0	-
	1	0.00 (0.00-Inf, p=0.985)
CIA_CHF	0	-
	1	2.42 (1.05-5.58, p=0.039)
CIA_CEVD	0	-
	1	3.40 (1.30-8.93, p=0.013)
CIA_DEM	0	-
	1	0.35 (0.10-1.22, p=0.098)
CIA_CKD	0	-
	1	4.19 (2.15-8.16, p<0.001)
CIA_CA	0	-
	1	4.04 (1.72-9.47, p=0.001)
CIA_CA_MET	0	-
	1	7.81 (2.65-22.98, p<0.001)
PREOP_HB	[4.3,34.1]	0.98 (0.93-1.04, p=0.593)
PREOP_WCC	[1.4,82.5]	1.06 (1.02-1.10, p=0.001)
PREOP_NA	[120.0,155.0]	1.07 (0.99-1.15, p=0.074)
SURG_SEV	0	-
	1	1.08 (0.57-2.04, p=0.818)
	2	2.06 (0.97-4.40, p=0.061)
SURG_SCHEDULED_TYPE	0	-
	1	6.67 (3.15-14.14, p<0.001)
PreopICU	0	-
	1	2.95 (0.79-11.05, p=0.108)

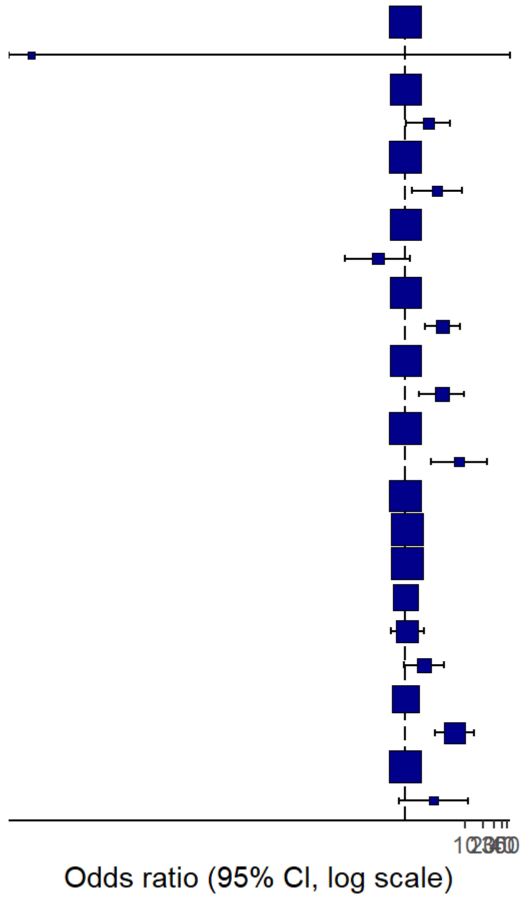

8.4 Model testing

We performed the model testing using the testing dataset for original and pooled imputed models.
Model testing has three components.
First, model discrimination ability using ROC curve analysis.
Second, regarding the threshold estimated from the training dataset, ROC analysis on testing dataset. This process assess the ability of the clinical dichromotous decision making such as “Yes” or “No” risk of complications.
Third, calibrations measurement as usual.

8.4.1 Original model testing

```
# Predicted probability and classification
lrdeathih.org.test.pred <- predict(lrdeathih.org.final
                                , newdata = test.org.na
                                , type = "response")
lrdeathih.org.test.class <- ifelse(lrdeathih.org.test.pred>=0.5, 1, 0)

# Confusion matrix
cl_table.lrdeathih.org.test <- table (test.org.na$DEATH_INHOSPITAL, lrdeathih.org.test.class)
rownames(cl_table.lrdeathih.org.test) <- c("Alive", "Death in-hospital")
colnames(cl_table.lrdeathih.org.test) <- c("Alive", "Death in-hospital")
addmargins(cl_table.lrdeathih.org.test)
```

```
##                      lrdeathih.org.test.class
##                       Alive Death in-hospital Sum
##   Alive                 400                 0 400
##   Death in-hospital      15                 1  16
##   Sum                   415                 1 416
```

```r
# Model accuracy (classification accuracy)
mean((test.org.na$DEATH_INHOSPITAL == lrdeathih.org.test.class))
```

```
## [1] 0.9639423
```

```r
# Discriminability
roc.lrdeathih.org.test <- roc(test.org.na$DEATH_INHOSPITAL
                          ,lrdeathih.org.test.pred
                          , add = TRUE)
```

```
## Setting levels: control = 0, case = 1
```

```
## Setting direction: controls < cases
```

```r
roc.lrdeathih.org.test$auc
```

```
## Area under the curve: 0.8086
```

```r
ci(roc.lrdeathih.org.test)
```

```
## 95% CI: 0.6762-0.941 (DeLong)
```

```r
plot.roc(roc.lrdeathih.org.test
         , print.auc = TRUE
         , print.thres = FALSE
         , auc.polygon = TRUE
)
```

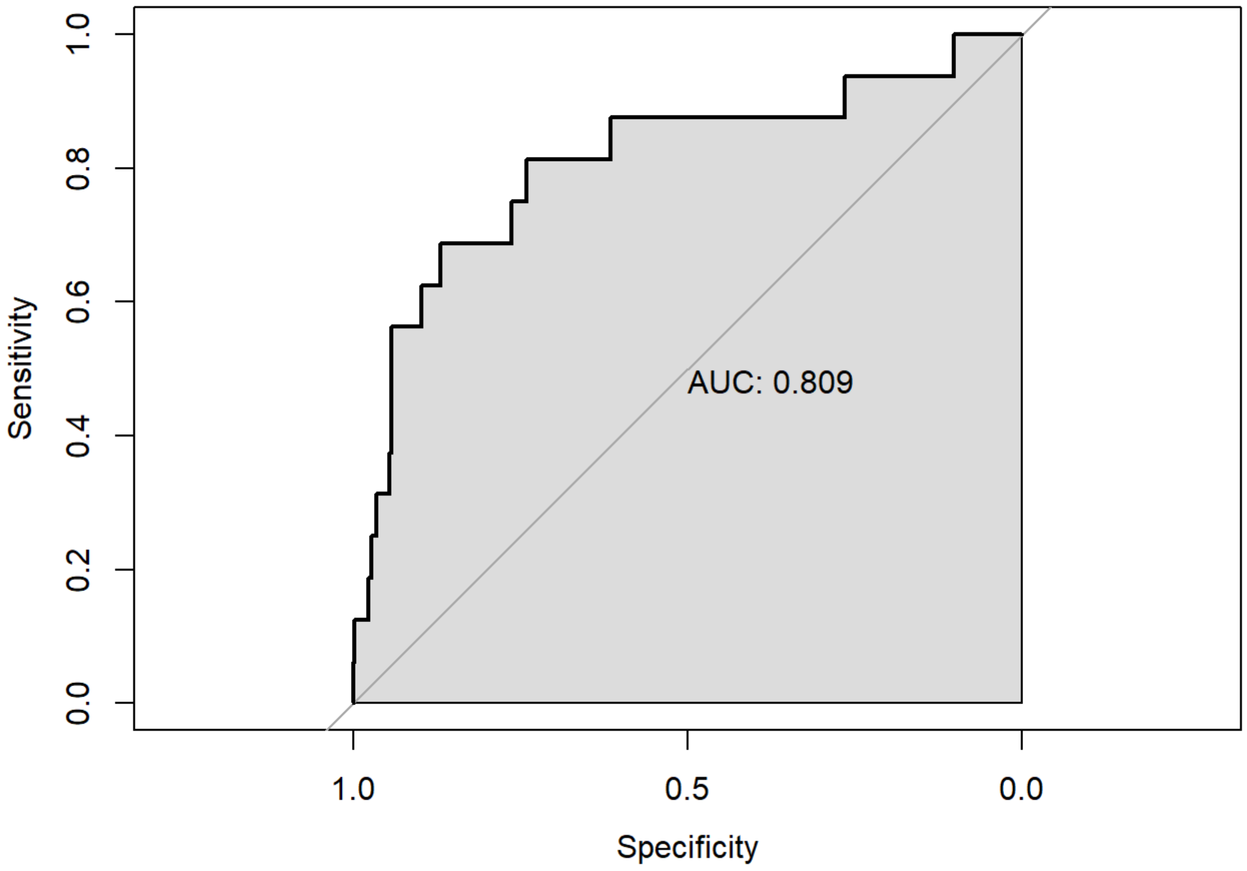


```
pROC::coords(roc.lrdeathih.org.test,
             "best",
             transpose = TRUE,
             best.method = "youden")
```

```
##   threshold specificity sensitivity
##  0.04745798  0.87000000  0.68750000
```

```
#Threshold discrimination ability

lrdeathih.org.test.pred.dcs <- ifelse(lrdeathih.org.test.pred > 0.02957133, 1, 0)

roc.lrdeathih.org.test.dcs <- roc(test.org.na$DEATH_INHOSPITAL
                              ,lrdeathih.org.test.pred.dcs
                              , add = TRUE)
```

```
## Setting levels: control = 0, case = 1
```

```
## Setting direction: controls < cases
```

```
roc.lrdeathih.org.test.dcs$auc
```

```
## Area under the curve: 0.755
```

```
ci(roc.lrdeathih.org.test.dcs)
```

```
## 95% CI: 0.6434-0.8666 (DeLong)
```

```
plot.roc(roc.lrdeathih.org.test.dcs
         , print.auc = TRUE
         , print.thres = FALSE
         , auc.polygon = TRUE
)
```

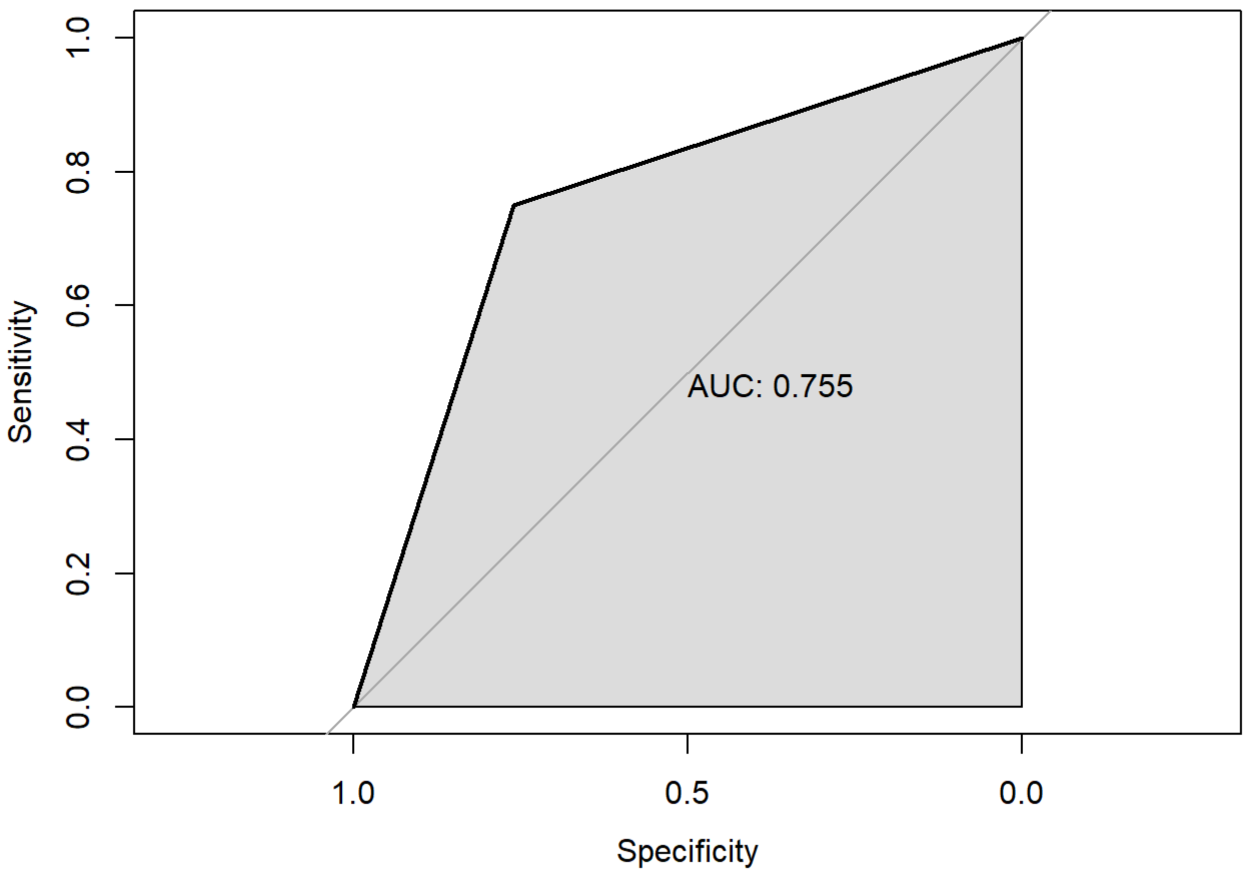


```
pROC::coords(roc.lrdeathih.org.test.dcs,
             "best",
             transpose = TRUE,
             best.method = "youden")
```

```
##   threshold specificity sensitivity
##        0.50        0.76        0.75
```

8.4.2 Imputed model Testing

8.4.2.1 Testing with the imputted result.

```
# Predicted probability and classification: pooled model
lrdeathih.imp.test.pred <- as.data.frame(predict(lrdeathih.imp, test.org.na, type = "response"))
lrdeathih.imp.test.class <- ifelse(lrdeathih.imp.test.pred>=0.5, 1, 0)

# Confusion matrix
cl_table.lrdeathih.imp.test <- table (test.org.na$DEATH_INHOSPITAL, lrdeathih.imp.test.class)
rownames(cl_table.lrdeathih.imp.test) <- c("Alive", "Death in-hospital")
colnames(cl_table.lrdeathih.imp.test) <- c("Alive", "Death in-hospital")
addmargins(cl_table.lrdeathih.imp.test)
```

```
##                    lrdeathih.imp.test.class
##                     Alive Death in-hospital Sum
##   Alive               400                 0 400
##   Death in-hospital    15                 1  16
##   Sum                 415                 1 416
```

```
# Sensitivity
round(cl_table.lrdeathih.imp.test[2,2]/(cl_table.lrdeathih.imp.test[2,1] + cl_table.lrdeathih.imp.te
st[2,2]),3)
```

```
## [1] 0.062
```

```
# Specificity
round(cl_table.lrdeathih.imp.test[1,1]/(cl_table.lrdeathih.imp.test[1,1] + cl_table.lrdeathih.imp.te
st[1,2]),3)
```

```
## [1] 1
```

```
# Positive predictive rate
round(cl_table.lrdeathih.imp.test[2,2]/(cl_table.lrdeathih.imp.test[1,2] + cl_table.lrdeathih.imp.te
st[2,2]),3)
```

```
## [1] 1
```

```
# Negative predictive rate
round(cl_table.lrdeathih.imp.test[1,1]/(cl_table.lrdeathih.imp.test[1,1] + cl_table.lrdeathih.imp.te
st[2,1]),3)
```

```
## [1] 0.964
```

```
#model accuracy (classification accuracy)
mean((test.org.na$DEATH_INHOSPITAL == lrdeathih.imp.test.class))
```

```
## [1] 0.9639423
```

```
#Discriminability

roc.lrdeathih.imp.test <- roc(test.org.na$DEATH_INHOSPITAL
                          , lrdeathih.imp.test.pred[,1]
                          , add = TRUE)
```

```
## Setting levels: control = 0, case = 1
```

```
## Setting direction: controls < cases
```

```
roc.lrdeathih.imp.test$auc
```

```
## Area under the curve: 0.7684
```

```
ci(roc.lrdeathih.imp.test)
```

```
## 95% CI: 0.612-0.9249 (DeLong)
```

```
pROC::coords(roc.lrdeathih.imp.test
             , "best"
             , transpose = TRUE
             , best.method="youden"
)
```

```
##                   [,1]       [,2]
## threshold   0.03993089 0.05897317
## specificity 0.81500000 0.87750000
## sensitivity 0.68750000 0.62500000
```

```
plot.roc(roc.lrdeathih.imp.test
         , print.auc = TRUE
         , print.thres = FALSE
         , auc.polygon = TRUE
)
```

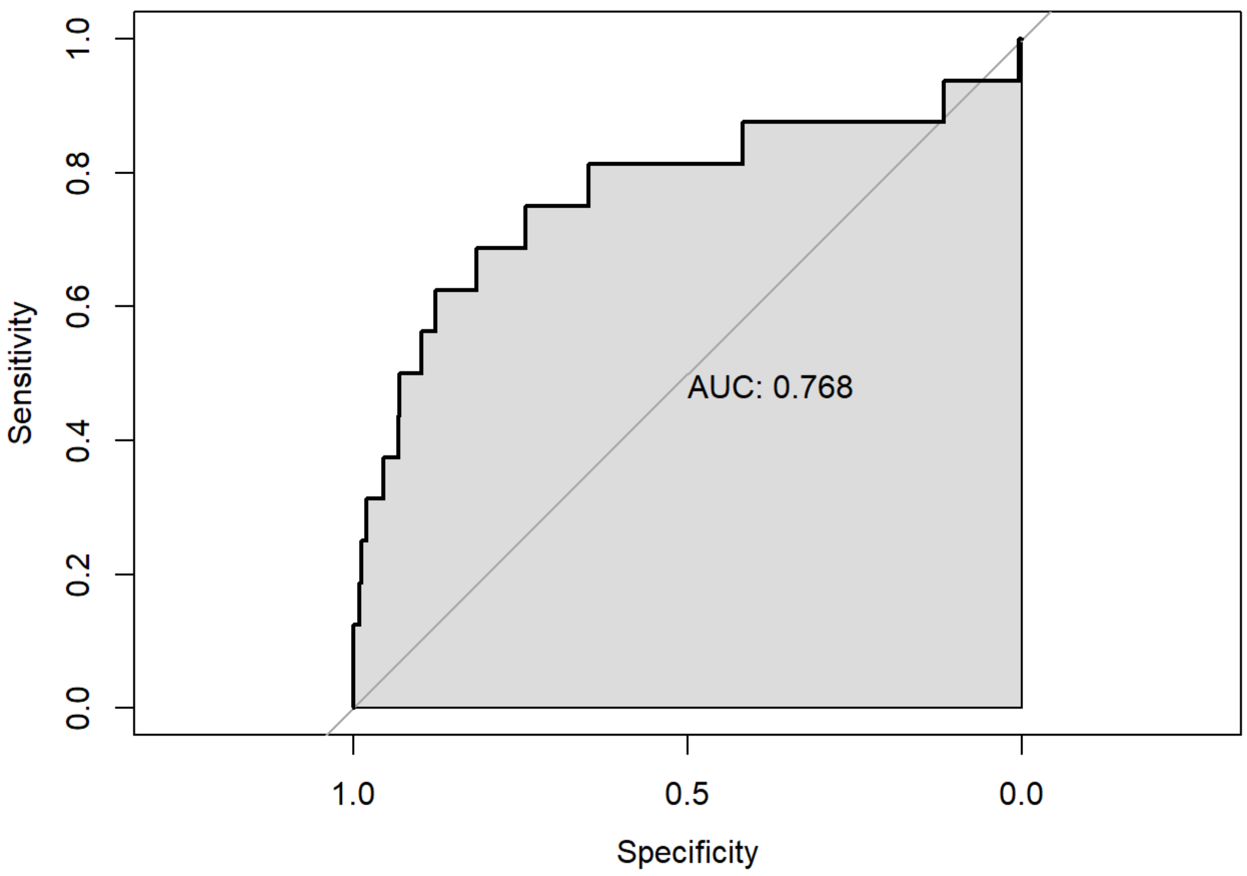


```
# Threshold discrimination ability

lrdeathih.imp.test.pred.dcs <- ifelse(lrdeathih.imp.test.pred[,1] > 0.02957133, 1, 0)

roc.lrdeathih.imp.test.dcs <- roc(test.org.na$DEATH_INHOSPITAL
                                  ,lrdeathih.imp.test.pred.dcs
                                  , add = TRUE)
```

```
## Setting levels: control = 0, case = 1
## Setting direction: controls < cases
```

```
roc.lrdeathih.imp.test.dcs$auc
```

```
## Area under the curve: 0.7225
```

```
ci(roc.lrdeathih.imp.test.dcs)
```

```
## 95% CI: 0.6033-0.8417 (DeLong)
```

```
plot.roc(roc.lrdeathih.imp.test.dcs
         , print.auc = TRUE
         , print.thres = FALSE
         , auc.polygon = TRUE
)
```

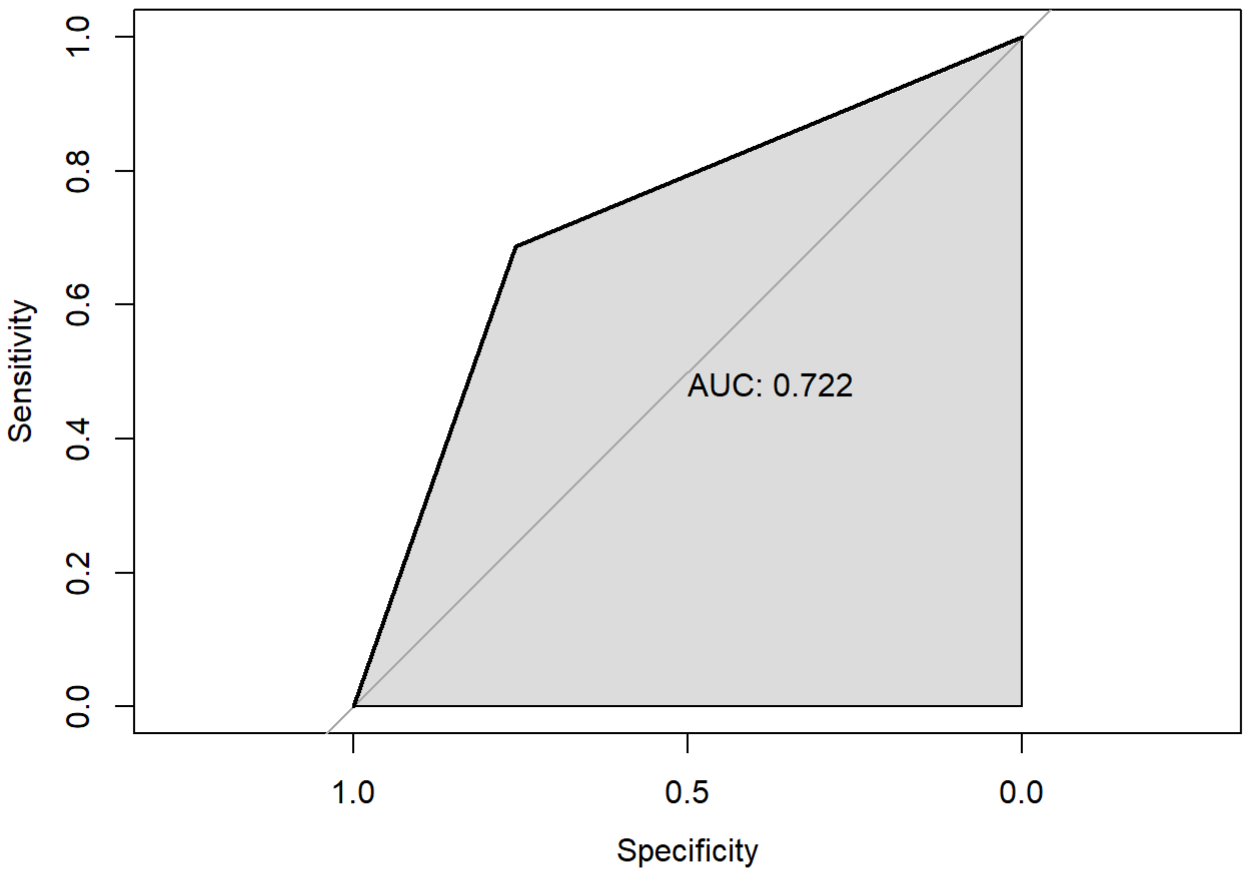


```
pROC::coords(roc.lrdeathih.imp.test.dcs,
             "best",
             transpose = TRUE,
             best.method = "youden")
```

```
##   threshold specificity sensitivity
##      0.5000      0.7575      0.6875
```

```
# Calibrations: on the testing dataset
cal.lrdeathih.imp.test <- val.prob(lrdeathih.imp.test.pred[,1]
                                , test.org.na$DEATH_INHOSPITAL
                                , pl = TRUE)
```

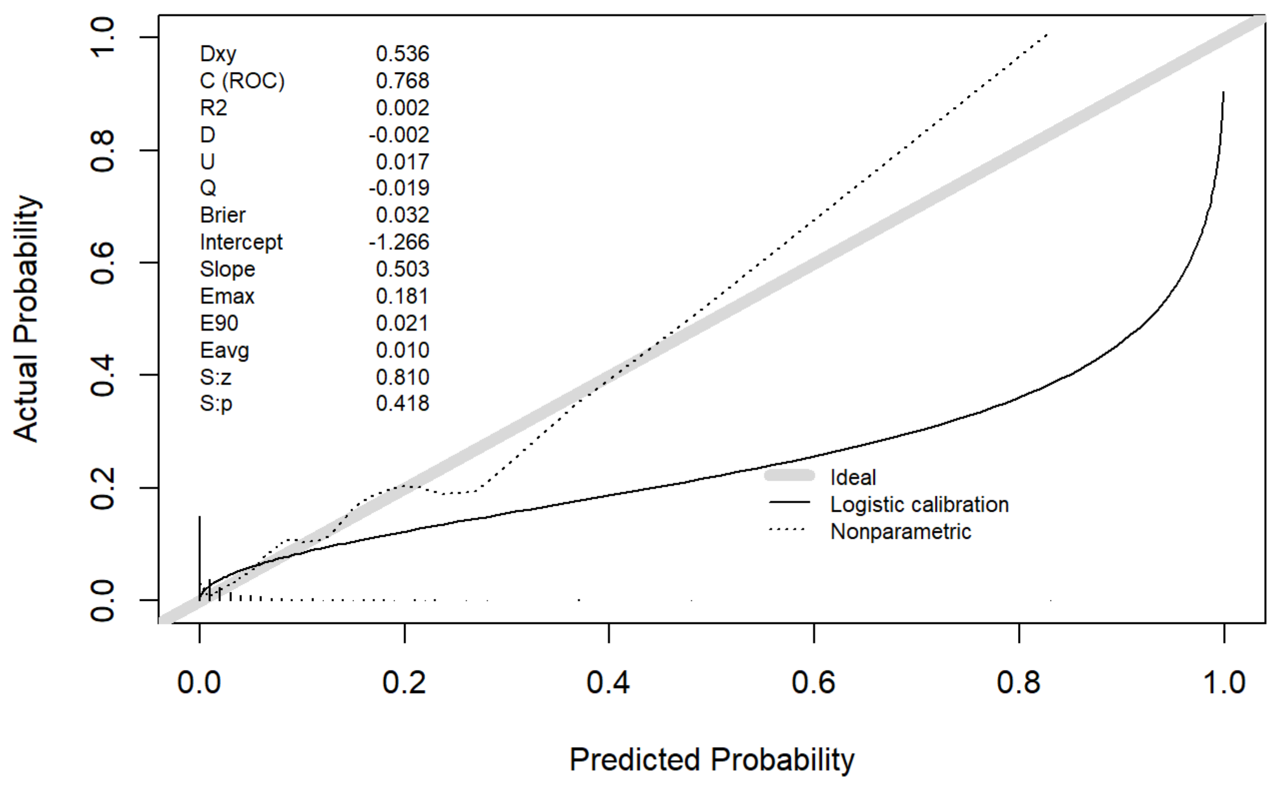


```
cal.lrdeathih.imp.test
```

```
##         Dxy      C (ROC)          R2            D     D:Chi-sq          D:p
##  0.535937500  0.767968750  0.002290195 -0.001766445  0.265159028           NA
##           U     U:Chi-sq          U:p            Q        Brier    Intercept
##  0.017014658  9.078097543  0.010683564 -0.018781102  0.032055593 -1.265828191
##       Slope         Emax          E90         Eavg          S:z          S:p
##  0.503157684  0.181197649  0.020854001  0.010308168  0.810129249  0.417865895
```

```
hosmer_lemeshow(test.org.na$DEATH_INHOSPITAL, lrdeathih.imp.test.pred[,1], 10, 'C')
```

```
## PVALUE 0.3916629
## stat 8.440519
```

```
## [1] 0.3916629
```

```
hosmer_lemeshow(test.org.na$DEATH_INHOSPITAL, lrdeathih.imp.test.pred[,1], 10, 'H')
```

```
## PVALUE 0.8136581
## stat 4.457619
```

```
## [1] 0.8136581
```

Calibration interpretations & decision of re-calibration requirement

Brier score: 0.032 <<1
Spiegelhalter Z-test P = 0.147: Fail to reject the null hypothesis
Hosmer-Lemeshow C & H test P values = 0.391 & 0.814 : appropriate for C & H.
Calibration curve: not acceptible.

8.5 Recalibrations using testing dataset

Recalibration: Isotonic regression model

```
# predicted probability testing dataset with pooled logistic regression
lrdeathih.imp.test.pred.prob <- predict(lrdeathih.imp,
                                    test.org.na[,-length(test.org.na)],
                                    type = "response")
lrdeathih.imp.iso.data = cbind(y=test.org.na$DEATH_INHOSPITAL
                           , yhat=lrdeathih.imp.test.pred.prob)
lrdeathih.imp.iso.data.order = lrdeathih.imp.iso.data[order(lrdeathih.imp.iso.data[,2]),]

# create calibration model: isodeathih.imp
isodeathih.imp.wostepfunc <- isoreg(lrdeathih.imp.iso.data.order[,2], lrdeathih.imp.iso.data.order[,
1])
isodeathih.imp.stepf_data = cbind(isodeathih.imp.wostepfunc$x, isodeathih.imp.wostepfunc$yf)
isodeathih.imp = stepfun(isodeathih.imp.stepf_data[,1], c(0,isodeathih.imp.stepf_data[,2]))

# Predicted probability of isotonic regression on testing dataset. Imputed model

isodeathih.imp.pred <- isodeathih.imp(as.data.frame(lrdeathih.imp.test.pred.prob)[,1])
```

Recalibration: Platt scailing

```
pltdeathih.imp <- glm(y~yhat, as.data.frame(lrdeathih.imp.iso.data), family=binomial)
ygrid_norm = as.data.frame(lrdeathih.imp.test.pred)
colnames(lrdeathih.imp.test.pred) <- c("yhat")



# Predicted probability of platt scailing
pltdeathih.imp.pred <- as.data.frame(predict(pltdeathih.imp, lrdeathih.imp.test.pred, type = "respon
se" ))
```

8.5.1 Discriminations & Calibration measurements of recalibrated models

8.5.1.1 Discrimination

```
# Discrimination ability: Isoregression model

roc.isodeathih.test.imp <- roc(test.org.na$DEATH_INHOSPITAL
                           , isodeathih.imp.pred
                           , add = TRUE)
```

```
## Setting levels: control = 0, case = 1
```

```
## Setting direction: controls < cases
```

```
roc.isodeathih.test.imp$auc
```

```
## Area under the curve: 0.8089
```

```
ci(roc.isodeathih.test.imp)
```

```
## 95% CI: 0.6834-0.9344 (DeLong)
```

```
pROC::coords(roc.isodeathih.test.imp
             , "best"
             , transpose = TRUE
             , best.method="youden"
)
```

```
##                   [,1]       [,2]
## threshold   0.03589744 0.06270903
## specificity 0.81500000 0.87750000
## sensitivity 0.68750000 0.62500000
```

```
plot.roc(roc.isodeathih.test.imp
         , print.auc = TRUE
         , print.thres = FALSE
         , auc.polygon = TRUE
)
```

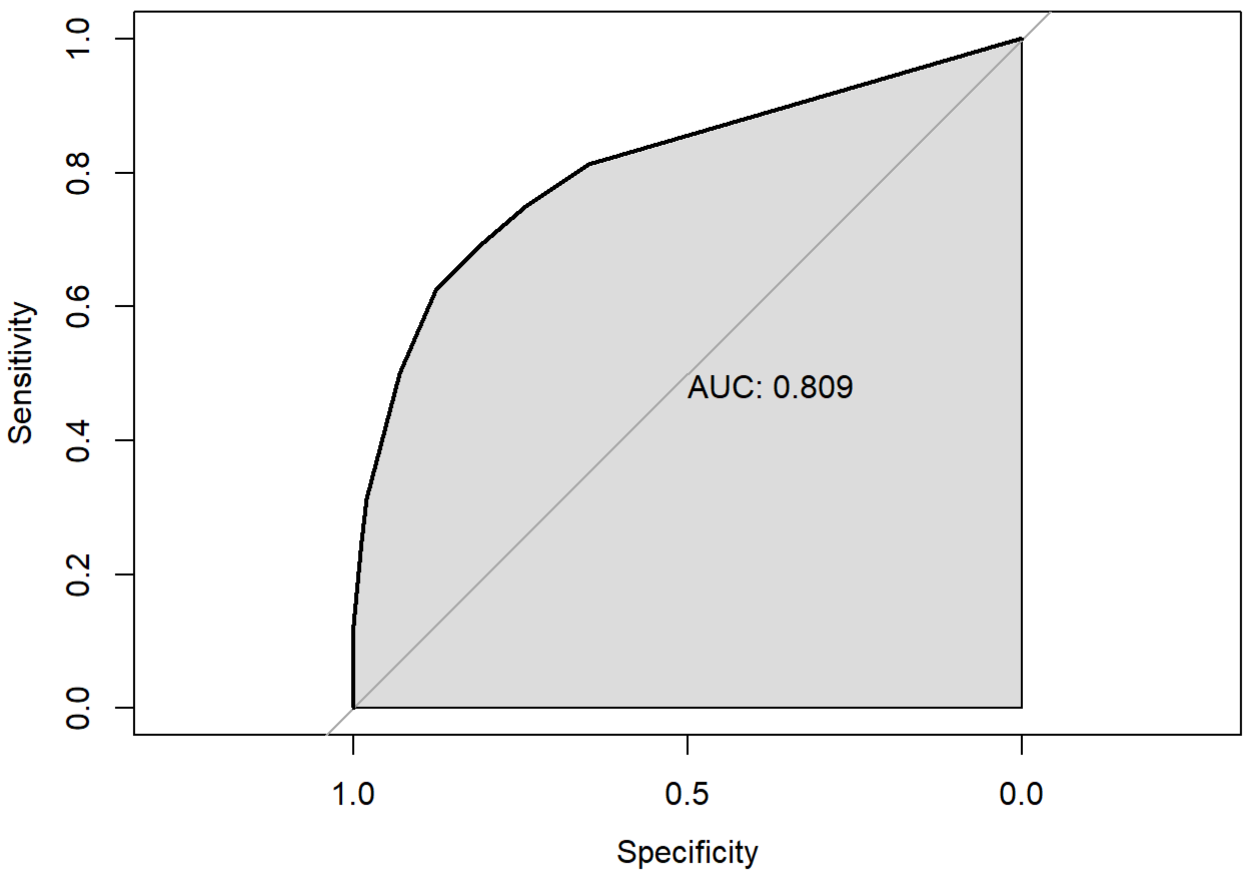

```
# Discrimination ability: Platt Scaling model
roc.pltdeathih.test.imp <- roc(test.org.na$DEATH_INHOSPITAL
                           , pltdeathih.imp.pred[,1]
                           , add = TRUE)
```

```
## Setting levels: control = 0, case = 1
## Setting direction: controls < cases
```

```
roc.pltdeathih.test.imp$auc
```

```
## Area under the curve: 0.7684
```

```
ci(roc.pltdeathih.test.imp)
```

```
## 95% CI: 0.612-0.9249 (DeLong)
```

```
pROC::coords(roc.pltdeathih.test.imp
             , "best"
             , transpose = TRUE
             , best.method="youden"
)
```

```
##                   [,1]       [,2]
## threshold   0.03105226 0.03783482
## specificity 0.81500000 0.87750000
## sensitivity 0.68750000 0.62500000
```

```
plot.roc(roc.pltdeathih.test.imp
         , print.auc = TRUE
         , print.thres = FALSE
         , auc.polygon = TRUE
)
```

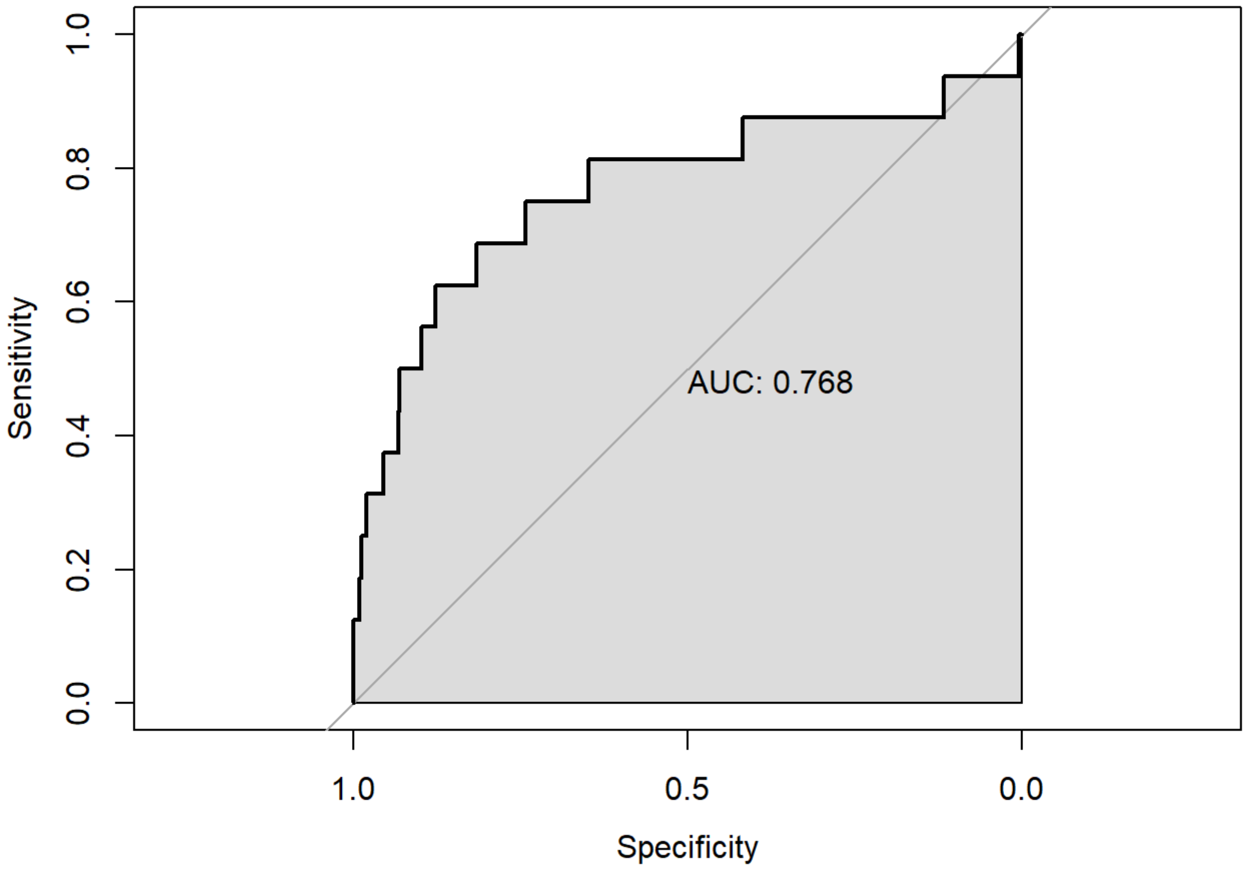

8.5.2 Threshold discrimination ability: Treshold from lrmodel with training data

```
# Isoregression model

isodeathih.imp.test.pred.dcs <- ifelse(isodeathih.imp.pred > 0.02957133, 1, 0)

roc.isodeathih.imp.test.dcs <- roc(test.org.na$DEATH_INHOSPITAL
                               ,isodeathih.imp.test.pred.dcs
                               , add = TRUE)
```

```
## Setting levels: control = 0, case = 1
```

```
## Setting direction: controls < cases
```

```
roc.isodeathih.imp.test.dcs$auc
```

```
## Area under the curve: 0.7463
```

```
ci(roc.isodeathih.imp.test.dcs)
```

```
## 95% CI: 0.6346-0.8579 (DeLong)
```

```
plot.roc(roc.isodeathih.imp.test.dcs
         , print.auc = TRUE
         , print.thres = FALSE
         , auc.polygon = TRUE
)
```

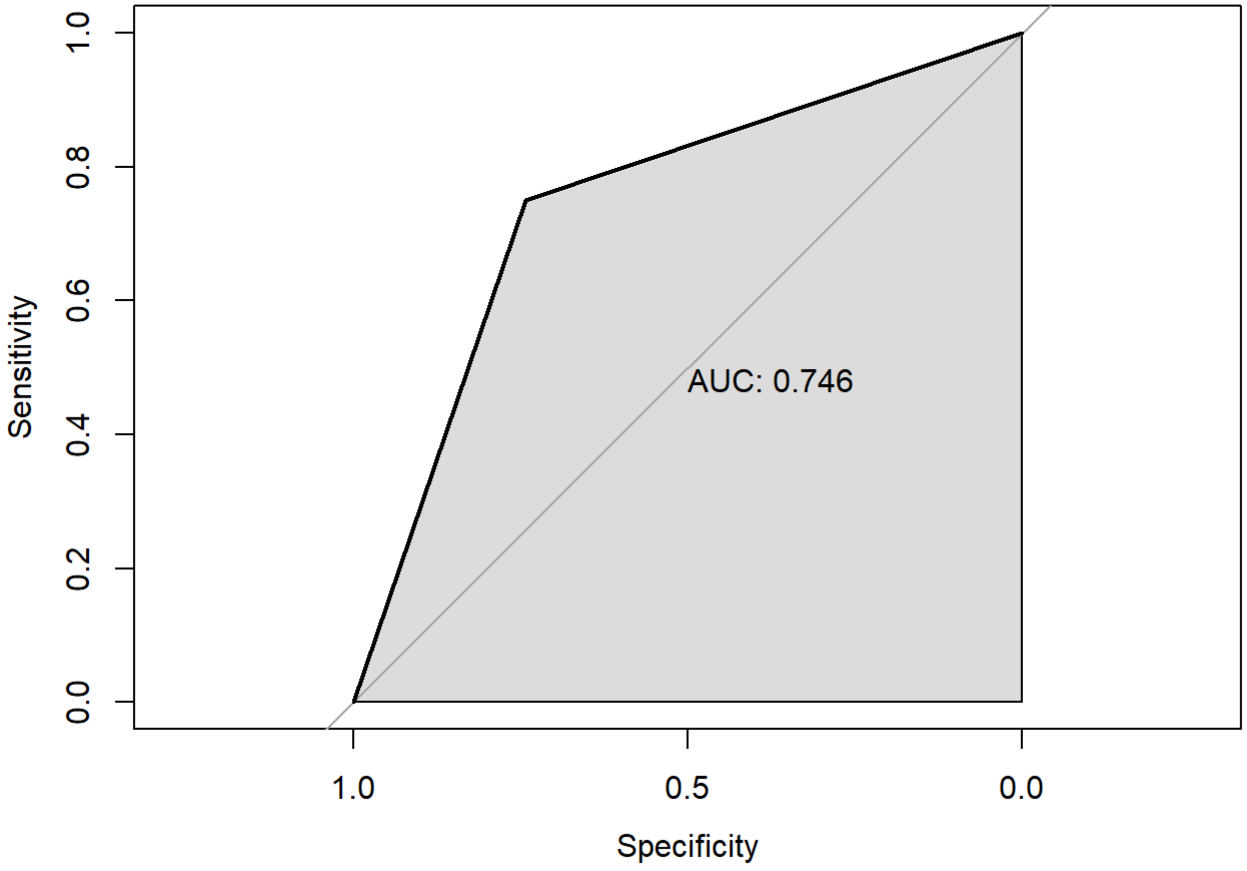


```
pROC::coords(roc.isodeathih.imp.test.dcs,
             "best",
             transpose = TRUE,
             best.method = "youden")
```

```
##   threshold specificity sensitivity
##      0.5000      0.7425      0.7500
```

```
# Platt Scailing model


pltdeathih.imp.test.pred.dcs <- ifelse(pltdeathih.imp.pred > 0.02957133, 1, 0)

roc.pltdeathih.imp.test.dcs <- roc(test.org.na$DEATH_INHOSPITAL
                                   ,pltdeathih.imp.test.pred.dcs
                                   , add = TRUE)
```

```
## Setting levels: control = 0, case = 1
## Setting direction: controls < cases
```

```
roc.pltdeathih.imp.test.dcs$auc
```

```
## Area under the curve: 0.74
```

```
ci(roc.pltdeathih.imp.test.dcs)
```

```
## 95% CI: 0.621-0.859 (DeLong)
```

```
plot.roc(roc.pltdeathih.imp.test.dcs
         , print.auc = TRUE
         , print.thres = FALSE
         , auc.polygon = TRUE
)
```

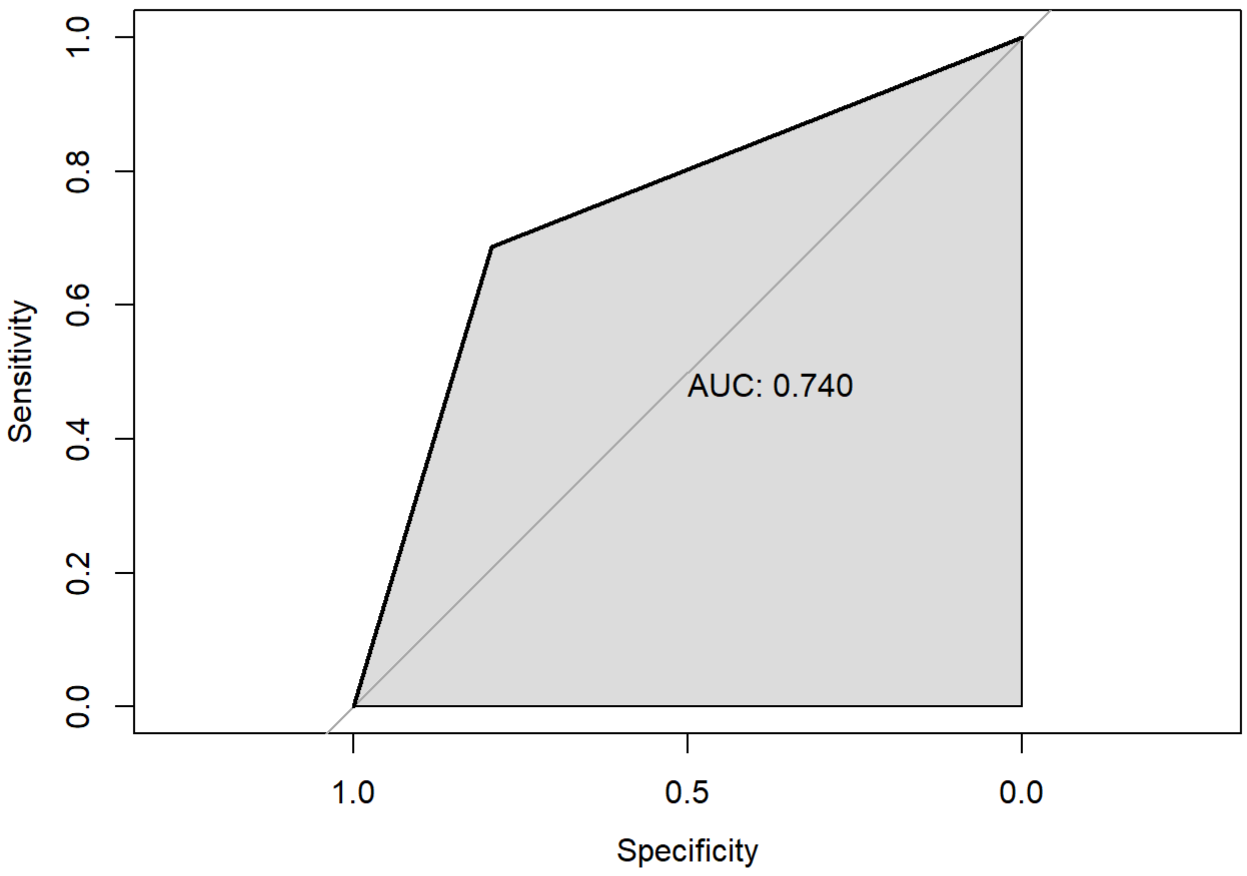


```
pROC::coords(roc.pltdeathih.imp.test.dcs,
             "best",
             transpose = TRUE,
             best.method = "youden")
```

```
##   threshold specificity sensitivity
##      0.5000      0.7925      0.6875
```

8.5.3 Calibrations of recalibrated models

```
cal.isodeathih.imp.test <- val.prob(isodeathih.imp.pred
                               , test.org.na$DEATH_INHOSPITAL
                               , pl = FALSE)


cal.pltdeathih.imp.test <- val.prob(pltdeathih.imp.pred[,1]
                               , test.org.na$DEATH_INHOSPITAL
                               , pl = FALSE)


cbind(cal.lrdeathih.imp.test, cal.isodeathih.imp.test, cal.pltdeathih.imp.test)
```

```
##           cal.lrdeathih.imp.test cal.isodeathih.imp.test
## Dxy                  0.535937500            5.610194e-01
## C (ROC)              0.767968750            7.805097e-01
## R2                   0.002290195            1.724435e-01
## D                   -0.001766445            4.284604e-02
## D:Chi-sq             0.265159028            1.865257e+01
## D:p                           NA                      NA
## U                    0.017014658           -4.854369e-03
## U:Chi-sq             9.078097543           -2.842171e-13
## U:p                  0.010683564            1.000000e+00
## Q                   -0.018781102            4.770041e-02
## Brier                0.032055593            3.000313e-02
## Intercept           -1.265828191           -2.540506e-10
## Slope                0.503157684            1.000000e+00
## Emax                 0.181197649            8.206366e-06
## E90                  0.020854001            8.206366e-06
## Eavg                 0.010308168            5.128979e-06
## S:z                  0.810129249            1.601537e-16
## S:p                  0.417865895            1.000000e+00
##           cal.pltdeathih.imp.test
## Dxy                   5.290625e-01
## C (ROC)               7.645312e-01
## R2                    1.853847e-01
## D                     5.055333e-02
## D:Chi-sq              2.203019e+01
## D:p                             NA
## U                    -4.807692e-03
## U:Chi-sq              2.842171e-14
## U:p                   1.000000e+00
## Q                     5.536102e-02
## Brier                 3.253849e-02
## Intercept             7.725188e-11
## Slope                 1.000000e+00
## Emax                  2.335438e-01
## E90                   2.764830e-02
## Eavg                  1.256022e-02
## S:z                   1.697792e-01
## S:p                   8.651838e-01
```

```
hosmer_lemeshow(test.org.na$DEATH_INHOSPITAL, lrdeathih.imp.test.pred[,1], 10, 'C')
```

```
## PVALUE 0.3916629
## stat 8.440519
```

```
## [1] 0.3916629
```

```
hosmer_lemeshow(test.org.na$DEATH_INHOSPITAL, isodeathih.imp.pred, 10, 'C')
```

```
## PVALUE 0.8088363
## stat 4.505962
```

```
## [1] 0.8088363
```

```
hosmer_lemeshow(test.org.na$DEATH_INHOSPITAL, pltdeathih.imp.pred[,1], 10, 'C')
```

```
## PVALUE 0.5675117
## stat 6.716449
```

```
## [1] 0.5675117
```

```
hosmer_lemeshow(test.org.na$DEATH_INHOSPITAL, lrdeathih.imp.test.pred[,1], 10, 'H')
```

```
## PVALUE 0.8136581
## stat 4.457619
```

```
## [1] 0.8136581
```

```
hosmer_lemeshow(test.org.na$DEATH_INHOSPITAL, isodeathih.imp.pred, 10, 'H')
```

```
## PVALUE 1
## stat 0
```

```
## [1] 1
```

```
hosmer_lemeshow(test.org.na$DEATH_INHOSPITAL, pltdeathih.imp.pred[,1], 10, 'H')
```

```
## PVALUE 0.8714094
## stat 3.838306
```

```
## [1] 0.8714094
```

```
CalibrationCurves::val.prob.ci.2(lrdeathih.imp.test.pred[,1], test.org.na$DEATH_INHOSPITAL
                                 , lty.smooth = 2
                                 , CL.smooth = FALSE
                                 , col.ideal = "black")
```

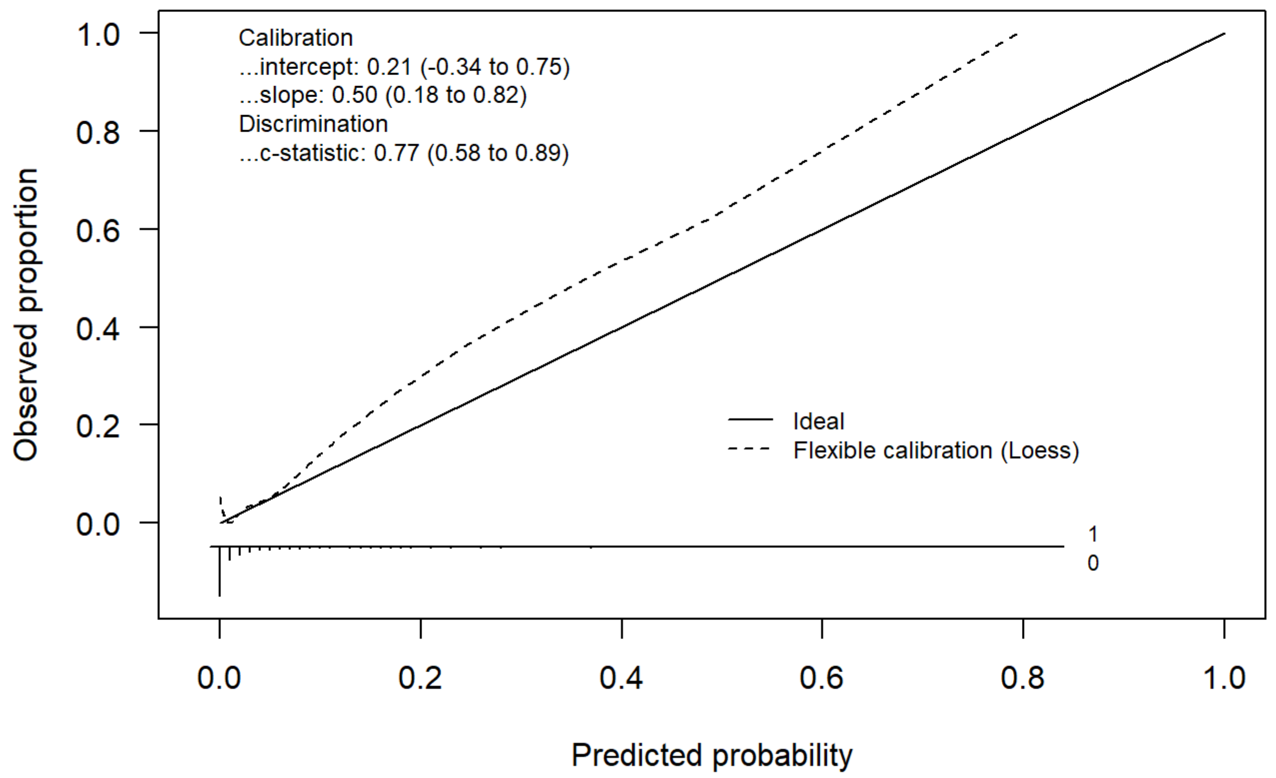


```
## Call:
## CalibrationCurves::val.prob.ci.2(p = lrdeathih.imp.test.pred[,
##     1], y = test.org.na$DEATH_INHOSPITAL, CL.smooth = FALSE,
##     lty.smooth = 2, col.ideal = "black")
##
## A 95% confidence interval is given for the calibration intercept, calibration slope and c-statist
ic.
##
##          Dxy       C (ROC)           R2             D      D:Chi-sq           D:p
##  0.536093750  0.768046875  0.079824227  0.020055905  9.343256571  0.002238075
##            U      U:Chi-sq          U:p             Q         Brier     Intercept
##  0.017014658  9.078097543  0.010683564  0.003041248  0.032055593  0.207008415
##        Slope          Emax Brier scaled          Eavg           ECI
##  0.503157684  0.447224324  0.133216769  0.016132474  0.088303727
```

```
CalibrationCurves::val.prob.ci.2(isodeathih.imp.pred, test.org.na$DEATH_INHOSPITAL
                                 , lty.smooth = 2
                                 , CL.smooth = FALSE
                                 , col.ideal = "black")
```

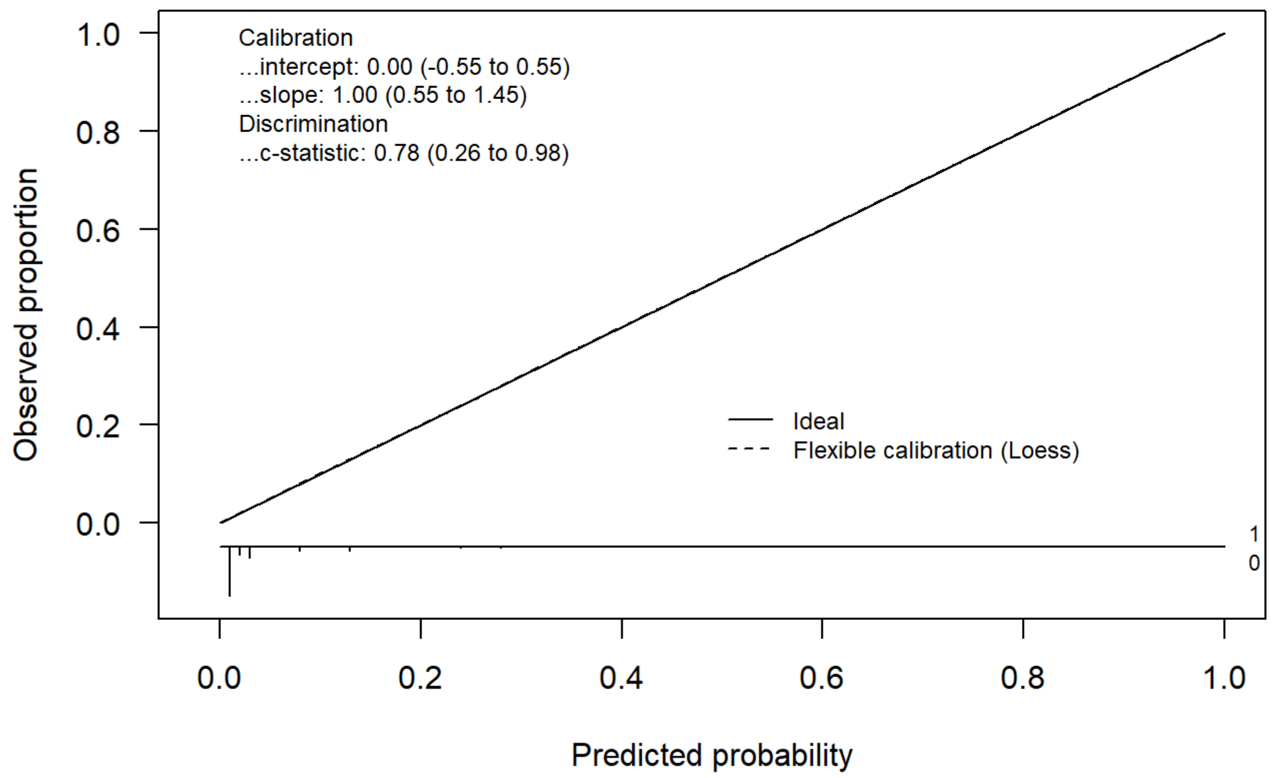


```
## Call:
## CalibrationCurves::val.prob.ci.2(p = isodeathih.imp.pred, y = test.org.na$DEATH_INHOSPITAL,
##     CL.smooth = FALSE, lty.smooth = 2, col.ideal = "black")
##
## A 95% confidence interval is given for the calibration intercept, calibration slope and c-statist
ic.
##
##           Dxy       C (ROC)            R2             D      D:Chi-sq
##  5.610194e-01  7.805097e-01  1.724435e-01  4.284604e-02  1.865257e+01
##           D:p             U      U:Chi-sq           U:p             Q
##  1.568357e-05 -4.854369e-03 -5.684342e-14  1.000000e+00  4.770041e-02
##         Brier     Intercept         Slope          Emax  Brier scaled
##  3.029442e-02  5.994586e-12  1.000000e+00  8.143253e-11  1.808388e-01
##          Eavg           ECI
##  1.176401e-16  1.257391e-29
```

```
CalibrationCurves::val.prob.ci.2(pltdeathih.imp.pred[,1], test.org.na$DEATH_INHOSPITAL
                                 , lty.smooth = 2
                                 , CL.smooth = FALSE
                                 , col.ideal = "black")
```

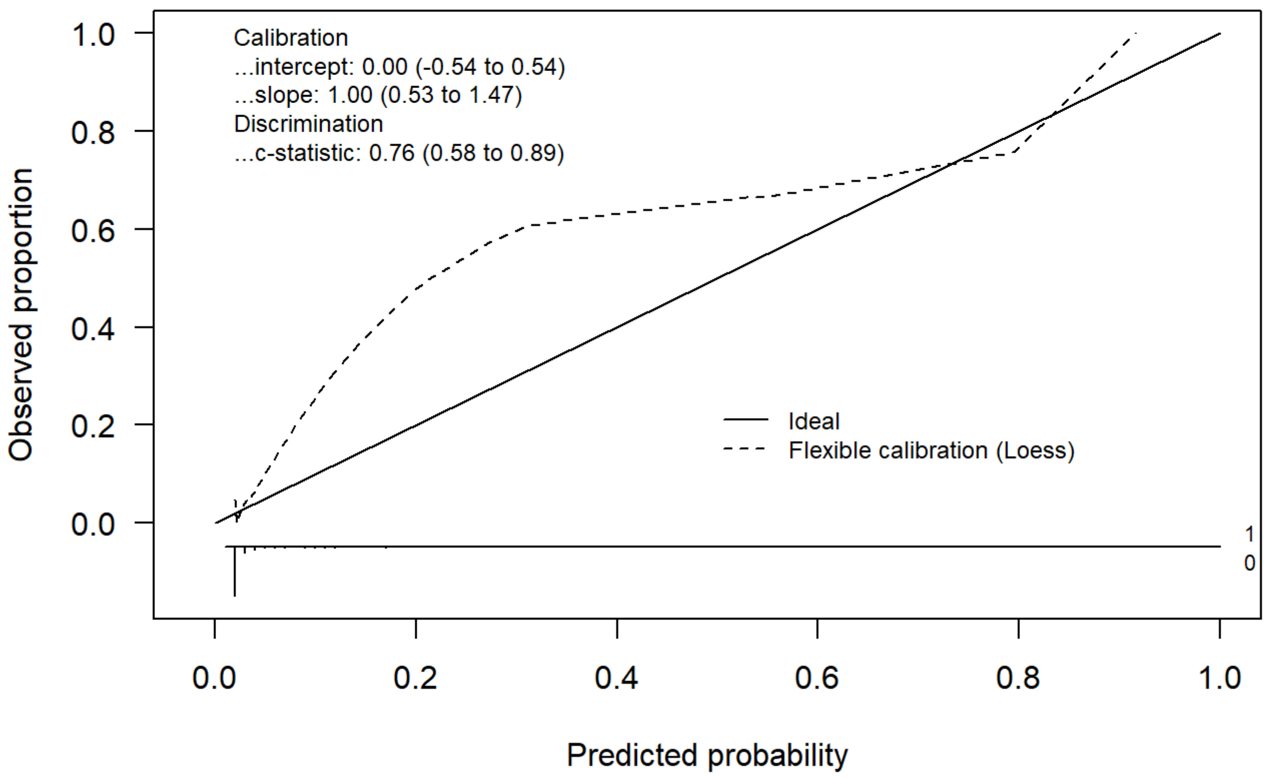


```
## Call:
## CalibrationCurves::val.prob.ci.2(p = pltdeathih.imp.pred[, 1],
##     y = test.org.na$DEATH_INHOSPITAL, CL.smooth = FALSE, lty.smooth = 2,
##     col.ideal = "black")
##
## A 95% confidence interval is given for the calibration intercept, calibration slope and c-statist
ic.
##
##           Dxy        C (ROC)            R2             D      D:Chi-sq
##  5.290625e-01  7.645312e-01  1.853847e-01  5.055333e-02  2.203019e+01
##           D:p              U      U:Chi-sq           U:p             Q
##  2.683961e-06 -4.807692e-03 -2.842171e-14  1.000000e+00  5.536102e-02
##         Brier      Intercept         Slope          Emax  Brier scaled
##  3.253849e-02  1.007316e-07  1.000000e+00  2.880529e-11  1.201591e-01
##          Eavg            ECI
##  2.336015e-02  2.697789e-01
```

Isoregression model shows perfect fit?!
Going on to the validation

8.6 Validation

8.6.1 Original data analysis: Validation

```
# Remove NA
valid.org.na <- na.omit(valid.org)

# Predicted probability and classification
lrdeathih.org.valid.pred <- predict(lrdeathih.org.final
                                , newdata = valid.org.na
                                , type = "response")

lrdeathih.org.valid.class <- ifelse(lrdeathih.org.valid.pred>=0.5, 1, 0)

# Confusion matrix
cl_table.lrdeathih.org.valid <- table (valid.org.na$DEATH_INHOSPITAL, lrdeathih.org.valid.class)
rownames(cl_table.lrdeathih.org.valid) <- c("Alive", "Death in-hospital")
colnames(cl_table.lrdeathih.org.valid) <- c("Alive", "Death in-hospital")
addmargins(cl_table.lrdeathih.org.valid)
```

```
##                      lrdeathih.org.valid.class
##                       Alive Death in-hospital Sum
##   Alive                 478                 0 478
##   Death in-hospital      18                 2  20
##   Sum                   496                 2 498
```

```
# Model accuracy (classification accuracy)
mean((valid.org.na$DEATH_INHOSPITAL == lrdeathih.org.valid.class))
```

```
## [1] 0.9638554
```

```
# Discriminability
roc.lrdeathih.org.valid <- roc(valid.org.na$DEATH_INHOSPITAL
                           ,lrdeathih.org.valid.pred
                           , add = TRUE)
```

```
## Setting levels: control = 0, case = 1
```

```
## Setting direction: controls < cases
```

```
roc.lrdeathih.org.valid$auc
```

```
## Area under the curve: 0.8218
```

```
ci(roc.lrdeathih.org.valid)
```

```
## 95% CI: 0.7393-0.9042 (DeLong)
```

```
pROC::coords(roc.lrdeathih.org.valid,
             "best",
             transpose = TRUE,
             best.method = "youden")
```

```
##   threshold specificity sensitivity
##  0.03032495  0.67782427  0.85000000
```

```
plot.roc(roc.lrdeathih.org.valid
         , print.auc = TRUE
         , print.thres = FALSE
         , auc.polygon = TRUE
)
```

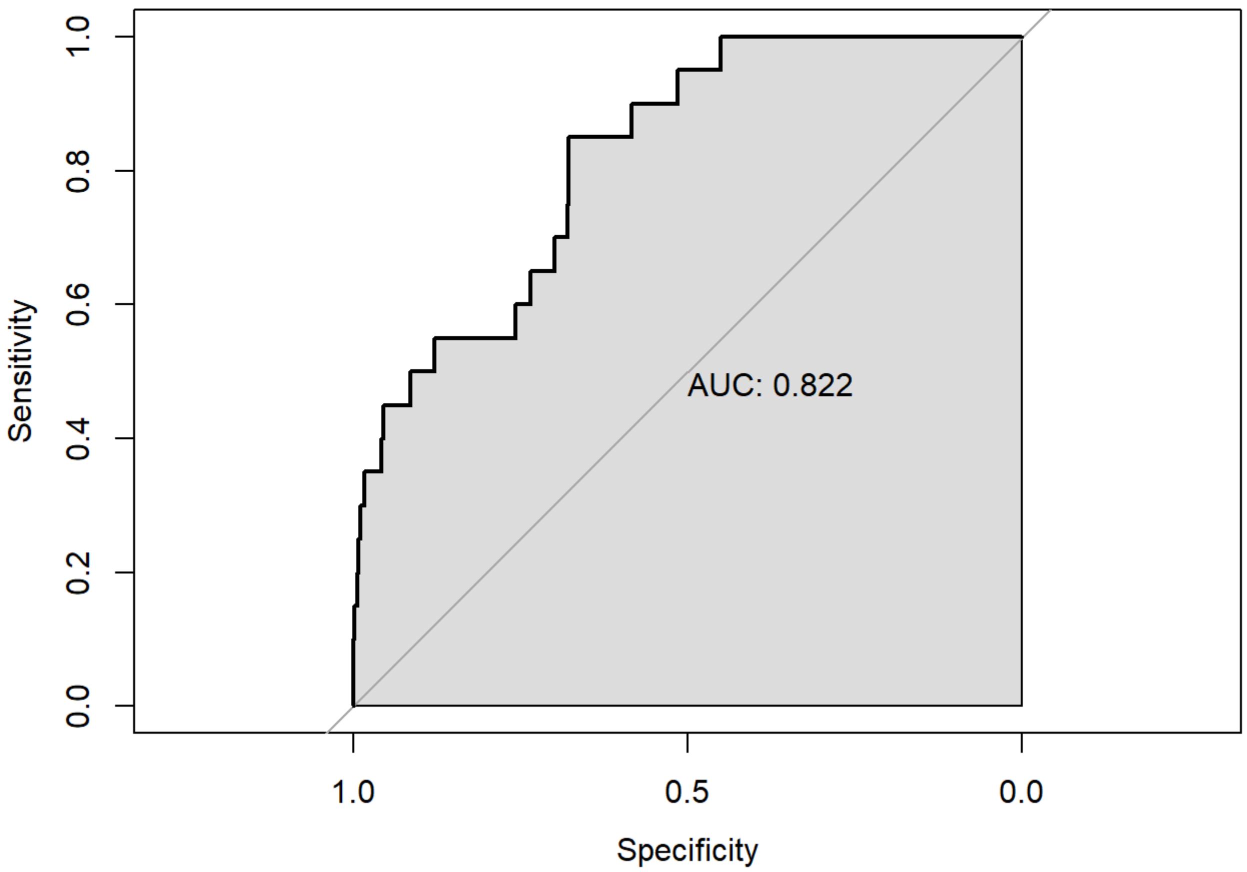


```
#Threshold discrimination ability

lrdeathih.org.valid.pred.dcs <- ifelse(lrdeathih.org.valid.pred > 0.02957133, 1, 0)

roc.lrdeathih.org.valid.dcs <- roc(valid.org.na$DEATH_INHOSPITAL
                               ,lrdeathih.org.valid.pred.dcs
                               , add = TRUE)
```

```
## Setting levels: control = 0, case = 1
## Setting direction: controls < cases
```

```
roc.lrdeathih.org.valid.dcs$auc
```

```
## Area under the curve: 0.7618
```

```
ci(roc.lrdeathih.org.valid.dcs)
```

```
## 95% CI: 0.6788-0.8448 (DeLong)
```

```
pROC::coords(roc.lrdeathih.org.valid.dcs,
             "best",
             transpose = TRUE,
             best.method = "youden")
```

```
##   threshold specificity sensitivity
##   0.5000000   0.6736402   0.8500000
```

```
plot.roc(roc.lrdeathih.org.valid.dcs
         , print.auc = TRUE
         , print.thres = FALSE
         , auc.polygon = TRUE
)
```

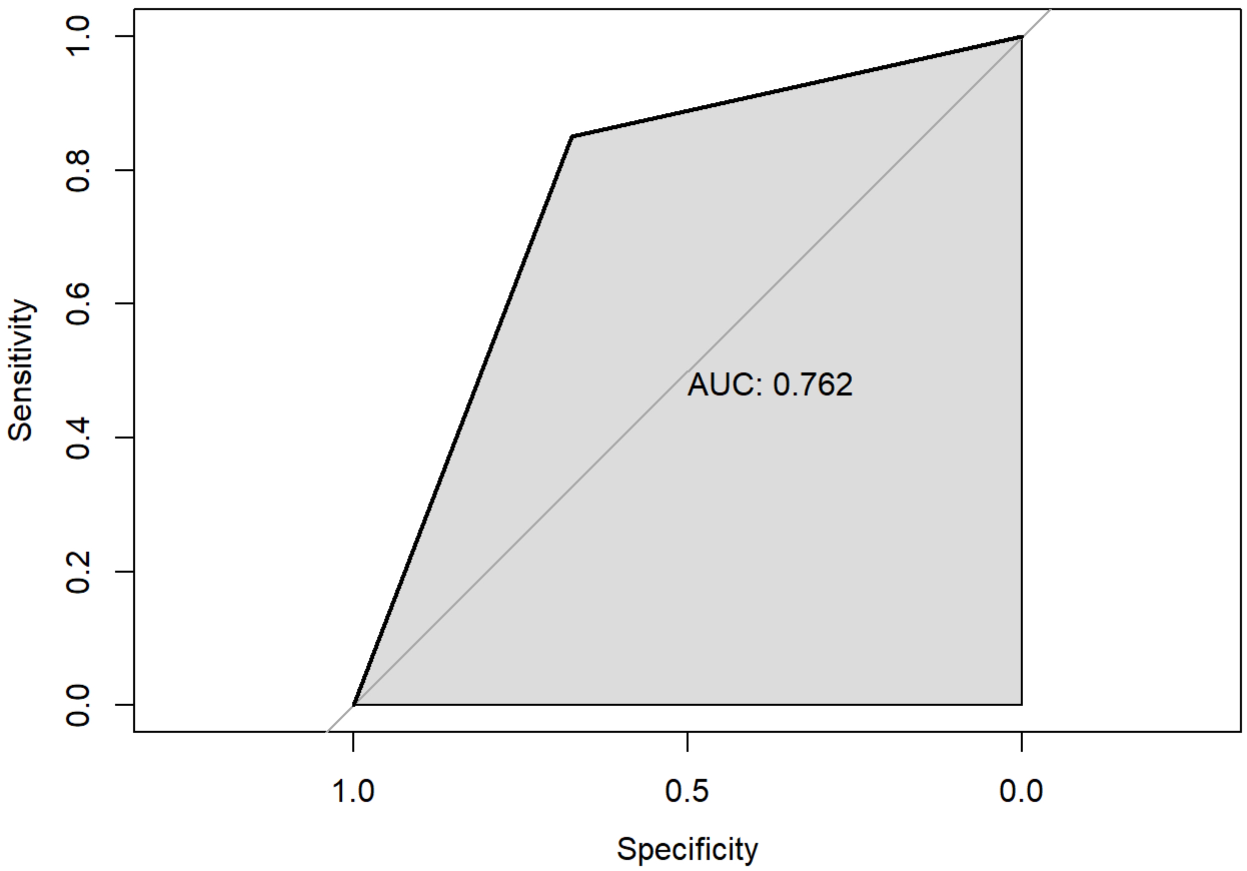

8.6.2 Imputted data analaysis: Validation

```
# Predicted probability and classification: pooled model
lrdeathih.imp.valid.pred <- as.data.frame(predict(lrdeathih.imp, valid.org.na, type = "response"))
lrdeathih.imp.valid.class <- ifelse(lrdeathih.imp.valid.pred>=0.5, 1, 0)

# Confusion matrix
cl_table.lrdeathih.imp.valid <- table (valid.org.na$DEATH_INHOSPITAL, lrdeathih.imp.valid.class)
rownames(cl_table.lrdeathih.imp.valid) <- c("Alive", "Death in-hospital")
colnames(cl_table.lrdeathih.imp.valid) <- c("Alive", "Death in-hospital")
addmargins(cl_table.lrdeathih.imp.valid)
```

```
##                      lrdeathih.imp.valid.class
##                       Alive Death in-hospital Sum
##   Alive                 478                 0 478
##   Death in-hospital      18                 2  20
##   Sum                   496                 2 498
```

```
# Sensitivity
round(cl_table.lrdeathih.imp.valid[2,2]/(cl_table.lrdeathih.imp.valid[2,1] + cl_table.lrdeathih.imp.
valid[2,2]),3)
```

```
## [1] 0.1
```

```
# Specificity
round(cl_table.lrdeathih.imp.valid[1,1]/(cl_table.lrdeathih.imp.valid[1,1] + cl_table.lrdeathih.imp.
valid[1,2]),3)
```

```
## [1] 1
```

```
# Positive predictive rate
round(cl_table.lrdeathih.imp.valid[2,2]/(cl_table.lrdeathih.imp.valid[1,2] + cl_table.lrdeathih.imp.
valid[2,2]),3)
```

```
## [1] 1
```

```
# Negative predictive rate
round(cl_table.lrdeathih.imp.valid[1,1]/(cl_table.lrdeathih.imp.valid[1,1] + cl_table.lrdeathih.imp.
valid[2,1]),3)
```

```
## [1] 0.964
```

```
#model accuracy (classification accuracy)
mean((valid.org.na$DEATH_INHOSPITAL == lrdeathih.imp.valid.class))
```

```
## [1] 0.9638554
```

```
#Discriminability

roc.lrdeathih.imp.valid <- roc(valid.org.na$DEATH_INHOSPITAL
                           , lrdeathih.imp.valid.pred[,1]
                           , add = TRUE)
```

```
## Setting levels: control = 0, case = 1
```

```
## Setting direction: controls < cases
```

```
roc.lrdeathih.imp.valid$auc
```

```
## Area under the curve: 0.7801
```

```
ci(roc.lrdeathih.imp.valid)
```

```
## 95% CI: 0.6676-0.8927 (DeLong)
```

```
pROC::coords(roc.lrdeathih.imp.valid
             , "best"
             , transpose = TRUE
             , best.method="youden"
)
```

```
##   threshold specificity sensitivity
##  0.02813173  0.65690377  0.80000000
```

```
plot.roc(roc.lrdeathih.imp.valid
         , print.auc = TRUE
         , print.thres = FALSE
         , auc.polygon = TRUE
)
```

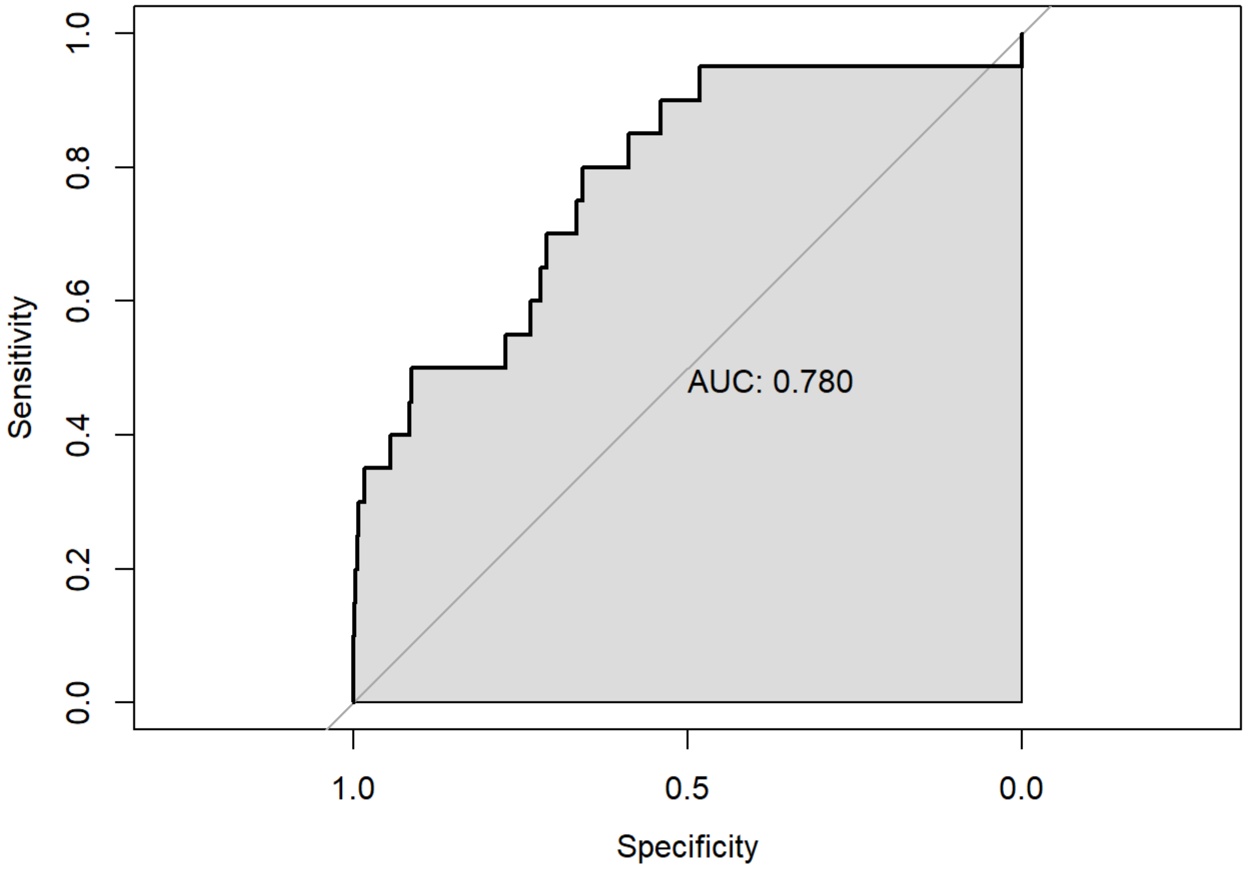


```
# Threshold discrimination ability

lrdeathih.imp.valid.pred.dcs <- ifelse(lrdeathih.imp.valid.pred[,1] > 0.02957133, 1, 0)

roc.lrdeathih.imp.valid.dcs <- roc(valid.org.na$DEATH_INHOSPITAL
                                ,lrdeathih.imp.valid.pred.dcs
                                , add = TRUE)
```

```
## Setting levels: control = 0, case = 1
## Setting direction: controls < cases
```

```
roc.lrdeathih.imp.valid.dcs$auc
```

```
## Area under the curve: 0.7045
```

```
ci(roc.lrdeathih.imp.valid.dcs)
```

```
## 95% CI: 0.6048-0.8041 (DeLong)
```

```
pROC::coords(roc.lrdeathih.imp.valid.dcs,
             "best",
             transpose = TRUE,
             best.method = "youden")
```

```
##   threshold specificity sensitivity
##   0.5000000   0.6589958   0.7500000
```

```
plot.roc(roc.lrdeathih.imp.valid.dcs
         , print.auc = TRUE
         , print.thres = FALSE
         , auc.polygon = TRUE
)
```

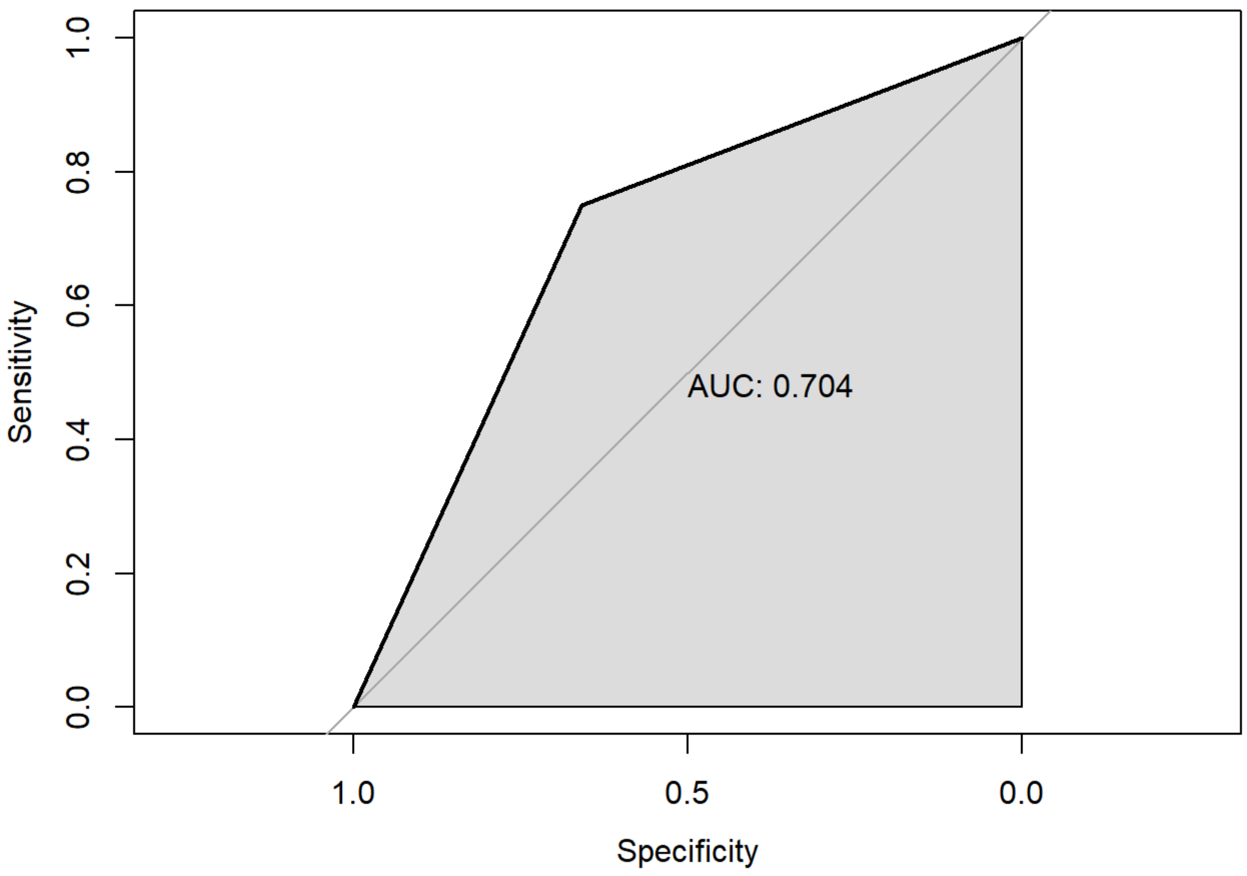

```
# Calibrations: on the validing dataset
cal.lrdeathih.imp.valid <- val.prob(lrdeathih.imp.valid.pred[,1]
                                  , valid.org.na$DEATH_INHOSPITAL
                                  , pl = FALSE)

cal.lrdeathih.imp.valid
```

```
##         Dxy     C (ROC)          R2           D    D:Chi-sq         D:p
##  0.559937238  0.779968619  0.057248366  0.014502031  8.222011513          NA
##           U    U:Chi-sq         U:p           Q       Brier   Intercept
##  0.008583986  6.274825059  0.043394936  0.005918045  0.033230957 -1.050237183
##       Slope        Emax         E90        Eavg         S:z         S:p
##  0.592071227  0.245421131  0.028252303  0.014949578  0.196711561  0.844053263
```

```
hosmer_lemeshow(valid.org.na$DEATH_INHOSPITAL, lrdeathih.imp.valid.pred[,1], 10, 'C')
```

```
## PVALUE 0.1917318
## stat 11.17955
```

```
## [1] 0.1917318
```

```
hosmer_lemeshow(valid.org.na$DEATH_INHOSPITAL, lrdeathih.imp.valid.pred[,1], 10, 'H')
```

```
## PVALUE 0.736178
## stat 5.198378
```

```
## [1] 0.736178
```

```
CalibrationCurves::val.prob.ci.2(lrdeathih.imp.valid.pred[,1], valid.org.na$DEATH_INHOSPITAL
                                   , lty.smooth = 2
                                   , CL.smooth = FALSE
                                   , col.ideal = "black")
```

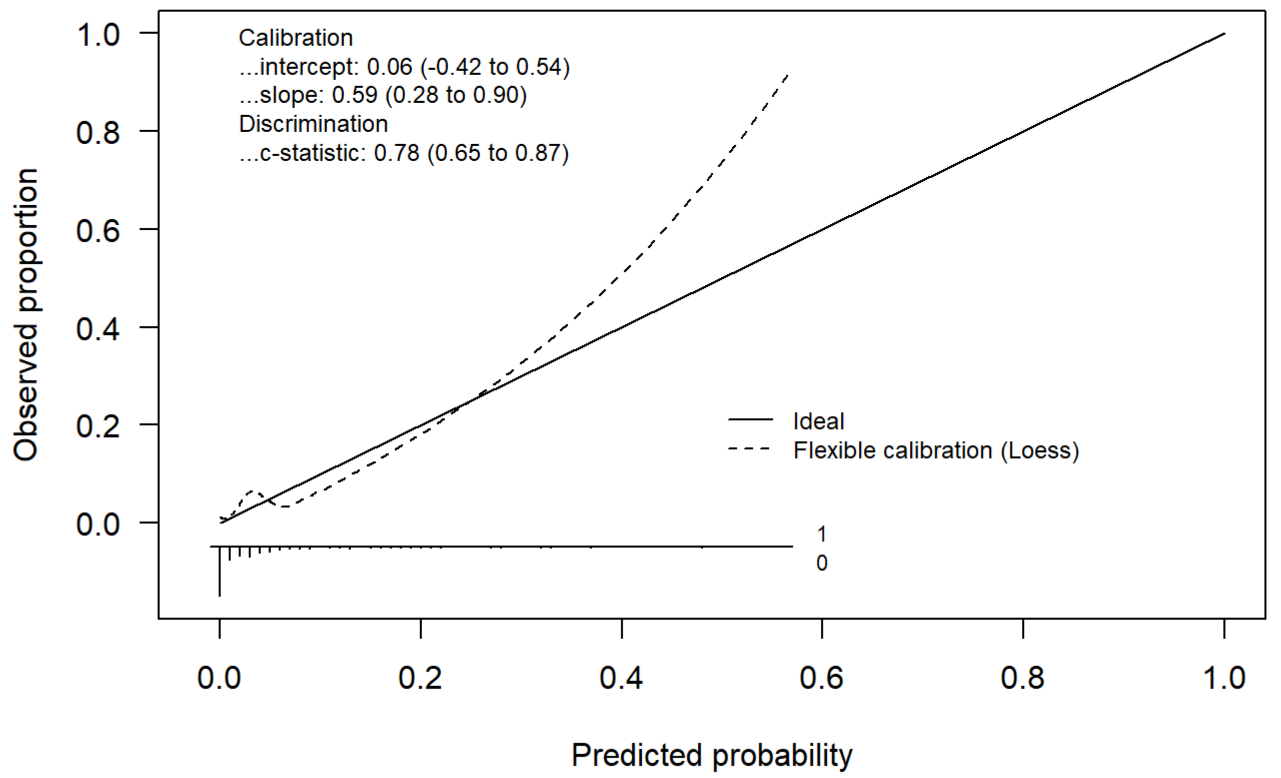


```
## Call:
## CalibrationCurves::val.prob.ci.2(p = lrdeathih.imp.valid.pred[,
##     1], y = valid.org.na$DEATH_INHOSPITAL, CL.smooth = FALSE,
##     lty.smooth = 2, col.ideal = "black")
##
## A 95% confidence interval is given for the calibration intercept, calibration slope and c-statist
ic.
##
##          Dxy      C (ROC)           R2            D     D:Chi-sq          D:p
## 5.601464e-01 7.800732e-01 1.003073e-01 2.710208e-02 1.449684e+01 1.403951e-04
##            U     U:Chi-sq          U:p            Q        Brier    Intercept
## 8.583986e-03 6.274825e+00 4.339494e-02 1.851810e-02 3.323096e-02 6.020855e-02
##        Slope         Emax Brier scaled         Eavg          ECI
## 5.920712e-01 3.580401e-01 1.379278e-01 1.550056e-02 8.669485e-02
```

8.6.3 Isoregression model validation

```
# Predicted probability and classification: isoregression model
isodeathih.imp.valid.pred.prob <- predict(lrdeathih.imp,
                                       valid.org.na[,-length(valid.org.na)],
                                       type = "response")
isodeathih.imp.valid.pred <- isodeathih.imp(as.data.frame(isodeathih.imp.valid.pred.prob)[,1])
isodeathih.imp.valid.class <- ifelse(isodeathih.imp.valid.pred>=0.5, 1, 0)

# Confusion matrix
cl_table.isodeathih.imp.valid <- table (valid.org.na$DEATH_INHOSPITAL, isodeathih.imp.valid.class)
rownames(cl_table.isodeathih.imp.valid) <- c("Alive", "Death in-hospital")
colnames(cl_table.isodeathih.imp.valid) <- c("Alive", "Death in-hospital")
addmargins(cl_table.isodeathih.imp.valid)
```

```
##                     isodeathih.imp.valid.class
##                      Alive Death in-hospital Sum
##   Alive               478                 0 478
##   Death in-hospital    18                 2  20
##   Sum                 496                 2 498
```

```
# Sensitivity
round(cl_table.isodeathih.imp.valid[2,2]/(cl_table.isodeathih.imp.valid[2,1] + cl_table.isodeathih.i
mp.valid[2,2]),3)
```

```
## [1] 0.1
```

```
# Specificity
round(cl_table.isodeathih.imp.valid[1,1]/(cl_table.isodeathih.imp.valid[1,1] + cl_table.isodeathih.i
mp.valid[1,2]),3)
```

```
## [1] 1
```

```
# Positive predictive rate
round(cl_table.isodeathih.imp.valid[2,2]/(cl_table.isodeathih.imp.valid[1,2] + cl_table.isodeathih.i
mp.valid[2,2]),3)
```

```
## [1] 1
```

```
# Negative predictive rate
round(cl_table.isodeathih.imp.valid[1,1]/(cl_table.isodeathih.imp.valid[1,1] + cl_table.isodeathih.i
mp.valid[2,1]),3)
```

```
## [1] 0.964
```

```
#model accuracy (classification accuracy)
mean((valid.org.na$DEATH_INHOSPITAL == isodeathih.imp.valid.class))
```

```
## [1] 0.9638554
```

```
#Discriminability

roc.isodeathih.imp.valid <- roc(valid.org.na$DEATH_INHOSPITAL
                            , isodeathih.imp.valid.pred
                            , add = TRUE)
```

```
## Setting levels: control = 0, case = 1
```

```
## Setting direction: controls < cases
```

```
roc.isodeathih.imp.valid$auc
```

```
## Area under the curve: 0.7748
```

```
ci(roc.isodeathih.imp.valid)
```

```
## 95% CI: 0.6648-0.8848 (DeLong)
```

```
pROC::coords(roc.isodeathih.imp.valid
             , "best"
             , transpose = TRUE
             , best.method="youden"
)
```

```
##   threshold specificity sensitivity
##  0.02948718  0.65690377  0.80000000
```

```
plot.roc(roc.isodeathih.imp.valid
         , print.auc = TRUE
         , print.thres = FALSE
         , auc.polygon = TRUE
)
```

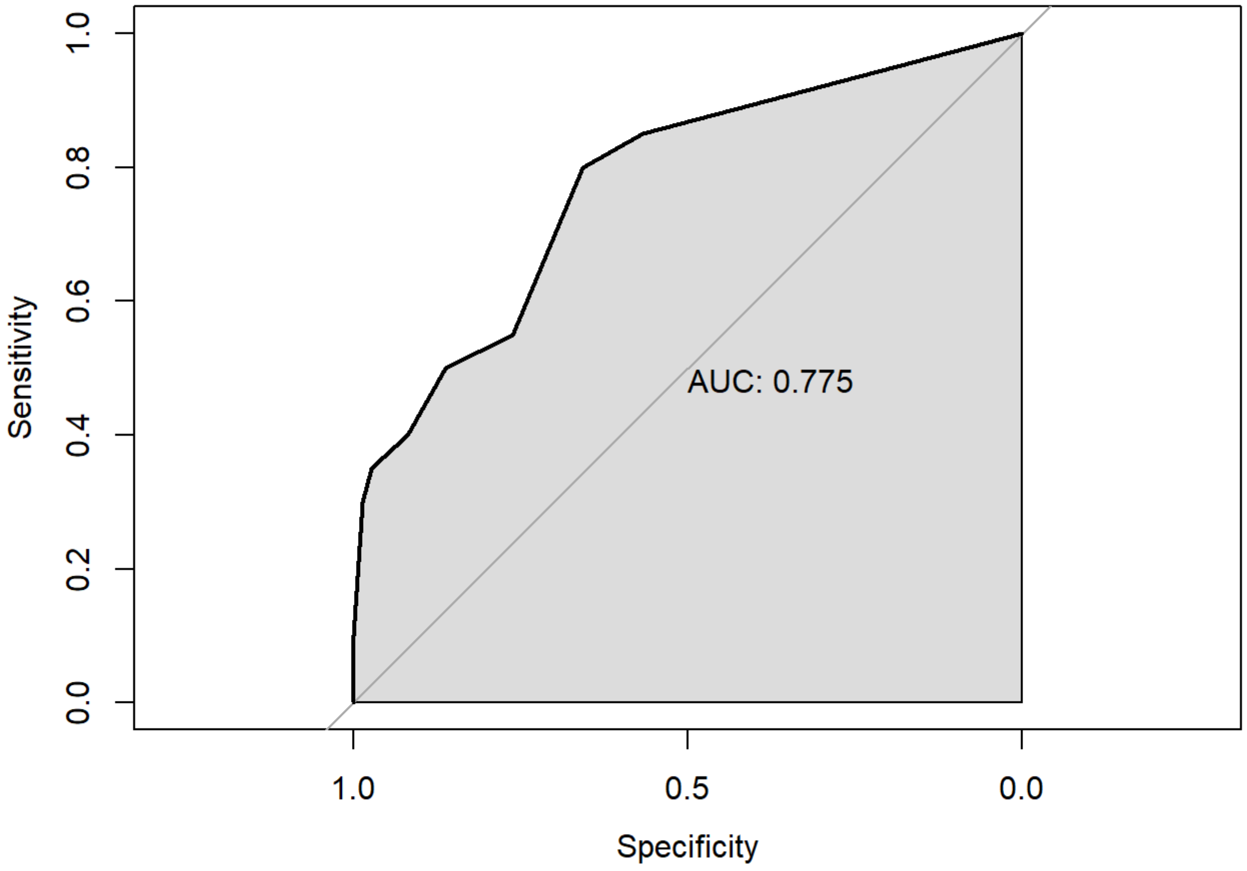


```
# Threshold discrimination ability

isodeathih.imp.valid.pred.dcs <- ifelse(isodeathih.imp.valid.pred > 0.02957133, 1, 0)

roc.isodeathih.imp.valid.dcs <- roc(valid.org.na$DEATH_INHOSPITAL
                                ,isodeathih.imp.valid.pred.dcs
                                , add = TRUE)
```

```
## Setting levels: control = 0, case = 1
## Setting direction: controls < cases
```

```
roc.lrdeathih.imp.valid.dcs$auc
```

```
## Area under the curve: 0.7045
```

```
ci(roc.lrdeathih.imp.valid.dcs)
```

```
## 95% CI: 0.6048-0.8041 (DeLong)
```

```
pROC::coords(roc.lrdeathih.imp.valid.dcs,
             "best",
             transpose = TRUE,
             best.method = "youden")
```

```
##   threshold specificity sensitivity
##   0.5000000   0.6589958   0.7500000
```

```
plot.roc(roc.lrdeathih.imp.valid.dcs
         , print.auc = TRUE
         , print.thres = FALSE
         , auc.polygon = TRUE
)
```

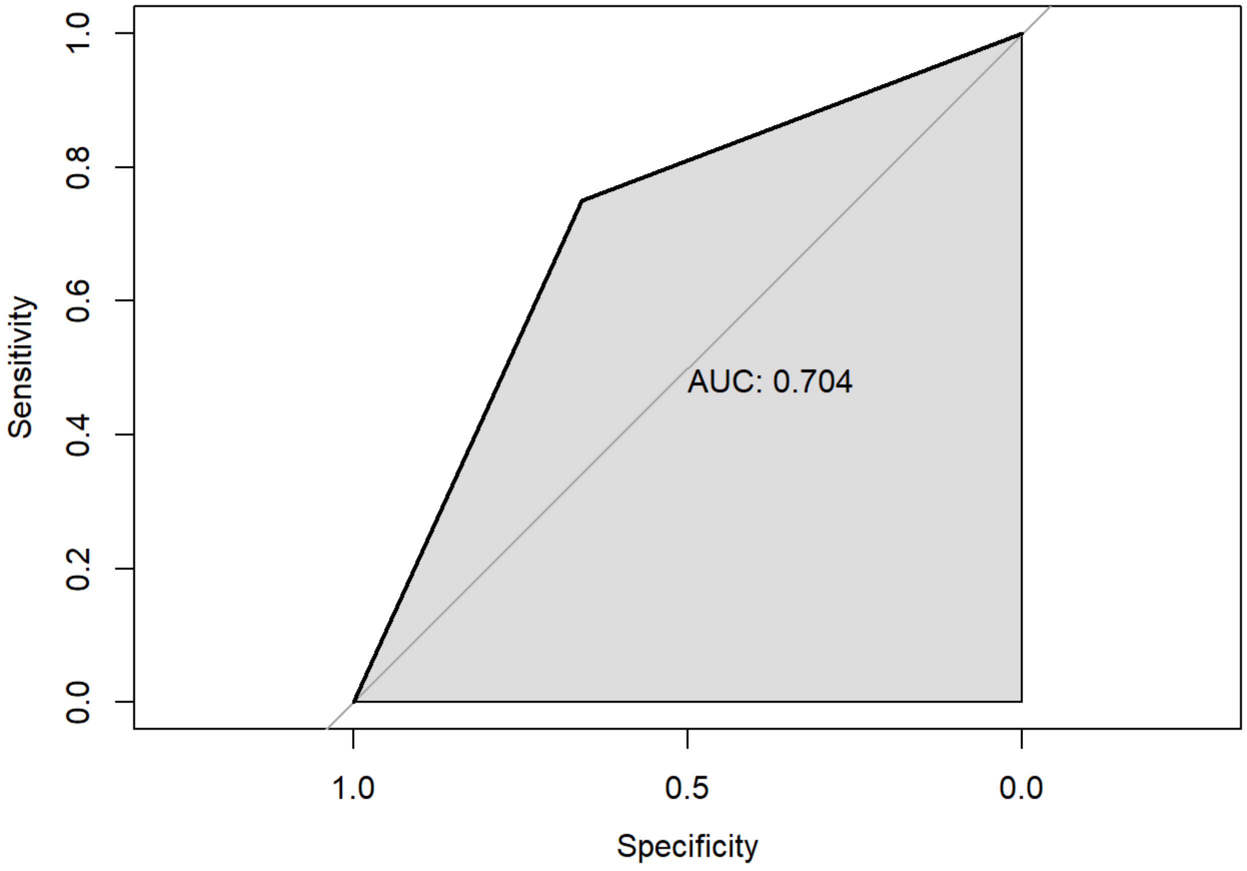

```
# Calibrations: on the validing dataset
cal.isodeathih.imp.valid <- val.prob(isodeathih.imp.valid.pred
                                   , valid.org.na$DEATH_INHOSPITAL
                                   , pl = FALSE)

cal.isodeathih.imp.valid
```

```
##          Dxy      C (ROC)           R2            D     D:Chi-sq          D:p
##  0.499535100  0.749767550  0.128889242  0.033133448 17.434190393           NA
##            U     U:Chi-sq          U:p            Q        Brier    Intercept
## -0.003122323  0.451327926  0.797986209  0.036255771  0.032706672 -0.405207491
##        Slope         Emax          E90         Eavg          S:z          S:p
##  0.872035880  0.085265585  0.033752199  0.010050928 -0.133301027  0.893955315
```

```
hosmer_lemeshow(valid.org.na$DEATH_INHOSPITAL, isodeathih.imp.valid.pred, 10, 'C')
```

```
## PVALUE 0.5139738
## stat 7.211699
```

```
## [1] 0.5139738
```

```
hosmer_lemeshow(valid.org.na$DEATH_INHOSPITAL, isodeathih.imp.valid.pred, 10, 'H')
```

```
## PVALUE 0.9708033
## stat 2.290058
```

```
## [1] 0.9708033
```

```
CalibrationCurves::val.prob.ci.2(isodeathih.imp.valid.pred, valid.org.na$DEATH_INHOSPITAL
                                   , lty.smooth = 2
                                   , CL.smooth = FALSE
                                   , col.ideal = "black")
```

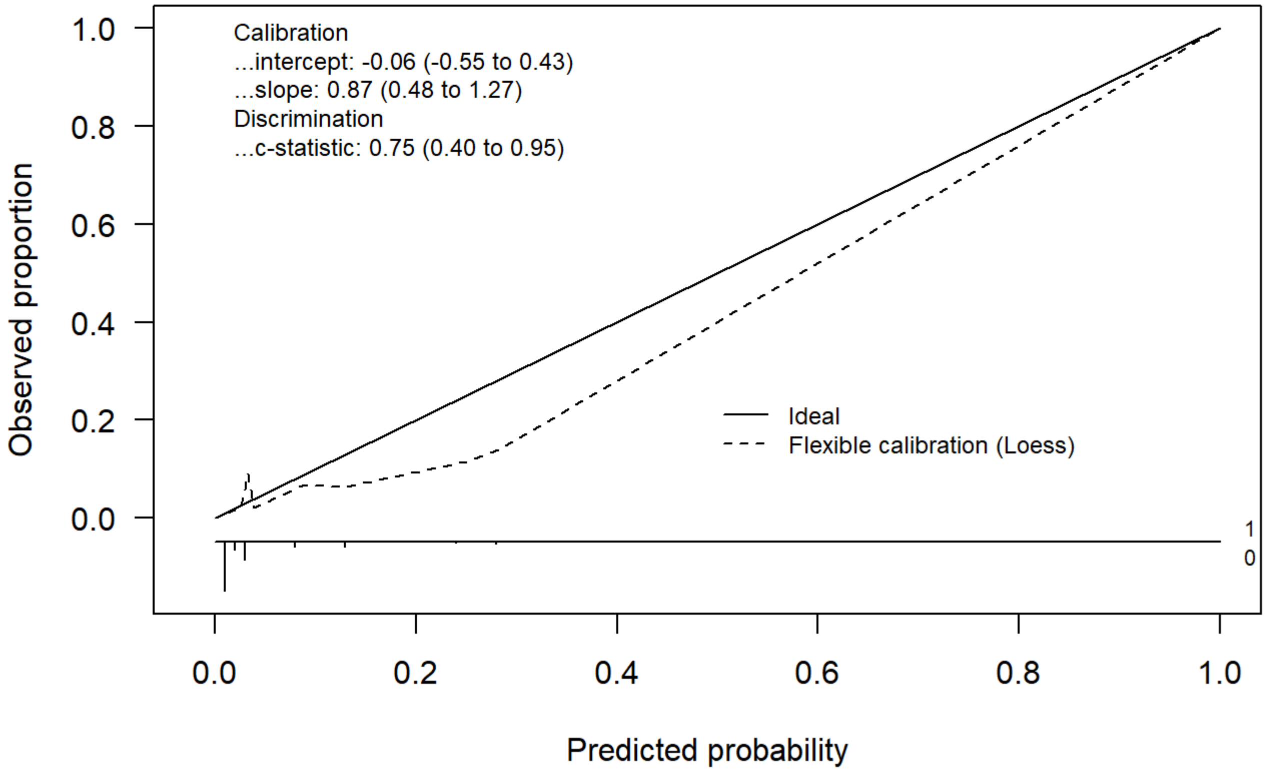


```
## Call:
## CalibrationCurves::val.prob.ci.2(p = isodeathih.imp.valid.pred,
##     y = valid.org.na$DEATH_INHOSPITAL, CL.smooth = FALSE, lty.smooth = 2,
##     col.ideal = "black")
##
## A 95% confidence interval is given for the calibration intercept, calibration slope and c-statist
ic.
##
##           Dxy       C (ROC)            R2             D      D:Chi-sq
##  4.995351e-01  7.497675e-01  1.321661e-01  3.404338e-02  1.788552e+01
##           D:p             U      U:Chi-sq           U:p             Q
##  2.345995e-05 -3.122323e-03  4.513279e-01  7.979862e-01  3.716571e-02
##         Brier     Intercept         Slope          Emax  Brier scaled
##  3.283855e-02 -5.772353e-02  8.720359e-01  1.173880e-01  1.481074e-01
##          Eavg           ECI
##  1.867939e-02  1.384978e-01
```

8.6.4 Platt scaling model validation

```
# Predicted probability and classification: Platt scaling model
colnames(lrdeathih.imp.valid.pred) <- c("yhat")
pltdeathih.imp.valid.pred <- as.data.frame(predict(pltdeathih.imp, lrdeathih.imp.valid.pred, type =
"response" ))
pltdeathih.imp.valid.class <- ifelse(pltdeathih.imp.valid.pred>=0.5, 1, 0)


# Confusion matrix
cl_table.pltdeathih.imp.valid <- table (valid.org.na$DEATH_INHOSPITAL, pltdeathih.imp.valid.class)
rownames(cl_table.pltdeathih.imp.valid) <- c("Alive", "Death in-hospital")
colnames(cl_table.pltdeathih.imp.valid) <- c("Alive", "Death in-hospital")
addmargins(cl_table.pltdeathih.imp.valid)
```

```
##                    pltdeathih.imp.valid.class
##                     Alive Death in-hospital Sum
##   Alive               476                 2 478
##   Death in-hospital    17                 3  20
##   Sum                 493                 5 498
```

```
# Sensitivity
round(cl_table.pltdeathih.imp.valid[2,2]/(cl_table.pltdeathih.imp.valid[2,1] + cl_table.pltdeathih.i
mp.valid[2,2]),3)
```

```
## [1] 0.15
```

```
# Specificity
round(cl_table.pltdeathih.imp.valid[1,1]/(cl_table.pltdeathih.imp.valid[1,1] + cl_table.pltdeathih.i
mp.valid[1,2]),3)
```

```
## [1] 0.996
```

```
# Positive predictive rate
round(cl_table.pltdeathih.imp.valid[2,2]/(cl_table.pltdeathih.imp.valid[1,2] + cl_table.pltdeathih.i
mp.valid[2,2]),3)
```

```
## [1] 0.6
```

```
# Negative predictive rate
round(cl_table.pltdeathih.imp.valid[1,1]/(cl_table.pltdeathih.imp.valid[1,1] + cl_table.pltdeathih.i
mp.valid[2,1]),3)
```

```
## [1] 0.966
```

```
#model accuracy (classification accuracy)
mean((valid.org.na$DEATH_INHOSPITAL == pltdeathih.imp.valid.class))
```

```
## [1] 0.9618474
```

```
#Discriminability

roc.pltdeathih.imp.valid <- roc(valid.org.na$DEATH_INHOSPITAL
                            , pltdeathih.imp.valid.pred[,1]
                            , add = TRUE)
```

```
## Setting levels: control = 0, case = 1
```

```
## Setting direction: controls < cases
```

```
roc.pltdeathih.imp.valid$auc
```

```
## Area under the curve: 0.7801
```

```
ci(roc.pltdeathih.imp.valid)
```

```
## 95% CI: 0.6676-0.8927 (DeLong)
```

```
pROC::coords(roc.pltdeathih.imp.valid
             , "best"
             , transpose = TRUE
             , best.method="youden"
)
```

```
##   threshold specificity sensitivity
##  0.02745647  0.65690377  0.80000000
```

```
plot.roc(roc.pltdeathih.imp.valid
         , print.auc = TRUE
         , print.thres = FALSE
         , auc.polygon = TRUE
)
```

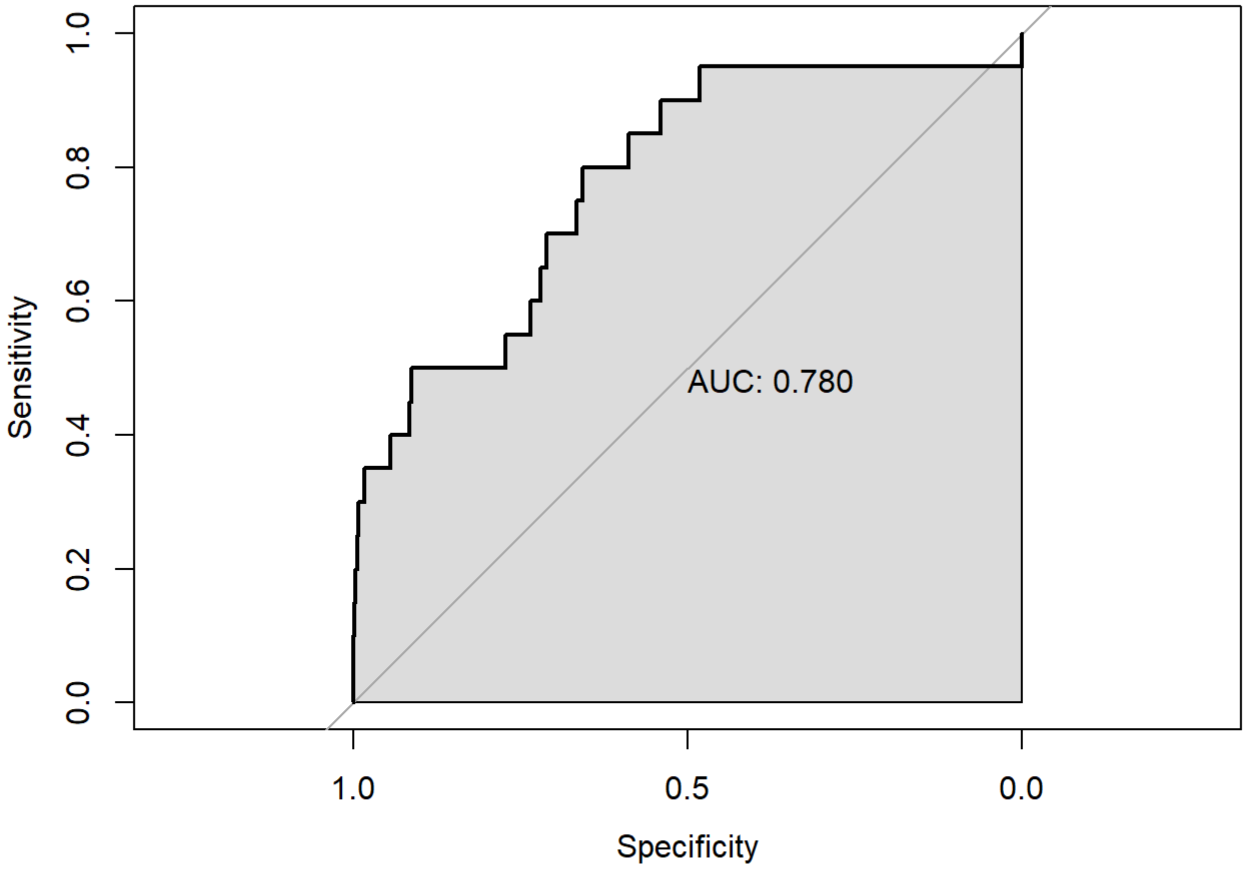


```
# Threshold discrimination ability

pltdeathih.imp.valid.pred.dcs <- ifelse(pltdeathih.imp.valid.pred > 0.02957133, 1, 0)

roc.pltdeathih.imp.valid.dcs <- roc(valid.org.na$DEATH_INHOSPITAL
                                    ,pltdeathih.imp.valid.pred.dcs[,1]
                                    , add = TRUE)
```

```
## Setting levels: control = 0, case = 1
## Setting direction: controls < cases
```

```
roc.lrdeathih.imp.valid.dcs$auc
```

```
## Area under the curve: 0.7045
```

```
ci(roc.lrdeathih.imp.valid.dcs)
```

```
## 95% CI: 0.6048-0.8041 (DeLong)
```

```
pROC::coords(roc.lrdeathih.imp.valid.dcs,
             "best",
             transpose = TRUE,
             best.method = "youden")
```

```
##   threshold specificity sensitivity
##   0.5000000   0.6589958   0.7500000
```

```
plot.roc(roc.lrdeathih.imp.valid.dcs
         , print.auc = TRUE
         , print.thres = FALSE
         , auc.polygon = TRUE
)
```

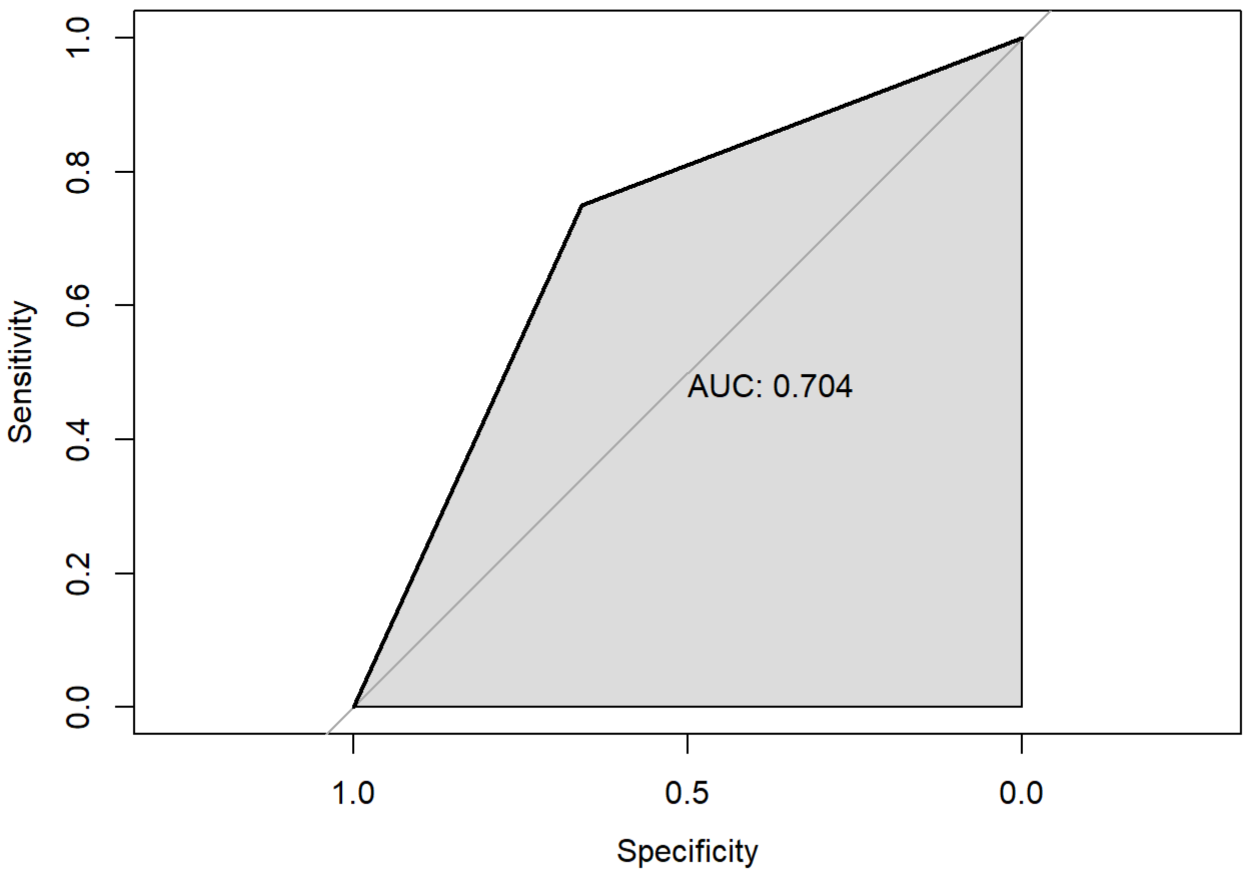

```
# Calibrations: on the validing dataset
cal.pltdeathih.imp.valid <- val.prob(pltdeathih.imp.valid.pred[,1]
                                   , valid.org.na$DEATH_INHOSPITAL
                                   , pl = FALSE)

cal.pltdeathih.imp.valid
```

```
##          Dxy      C (ROC)           R2            D     D:Chi-sq          D:p
##  0.560669456  0.780334728  0.201454613  0.057339983 29.555311743           NA
##            U     U:Chi-sq          U:p            Q        Brier    Intercept
## -0.003477026  0.268441093  0.874397198  0.060817009  0.032506417 -0.245745452
##        Slope         Emax          E90         Eavg          S:z          S:p
##  0.949547307  0.087200281  0.023696661  0.014929661 -0.233374520  0.815470597
```

```
hosmer_lemeshow(valid.org.na$DEATH_INHOSPITAL, pltdeathih.imp.valid.pred[,1], 10, 'C')
```

```
## PVALUE 0.2463007
## stat 10.27428
```

```
## [1] 0.2463007
```

```
hosmer_lemeshow(valid.org.na$DEATH_INHOSPITAL, pltdeathih.imp.valid.pred[,1], 10, 'H')
```

```
## PVALUE 0.5694543
## stat 6.698769
```

```
## [1] 0.5694543
```

```
CalibrationCurves::val.prob.ci.2(pltdeathih.imp.valid.pred[,1], valid.org.na$DEATH_INHOSPITAL
                                   , lty.smooth = 2
                                   , CL.smooth = FALSE
                                   , col.ideal = "black")
```

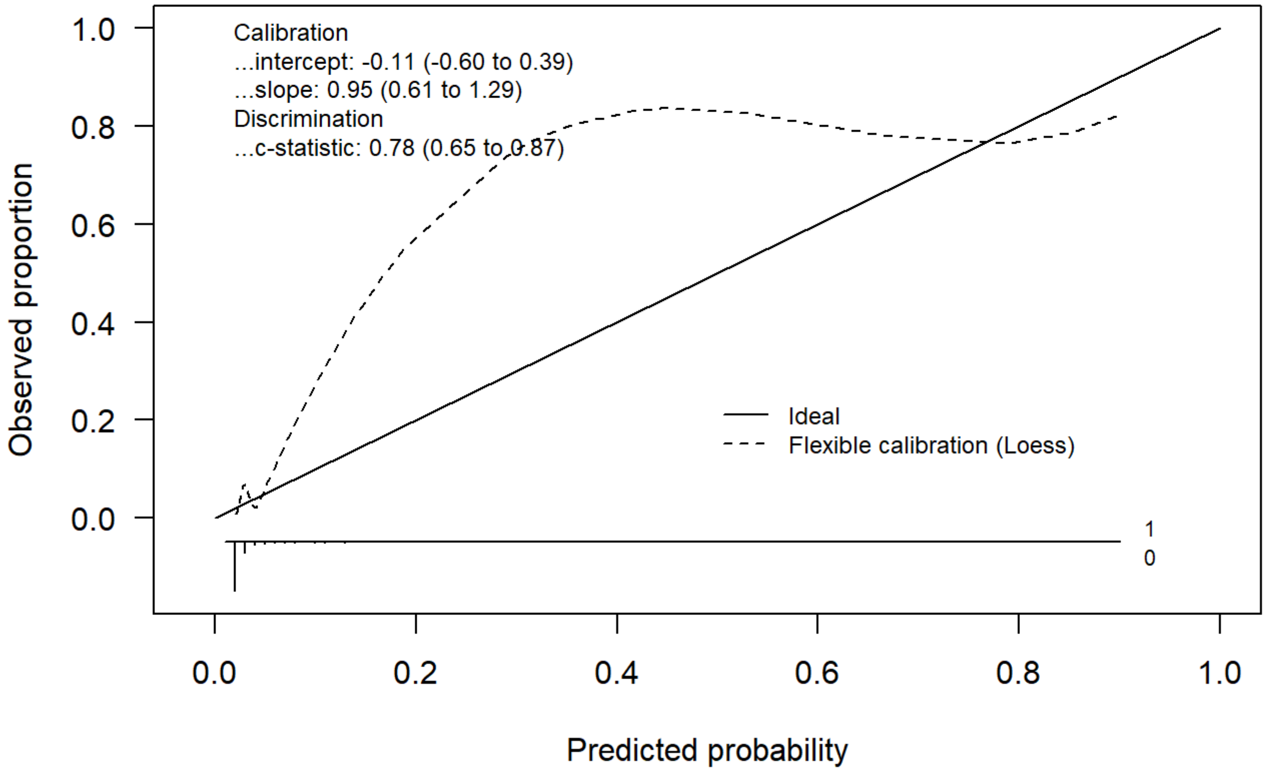


```
## Call:
## CalibrationCurves::val.prob.ci.2(p = pltdeathih.imp.valid.pred[,
##     1], y = valid.org.na$DEATH_INHOSPITAL, CL.smooth = FALSE,
##     lty.smooth = 2, col.ideal = "black")
##
## A 95% confidence interval is given for the calibration intercept, calibration slope and c-statist
ic.
##
##           Dxy       C (ROC)            R2             D      D:Chi-sq
##  5.621339e-01  7.810669e-01  2.032301e-01  5.787902e-02  2.982375e+01
##           D:p             U      U:Chi-sq           U:p             Q
##  4.731593e-08 -3.477026e-03  2.684411e-01  8.743972e-01  6.135605e-02
##         Brier     Intercept         Slope          Emax  Brier scaled
##  3.250642e-02 -1.073863e-01  9.495473e-01  6.545893e-02  1.567237e-01
##          Eavg           ECI
##  3.208313e-02  5.765884e-01
```

9 References: Interpretation of the calibrations

R package “rms” provides val.prob function which calculates various calibrations.
First, this function prints out a calibration plot; the dashed line indicates a ideal line of calibration, the solid line as a logistic regression fitted to the actual results and the predicted probabilities. The dotted line is a non-parametric fit of the actual and predicted probability.
Logistic regression has two parameters, an intercept and a slope, so it sometimes hard to fit all the twists and turns of the underlying data. Non-parametric fit provides a better fit to the actual data but is highly non-linear and is not constrained to the interval of 0~1. So, risk of overfitting.
The ticks at the bottom of the chart indicate the relative prevalence of predictions ot the relevant probability.

Dxy: corrlation between probabilities and observations.+1 is perfect, postive and higher is better.
C (ROC): AUC of relevant ROC curve.1 is perfect and 0.5 is prediction at random.
R2: a pseudo-R-squared measrue. +1 is perfect and 0 reflects randomness.
D: Discrimination index. 1 is perfect and 0 is randomness.
Brier: the Brier score. The mean squared error between the acutal outcomes and the estimated probailities. ranging 0 ~ 1. smaller is better.
S:z : Spiegelhalter Z-test for calibration accuracy. The null hypothensis is “the actual probability is equal to the predicted probability for all actual probability.” Fail to reject the null hypothesis is better.
Hosmer-Lemeshow test: A P value of .1 or higher is considered appropriate, P value <.1 and >.05 indicates that the model is neigher well calibrated nor grossly miscalibrate, and a P value <.05 indicates micalibrated estimates.
